# Prion 2022 Conference abstracts: pushing the boundaries

**DOI:** 10.1080/19336896.2022.2091286

**Published:** 2022-09-11

**Authors:** Inga Zerr

**Affiliations:** *On behalf of the PRION2020/2022 Organizing Committee*Jean-Philippe DeslysMarkus GlatzelMathias JuckerNoriyuki NishidaTiago OuteiroJesus Rodríguez RequenaHermann Schaetzl

**Combining vaccination with genetic resistance to protect caribou against CWD**

Dalia Abdelaziz, Hanaa Ahmed Hassan, Byron Kruger, Kevin Low, Mariam Ansari, Maria Arifin, Sabine Gilch and Hermann M. Schätzl

Department of Comparative Biology and Experimental Medicine, Faculty of Veterinary Medicine & Hotchkiss Brain Institute, University of Calgary, Calgary, Canada

**Aims**: Chronic wasting disease (CWD) in cervids and BSE in cattle are prion diseases that negatively affect economy, ecology as well as animal and possibly human health in Canada and elsewhere. CWD incidence is reaching 15% in mule deer in Alberta hunting areas doing testing, a tenfold increase over the last 10 years. Caribou are still free of CWD, but transmission into caribou will happen soon. Caribou are a major food source for Native and Northern populations, so food security and safety are at risk. We described genetic factors in the prion protein (PrP) that likely provide caribou relative resistance to CWD. Based on our previous work, we propose that vaccination combined with relative genetic resistance creates additive effects. Our long-term goal is to develop a CWD vaccine to protect caribou and other cervids against CWD.

**Figure:**
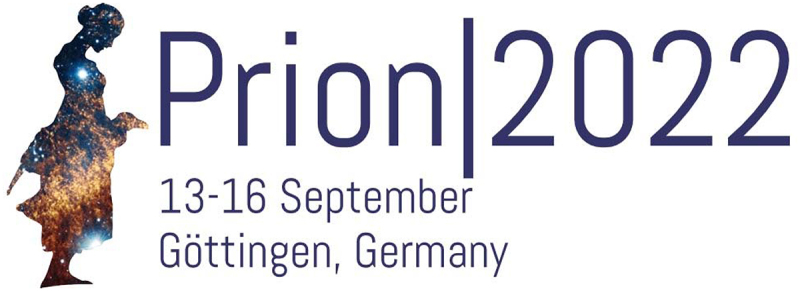

**Material and Methods**: We have established a solid proof-of-concept in rodent models that vaccination provides relative protection in CWD challenge models. We recently developed knock-in (KI) mice with different PrP genotypes that recapitulate CWD pathogenesis as found in cervids. Recombinant prion proteins in monomeric and dimeric form will be expressed in E.coli, purified and used as immunogens, with CpG as adjuvant. In addition, immunogens and adjuvant will be co-encapsulated into nanospheres, to generate nanovaccines for oral vaccination. Mice and reindeer will be immunized as described previously, and blood collected for determining humoral and cellular immune responses. Vaccinated and unvaccinated KI mice will be infected with reindeer-derived CWD, and vaccine effects on incubation time and prion shedding analyzed.

**Results**: The first part of our work analyzes the effect of PrP codon 138 on vaccination followed by CWD challenge in KI mice. Mice were vaccinated and challenge with CWD prions (i.p. route) is ongoing. Vaccination results will be discussed. These experiments will show that vaccination extends time to prion disease in KI mice infected with CWD prions. This work will also analyze whether vaccination reduces CWD shedding. Vaccination studies are done in parallel in reindeer to test vaccination efficacy in cervids and prepare the stage for vaccination of caribou.

Additional work will determine effects of oral vaccination on CWD pathogenesis and prion shedding, using nanovaccines and vector-based vaccines. CWD challenge studies will be done with white-tailed deer in different settings.

**Conclusions**: Our work will result in tools that help to protect caribou and other cervid species against CWD infection.

**Funded by**: The Alberta Prion Research Institute/Alberta Innovates, Canada; Alberta Environment and Parks; National Institutes of Health, USA.

**Grant number**: 212200714, 201600023, 1R01AI156037.

**Acknowledgement**: We thank the University of Calgary animal facilities staff for animal care.

**Cerebrospinal fluid levels of prodynorphin and proenkephalin are differentially altered in sporadic Creutzfeldt-Jakob disease subtypes and reflect the divergent neuronal targeting**

Samir Abu-Rumeileh^a^, Peggy Barschke^b^, Patrick Oeckl^b,c^, Simone Baiardi^d,e^, Angela Mammana^d^, Andrea Mastrangelo^d,f^, MHD Rami Al Shweiki^b^, Petra Steinacker^a^, Anna Ladogana^g^, Sabina Capellari^d,f^, Markus Otto^a^ and Piero Parchi^d,e^

^a^Department of Neurology, Martin-Luther-University Halle-Wittenberg, Halle, Germany; ^b^Department of Neurology, Ulm University Hospital, Ulm, Germany; ^c^German Center for Neurodegenerative Diseases (DZNE e.V.), Ulm, Germany; ^d^IRCCS Istituto delle Scienze Neurologiche di Bologna, Bologna, Italy; ^e^Department of Experimental Diagnostic and Specialty Medicine (DIMES), University of Bologna, Bologna, Italy; ^f^Department of Biomedical and NeuroMotor Sciences (DIBINEM), University of Bologna, Bologna, Italy; ^g^Department of Neuroscience, Istituto Superiore di Sanità, Rome, Italy

**Aims**: Prodynorphin (PDYN) and proenkephalin (PENK) are peptides mainly produced by the striatum and, to a lesser extent, by the cerebral cortex. Previous studies reported dysregulated metabolism and altered cerebrospinal fluid (CSF) levels of PDYN and PDYN in neurodegenerative diseases, such as Huntington’s disease and dementia with Lewy bodies. Here, we investigated, for the first time, CSF PDYN and PENK values in the phenotypic spectrum of sporadic Creutzfeldt-Jakob disease (sCJD).

**Material and Methods**: We measured CSF PDYN- and PENK-derived peptide concentrations in 63 patients with the most prevalent molecular subtypes of sCJD [MM(V)1, VV2 and MV2K], and in 25 controls using a liquid chromatography−multiple reaction monitoring mass spectrometry. Additionally, we performed a semiquantitative analysis of neuronal loss and astrogliosis in sCJD MM(V)1, VV2, and MV2K brains.

**Results**: sCJD MV2K showed selectively decreased CSF PDYN-derived peptides compared to the other subtypes. Accordingly, the semiquantitative analysis of cortical and striatal neuronal loss and astrogliosis revealed a more widespread overall pathology in the MV2K group than the sCJD MM(V)1 and the VV2 groups. We detected decreased levels of one of the PENK-derived peptides in all CJD subtypes, without difference between subtypes. PENK and PDYN were associated with CSF biomarkers of neurodegeneration. However, PENK and PDYN did not correlate with disease stage and survival and yielded a poor diagnostic value in sCJD.

**Conclusions**: Despite the poor diagnostic and prognostic performance of CSF PDYN and PENK-derived peptides, we speculate that the observed differential alteration of CSF peptide levels between sCJD subtypes might reflect the divergent neuronal targeting and might help to understand better the basis of sCJD phenotypic heterogeneity.

**Funded by**: This research was funded by grants from the Italian Ministry of Health (‘Ricerca corrente’), the German Federal Ministry of Education and Research (projects: FTLDc 01GI1007A), the EU Joint Programme-Neurodegenerative Diseases networks Genfi-Prox (01ED2008A), the Foundation of the State Baden-Württemberg (D.3830), the EU (Moodmarker) program (01EW2008), the German Research Foundation/DFG (SFB1279), the Boehringer Ingelheim Ulm University BioCenter (D.5009) and the Thierry Latran foundation (D.2468).

**Acknowledgement**: The authors wish to thank Stephen Meier, Barbara Polischi, M.Sc. and Benedetta Carlà M.Sc. for their valuable technical assistance.

**BSE pathogenesis in the ileal Peyer’s patches and the central and peripheral nervous system of young cattle 8 months post oral BSE challenge**

Ivett Ackermann^a^, Reiner Ulrich^b^, Kerstin Tauscher^c^, Olanrewaju I. Fatola^a^, Christine Fast^a^, Markus Keller^a^, James C. Shawulu^a,d^, Mark Arnold^e^, Stefanie Czub^f^, Martin H. Groschup^a^, and Anne Balkema-Buschmann^a^

^a^Friedrich-Loeffler-Institut, Institute of Novel and Emerging Infectious Diseases, Greifswald-Insel Riems, Germany; ^b^Institute of Veterinary Pathology, Faculty of Veterinary Medicine, Leipzig University, Germany; ^c^Friedrich-Loeffler-Institut, Department of Experimental Animal Facilities and Biorisk Management, Greifswald-Insel Riems, Germany; ^d^Department of Veterinary Anatomy, University of Abuja, Nigeria; ^e^Animal and Plant Health Agency Sutton Bonington, Sutton Bonington, Loughborough, England; ^f^Canadian Food Inspection Agency, Lethbridge Laboratory, Lethbridge, Alberta, Canada

**Aims**: After oral exposure of cattle with classical bovine spongiform encephalopathy (C-BSE), the infectious agent ascends from the gut to the central nervous system (CNS) primarily via the autonomic nervous as the first entry port to system. However, the early timeline of the progression from the gut to the brain has so far remained widely undetermined. To shed light on the early BSE pathogenesis in unweaned calves, we orally infected calves at six to eight weeks of age with a high dose of classical BSE, and followed the pathogenesis within the first eight months post infection.

**Material and Methods**: 18 unweaned Simmental calves aged 4 to 6 weeks were orally challenged with 100 g each of a classical BSE brainstem pool, while two calves served as negative controls. The animals were euthanized and necropsied at predetermined time points of 1 week as well as 2, 4, 6 and 8 months post infection (mpi). Two infected cattle were kept until the development of clinical symptoms of BSE and served as positive controls. For each of the 18 infected and two negative control calves, samples of the ileal Peyer’s patches as well as the CNS and peripheral nervous system (PNS) were examined by immunohistochemistry (IHC), protein misfolding cyclic amplification (PMCA) and by transgenic Tgbov XV mouse bioassay.

**Results**: In the ileal Peyer’s patches, BSE prions were detectable as early as two mpi by PMCA and transgenic mouse bioassay. From four mpi, PrP^Sc^accumulation was detectable by IHC in tingible body macrophages (TBMs) of the IPP follicles and already in follicular dendritic cells (FDCs). We were also able to show that as early as 8 mpi, the thoracic spinal cord as well as the parasympathetic nodal ganglion of these animals may contain PrP^BSE^and BSE infectivity. The positive control animals developed clinical signs of BSE after incubation periods of 32 mpi and 36 mpi, respectively.

**Conclusions**: Our study demonstrates for the first time PrP^BSE^(by PMCA) and prion infectivity (by mouse bioassay) in the ileal Peyer’s patch (IPP) of young calves as early as 2 months after infection. From 4 mpi nearly all calves showed PrP^BSE^positive IPP follicles by IHC. We could also show that the centripetal prion spread starts early after challenge at least in this age group, which represents an essential piece of information for the risk assessments for food, feed and pharmaceutical products produced from young calves.

**Funded by**: This research was funded by WALA Heilmittel GmbH. The sponsors had no role in the design, execution, interpretation, or writing of the study.

**Acknowledgement**: We thank the Scientific Advisory Group (SAG), namely Thierry Baron (ANSES Lyon), Michael Beekes (RKI Berlin), Jim Hope, Marion Simmons, John Spiropoulos (all APHA Weybridge) and Paul Brown (NINDS Bethesda, USA) for their advice and input regarding the design and interpretation of this study. Julia Neumeister, Daniel Balkema and Bärbel Hammerschmidt are acknowledged for their skillful technical assistance. We are thankful to Lukas Steinke, Nicole Sinkwitz, Kerstin Kerstel and Doreen Fiedler for their excellent care of the bioassay mice. We are grateful to Stefanie Marzahl, Ben Schiller and Volker Netz for the great care and handling of the experimental cattle.

**The combinatorial effect of chronic drug intake and microgravity on Amyloid formation**

Shimon Amselem^a^, Yariv Marmur^b^, Natalia Szenkier^c^, Ami Navon^c^, Atan Gross^c^, Lawrence Steinman^d,e^, Jonathan Rothbard^d,e^, and Marcela Viviana Karpuj^b^

^a^SpacePharma R & D – A Bio-Space Co., Israel, ^b^Department of Industrial Engineering and Management, Ort Braude, Karmiel, Israel, ^c^Department of Biological Regulation, The Weizmann Institute of Science, Israel, ^d^Department of Neurology and Neurological Sciences & Pediatrics, Stanford Univ, Stanford USA, ^e^Department of Biotechnology Engineering, Ort Braude, Karmiel, Israel

Environmental factors such as temperature, and pH affect amyloid formation. Very little is known regarding the impact of gravity on these aggregates. Here, we demonstrate, using an in vitro model, the effect of microgravity on amyloid formation in the presence and absence of newly identified anti-amyloidogenic drugs. This includes drugs that are known to penetrate the BBB as well as Nutraceuticals that could be approved quickly if found to be effective in more advanced models. We identified drugs that reverse existing as well as inhibit the formation of amyloid structures. Unexpectedly we identified two drugs that are known to be chronically taken and induce the formation of these aggregates. Interestingly, the impact of some of these drugs was attenuated under microgravity conditions. Our findings are important since when traveling to space it might be important to utilize this screening method to test the potential harmful effect of the combinatorial effect of microgravity and certain drugs. The opportunity to study drugs and cells at microgravity permits exploration into chemical and biological properties that might not be present at usual conditions due to the masking effects of gravity forces.

**Biobank of genetic CJD in Israel**

Alice Anane, and Victor Novack

CJD Foundation Israel, and Soroka University Medical Center, Israel

**Aims**: A sharing longitudinal biobank of samples and values of genetic families with E200K mutation and patients.

**Material and Methods**: The world prevalence of the disease is 1:500,000–1,000,000 a year. About 85% are sporadic cases, and about 15% are genetic. In Israel, there is the highest prevalence in the world of the genetic form (gCJD), estimated by about 1:200,000 a year, due to a common mutation that induces the risk, at the Libyan-Tunisian community at Israel, which is characterized by many members at each family. The estimation is thousands of families with the genetic risk, concentrate in rather small area of the state of Israel.

My name is Alice Anane, a daughter of a father, who died from this disease at a young age (49). After discovering I and my siblings are carriers, I founded the association in 2008 and I am very active worldwide to advance research for this horrible and devastating disease. We unite a community of the genetic families. Our registry counts more than 400 members (representatives of families) and it grows constantly.

Our partner – Prof. Victor Novack, Clinical Epidemiologist. MD, Ph.D. Head of The Research Authority, Supervising Negev Bio Bank (NBB), a high-quality biorepository of biological samples that operates with MIDGAM, a national Israeli infrastructure for biomedical research and includes five Israeli medical centers, which located in different areas and works as biobanking sites. NBB will be in charge of managing and maintaining the samples of CJD from all the sites in its facilities. We invite researchers to apply and initiate new research on our human samples, sharing values of results with us, for conducting AI of Big Data research.

**Results**: Longitude studies for biomarkers, early diagnosis and therapeuties for gCJD.

**Conclusions**: Conducting and sharing data on the same specific samples for longitude studies can bring a revelation for science and for us at the shortest time in the most efficient way. https://www.youtube.com/watch?v=8UQTpoBYvdU&t=10s

**Comprehensive Characterization of Genetic Creutzfeldt-Jakob Disease Caused by the E200K Mutation in the U.S.**

Brian S. Appleby^a^, Megan Piazza^b,c^, Melissa Keinath^b^, Alberto Bizzi^d^, Curtis Tatsuoka^e^, Thomas W. Prior^b^, Keisi Kotobelli^a^, Wenquan Zou^a^, Mark L. Cohen^a^, and Ignazio Cali^a^

^a^National Prion Disease Pathology Surveillance Center, Case Western Reserve University, Cleveland, OH, USA; ^b^Center for Human Genetics, University Hospitals Cleveland Medical Center, Cleveland, OH, USA; ^c^Prevention Genetics, Marshfield, WI, USA; ^d^Fondazione IRCCS, Istituto Neurologico Carlo Besta, Milan, Italy; ^e^Department of Population and Quantitative Health Sciences, Case Western Reserve University, Cleveland, OH, USA

**Aims**: E200K is the most common cause of genetic Creutzfeldt-Jakob disease (gCJD) worldwide. Most are of the E200K-129 M haplotype and information regarding the clinicopathologic profile of E200K-129 V is limited. The objective of this project was to systematically evaluate all E200K cases submitted to the National Prion Disease Pathology Surveillance Center (NPDPSC).

**Material and Methods**: The NPDPSC database was queried for cases submitted to the NPDPSC from 1997–2018 with a final diagnosis of gCJD due to E200K. Demographic, clinical, diagnostic, and neuropathologic data were collected and analyzed. Cases were divided into four genotypes dependent on the *PRNP* codon 129 polymorphic variations.

**Results**: 181 autopsy-confirmed E200K cases were used for this study. Most cases were of the E200K-129 M haplotype (n = 156, 88%) and 21 (12%) were E200K-129 V. The mean age at death was 61 years (STD: 10.1, range: 37–85) and did not differ significantly between haplotypes. There was a statistically significant difference in mean illness duration between genotypes, with E200K-129 MM having the quickest duration (3.7 ± 3.1 months) and E200K-129 MV having the longest duration (11.1 ± 10 months) (p < 0.001). Codon 129 heterozygotes had a longer mean disease duration (10.4 ± 9.2 months) compared to homozygotes (3.8 ± 3.3 months), regardless of haplotype (p < 0.001). Codon 129 homozygotes were less likely to have a known family history compared to heterozygotes (78.5% vs 93.6%, p = 0.021). Mean age at death varied by presenting symptoms (p = 0.014). E200K-129 M had lower mean cerebrospinal fluid (CSF) tau levels compared to E200K-129 V (4,997 vs 10,221, p = 0.037). Codon 129 homozygotes experienced sleep disorders more commonly than heterozygotes, regardless of haplotype (23% vs 10%, p = 0.038), had more cases with elevated CSF 14-3-3 protein (46% vs 23%, p = 0.003) and periodic sharp wave complexes on electroencephalogram (51% vs 21%, p = 0.033). All cases that underwent CSF real time quaking induced conversion were positive, except for one case in which the sample was slightly bloody. Initial brain magnetic resonance imaging was suggestive of CJD in 50% of subjects. E200K-129 M differed from E200K-129 V with respect to histologic lesion profiles and immunohistochemical features. Five histological subtypes were identified across all cases.

**Conclusions**: We will summarize clinicopathologic characteristics of the largest autopsy confirmed group of E200K cases. E200K haplotypes demonstrated different histologic profiles and disease durations. Homozygosity at codon 129, regardless of haplotype, was associated with several important clinical and diagnostic features. Codon 129 polymorphic changes should be considered when evaluating and studying individuals with the E200K mutation.

**Funded by**: Centers for Disease Control and Prevention and National Institutes of Health

**Grant number**: CDC 1 NU38CK000480 (Appleby) and NIH AG068359 (Cali)

**Acknowledgement**: NPDPSC staff, CJD Foundation, referring physicians, patients, and families

**Heterozygosity at cervid *Prnp* codon 138 progressively blocks prion conversion *in vitro* and partly confines prion propagation to the periphery in knock-in mice**

Maria I. Arifin^a^, Doris Zeng^a^, Samia Hannaoui^a^, Michael Beekes^b^, Gordon Mitchell^c^, Sylvie Benestad^d^, Lech Kacmarczyk^e^, Walker Jackson^e^, and Sabine Gilch^a^

^a^Department of Comparative Biology and Experimental Medicine, University of Calgary, Calgary, Canada; ^b^Prion and Prionoid Research Unit, Centre for Biological Threats and Special Pathogens, Robert Koch Institute, Berlin, Germany; ^c^National and OIE Reference Laboratory for Scrapie and CWD, Canadian Food Inspection Agency, Ottawa, Canada; ^d^Norwegian Veterinary Institute, OIE Reference Laboratory for CWD, Ås, Norway; ^e^Wallenberg Center for Molecular Medicine, Department of Clinical and Experimental Medicine, Linkoping University, Linköping, Sweden

**Aims**: A serine to asparagine amino acid substitution at *Prnp* codon 138, unique to reindeer/caribou and fallow deer, has been associated with reduced susceptibility to chronic wasting disease (CWD). We aim to determine the susceptibility of transgenic ‘knock-in’ mice expressing this 138 N cervid *Prnp* variant to various CWD isolates and to establish the molecular mechanisms behind their partial resistance to the disease.

**Material and Methods**:

1. *In vivo* studies: knock-in mice expressing cervid *Prnp*, instead of mouse *Prnp*, with either serine and/or asparagine at codon 138 (138 S/S [wild-type], S/N or N/N) were intracerebrally or intraperitoneally inoculated with tissue homogenates from CWD-positive white-tailed deer from North America, reindeer from an experimental study, and reindeer, moose, or red deer from Norway. Brain, spleen, spinal cord, and feces were harvested upon euthanasia when the mice reached terminal prion disease. The presence of prions was detected by proteinase-K (PK) digestion followed by Western blotting (WB) and/or real-time quaking-induced conversion (RT-QuIC) assay.
2. *In vitro* studies: we used brain homogenates from naïve 138 S/S, S/N, or N/N knock-in mice as substrates in protein misfolding cyclic amplification (PMCA) assay to determine whether the 138 N *Prnp* allele affects prion conversion *in vitro*. Reactions were seeded with brain homogenates from CWD-positive wild-type reindeer or knock-in mice, then subjected to PK digestion followed by WB to determine the presence of PK-resistant prions (PrP^res^).

**Results**:

- Knock-in mice expressing at least one N allele at codon 138 (138 S/N and 138 N/N) were resistant to clinical CWD, except when inoculated with the moose isolate from Norway. PrP^res^was not detected in the brains and spinal cords of these non-clinical mice. However, seeding activity was detected using RT-QuIC predominantly in the mice spleens, regardless of inoculation route.
- PrP^C^harboring the 138 N allele was less efficiently converted to PrP^res^in PMCA, even upon serial passaging (sPMCA). Interestingly, PrP^C^from heterozygous 138 S/N mice brains but not 138 N/N mice, lost their conversion ability upon sPMCA, with PrP^res^no longer detectable by passage 3.

**Conclusions**: Our results suggests that free-ranging reindeer/caribou carrying the 138 N *Prnp* variant, particularly in the heterozygous state (138 S/N), are more resistant to CWD than their homozygous wild-type or even 138 N/N counterparts. However, if infected with CWD prions, these animals also have the potential to be silent carriers and may shed prions throughout their lifespan, raising concerns for intra- and interspecies transmission.

**Funded by**: Genome Canada, Genome Alberta, Alberta Prion Research Institute, Margaret Gunn Foundation for Animal Research, University of Calgary Seed Grant, Alberta Graduate Excellence Scholarship (International)

**Acknowledgement**: We would like to thank Debbie McKenzie and Trent Bollinger for providing us with the white-tailed deer isolates.

**Loss of prion protein control of glucose metabolism contributes to neurodegeneration: dichloroacetate as a promising medicine to treat Creutzfeldt-Jakob disease**

Hélène Arnould^a,b^, Vincent Baudouin^a,b^, Anne Baudry^a,b^, Luiz W. Ribeiro^a,b^, Mathéa Pietri^a,b^, Hector Ardila-Osorio^a,b^, Carole Crozet^f^, Fatima Djouadi^g,h^, Mireille Laforge^a,b^, Gildas Bertho^c,d^, Odile Kellermann^a,b^, Jean-Marie Launay^i,j^, Gerold Schmitt-Ulms^e^, and Benoit Schneider^a,b^

^a^INSERM, UMR-S 1124, Paris, France; ^b^Université Paris Cité, UMR-S 1124, Paris, France; ^c^CNRS, UMR 8601, Paris, France; ^d^Université Paris Cité, UMR 8601, Paris, France; ^e^University of Toronto, Tanz Centre for Research in Neurodegenerative Diseases, Canada; ^f^IRMB, Université de Montpellier, INSERM, CHU de Montpellier, Montpellier, France; ^g^INSERM, UMR-S 1138, Paris, France; ^h^Université Paris Cité, UMR-S 1138, Paris, France; ^i^Assistance Publique des Hôpitaux de Paris, INSERM UMR942, Hôpital Lariboisière, Paris, France; ^j^Pharma Research Department, Hoffmann La Roche Ltd, Basel, Switzerland

**Aims**: The cellular prion protein (PrP^C^) is well known for its central role in Creutzfeldt-Jakob disease and has also been implicated in the two most prevalent neurodegenerative diseases, Alzheimer’s and Parkinson’s diseases. Yet, despite considerable interest in this protein, the role of PrP^C^is still partially understood and debated. We aimed to identify novel cell functions governed by PrP^C^and investigate whether dysregulation of those functions contributes to prion diseases.

**Material and Methods**: To this purpose, we compared the proteome and metabolome of PrP^C^expressing 1C11 neuronal cells to those of PrP^null^-1C11 cells stably repressed for PrP^C^expression and analyzed the phenotype of primary cultures of hippocampal neurons isolated from PrP^+/+^- and PrP^0/0^-FVB mice. The status of novel cell functions governed by PrP^C^was then probed in prion-infected C57Bl/6 J mice.

**Results**: We show that PrP^C^contributes to the regulation of the energetic metabolism by orienting cells towards mitochondrial oxidative degradation of glucose. Through its coupling to cAMP/protein kinase A signaling, PrP^C^tones down the expression of the pyruvate dehydrogenase kinase 4 (PDK4) and optimizes the activity of the mitochondria pyruvate dehydrogenase complex. Such PrP^C^action favors the transfer of cytosolic pyruvate into mitochondria and its conversion into acetyl-CoA to produce ATP, which thereby limits fatty acid β-oxidation and subsequent onset of oxidative stress conditions. The corruption of PrP^C^metabolic regulatory role by pathogenic prions PrP^Sc^causes in the mouse brain an imbalance between glucose oxidative degradation and fatty acid β-oxidation in a PDK4-dependent manner. The metabolic reprogramming of the prion-infected brain towards fatty acid β-oxidation promotes neurodegeneration. The inhibition of PDK4 with the medicine dichloroacetate (DCA) at a late stage of the disease counteracts PrP^Sc^-induced metabolic derangements, attenuates neurodegeneration in the brain, and extends the survival of prion-infected mice.

**Conclusions**: Our study reveals a new role of PrP^C^in the control of the glucose energetic metabolism whose dysregulation by PrP^Sc^contributes to prion diseases. Our work introduces PDK4 as a potential therapeutic target to combat prion diseases. Our data further suggest DCA, a medicine already approved for the treatment of congenital lactic acidosis and tested in diverse clinical trials for an anti-cancer action, would be used as a symptom-modifying drug for treating Creutzfeldt-Jakob disease patients.

**Funded by**: INSERM, the French Agence Nationale de la Recherche (ANR) and the ARSLA foundation.

**Grant number**: ANR-16-CE16-0021-01, ANR-14-JPCD-0003-01, ARSLA n°J19D08DOC026.

**Acknowledgement**: We thank the Cytometry and Molecular Biochemistry-Biology core facility (Cyto2BM) of BioMedTech Facilities INSERM US36 | CNRS UMS2009 | Université Paris Cité.

**Prion propagation is dependent upon key N-terminal amino acids within the prion protein**

P Arora, MLD Rayner, P Klöhn, C Schmidt, J Collinge and P Jat

MRC Prion Unit at UCL, London W1W 7FF, UK

**Aims**: To identify residues within the unstructured N-terminal domain of the prion protein that are critical for efficient prion propagation. Previous work suggested that most of the N-terminal residues could be disregarded whereas the C-terminal region of the protein mediated formation of infectious prions. However subsequent studies involving reconstitution of *Prnp^0/0^* mice with amino-terminal deletion mutants indicated that the N-terminus was essential for efficient prion propagation.

**Material and Methods**: We have generated derivatives of PK1 and CAD5 cells, PK1-KD and CAD5-KD, stably knocked-down for PrP^C^expression to a level that renders them fully resistant to prion infection, but regain susceptibility to infection upon restoring PrP expression. A library of single, double, or triple alanine replacements within residues 23–111 were prepared and used to stably reconstitute PK1-KD cells. The reconstituted cells were challenged with RML prions in scrapie cell assays (SCA) to identify mutants that affected prion propagation. PrP mutants that identified key residues were stably transduced into CAD5-KD cells and challenged with ME7, 22 L and MRC2 prions to identify residues required for their propagation. Identification of key residues was refined by replacing single amino acids (aa) with alanine, expressing them in CAD5-KD cells and challenging them in a SCA.

**Results and Conclusions**: Alanine replacement of aa within 105–111, in Charge Cluster 2, were required for propagation of RML, ME7, 22 L and MRC2 prions. Replacement of other residues exhibited strain-specific effects or had no effect. Replacements in Charge Cluster 2 (90–111) including aa 105–111 dominantly inhibited prion propagation in the presence of endogenous wild type PrP^C^whilst other changes were not inhibitory. None of the alanine mutants blocked prion propagation when expressed in chronically prion-infected cells. Analysis of single alanine replacements within aa 105–111 indicated that efficient prion propagation was dependent on leucine 108 and valine 111 acting alone or lysine 105, threonine 106 and asparagine 107 acting together at the infection stage. This is highly interesting in light of the 2.7 Å cryo-EM structure of *ex vivo* RML prion fibrils indicating that K105, T106, N107 are part of the positively charged patch on the surface of proto filaments whereas L108 and V111 are hydrophobic and face inwards forming hydrophobic interactions with P136 and I138 for stability of the prion fibrils.

**Funded by**: MRC Prion Unit core funding.

**Grant number**: PR-R17- 0916- 23,004

**Understanding the Conformational Dynamics of Infectious Prion Fibrils**

Efrosini Artikis and Byron W. Caughey

Laboratory of Persistent Viral Diseases, Rocky Mountain Laboratories, National Institute of Allergy and Infectious Diseases, National Institutes of Health, Hamilton, MT USA

**Aims**: The conformational conversion of endogenous cellular prion protein (PrP^C^) into misfolded protein assemblies (PrP^Sc^) is a signature of prion disease. The mechanism by which PrP^C^adopts the misfolded PrP^Sc^template and undergoes structural conversion remains unknown. We aim to elucidate aspects of the conversion process by first understanding the structural elements of infectious PrP^Sc^fibrils (263 K, aRML). The identification of structural ‘hot spots’ on the fibrillar surface will provide insight into the recruitment and binding of PrP^C^monomers. Furthermore, the systematic comparison of the key conformational regions among different strains will illuminate the requirements for conversion fidelity. Secondly, investigating the presence of lipids and neutralizing cofactors will clarify why and how these factors help push the PrP^C^away from its native fold.

**Material and Methods**: A variety of molecular dynamics (MD) techniques are employed to probe conformational changes, cofactor and lipid interactions, and strain variations.

**Results**: High resolution cryo-EM structures have demonstrated that infectious PrP^Sc^fibrils contain common structural elements. Our MD simulations have identified regions of conformational plasticity within conserved structural motifs which may aid in the initial interaction and recruitment with PrP^C.^Although the fibrillar surface contains highly charged and hydrophobic patches, segments of these potential binding sites are occluded by the accumulation of divalent cations and water molecules. Pores and pockets within the fibril may play important roles in the propagation mechanism. Furthermore, *in silico* mutagenesis of single amino acid residues that are important in conversion implicates nuanced structural changes that can be allosteric and long-range. Specifically, we have identified hydrogen bonding networks and persistent aromatic interactions as vital aspects of structural integrity. Lastly, accelerated MD allows for simulations in the microsecond time regime where we can observe interactions between monomeric PrP^C^and a GPI-anchored 263 K fibril inserted in a neuronal lipid bilayer.

**Conclusions**: Understanding the structural underpinnings of prion conversion allows for insight into the molecular pathogenesis and strain diversity of prion diseases. Such knowledge will ultimately aid in the rational design of conversion inhibitors.

**Funded by**: Division of Intramural Research, National Institute of Allergy and Infectious Diseases

**Grant number**: ZIA AI000580

**Pathomorhological analysis and atomic force microscopy examination of infectious prion protein, isolated from the brain with Creutzfeldt-Jakob disease**

Andrei N. Astashonok^a^, Sergei A. Guzov^b^, Тatyana V. Dokukina^c^, Michael V. Mackhrov^c^, and Nikolai N. Poleshchuk^a^

^a^The Republican Research and Practical Center for Epidemiology and Microbiology, Minsk, Belarus; ^b^Belarusian State Medical University, Minsk, Belarus; ^c^Republican Research and Practice Mental Health Center, Minsk, Belarus

**Aims**: To perform neuropathological analysis in case of Creutzfeldt-Jakob disease and characterize the nanostructural organization of misfolded prion protein by atomic force microscopy

**Material and Methods**: We report here a neuropathological examination of case of Creutzfeldt-Jakob disease from Belarus. Neuropathological analysis was performed by standard protocols. For an in-depth description of pathological features, *fragments* of the human *brain, containing* cortex, thalamus, hippocampus, cerebellum, globus pallidus, etc., were analyzed by electron microscopy (JEM-1011, Japan). Using tapping mode atomic force microscopy (Nanoscope 3D Multimode) the purified brain fractions, containing protease-resistant prion protein, were investigated.

**Results**: The neuropathological findings were characterized by spongiform changes in gray matter, extensive loss of neurons, synaptic abnormalities, astocytic and microglial proliferation. Multiple vacuoles in different size, containing the fragments of structurally modified membranes, in cerebral cortex were found. Using the purified brain fractions, treated with proteinase K, we found the presence of prion rods (100–200 nm) and globules (10–25 nm). Atomic force microscopy analysis also revealed differences in height of prion rods, which were characterized by inhomogeneous relief areas. At the same time, the central part (core), as a rule, did not have sharp changes in roughness compared to the peripheral areas.

**Conclusions**: The obtained data, in our opinion, are important for understanding the role of conformational rearrangements in infectology, and may also be useful in the creation of recombinant prions. In addition, we hope that this study will help clarify strain differences in CJD and possibly other prion disorders.

**Funded by**: This work was supported by State Applied Research Program (registration number 20,210,323, Belarus)

**Acknowledgement**: We are grateful the clinical specialists, who performed the instrumental diagnostics (MRI), EEG and some laboratory analysis.

**Different α-Synuclein Prion Strains Cause Dementia with Lewy Bodies and Multiple System Atrophy**

Jacob I. Ayers^a,b^, Joanne Lee^a^, Octovia Monteiro^a^, Amanda L. Woerman^a,b^, Ann A. Lazar^c^, Carlo Condello^a,b^, Nick A. Paras^a,b^, and Stanley B. Prusiner^a^

^a^Institute for Neurodegenerative Diseases, Weill Institute for Neurosciences, University of California San Francisco, San Francisco, CA; ^b^Department of Neurology, Weill Institute for Neurosciences, University of California San Francisco, San Francisco, CA; ^c^Division of Biostatistics, University of California San Francisco, San Francisco, CA; ^d^Department of Biochemistry and Biophysics, University of California San Francisco, San Francisco, CA

**Aims**: The aim of this study was to characterize **α**-synuclein prion activity from the brains of multiple system atrophy (MSA), dementia with Lewy bodies (DLB), and Parkinson’s disease (PD) patients.

**Materials and Methods**: We first employed detergent extraction and limited proteolysis followed by sodium phosphotungstic acid (PTA) precipitation to increase the concentration of the putative PD and DLB **α**-synuclein prions. We then tested their infectivity in several HEK293 T cell lines overexpressing wild-type (WT) **α**-synuclein or one of three familial PD–causing mutations (A53T, A30P, and E46K).

**Results**: The modified protocol to digest and purify **α**-synuclein prions from patient brain samples resulted in the detection of **α**-synuclein prion activity from DLB samples. Low **α**-synuclein prion levels in PD samples prevented reliable characterization. Contrary to the finding that MSA prions were unable to infect the E46K-expressing cell line, DLB **α**-synuclein prions were capable of replicating in all four of the **α**-synuclein cell models tested.

**Conclusions**: Our findings argue that the MSA strain of **α**-synuclein prions differs from those that accumulate in PD/DLB. Manipulating dominant negative inhibition of **α**-synuclein prion replication has created a new approach to identifying novel prions and deciphering the features of prion multiplication.

**Funded by:/Grant number**: This work was supported by a grant from the National Institutes of Health (AG002132) (S.B.P.), as well as by support from the Sherman Fairchild and Sergey Brin Foundations (S.B.P.).

**Acknowledgement**: We acknowledge the very special expertise of Dr. Jill Ostrem in helping us with the complex nosology of the synucleinopathies. We thank Roy Vaz for assisting with computational modeling. We also thank Shoko Honzumi and Jisoo Lee for optimization of our HTRF and cell assays. Synucleinopathy and control tissue samples were supplied by the neuropathology core of the Massachusetts Alzheimer’s Disease Research Center (AG005134); the Parkinson’s UK Brain Bank at Imperial College London, funded by Parkinson’s UK, a charity registered in England and Wales (948,776) and in Scotland (SC037554); and the Banner Sun Health Research Institute Brain and Body Donation Program of Sun City, AZ, funded by the National Institute of Neurological Disorders and Stroke (U24 NS072026 National Brain and Tissue Resource for Parkinson’s Disease and Related Disorders), the National Institute on Aging (P30 AG19610 Arizona Alzheimer’s Disease Core Center), the Arizona Department of Health Services (contract 211,002, Arizona Alzheimer’s Research Center), the Arizona Biomedical Research Commission (contracts 4001, 0011, 05–901, and 1001 to the Arizona Parkinson’s Disease Consortium), and the Michael J. Fox Foundation for Parkinson’s Research.

**Inside the kuru-plaque variant (MV2K) of sporadic Creutzfeldt-Jakob disease: a detailed clinical and histo-molecular appraisal**

Simone Baiardi^a,b^, Angela Mammana^b^, Marcello Rossi^b^, Sofia Dellavalle^b^, Giorgio Giaccone^c^, Anna Ladogana^d^, Sabina Capellari^b,e^, and Piero Parchi^a,b^

^a^Department of Experimental, Diagnostic and Specialty Medicine (DIMES), University of Bologna, Bologna, Italy; ^b^IRCCS, Istituto delle Scienze Neurologiche di Bologna, Bologna, Italy; ^c^Fondazione I.R.C.S.S. Istituto Neurologico Carlo Besta, Milano, Italy; ^d^Department of Neuroscience, Istituto Superiore di Sanità, Rome, Italy; ^e^Department of Biomedical and Neuromotor Sciences (DIBINEM), University of Bologna, Bologna, Italy

**Aims**: To investigate in-depth the clinicopathological heterogeneity characterizing the kuru-plaque variant (MV2K subtype) of sporadic Creutzfeldt-Jakob disease (sCJD).

**Material and Methods**: We evaluated neurological histories, results of cerebrospinal fluid (CSF) biomarker studies (RT-QuIC, 14-3-3 protein, total-tau, NfL), brain diffusion-weighted resonance imaging (DW-MRI), and electroencephalographic recordings (EEG) in 126 patients with a definite (n = 87) or probable (n = 39) diagnosis of sCJDMV2K. In the definite cases, the histo-molecular assessment included PrP^Sc^typing by western blot, standard histologic staining, and PrP immunohistochemistry in several brain areas. We also investigated the prevalence and topographic extent of mixed histotypes (i.e., MV2K+MM2C) and the number of cerebellar kuru plaques and their effect on clinical phenotype.

**Results**: The mean disease duration was 18.0 ± 11.8 months. Duration correlated positively with the severity of pathologic change and the number of cerebellar kuru plaques (rho = 0.397, p = 0.002). At the clinical onset and in the early stages patients manifested prominent, often mixed, cerebellar symptoms and cognitive complaints (mainly memory loss), variably associated with behavioral/psychiatric, and sleep disturbances. Gait instability was the most frequent isolated presentation (19.8%). Full-blown dementia was rare in the early stage. No significant clinical differences were detected by comparing pure and mixed histotypes (MV2K+MM2C). CSF prion RT-QuIC was positive in 74/76 (97.3%) cases, while 14-3-3 protein and total-tau (cut-off >1250 pg/ml) in 52.6% and 75.9% respectively. NfL was increased in all tested cases (mean 8447.6 ± 4772.6 pg/ml). Brain DW-MRI showed hyperintensity of striatum, cerebral cortex, and thalamus in 81.4%, 49.3%, and 33.8% of cases, and a profile typical for CJD in 71 out 77 (92.2%). An abnormal cortical signal was most frequently detected in mixed MV2K+MM2C than pure MV2K (64.7% vs. 16.7%, p = 0.007). EEG revealed periodic sharp-wave complexes in only 8.7% of cases.

**Conclusions**: sCJDMV2K is a relatively common subtype showing some ‘atypical’ features. Consequently, often patients do not fulfill diagnostic criteria in the early disease stages. Atypical features include the long disease duration, the relative slow worsening of cognitive decline, and the poor sensitivity of some diagnostic tests such as CSF 14-3-3 protein detection and EEG. Moreover, this subtype uniquely accumulates PrP in the form of cerebellar amyloid kuru plaque. Clinicians should consider sCJDMV2K in any patient presenting (or early developing) mixed cognitive and cerebellar dysfunctions. CSF prion RT-QuIC and brain DW-MRI represent the most sensitive diagnostic tests. These data strongly suggest that, despite some atypical features, sCJDMV2K can be clinically diagnosed accurately based on clinical data, DW-MRI, CSF prion RT-QuIC assay, and codon 129 genotyping.

**Incidences Trends of Creutzfeldt-Jakob Disease in Israel**

Yacov Balash^a^, Meir Walker^b^, Esther Kahana^c,d^, Hadeel Nabal^e^, Emilia Anis^e^, Hanna Rosenmann^f^, Ron Milo^c,d^, and Amos D. Korczyn^b^

^a^Department of Neurology, Kaplan Medical Center, Rehovot, Israel; ^b^Sackler Faculty of Medicine, Tel-Aviv University, Tel-Aviv, Israel; ^c^Department of Neurology, Barzilai University Medical Center, Ashkelon, Israel; ^d^Faculty of Health Sciences, Ben Gurion University of the Negev, Beer-Sheva, Israel; ^e^Division of Epidemiology, Ministry of Health, Jerusalem, Israel; ^f^Department of Neurology, the Agnes Ginges Center for Human Neurogenetics, Hadassah Hebrew University Medical Center, Jerusalem, Israel

**Aims**: Sporadic Creutzfeldt-Jakob disease (s-CJD) is a rare, fatal neurodegenerative disorder. Familial cases of Creutzfeldt-Jakob disease (f-CJD) due to mutations in the *PRNP* gene are even rarer around the world; however, in Israel there is an unusual focus of f-CJD patients carrying the E200K mutation. The number of E200K mutation carriers in Israel is increasing, which raised the suspicion of CJD transmission from person to person. If such transmission does occur, the incidence of s-CJD is expected to increase.

**Materials and Methods**: Using data from the national CJD registry and official statistics on the Israeli population, we studied incidence rates of f-CJD and s-CJD for the period from 1985 to 2018 applying the SEER (Surveillance Epidemiology and End Results) statistical packet elaborated in the US National Cancer Institute

**Results**: In total, we identified 621 CJD patients (405 f-CJD and 216 s-CJD) cases. In the cohort of f-CJD patients the mean age-adjusted annual incidence rate over the above-mentioned period was 1.88 ± 0.09 (95% CI: 1.7–2.08) per 1,000,000. In the cohort of s-CJD patents the mean age-adjusted incidence rate over the same period was 0.93 ± 0.06 (95% CI: 0.81–1.06) per 1,000,000 people. No significant time trends were found according to the permutation test of joinpoint regression in either of them.

When both cohorts were combined, the mean annual age-adjusted incidence of CJD in Israel was 2.81 ± 0.11 (95% CI: 2.58–3.04) per 1,000,000. From 1985 to 2018, there was a borderline increase in incidence of 0.8% per year, (95% CI: 0–1.6, p = 0.37), which is non significant.

**Conclusions**: Israel has a great predominance of f-CJD compared to s-CJD. The mean incidence of s-CJD in Israel is similar to most countries. Between 1985 and 2018, the annual age adjusted incidence rates for both forms of CJD have remained stable. There is no evidence for transmission of the disease.

**Glycans are not necessary to maintain the pathobiological features of Bovine Spongiform Encephalopathy**

Tomás Barrio^a,^*, Alicia Otero^b,^*, Hasier Eraña^c,d,e^, Jorge M. Charco^c,d,e^, Marina Betancor^b^, Carlos M. Díaz-Domínguez^c^, Olivier Andréoletti^a^, Juan María Torres^f^, Qingzhong Kong^g^, Juan José Badiola^b^, Rosa Bolea^b^, and Joaquín Castilla^c,e,h^

^a^UMR INRAE-ENVT 1225 Interactions Hôtes-Agents Pathogènes (IHAP), École Nationale Vétérinaire de Toulouse, Toulouse, France; ^b^Centro de Encefalopatías y Enfermedades Transmisibles Emergentes, Facultad de Veterinaria, Instituto Agroalimentario de Aragón – IA2 (Universidad de Zaragoza-CITA), Zaragoza, Spain; ^c^Center for Cooperative Research in Biosciences (CIC bioGUNE), Basque Research and Technology Alliance (BRTA), Bizkaia Technology Park, Derio, Spain; ^d^ATLAS Molecular Pharma, S.L., Derio, Spain; ^e^Centro de Investigación Biomédica en Red de Enfermedades infecciosas (CIBERINFEC), Carlos III National Health Institute, Madrid, Spain; ^f^Centro de Investigación en Sanidad Animal (CISA-INIA), Madrid, Spain; ^g^Departments of Pathology and Neurology & National Center for Regenerative Medicine, Case Western Reserve University, Cleveland, OH, USA; ^h^IKERBASQUE, Basque Foundation for Science, Bilbao, Spain

*These authors contributed equally to this work.

**Aim**: The role of the glycosylation status of PrP^C^in the conversion to its pathological counterpart and on cross-species transmission of prion strains has been widely discussed. Here, we assessed the effect of glycosylation on the strain characteristics of BSE isolates with different transmission histories upon propagation on a model expressing a non-glycosylated human PrP^C^.

**Material and methods**: Bovine, ovine and porcine-passaged BSE, and vCJD isolates were used as seeds/inocula in both *in vitro* and *in vivo* propagation assays using a non-glycosylated human PrP^C^-expressing mouse model (TgNN6h).

**Results:** On PMCA, all isolates maintained the biochemical characteristics of BSE. On bioassay, all PMCA-propagated BSE prions were readily transmitted to TgNN6h mice, in agreement with our previous *in vitro* results. TgNN6h mice reproduced the characteristic neuropathological and biochemical hallmarks of BSE, suggesting that the absence of glycans did not alter the pathobiological features of BSE prions. Moreover, back-passage of TgNN6h-adapted BSE prions to transgenic mice expressing bovine PrP^C^(BoTg110) mice recovered the full BSE phenotype.

**Conclusions**: Our results support the notion that the glycosylation of human PrP^C^is not essential for the preservation of the human transmission barrier for BSE prions or for the maintenance of BSE strain properties.

**Funded by**: MINECO Ministerio de Economía y Competitividad (Spanish Government)

**Grant number**: AGL2015-65,560-R

**Funded by**: Ministerio de Ciencia, Innovación y Universidades (Spanish Government)

**Grant number**: RTI2018-098711-B-I00, RTI2018-098515-B-I00

**Funded by**: European Region Development Fund (ERDF)

**Grant number**: POCTEFA EFA148/16

**Acknowledgement**: The authors want to acknowledge the excellent technical assistance of Patricia Piñeiro, Sandra Felices and Daniel Romanos. We also thank MINECO for the Severo Ochoa Excellence Accreditation (SEV-2016-0644). We thank the Biobank from Fundación Hospital Alcorcón de Madrid (Madrid – Spain) and Basque Biobank – Fundación Vasca de Innovación de Investigación Sanitaria (Bizkaia – Spain) for providing the human samples.

**Translational profiling of neuronal subtypes in pre-symptomatic fatal familial insomnia mice reveals TOR signaling in somatostatin-expressing neurons**

Susanne Bauer^a^, Lars Dittrich^b^, Lech Kaczmarczyk^a,b^, Melvin Schleif^b^, Rui Benfeitas^c^, and Walker S. Jackson^a,b^

^a^Wallenberg Center for Molecular Medicine, Department of Biomedical and Clinical Sciences, Linköping University, Linköping, Sweden; ^b^German Center for Neurodegenerative Diseases (DZNE), Bonn, Germany; ^c^Department of Biochemistry and Biophysics, Science for Life Laboratory, National Bioinformatics Infrastructure Sweden (NBIS), Stockholm University, Sweden

**Aims**: Analysis of cell type-specific gene expression changes in pre-symptomatic stages of genetic prion diseases fatal familial insomnia (FFI) and Creutzfeldt-Jakob disease (CJD).

**Material and Methods**: To determine how distinct neurons respond to different mutations in the prion protein gene at pre-symptomatic stages, we used RiboTag to isolate cell type-specific, translating mRNA from GABAergic, glutamatergic, somatostatin- (SST) and parvalbumin- (PV) expressing neurons of 9-month-old knock-in mouse models of FFI and CJD. Differential gene expression analysis followed by gene set enrichment analysis (GSEA) was performed for all cell types in both diseases. We further constructed an undirected weighted gene co-expression network for SST neurons to identify functional models and hub genes.

**Results**: SST^+^neurons showed the most prominent gene expression changes in both diseases, especially in FFI, with high similarities between the two diseases. GSEA demonstrated similar enrichment patterns of functional terms for GABAergic cell types in both FFI and CJD, whereas responses in glutamatergic neurons were disease specific. For SST^+^neurons, functional analysis revealed upregulation of ribosomal biogenesis, mitochondrial function and neurodegenerative disease pathways, and downregulation of synaptic function and small GTPase mediated signaling in FFI. Analysis of an SST co-expression network revealed a disease-associated module, functionally associated with autophagy and TORC1 signaling. Of the identified module hub genes, three were further significantly differentially expressed in FFI SST neurons, including *Depdc5*, a component of mTOR regulator complex GATOR1, and Nucleosome Remodeling Deacetylase complex component *Mta3*. Importantly, the molecular changes reported here were very different from those reported for an acquired prion disease model.

**Conclusions**: This study identifies SST neurons as an early affected cell type, showing similar responses in both FFI and CJD with downregulation of mTOR signaling as a potential explanatory mechanism underlying many of the observed changes. The observation that FFI and CJD have such similar cellular and molecular signatures indicates that a common therapy may be effective for multiple inherited prion diseases, but possibly less effective for acquired prion disease.

**Funded by**: This work was supported by the Knut and Alice Wallenberg foundation and the German Center for Neurodegenerative Diseases (DZNE).

**Correlation between bioassay and PMCA for human prion decontamination studies**

Maxime Bélondrade^a^, Christelle Jas-Duval^a,b^, Simon Nicot^a^, Lilian Bruyère-Ostells^a^, Charly Mayran^a^, Laetitia Herzog^b^, Fabienne Reine^b^, Juan Maria Torres^c^, Chantal Fournier-Wirth^a^, Vincent Béringue^b^, Sylvain Lehmann^d^, and Daisy Bougard^a^

^a^Pathogenesis and control of chronic and emerging infections, Université de Montpellier, Etablissement Français du Sang, Inserm, Université des Antilles, Montpellier, France; ^b^VIM INRA, Université Paris-Saclay, Jouy-en-Josas, France; ^c^Centro de Investigación en Sanidad Animal, Instituto Nacional de Investigación y Tecnología Agraria y Alimentaria (CISA-INIA), Madrid, Spain; ^d^CHRU de Montpellier and Université de Montpellier, IRMB, INSERM U1183, Laboratoire de Biochimie Protéomique Clinique, Montpellier, France

**Aims**: To date, about 500 iatrogenic Creutzfeldt-Jakob disease cases have been reported worldwide. The unusual resistance of prions to decontamination processes, its large tissue distribution, and the uncertainty about the prevalence of variant Creutzfeldt-Jakob disease (vCJD) in the general population, raise the possibility that some surgical procedures may be at risk of iatrogenic CJD transmission in healthcare facilities. It is therefore vital that decontamination procedures applied to medical devices before their reprocessing are thoroughly validated. We previously described an *in vitro* assay, based on PMCA technology associated to steel wires as a surface model, called Surf-PMCA. This method allowed us to classify prion decontamination treatments according to their effectiveness on vCJD prions and highlight the lack of efficacy of several marketed reagents to inactivate human vCJD prions. The objective of the present study is to confirm previous results obtained *in vitro* by *in vivo* transmission studies in transgenic mice susceptible to vCJD prions.

**Material and Methods**: We used here a transgenic mouse line permissive to variant CJD prions (tgBov) to study the correlation between a residual seeding activity measured *in vitro* by Surf-PMCA and residual *in vivo* infectivity. Stainless steel wires, used as carrier models of prions for inactivation studies by mimicking surfaces of surgical instruments, were contaminated by contact with vCJD infected brain homogenate. The presence of residual prion infectivity after several decontamination methods was evaluated by inoculated processed wires in the brain of tgBov mice.

**Results**: The use of NaOH 1 N, NaClO 0.2 or 2% and 134°C steam sterilization led to 100% survival of the animals confirming their full efficacy on vCJD prions. Regarding the partially effective treatments, 1/10 mouse died in the NaOH 0.1 N group and 2/8 mice died in the group inoculated with steel wires sterilized at 121°C. Results obtained with 6 marketed reagents were well-correlated to previous ones obtained by Surf-PMCA and show that 4 of them had a low efficacy to remove vCJD prions.

**Conclusions**: In this study, we demonstrate a good correlation between our previous *in vitro* results with infectivity studies in transgenic mice susceptible to vCJD prions. These experiments emphasize the strength of the Surf-PMCA method as a rapid and sensitive assay for the evaluation of prion decontamination procedures and also confirm the lack of efficacy of several marketed reagents on vCJD prions decontamination.

**Large-scale PMCA screening of retropharyngeal lymph nodes and in white-tailed deer and comparisons with ELISA and IHC: the Texas CWD study**

Rebeca Benavente^a^, Paulina Soto^a^, Mitch Lockwood^b^, and Rodrigo Morales^a^

^a^Department of Neurology, McGovern Medical School, University of Texas Health Science Center at Houston, Texas, USA; ^b^Texas Park and Wildlife Department, Texas, USA

Chronic wasting disease (CWD) is a transmissible spongiform encephalopathy that affects various species of cervids, and both free-ranging and captive animals. Until now, CWD has been detected in 3 continents: North America, Europe, and Asia. CWD prevalence in some states may reach 30% of total animals. In Texas, the first case of CWD was reported in a free-range mule deer in Hudspeth and now it has been detected in additional 14 counties.

Currently, the gold standard techniques used for CWD screening and detection are ELISA and immunohistochemistry (IHC) of obex and retropharyngeal lymph nodes (RPLN). Unfortunately, these methods are known for having a low diagnostic sensitivity. Hence, many CWD-infected animals at pre-symptomatic stages may be misdiagnosed. Two promising *in vitro* prion amplification techniques, including the real-time quaking-induced conversion (RT-QuIC) and the protein misfolding cyclic amplification (PMCA) have been used to diagnose CWD and other prion diseases in several tissues and bodily fluids. Considering the low cost and speed of RT-QuIC, two recent studies have communicated the potential of this technique to diagnose CWD prions in RPLN samples. Unfortunately, the data presented in these articles suggest that identification of CWD positive samples is comparable to the currently used ELISA and IHC protocols. Similar studies using the PMCA technique have not been reported.

**Aims**: Compare the CWD diagnostic potential of PMCA with ELISA and IHC in RPLN samples from captive and free-range white-tailed deer.

**Material and Methods**: In this study we analyzed 1,003 RPLN from both free-ranging and captive white-tailed deer collected in Texas. Samples were interrogated with the PMCA technique for their content of CWD prions. PMCA data was compared with the results obtained through currently approved techniques.

**Results**: Our results show a 15-fold increase in CWD detection in free-range deer compared with ELISA. Our results unveil the presence of prion infected animals in Texas counties with no previous history of CWD. In the case of captive deer, we detected a 16% more CWD positive animals when compared with IHC. Interestingly, some of these positive samples displayed differences in their electroforetic mobilities, suggesting the presence of different prion strains within the State of Texas.

**Conclusions**: PMCA sensitivity is significantly higher than the current gold standards techniques IHC and ELISA and would be a good tool for rapid CWD screening.

**Funded by: USDA**

**Grant number**: AP20VSSPRS00C143

**A miRNA fingerprint in Plasma-derived extracellular vesicles of hSOD1G93A transgenic swine**

V Benedetti^a^, E Berrone^a^, M Garofalo^b,c^, C Tessarolo^a^, S Gagliardi^b^, L Messa^b,d^, S Carelli^d,e^, V Carta^a^, G Cagnotti^f^, C Testori^a^, M Gallo^a^, C Galli^g^, A Perota^g^, R Duchi^g^, L Bergamaschi^g^, C Cereda^b^, C Casalone^a^, and C Corona^a^

^a^SC Neuroscienze, Istituto Zooprofilattico Sperimentale del Piemonte Liguria e Valle d’Aosta; Turin, Italy; ^b^Genomic and post-Genomic Unit, IRCCS Mondino Foundation, Pavia, Italy; ^c^Department of Biology and Biotechnology ‘L. Spallanzani’, University of Pavia, Pavia, Italy; ^d^Department of Biomedical and Clinical Sciences ‘L. Sacco’, University of Milan, Milan, Italy; ^e^Pediatric Clinical Research Center Fondazione ‘Romeo ed Enrica Invernizzi’, University of Milan, Milan, Italy; ^f^Department of Veterinary Science, University of Turin, Grugliasco (TO), Italy; ^g^ Avantea, Cremona, Italy

**Aims**: Two goals of Amyotrophic Lateral Sclerosis (ALS) research are: i) validation of new experimental models ii) identification of diagnostic biomarkers, in order to speed up the diagnosis, to monitor its progression and to assess whether a new therapy may be effective. Extracellular vesicles (EVs) and their content may be reliable clinical biomarkers for ALS, as they have yet been used for the diagnosis and prognosis of various diseases. In this context, we developed a hSOD1G93A transgenic swine characterized by a reproducible preclinical and clinical phase in order to clarify certain ALS etiopathogenetic aspects. In particular, EVs characterization in this animal model could elucidate their role in relation to key elements of the pathological process. Therefore, this study aimed at evaluating of miRNA into EVs isolated from hSOD1G93A transgenic swine plasma, in preclinical and clinical phase.

**Material and Methods**: EVs were isolated from plasma of hSOD1G93A and control pigs by a modified precipitation method. EVs were characterized by Nanosight, flow cytometry and Western blotting. miRNAs were sequenced on a NextSeq 500/550.

**Results**: Phenotype characterization confirmed that the majority of EVs were exosomes expressing the typical exosome markers (CD63, TSG101, Flotillin 1, Alix). As regard miRNA analysis, some miRNAs were deregulated in transgenic swine, associated to a specific expression pattern in the model. Furthermore, an evident up-regulation of miR-206 was observed. This miRNA is involved in the proper formation and regeneration of the mature neuromuscular junction. Interestingly, its increased exosomal expression was also detected comparing hSOD1G93A samples at clinical phase with their corresponding preclinical samples. Nonetheless, miR-206 was up-regulated in homozygous transgenic swine compared to heterozygous ones.

**Conclusions**: In conclusion, these data show that in the swine model, biomarkers already associated to ALS are expressed and that miRNAs may be used as monitoring tools for disease severity.

**Funded by**: Supported by *Compagnia di San Paolo grant n.2016.0097, Regione Lombardia* and *Italian Ministry of Health*

**Grant number:** ‘INnovazione, nuovi modelli TEcnologici e Reti per curare la SLA’ – ID 1157625

**Acknowledgement**: The project has been co-funded by the 2014–2020 ROP ERDF resources

**A multiparametric imaging-based cellular assay sensitive to the toxicity of prion-infected brain tissue demonstrates that purified highly infectious scrapie prions are not directly neurotoxic**

Iryna V. Benilova, Madeleine Reilly, Aline T. Marinho, Emmanuel Risse, Isabella Sheikh, Parmjit S. Jat, and John Collinge

MRC Prion Unit at University College London, Institute of Prion Diseases, London, UK

**Aims**: Prions are misfolded multichain assemblies of cellular prion protein (PrP^C^). Prion diseases are associated with accumulation of a broad range of PrP assemblies, a minority of which meet the biochemical criteria of classical infectious prions PrP^Sc^, resistant to proteinase K digestion. Infectious prions can accumulate to a high titre without signs of neurodegeneration, a process known as subclinical infection. Studies of prion pathogenesis in mice showed that prion infectivity and neurotoxicity follow distinct pathways and can be uncoupled, suggesting that the onset of neuropathology is driven by a toxic species distinct from the infectious species. To test this hypothesis, we aimed at developing an assay that could discriminate between toxicity of prion-infected and uninfected brain tissue.

**Material and Methods**: We developed a multiparametric, imaging-based cellular assay sensitive to the toxicity of crude brain homogenates (BH) prepared from scrapie-sick CD1 mice inoculated with the Rocky Mountain Laboratory (RML) strain of scrapie. Uninfected CD1 BH was used as a control. RML BH was also used for purification of highly infectious prion rods according to an established protocol (Wenborn et al, Scientific Reports 2015).

Toxicity assay consisted of time course studies of neurite retraction on IncuCyteS3 imaging system followed by an end-point analysis of other morphological features such as dendritic spine density and neurite fragmentation using Opera Phenix platform (Benilova, Reilly et al, PNAS 2020; Reilly et al., Scientific Reports, in press).

**Results**: Prion-infected BH caused a multitude of dose-dependent changes of neuronal phenotype that were quantified in a high-throughput automatic fashion. Purified RML prions at titres exceeding the titre of RML BH eliciting maximal neurodegeneration were not acutely neurotoxic, whether dissolved in cell culture medium or brain homogenate from PrP-expressing or null mice. As a prerequisite for undertaking fractionation of the toxic species from RML BH, we also searched for a detergent compatible with toxicity. Pretreatment of RML BH with sarkosyl abolished toxicity without diminishing the infectious titre. Moreover, we showed that ICSM18, a mouse monoclonal antibody against amino acid residues 143–153 of murine PrP, known to cure infected cells and delay the onset of prion disease in infected mice, lacked inherent neurotoxicity and blocked toxicity of RML BH.

**Conclusions**: The lack of detectable direct toxicity of highly infectious prions or sarkosyl-treated infectious BH is consistent with models of prion neurotoxicity being mediated by toxic species distinct from infectious prion assemblies.

**Funded by**: The High-Content Biology Platform at the MRC-UCL Laboratory for Molecular Cell Biology University Unit is supported by the MRC Dementia Platform UK (MR/M02492X/1) and MRC core funding (MC_U12266B).

**Efficient propagation and strain diversity of prions from pure synthetic origin**

V. Béringue^a^, L. Herzog^a^, F. Reine^a^, N. Aron^b^, J. Torrent^a^, A. Igel^a^, M. Moudjou^a^, P. Sibille^a^, O. Andréoletti^b^, D. Martin^a^, and H. Rezaei^a^

^a^Université Paris-Saclay, INRAE, UVSQ, VIM, Jouy-en-Josas, France; ^b^INRAE, ENV Toulouse, IHAP, Toulouse, France

**Aims**: The pathogenic activity of bacterially-derived recombinant PrP (recPrP) refolded *in vitro* into amyloid fibrillar assemblies has been key to the demonstration of prion proteinaceous nature. While a global consensus on the *bona fide* infectivity of recPrP fibrils exists, minimalistic amyloid preparations most often trigger incomplete attack rates, with long incubation periods or asymptomatic disease on primary transmission to susceptible hosts. This was interpretated as i) limited conversion capacity of recPrP fibrils due to conformational mismatch, necessitating serial transmission for conformational switch and adaptation (‘deformed templating’), and/or ii) low proportion of infectious conformers in the fibrils preparation, and/or iii) and/or necessity of co-factor(s) assisting the conversion or conferring infectivity.

**Material and Methods**: We generated co-factor free preparations of recPrP assemblies from three different species (hamster, human and mouse) and inoculated them by intracerebral route to transgenic mice expressing hamster PrP. In parallel, we characterized their ultra-structure and morphology by atomic force microscopy (AFM).

**Results**: The three preparations of recPrP assemblies induced a clinical disease with a 100% attack rate in hamster PrP transgenic mice. Serial passaging allowed identification of divergent synthetic prion strains. Their selection/propagation appears to be determined, as for natural prions, by the potential presence of a species barrier. Remarkably, these synthetic strains were formed from short PrP^Sc^fragments from ~140 to 230 amino acid residues. Depending on the strain type, these short fragments were found alone or co-propagated with ‘classical’ PrP^Sc^forms. AFM analyses of the three recPrP assemblies preparations with a 10 Å nominal cantilever revealed an overall fibrillar shape. However, numerous spherical objects were observed, staked on the fibrils as protrusions or at the vicinity of the fibers or as isolated objects.

**Conclusions**: The obtention of these ‘mini-prions’ stands in striking contrast to many previous studies pointing to region ~90-140 as key to prion infectivity and has important implications for the ongoing debate on prion structure(s). The ultrastructure of the recPrP fibers is heterogeneous and remarkably differs from homogeneous fibrillar structures observed so far. Collectively, these studies offer unprecedented opportunities for further structural investigation of the PrP domains conferring infectivity and strain properties.

**Formation and localization of disease-associated PrP aggregates in primary neuronal and glial culture systems**

Antonio Berretta, Juan M. Ribes, Hazim Halim, George Thirlway, John Collinge, and Peter C. Kloehn

MRC Prion Unit, UCL, London, UK

**Aims**: Disease-associated prion protein (PrP^d^) is a misfolded, aggregated, protease-resistant and infectious conformer of the cellular prion protein (PrP^c^). Although accumulation of PrP^d^is the main hallmark of prion diseases, cellular mechanisms that facilitate PrP conversion are not well understood. The aim of this project is to develop primary neuronal and glial culture systems to investigate the role of these cells in the propagation of prion strains.

**Material and Methods**: Pure, mixed and co-cultures of neurons and glial cells were prepared from E17 and postnatal mice. Cultures were infected with brain homogenates from RML, Me7 and 22 L diseased animals or with extracellular vesicles isolated from infected cells (PrP^d^-EV). By using 1) prion-infected primary cultures from PrP-knockout animals; 2) treatments with brain homogenates from healthy animals; 3) pre-treatment with neutralising anti-PrP antibodies were used as negative control to prevent infection. An adapted scrapie cell assay was developed to monitor the formation of protease-resistant PrP aggregates. Immunolabelling of cultures with anti-PrP antibodies and confocal analysis were used to visualise fibril-like aggregates. Subsequently, antibodies against synaptic vesicles and markers to label lipid rafts and detergent-resistant membranes were used to identify the subcellular localisation of PrP^d^.

**Results**: In hippocampal neuronal cultures, treatments for at least 2–3 weeks with RML and 22 L brain homogenates and with PrP^d^-EV resulted in prion infection. Prion infection was corroborated using both an adapted scrapie cell assay and immunofluorescence showing the presence of Proteinase K resistant PrP^d^and over several micrometers of fibril-like aggregates, respectively. PrP^d^fibrils were attached to neuronal dendrites with no apparent colocalization with glutamatergic synaptic proteins. PrP^d^infection was also observed in astrocytes in mixed cultures with neurons and in pure cultures only if treated with cAMP, which mimics *in vivo* astrocytes after neuronal activity.

**Conclusions**: We established complementary primary culture systems for visualizing PrP^d^aggregates in neurons and glial cells. Further ongoing experiments aim to identify the subcellular localization of PrP^d^aggregates focusing on synapses and lipid microdomains.

**Funded by**: UK Biotechnology and Biological Sciences Research Council (BBSRC, BB/V001310/1) and UK Medical Research Council (MRC, MC_UU_00024/4)

**The Amyloid Aggregation Study on board The International Space Station**

E. Berrone^a^, F. Cardone^b^, C. Corona^a^, M. Sbriccoli^b^, V. Benedetti^a^, A. Favole^a^, C. Palmitessa^a^,F. Porreca^b^, A. Cornacchia^b^, S. Camerini^b^, M. Casella^b^, M. Crescenzi^b^, S. Sirigu^c^, A. Crisafi^c^, C. Piacenza^d^, G. Truscelli^d^, D. Castagnolo^e^, C. Pacelli^f^, M. Crisconio^f^, G. Valentini^f^, G. Mascetti^f^, S. P.^f^, S. Sennato^g^, F.A. Scaramuzzo^h^, G. Meli^i^, E. Fiori^i^, A. Manca^i^, and C. Casalone^a^

^a^Istituto Zooprofilattico Sperimentale del Piemonte, Liguria e Valle d’Aosta, Turin, Italy; ^b^Istituto Superiore di Sanità, Rome, Italy; ^c^Altec S.p.A., Turin, Italy; ^d^Argotec, Turin, Italy; ^e^Telespazio, Naples, Italy; ^f^Italian Space Agency (ASI), Rome, Italy; ^g^ISC – CNR, Rome, Italy; ^h^‘Sapienza’ University of Rome, Rome, Italy; ^i^European Brain Research Institute, Rome, Italy

**Aims**: Pathological aggregates made of amyloid β (Aβ) peptides are one of the main hallmarks of the Alzheimer disease (AD). The study of the effects of longlasting space stays on the development of AD and other proteinopathies in astronauts is an urgent need in view of the programs for interplanetary travels announced by national and international space agencies. ‘Amyloid Aggregation’ is an Italian Space Agency (ASI) simple test tube aiming to assess if and how Aβ peptides aggregation is affected by microgravity, identifying a possible professional risk in astronauts spending long periods in space.

**Material and Methods**: The experiment was performed on the International Space Station (ISS) during the ‘BEYOND’ mission. Solubilized Aβ peptides were encapsulated in the cap of special jars, also containing the reaction fluid in a separate lower compartment. Once on the ISS, Aβ peptides were mixed with the reaction fluid and left to aggregate at ambient temperature for various times. At the end of each interval, samples were frozen and then returned to Earth. The experiment was repeated on Earth recapitulating the same ISS conditions except for the absence of weight.

**Results**: We have already completed all the parts regarding the preparation of peptide aggregates in orbit and on Earth. Four major analytical techniques (Western Blotting, Mass Spectrometry, Atomic Force Microscopy, Dynamic Light Scattering) have been optimized which are expected to provide insight into the structure and kinetic of aggregate formation. Even if sample analysis has been started only a few months ago, interesting preliminary results are summing up comparing the ISS samples with those processed on Earth.

**Conclusions**: The possibility to perform experiments on board of the ISS represents a unique opportunity to study if and how ISS microgravity influences Aβ aggregates formation. Hopefully, results from this project will help to design more stringent scientific studies for a better understanding of the possible risk of developing protein aggregation diseases in astronauts and eventually to identify specific tools to protect human health from neurodegenerative proteinopathies during long-lasting space missions.

**Funded by**: Italian Space Agency (ASI)

**Grant number**: ASI n. 2018-4-r.0

**Preclinincal biomarkers in scrapie: assessment of neurogranin (Ng) and neurofilament light chain (NfL)**

Marina Betancor^a^, Sonia Pérez-Lázaro^a^, Alicia Otero^a^, Belén Marín^a^, Inmaculada Martín-Burriel^a,b,c^, Kaj Blennow^d,e^, Juan José Badiola^a^, Henrik Zetterberg^d,e,f,g,h^, and Rosa Bolea^a^

^a^Centro de Encefalopatías y Enfermedades Transmisibles Emergentes, Universidad de Zaragoza, IA2, IIS Aragon, Zaragoza, Spain; ^b^Laboratory of Biochemical Genetics (LAGENBIO), Faculty of Veterinary, Institute for Health Research Aragon (IIS Aragón), AgriFood Institute of Aragon (IA2), University of Zaragoza, Miguel Servet 177, 50013 Zaragoza, Spain; ^c^Centro de Investigación Biomédica en Red de Enfermedades Neurodegenerativas (CIBERNED), Instituto Carlos III.; ^d^Department of Psychiatry and Neurochemistry, Institute of Neuroscience & Physiology, the Sahlgrenska Academy at the University of Gothenburg, Mölndal, Sweden; ^e^Clinical Neurochemistry Laboratory, Sahlgrenska University Hospital, Mölndal, Sweden; ^f^Department of Neurodegenerative Disease, UCL Institute of Neurology, Queen Square, London, UK; ^g^UK Dementia Research Institute at UCL, London, UK; ^h^Hong Kong Center for Neurodegenerative Diseases, Clear Water Bay, Hong Kong, China

**Aims**: Prion diseases are usually diagnosed in the symptomatic stage when the neuronal damage is spread throughout the central nervous system (CNS). The assessment of biological molecules that allow to detect asymptomatic cases is needed, and in this context, scrapie, where pre-symptomatic infected animals can be detected through rectal biopsy, becomes a good model to evaluate biopathological markers of prion diseases in early stages. Neurogranin (Ng) and neurofilament light chain (NfL) are proteins that reflect synaptic and axonal damage, respectively, and have been studied as cerebrospinal fluid (CSF) biomarkers in different neurodegenerative disorders. In this study, we evaluate Ng and NfL expression both at the protein and transcript level in the CNS of preclinical and clinical scrapie-affected sheep compared with healthy control sheep. We also assessed the level of these proteins in ovine CSF. The possible correlation between these proteins and the main neuropathological events in prion diseases, PrP^Sc^deposition and spongiosis, is also assessed.

**Material and Methods**: Twenty-one sheep from three groups (scrapie clinical, scrapie preclinical and uninfected) were used. The assessment of Ng and NfL protein expression was performed in nine CNS areas by immunohistochemistry followed by evaluation with the Image J software. PrP^Sc^deposition was assessed by immunohistochemistry and spongiosis by hematoxylin-eosin, both followed by semiquantitative evaluation. The gene expression of *NRGN* and *NEFL* was carried out in four of these same areas by real-time PCR. The concentration of NfL and Ng in CSF was measured by internal ELISA based on monoclonal antibodies NfL21 and NfL23 and monoclonal antibodies Ng2 and Ng36.

**Results**: Results showed a decrease in Ng and NfL at the protein and gene expression levels in several brain regions as the disease progresses, and significant changes between its levels in control and preclinical animals. On the contrary, CSF levels of NfL increased throughout the progression of the disease. Negative correlations between neuropathological markers of prion disease and the concentration of the studied proteins were also found.

**Conclusions**: Although further research is needed, the results suggest that Ng and NfL could act as biomarkers for neurodegeneration onset and intensity in preclinical cases of scrapie, and therefore this could be the base for future research evaluating its diagnostic use.

**Funded by:/ Grant number**: RTI2018-098711-B-I00, IU/2023/2017, POCTEFA EFA148/16

**Folding Intermediates of the Cellular Prion Protein Across Disease and Therapy**

Emiliano Biasini

Department CIBIO, University of Trento

**Aims**: Recent computational advancements in the simulation of biochemical processes allow investigating the mechanisms involved in protein regulation with realistic physics- based models, at an atomistic level of resolution. Using these techniques to study the physiological regulation of different model proteins, we discovered a key functional role played by non-native metastable states appearing along the folding pathways. This unexpected observation led us to design a completely novel drug discovery paradigm, named Pharmacological Protein Inactivation by Folding Intermediate Targeting (PPI-FIT), based on the rationale of negatively regulating protein expression by targeting folding intermediates (1). In this work, PPI-FIT was tested for the first time on the cellular prion protein (PrP).

**Material and Methods**: We combined computational technologies and a wide range of in vitro and cell-based experimental techniques to identify and characterize the pharmacological properties of small ligands for a folding intermediate of PrP.

**Results**: We predicted the all-atom structure of an intermediate appearing along the folding pathway of PrP, and identified four different small molecule ligands for this conformer, all

capable of selectively lowering the expression of the protein by promoting its lysosomal degradation directly from the Endoplasmic Reticulum.

**Conclusions**: Our data support the notion that the level of target proteins like PrP could be modulated by acting on their folding pathways, implying a previously unappreciated role for folding intermediates in the biological regulation of protein expression.

**References**: 1. Spagnolli et al. Commun Biol 2021

**Funded by**: Fondazione Telethon, Italy Grant number: GGP20043 **Funded by**: CJD Foundation, USA Grant number: -

**Granagard as an anti-aging and neuroprotective agent in animals and humans suffering from neurological diseases**

Orli Binyamin^a,b^, Panayiota Petrou^c^, Kati Frid^a,b^, Ariel Ginzberg^c^, Guy Keller ^a,b^, Ann Saada^d^, Dimitrios Karussis^c^, Ruth Gabizon^a^

^a^Department of Neurology, The Agnes Ginges Center for Human Neurogenetics, Hadassah Medical Center, Jerusalem, Israel^b^Faculty of Medicine, The Hebrew University of Jerusalem, Jerusalem, Israel.^c^Multiple sclerosis Center and cell therapies Unit, Unit and Laboratory of Neuroimmunology and The Agnes-Ginges Center for Neurogenetics, Hadassah University Hospital, Jerusalem.^d^Department of Genetic and Metabolic Diseases, Hadassah Medical Center, Jerusalem Israel, Jerusalem, Israel; Faculty of Medicine, The Hebrew University of Jerusalem, Jerusalem, Israel.

**Aims:** We have shown that Granagard, a nanoformulation of pomegranate seed oil, can delay disease advance in several models of brain diseases by neuroprotective pathways. Since aging is the main risk factor for the manifestation of neurodegenerative diseases, we compared the anti-aging and anti-prion activity of Granagard to that of Metformin, an anti-diabetic drug shown to extend longevity. To this effect, both compounds were administrated to TgMHu2ME199K mice, a model of gCJD. In parallel, we tested the safety and clinical effect of Granagard on the cognitive function of multiple sclerosis (MS) patients. Cognitive impairment is a common feature appearing in different studies at 43-70% of MS patients. None of the novel MS treatments can protect against cognitive disability in MS.

**Material and Methods:** Groups of TgMHu2ME199K mice and wt controls were treated for 2 weeks or 4 months with either Metformin or Granagard and followed for their appropriate neurologic score. Subsequently, organs including brains were collected for biochemical and pathologic evaluation of aging and prion disease hallmarks. These included levels of phosphorylated AMPK, NRF2, GFAP, stem cell markers, disease related PrP and neurological scores. In the human study, Granagard was administrated to MS patients in addition to their individual immunomodulatory MS-treatments. Group-A was given GranaGard for the first three months and then placebo pills containing soybean oil for additional three months, while group B was given first Placebo and then Granagard. All patients were subjected to extensive cognitive testing at the diffe rent time points.

**Results:** We demonstrate that administration of both Granagard and Metformin to TgMHu2ME199K mice increased the activation of anti-aging hallmarks activities such as AMPK, the main energy sensor of cells as well as Nrf2, Hif and COX IV-1, regulators of oxidation and mitochondrial activity. Both compounds also reduced inflammation and increased stem cells production, however did not decrease PrP^Sc^ accumulation. In contrast to Granagard, Metformin had no effect on clinical prion disease features. In the human study, there was a significant beneficial effect of GranaGard to patient's cognitive features, including 12% improvement in the results of verbal abilities. No side effects were reported after 6-12 months administration.

**Conclusions:** While Granagard compares to Metformin in its anti–aging properties, it also presents unique beneficial clinical properties. A first pilot controlled human trial provide indications that long term GranaGard administration to humans is safe and may improve/stabilize cognitive disability in MS patients. Trials in other diseases are in progress.

**Funded by:** Granalix Biotechnologies and The Prusiner-Abramski Research Awards

**Identifying promising therapeutics drugs entering the brain for genetic prion diseases in *C. elegans***

Nicolas Bizat^a,b*^, Valeria Parrales^a^†, Sofian Laoues^a^†, Sébastien Normant^a^, Etienne Levavasseur^a^, Julian Roussel^a^, Nicolas Privat^a^, Alexianne Gougerot^a^, Philippe Ravassard^a^, Patrice Beaudry^a^, Jean-Philippe Brandel^a,c,^Jean-Louis Laplanche^b,d^, and Stéphane Haïk^a,c*^

^a^Paris Brain Institute, Inserm U 1127, CNRS UMR 7225, Sorbonne University, Hospital Pitié-Salpêtrière Paris, France; ^b^Faculté de Pharmacie de Paris, Paris Cité, 4 avenue de l’Observatoire, Paris France; ^c^AP-HP, Cellule Nationale de Référence des Maladies de Creutzfeldt-Jakob, University Hospital Pitié-Salpêtrière, Paris France; ^d^Inserm, UMR-S 1144, Paris France

**Aims**: Human prion diseases are fatal neurodegenerative disorders that include sporadic, infectious and genetic forms. Inherited Creutzfeldt-Jakob disease due to the E200K mutation of the prion protein-coding gene is the most common form of genetic prion diseases. The phenotype resembles that of sporadic Creutzfeldt-Jakob disease at both the clinical and pathological levels, with median disease duration of four months. To date, there is no available treatment for delaying the occurrence or slowing the progression of human prion diseases. Existing *in vivo* models do not allow high-throughput approaches that may facilitate the discovery of compounds targeting pathological assemblies of human prion protein or their effects on neuronal survival.

**Material and Methods**: We generated a genetic model in the nematode *C. elegans*, which is devoid of any homolog of the prion protein, by expressing human prion protein with the E200K mutation in the mechanosensitive neuronal system.

**Results**: Expression of E200K prion protein induced specific behavioural pattern and neurodegeneration of GFP-expressing mechanosensitive neurons, in addition to the formation of intraneuronal inclusions associated with the accumulation of a protease-resistant form of the prion protein. We demonstrated that this experimental system is a powerful tool to study the efficacy of anti-prion compounds on both prion-induced neurodegeneration and prion protein misfolding, moreover in a human PrP context. Within a library of 320 compounds approved for human use and crossing the blood-brain-barrier, we identified five molecules that were active against the aggregation of E200K prion protein and the neurodegeneration it induced in transgenic animals.

**Conclusions**: This model breaks a technological limitation in prion therapeutic research and provides a key tool to study the deleterious effect of misfolded prion protein in a well-described neuronal system and genetic organism model.

**Funded by**: This work was supported by the *CJD foundation* and *Santé Publique France.*

**Acknowledgement**: We thank the *Caenorhabditis Genetics Center* for provided strains. We acknowledge the technical service from the *Brain Institute* platforms in particular: Delphine Boutelier and Yannick Marie (*IGenSeq*); David Akbar and Patrick Michel (*Celis*); Dominique Langui, Aymeric Millecamps, Claire Lovo and Basile Gurchenkov (*icm.Quant*). We acknowledge Vincent Galy for technical help.

**Cross-disease implication of the PrP^C^-PDK1-TACE pathway in amyloid-based neurodegenerative diseases**

Chloé Bizingre^a,b^, Vincent Baudouin^a,b^, Anne Baudry^a,b^, Laura M.A. Camassa^c^, Luiz W. Ribeiro^a,b^, Mathéa Pietri^a,b^, Aurélie Alleaume-Butaux^a,b^, Pierre Nioche^a,b^, Hector Ardila-Osorio^a,b^, Vidar Gundersen^c^, Odile Kellermann^a,b^, Jean-Marie Launay^d,e^, and Benoit Schneider^a,b^

^a^Inserm UMR-S 1124, Paris, France; ^b^Université Paris Cité, UMR-S 1124, Paris, France; ^c^Centre for Molecular Medicine Norway, University of Oslo, Norway; ^d^Inserm UMR942, Hôpital Lariboisière, Paris, France; ^e^Hoffmann-La Roche-Ltd, Basel, Switzerland

**Aims**: Although amyloid-based neurodegenerative diseases such as Alzheimer’s (AD), prion (PrD), Parkinson’s (PD) diseases, and Amyotrophic Lateral Sclerosis (ALS) display distinct etiologies and clinical manifestations, it is suspected that these diseases share common pathocascades. We provided prime evidence for the occurrence of common neurodegenerative pathways in PrD and AD by showing that the scrapie prion protein (PrP^Sc^) in PrD and Aβ peptides in AD both provoke neurodegeneration through dysregulation of the neuroprotective PDK1 kinase – TACE **α**-secretase signaling pathway upon the interaction of PrP^Sc^and Aβ with normal cellular prion protein PrP^C^. We aimed to assess whether another unrelated β-sheet enriched amyloid protein, *i.e*., SOD1^G93A^mutant of ALS, also exerts its neurotoxicity through dysregulation of the PrP^C^-PDK1-TACE pathway.

**Material and Methods**: To this purpose, we conducted experiments with (i) neuronal cell lines (1C11 bioaminergic neuronal cells, NSC34 motor neuron-like cells) and primary neuronal cultures that were exposed to human SOD1^G93A^(hSO1^G93A^) or endogenously expressed hSOD1^G93A^, and (ii) a mouse model with ALS-like pathology (Tg-SOD1^G93A^).

**Results**: We show that PrP^C^is a neuronal receptor for hSOD1^G93A^. The interaction between PrP^C^and this amyloid corrupts the PrP^C^-PDK1-TACE pathway leading to TACE neutralization by internalization. Internalized TACE loses its cleavage activity towards one of its main substrates, TNF**α** receptors (TNFR), whose accumulation at the neuron cell surface renders ALS neurons hypersensitive to TNF**α** inflammatory stress, a strong component of ALS pathogenesis. The silencing of PrP^C^or the pharmacological inhibition of PDK1 relocate TACE back to the plasma membrane where it recovers its neuroprotective cleavage activity towards TNFR, protecting ALS neurons from neuroinflammation. In Tg-SOD1^G93A^ ALS mice, the intrathecal injection of a siRNA against PrP mRNA or the infusion of the PDK1 inhibitor by the intraperitoneal route rescue TNFR shedding, which protects spinal cord motor neurons from neurodegeneration, increases motor performance, and extends the survival time of these mice.

**Conclusions**: This work supports the view that (i) PrP^C^is a global neuronal sensor of amyloids, (ii) dysregulation of the PrP^C^-PDK1-TACE pathway is a trait of neurodegeneration common to several unrelated amyloid-based neurodegenerative diseases, and (iii) posits PDK1 as a potential therapeutic target with broad spectrum to combat these diseases.

**Funded by**: INSERM, the French Agence Nationale de la Recherche (ANR), the European Joint Program on Neurodegenerative Diseases (JPND), and the ARSLA Foundation.

**Grant number**: ANR-14-JPCD-0003–01; ANR-16-CE16–0021–01; ARSLA n°J19D08DOC026.

**Acknowledgement**: Imaging experiments and mRNA/protein studies were performed at the SCM and Cyto2BM core facilities, respectively, of BioMedTech Facilities INSERM US36/CNRS UMS2009/Université Paris Cité.

**Mechanisms of adaptation of synthetic prions in hamsters**

Alyssa J. Block^a^, Ronald A. Shikiya^a^, Thomas E. Eckland^a^, Anthony E. Kincaid^b^, Ryan W. Walters^c^, Jiyan Ma^d^, and Jason C. Bartz^a^

^a^Department of Medical Microbiology and Immunology, Creighton University, Omaha, United States; ^b^Department of Pharmacy Science, Creighton University, Omaha, US; ^c^Department of Medicine, Creighton University, Omaha, US; ^d^Center for Neurodegenerative Science, Van Andel Institute, Grand Rapids, US

**Aims**: Prion diseases are a group of neurodegenerative disorders that affect humans and other mammals. Prions are comprised of PrP^Sc^, the self-templating disease specific conformation of the cellular prion protein, PrP^C^. Synthetic prions are generated *in vitro* from minimal components and cause *bona fide* prion disease in animals. It is unknown, however, if synthetic prions can cross the species barrier *in vivo* following interspecies transmission or if synthetic strains recapitulate brain-derived strains.

**Material and Methods**: To investigate transmission, either murine wildtype (WT) or hamster WT/mutant synthetic prions were inoculated into male Syrian hamsters. Hamsters were monitored for onset of clinical signs including weight gain. Serial intraspecies transmission of murine WT and hamster (WT, D54 mutant) synthetic prions was performed. PrP^Sc^from hamsters inoculated with synthetic prions was biochemically characterized by evaluating electrophoretic mobility and glycoform ratio by Western blot, conformational stability using the denaturant guanidine hydrochloride (Gdn-HCl), and biological activity using protein misfolding cyclic amplification (PMCA). To analyze neuropathology, brain sections were stained with hematoxylin and eosin to assess spongiform degeneration and underwent immunohistochemistry to assess PrP deposition.

**Results**: All hamsters inoculated with murine synthetic prions (MSP) developed prion disease at first passage, exhibiting a high uniformity in onset and clinical signs of disease. Serial intraspecies transmission resulted in a rapid adaptation to hamsters. Throughout adaptation in hamsters, the biochemical characteristics of PrP^Sc^from MSP-infected hamsters remained constant. Interestingly, the strain that emerged shares a striking resemblance to brain-derived strain 139 H, a hamster-adapted form of the murine strain 139A, with a similar transmission history, incubation period, clinical manifestation, pathology and biochemical and biological features of PrP^Sc^. In contrast, all hamsters inoculated with hamster synthetic prions (HSP) did not develop clinical signs of prion disease. Western blot analysis of brain homogenate from HSP^WT^- and HSP^D54^-infected hamsters identified protease-resistant PrP^Sc^, suggesting subclinical infection. Serial intraspecies transmission resulted in clinical disease at second passage and a divergence of disease phenotype between the wildtype and the D54 mutant by third serial passage.

**Conclusions**: Murine synthetic prions efficiently cross the species barrier and rapidly adapt to hamsters resulting in the emergence of a single strain. The emergence of a strain with similar characteristics to 139 H suggest the MSPs are comprised of bona fide PrP^Sc^with 139A-like strain properties. The decreased transmission efficiency observed with the HSPs suggests the HSPs are not authentic PrP^Sc^and instead replicate through the conversion model termed deformed templating.

**Funded by**: National Institutes of Health National Institute of Neurological Disorders and Stroke and National Institute of Allergy and Infectious Disease

**Grant number**: R01NS103763 (NINDS) and 2P01 AI077774 (NIAID)

**Acknowledgements**: We would like to thank the Creighton University Animal Research Facility for excellent animal care.

**Quantitative Detection of α-Synuclein and Tau Oligomers and other Aggregates by Digital Single Particle Counting**

Lara Blömeke^a,c^, Marlene Pils^b,c^, Victoria Kraemer-Schulien^a^, Alexandra Dybala^b,c^, Anja Schaffrath^a^, Andreas Kulawik^a,b,c^, Fabian Rehn^a^, Anne Cousin^a^, Volker Nischwitz^d^, Johannes Willbold^a^, Rebecca Zack^e^, Thomas F. Tropea^e,f^, Tuyen Bujnicki^a^, Gültekin Tamgüney^a,b^, Daniel Weintraub^f,g,h^, David Irwin^e,f^, Murray Grossman^e,f^, David A. Wolk^e^, John Q. Trojanowski^f,i^, Oliver Bannach^a,b,c^, Alice Chen-Plotkin^e,f^, and Dieter Willbold^a,b^

^a^Institute of Biological Information Processing (Structural Biochemistry: IBI-7), Forschungszentrum Jülich, Jülich Germany; ^b^Institut für Physikalische Biologie, Heinrich-Heine-Universität Düsseldorf, Düsseldorf Germany; ^c^attyloid GmbH, Düsseldorf Germany; ^d^Central Institute for Engineering, Electronics and Analytics, Analytics (ZEA-3), Forschungszentrum Jülich, Jülich Germany; ^e^Department of Neurology, Perelman School of Medicine at the University of Pennsylvania, Department of Neurology, Philadelphia, PA, USA; ^f^Center for Neurodegenerative Disease Research, Perelman School of Medicine, University of Pennsylvania, Philadelphia, PA, USA; ^g^Department of Psychiatry, Perelman School of Medicine, University of Pennsylvania, Philadelphia, PA, USA; ^h^Parkinson’s Disease and Mental Illness Research, Education, and Clinical Centers, Philadelphia Veterans Affairs Medical Center, Philadelphia, PA, USA; ^i^Department of Pathology and Laboratory Medicine, Perelman School of Medicine, University of Pennsylvania, Philadelphia, PA, USA

**Aims**: The pathological hallmark of neurodegenerative diseases is the formation of toxic oligomers by proteins such as alpha-synuclein (aSyn) or microtubule-associated protein tau (Tau). Consequently, such oligomers are promising biomarker candidates for diagnostics as well as drug development. In this work, we measured aSyn and Tau aggregate concentrations of 237 cerebrospinal fluid (CSF) samples from five cohorts: Parkinson’s disease (PD), dementia with Lewy bodies (DLB), Alzheimer’s disease (AD), progressive supranuclear palsy (PSP), and a neurologically-normal control group.

**Material and Methods**: We previously developed surface-based fluorescence intensity distribution analysis (sFIDA) featuring single-particle sensitivity and absolute specificity for aggregates. sFIDA is an ELISA-like technique using the same capture and detection antibody but features single-particle sensitivity through a microscopy-based readout.

**Results**: aSyn aggregate concentration discriminates PD and DLB patients from normal controls (sensitivity 73 %, specificity 65 %, area under the receiver operating curve (AUC) 0.68). Tau aggregates were significantly elevated in PSP patients compared to all other groups (sensitivity 87 %, specificity 70 %, AUC 0.76). Further, we found a tight correlation between aSyn and Tau aggregate titers among all patient cohorts (Pearson coefficient of correlation r = 0.81).

**Conclusions**: Our results demonstrate that aSyn and Tau aggregate concentrations measured by sFIDA differentiate neurodegenerative disease diagnostic groups. Moreover, sFIDA-based Tau aggregate measurements might be particularly useful in distinguishing PSP from other parkinsonisms. Finally, our findings suggest that sFIDA can improve pre-clinical and clinical studies by identifying those individuals that will most likely respond to compounds designed to eliminate specific oligomers or to prevent their formation.

**Funded by**: The Michael J. Fox Foundation for Parkinson’s Research

**Grant number**: 14977, 009889

**Strain determinant minimal substructure revealed by dissociation of PrPSc assemblies**

Jan Bohl^a,b^, Angélique Igel-Egalon^a^, Mohammed Moudjou^a^, Laetitia Herzog^a^, Fabienne Reine^a^, Frederic Halgand^b^, Guillaume Van der Rest^b^, Vincent Béringue^a^, and Human Rezaei^a^

^a^VIM, INRAe, Université Paris-Saclay, Jouy-en-Josas, France; ^b^ICP, CNRS, Université Paris Sud, Orsay, France

**Aims**: While the current prion paradigm considers the prion replication as a simple amyloid Ends-growth process, how the strain information is encoded in prion assemblies, their diversity and their dynamic are fare to be clear. In the prion literature, there are multiple examples supporting the propagation of different strains on one given PrP primary structure, indicating therefore the existence of multiple strain -structural-determinants. How and in which PrP protein domain such diversity is structurally encoded, remain entirely open questions.

**Material and Methods**: Extractive prion assemblies from seven prion strains were solubilized in soft detergent conditions to dissociate them into their simplest infectious elementary building block. Their quaternary structure was then studied by size exclusion chromatography. Their infectivity and strain properties were studied by bioassay and PMCA experiments.

**Results**: Our studies demonstrated that PrPSc assemblies from seven different prion strains can be dissociated into a small oligomeric conformer. Biochemical characterization of this oligomeric object by size exclusion chromatography, static light scattering and covalent crosslinking indicated a dimeric PrPres object. Bioassays experiments performed on relevant transgenic mice demonstrated that these purifiable dimeric objects were fully infectious and harbored the strain structural determinant.

Studying the disassembly dynamic at different ionic strengths or through dilution experiments highlighted the existence of an exchange between the condensed state of PrP^Sc^assemblies and the dimeric objects. The characterization of the hydrodynamic radius of the dimeric objects resulting from the dissociation of seven prion strains from three different species revealed a commune elementary building bloc quaternary structure, highlighting thus a strain-invariant oligomerization domain.

**Conclusions**: The existence of such dimeric object harbouring the infectivity and strain structural determinant leads us to conclude that the strain information is not defined by the size of PrP^Sc^elementary brick but by the conformation of this dimer or at least the conformation of a specific domain of this dimer. By considering the number of prion strains identified until know by bioassay in reporter animals, it is not trivial to imagine how such diversity could be encoded in a common quaternary structure in a stable manner during the replication process and without affecting the stability of the dimerization interface. We overcome this thermodynamical paradox by considering the dimerization interface independent in term of folding from the domain encoding the strain information.

**Expression and characterization of the human full-length prion protein in *Leishmania tarentolae***

Najoua Bolakhrif^a,b^, Lothar Gremer^a,b^, and Dieter Willbold^a,b^

^a^Institut für Physikalische Biologie, Heinrich-Heine-Universität Düsseldorf, Germany; ^b^Institute of Biological Information Processing, Structural Biochemistry (IBI-7) Forschungszentrum Juelich, Germany

**Aims**: Transmissible spongiform encephalopathies (TSEs) are also known as prion diseases, as they are based on the conversion of the non-infectious cellular prion protein (PrP^C^) into the infectious isoform, called PrP scrapie (PrP^Sc^). Since prion diseases belong to a group of fatal neurodegenerative diseases and the conversion is yet not fully understood, it is crucial to get deeper insights into the structure and characterization of the membrane bound full-length human PrP expressed by an organism enabling similar posttranslational modifications to human (e.g. glycosylation pattern).

The aim of this project is therefore to express and characterize the full-length human prion protein, including GPI-membrane anchoring and mammalian-like glycosylation.

**Material and Methods**: The following methods were used: cloning, recombinant protein expression, gel electrophoresis, western blotting, bright-field and fluorescence microscopy.

**Results and Conclusion**: Diagnostic PCR proves the genomic integration of the plasmid. The human full-length prion protein was successfully expressed in *Leishmania tarentolae*, indicated by western blotting. Fluorescence microscopy demonstrate the attachment of the prion protein on the outside of the cell membrane. These results will be used to further analyze and characterize the mammalian-like glycosylated PrP^C^and its conversion into PrP^Sc^.

**Funded by**: MobilitätsFonds der Zentrale Gleichstellungsbeauftragte der Heinrich-Heine- Universität im Rahmen der Koordination im Professorinnenprogramm III and Dieter Willbold (Institut für Physikalische Biologie)

**Improved detection of pathological α-synuclein in olfactory mucosa of patients with Parkinson’s disease**

Matilde Bongianni^a^, Mauro Catalan^b^, Daniela Perra^a^, Elena Fontana^a^, Francesco Janes^c^, Claudio Bertolotti^b^, Luca Sacchetto^d^, Stefano Capaldi^e^, Matteo Tagliapietra^a^, Paola Polverino^b^, Valentina Tommasini^b^, Giulia Bellavita^b^, Elham Ataie Kachoie^e^, Roberto Baruca^f^, Andrea Bernardini^c^, Mariarosaria Valente^c^, Michele Fiorini^a^, Erika Bronzato^a^, Stefano Tamburin^a^, Laura Bertolasi^a^, Lorenzo Brozzetti^a^, Maria Paola Cecchini^a^, Gianluigi Gigli^c^, Salvatore Monaco^a^, Paolo Manganotti^b^, Gianluigi Zanus^a^

^a^Department of Neurosciences, Biomedicine, and Movement Sciences, University of Verona, Policlinico G. B. Rossi, Verona, Italy;^b^ Neurology Unit, Department of Medicine, Surgery and Health Sciences, University of Trieste, Ospedale Cattinara, Trieste, Italy;^c^ Neurology Unit, University of Udine Academic Hospital, Udine, Italy;^d^Department of Surgical Sciences, Dentistry, Gynecology and Pediatrics, University of Verona, Verona, Italy;^e^ Biocrystallography Laboratory, Department of Biotechnology, University of Verona, Italy;^f^ Otolaryngology Unit, Department of Medicine, Surgery and Health Sciences, University of Trieste, Ospedale Cattinara, Trieste, Italy

**Aims**: To investigate whether nasal swabbing (NS) performed through OM areas with a different concentration of olfactory neurons, such as *agger nasi* (AN) and middle turbinate (MT), might improve pathological α-syn detection.

**Material and Methods**: OM samples were collected from 95 patients including 66 with PD and 29 with other neurodegenerative disorders in two rounds, between September 2018 and April 2021, at three neurology units. NS were performed by local otolaryngologists and analyzed for RT-QuIC and morphological studies. In 49 patients CSF was also obtained.

**Results**: At first round, 72 OM samples were tested by α–syn RT-QuIC assay and 35 resulted positive. 32/43 positive samples were from PD patients and 3/29 from non-PD yielding an overall sensitivity of 74% and 90% and a specificity of 90% but NS collected from AN were positive in 27 out of 32 (84%) and only in 5 out of 11 (45%) from MT. To confirm the influence of NS procedure on RT-QuIC results, 23 additional patients with PD underwent NS at both AN and MT. NS samples collected at AN were positive in 18/23 (78%) while those from MT in 10/23 (44%). We showed by immunocytochemistry that this difference might be related to a higher representation of olfactory neural cells in AN compared to MT. Alpha-syn and phospho-α-syn deposits were also found in NS from PD patients, but faintly in normal controls. Finally, RT-QuIC analysis of CSF was positive in 22/24 samples from PD patients (92% sensitive) and in 1/19 non-PD (95% specific).

**Conclusions**: In PD patients, RT-QuIC sensitivity is significantly increased when NS is performed at AN indicating that α-syn aggregates are preferentially found in olfactory areas with higher representation of OM. Finally, RT-QuIC analysis of CSF showed a high diagnostic accuracy strengthened by combination with NS.

**Funded by**: Fondazione Cariverona and Brain Research Verona Foundation.

**Acknowledgement**: We deeply thank Dr. Santina Castriciano (COPAN group) for providing nasal swabs.

**Protein misfolding cyclic amplification (PMCA) as an ultra-sensitive technique for the screening of CWD prions in different sample types**

Francisca Bravo‐Risi^a,b^, Paulina Soto^a,b^, Rebeca Benavente^a^, Hunter Reed^c^, Mitch Lockwood^c^, Tracy Nichols^d^, and Rodrigo Morales^a,b^

^a^Department of Neurology, The University of Texas Health Science Center at Houston, Houston, TX, USA; ^b^Centro Integrativo de Biologia y Quimica Aplicada (CIBQA), Universidad Bernardo O’Higgins, Santiago, Chile; ^c^Texas Park and Wildlife Department, Texas, USA; ^d^Veterinary Services Cervid Health Program, United States Department of Agriculture, Animal and Plant Health Inspection Service, Fort Collins, Colorado, USA

Chronic wasting disease (CWD) is a prion disease that affects farmed and free-ranging cervids. The infectious agent in CWD is a misfolded form of the prion protein (PrP^Sc^) that promotes conformational changes in the host’s cellular prion protein (PrP^C^). Currently, definitive CWD status is confirmed in the brain and lymphoid tissues by immunohistochemistry. The limitation of this technique is its poor sensitivity. Protein misfolding cyclic amplification (PMCA) and real-time quaking-induced conversion (RT- QuIC) are ultra-sensitive techniques that overcome these issues. PMCA mimics the self- propagation of infectious prions *in vitro* through multiple incubation/sonication cycles, increasing the number of prion particles present in a given sample. The detection of proteinase K (PK) -resistant PrP^Sc^by PMCA has been performed in experimental and natural samples that might harbor subclinical levels of prions. These samples include several tissues, bodily fluids, excreta, and different manmade and natural materials, including mineral licks, soils, and plants.

**Aims**: In this study, we highlight recent advances and contributions that our group has performed in the detection of CWD prions from samples collected in farmed and free-ranging cervids, as well as other specimens involving the environment that contains CWD-infected deer.

**Material and Methods**: A set of diverse samples analyzed in this study were collected by USDA and TPWD personnel in breeding and taxidermy facilities, and deer breeding facilities. These included animal and environmental samples. Additional samples from free-ranging animals were provided by hunters.

**Results**: The diverse range of samples successfully detected for CWD prion infection in this study include blood, semen, feces, obex, retropharyngeal lymph node, fetuses (neural and peripheral tissues) and gestational tissues, parasites, insects, plants, compost/soil mixtures, and swabs from trash containers. Importantly, these results helped to identify seeding-competent prions in places reported to be free of CWD. The levels of prion infectivity in most of these samples are currently being investigated.

**Conclusions**: Our findings contribute to the understanding of the transmission dynamics and prevalence of CWD. In addition, our data have helped to identify CWD in areas previously considered to be free of CWD. We also demonstrate that PMCA is a powerful technique for the screening of biological and environmental samples. Overall, our research suggests that PMCA may be a useful tool to implement for the surveillance and management of CWD.

**Funded by**: NIH/NIAID and USDA

**Grant number**: 1R01AI132695 (NIH) and AP20VSSPRS00C143 (USDA)

**Detection of CWD prion in feces of naturally infected, pre-symptomatic, North American white-tailed deer**

Francisca Bravo‐Risi^a,b^, Paulina Soto^a,b^, Rebeca Benavente^a^, Tracy Nichols^c^, and Rodrigo Morales^a,b^

^a^Department of Neurology, The University of Texas Health Science Center at Houston, Houston, TX, USA; ^b^Centro Integrativo de Biologia y Quimica Aplicada (CIBQA), Universidad Bernardo O’Higgins, Santiago, Chile; ^c^Veterinary Services Cervid Health Program, United States Department of Agriculture, Animal and Plant Health Inspection Service, Fort Collins, Colorado, USA

Chronic wasting disease (CWD) is a prion disease affecting cervids. Confirmatory testing of CWD is currently performed *postmortem* in obex and lymphoid tissues. Detection of CWD in live animals could enable the detection of CWD at earlier time points. Our group has explored CWD-prion detection in a variety of samples using the protein misfolding cyclic amplification (PMCA) technique. Extensive evidence demonstrates the presence of infectious prions in feces of CWD-infected deer using *in vitro* prion-amplification techniques and bioassays. In experimental conditions, this has been achieved as soon as 6-month post- inoculation in cervids, suggesting this sample type is a candidate for *antemortem* screening.

**Aims**: We optimized the detection of CWD prions in fecal samples from naturally infected, pre-symptomatic, white-tailed deer (WTD). The screening results were compared with those from blood with the purpose of identifying a suitable *antemortem* sample for diagnosis in terms of sensitivity. Our analysis also considered the genetic variability at position 96 of the prion protein.

**Material and Methods**: A set of 169 feces samples were collected by USDA personnel from farmed WTD. Deer displayed different *PRNP* polymorphisms at position 96. PMCA optimization for fecal samples was performed by comparing enrichment of CWD prions by NaPTA, ultracentrifugation, and direct spiking of the sample to the PMCA reactions.

**Results**: Direct spiked fecal samples resulted in the best PMCA efficiency. From our screening, fifty-eight samples were positive for CWD prions, providing a sensitivity of 55% and a specificity of 99%. Seventy-eight fecal sample results were compared with those from blood results (matched animals). Our data shows that at the early pre-symptomatic stages (EPS) of disease, 28% of fecal samples were positive, compared to the 47% of positivity in blood from the same animals. At late pre-symptomatic (LPS) stages, positive detection in both samples was similar: 84% (feces) and 87 % (blood). Interestingly, LPS animals coding for the 96 GG variant showed ≥ 93% of positivity in feces and blood, while samples from PrP96 GS animals showed ~70% of positivity in both specimens at LPS.

**Conclusions**: Our results demonstrate that CWD prion detection in feces performed best in the absence of sample pre-treatments and showed that the detection of prion seeding activity in both feces and blood is comparable at LPS stages. Overall, our findings contribute to understand prion distribution across different biological samples and polymorphic variants of WTD. This information is relevant for the current effort to identify platforms to diagnose CWD.

**Funded by**: NIH/NIAID

**Grant number**: R01AI132695

**Hypochlorous acid solutions reduce disease-associated tau seeding activity**

Danielle F. Browne, and Allison Kraus

Department of Pathology, Case Western Reserve University School of Medicine, Cleveland, USA

**Aims**: Neurodegenerative diseases, including tauopathies, often coincide with neuroinflammatory events. However, the degree to which neuroinflammation either furthers damages or has protective properties against neurodegenerative disease is not fully elucidated. One means through which neuroinflammation may modulate neurodegenerative disease is immune cell derived reactive oxygen species (ROS). ROS have been implicated in neurotoxicity and neurodegenerative disease progression, but the mechanisms by which this may occur are not understood. Hypochlorous acid (HOCl) is a ROS that is produced by innate immune cells in response to pathogens and/or stress signals. HOCl has been long known for its microbicidal activity, contributing to the clearance of unwanted bacteria, fungi, and viruses through deactivating chemical modifications. However, it is currently unknown if/how the production of HOCl influences the trajectory of neurodegenerative diseases characterized by the accumulation of misfolded, self-propagating protein pathogens. Previous data indicates HOCl pretreatment of PrP prions results in abrogation of their seeding activity and infectivity when reintroduced to a healthy animal.^a^Here, we extend these findings to investigate other self-propagating neurodegenerative disease proteins to test if HOCl mitigates seeding activities of brain derived tau seeds.

**Material and Methods**: The Real Time Quaking Induced Conversion (RT-QuIC) assay is a high-throughput, selective, in vitro seed amplification assay used to detect and quantify protein seeds from human biospecimens. RT-QuIC was utilized to quantitate HOCl effects on the seeding activity of tau seeds from brain tissue of Alzheimer disease cases and primary synucleinopathies with concurrent tau pathology. SDS-PAGE analysis was used to examine how changes in seeding activities correlated with structural changes to the tau seeds.

**Results**: HOCl reduced tau seeding activities up to 10,000-fold when compared to untreated controls. Tau seeds from primary tauopathies as well as co-pathologies in other diseases exhibited decreased seeding activity after HOCl treatment. SDS-PAGE analysis confirmed HOCl solutions structurally modified tau conformers derived from human brain tissue.

**Conclusions**: HOCl solutions modify and reduce seeding activities of disease-related brain derived tau seeds, providing preliminary evidence that interactions with this reactive oxygen species have the potential to drastically alter tau seed self-propagation properties. This could have implications for how in vivo ROS generation impacts seed accumulation and disease trajectories. With use of cell models, we will test if HOCl generation in vivo similarly modifies seeds. Assessing whether immune cells have the propensity to modify brain derived tau seeding activity will bolster the biological relevance of our findings and perhaps reveal a previously unknown mechanism of disease modulation.

Hughson A.G., Race B., Kraus A., Sangare L.R., Robins L., Groveman B.R., Saijo E., Phillips K., Contreras L., Dhaliwal V., Manca M., Zanusso G., Terry D., Williams J.F., and B. Caughey. Inactivation of Prions and Amyloid Seeds with Hypochlorous Acid. PLOS Pathogens **12**, e1005914 (2016)

**Funded by**: This work was supported by Case Western Reserve University.

**Acknowledgement**: We thank Dr. Xiongwei Zhu, Sandra Siedlak, and Dr. Mark Cohen at Case Western Reserve University, and Drs. Douglas Galasko, David Coughlin, and Annie Hiniker at University of California San Diego for characterization of and/or provision of brain tissue used in this project.

**Stable and highly zoonotic cervid prion strain is possible**

Manuel Camacho, Xu Qi, Liuting Qing, Sydney Smith, Jieji Hu, Wanyun Tao, Ignazio Cali, and Qingzhong Kong

Department of Pathology, Case Western Reserve University, Cleveland, USA

**Aims**: Whether CWD prions can infect humans remains unclear despite the very substantial scale and long history of human exposure of CWD in some areas. Multiple in vitro conversion experiments and in vivo animal studies suggest that the CWD-to-human transmission barrier is not unbreakable. A major public health concern on CWD zoonosis is the emergence of highly zoonotic CWD strains. We aim to address the question of whether highly zoonotic CWD strains are possible.

**Material and Methods**: We inoculated a few sCJD brain samples into cervidized transgenic mice, which were intended as negative controls for bioassays of brain tissues from sCJD cases who had hunted or consumed vension from CWD-endemic states. Some of these mice became infected and their brain tissues were further examined by serial passages in humanized or cervidized mice.

**Results**: Passage of sCJDMM1 in transgenic mice expressing elk PrP (Tg12) resulted in a ‘cervidized’ CJD strain that we termed CJD^ElkPrP^. We observed 100% transmission of CJD^ElkPrP^in transgenic mice expressing human PrP (Tg40h). We passaged CJD^ElkPrP^two more times in the Tg12 mice. We found that such second and third passage CJD^ElkPrP^prions also led to 100% infection in the Tg40h mice. In contrast, we and others found zero or poor transmission of natural elk CWD isolates in humanized mice, despite that natural elk CWD isolates and CJD^ElkPrP^share the same elk PrP sequence.

**Conclusions**: Our data demonstrate that highly zoonotic cervid prion strains are not only possible but also can be stably maintained in cervids and that CWD zoonosis is prion strain-dependent.

**Funded by**: NIH

**Grant number**: R01NS052319, R01NS088604, R01NS109532

**Acknowledgement**: We want to thank the National Prion Disease Pathology Surveillance Center and Drs. Allen Jenny and Katherine O’Rourke for providing the sCJD samples and the CWD samples, respectively.

**Validation of Plasma- and CSF-Neurofilament light chain as a marker for sporadic Creutzfeldt-Jakob disease**

Sezgi Canaslan Eyyuboglu^a,b^, Matthias Schmitz^a,b^, and Inga Zerr^a,b^

^a^Department of Neurology, University Medicine Göttingen; ^b^German Center for Neurodegenerative Diseases (DZNE), Göttingen, Germany

**Aims**: The importance of biomarkers for the differential diagnosis of neurodegenerative diseases is undeniable. They are not only helpful for the diagnosis of the disease but also be quite useful to track disease progression and the efficiency of the possible treatments. Previous findings showed that neurofilament (NfL) is one of the potential markers for sporadic Creutzfeldt-Jakob disease (sCJD) diagnosis. Therefore, we investigated NfL levels in our patient cohort. Firstly, we analysed the stability of NfL in different conditions and inter-and intra-assay variation both in plasma and CSF. Afterward, we measured the concentration of NfL in cohorts that include individuals with sCJD and different controls.

**Material and Methods**: We have measured NfL concentration using the ultra-sensitive method which is the Single Molecule Array (SIMOA). We have applied different conditions like incubation at RT; 4°C and several freeze-thaw cycles to find out the stability of NfL. We also examined the regulation of NfL in sCJD and control cohorts consisted of neurodegenerative controls, non-neurodegenerative controls, and healthy controls.

**Results**: In our analysis, we found that NfL in plasma and CSF is highly stable under defined storage conditions. Additionally, the coefficient variation of the inter-assay and intra-assay measurement of NfL remained in an acceptable range. When we analysed our patient cohorts, the level of NfL is visibly high in sCJD patients not only in their CSF but also in their plasma. To determine the diagnostic accuracy, we have applied ROC curve analysis which indicated high diagnostic accuracy for both CSF (80% sensitivity and 87% specificity) and plasma (exhibited 83.5% sensitivity and 91% specificity) to discriminate sCJD from controls. By virtue of a large cohort, the cut-off for CSF is ≥ 2180 pg/mL and for plasma is ≥ 47.83 pg/mL to distinguish sCJD from controls according to the *Youden* index.

**Conclusions**: Our findings showed and confirmed that NfL is a stable marker under different conditions in CSF and plasma. ROC curve analysis also showed perfect AUC values, which are 0.92 and 0.93, to distinguish sCJD from controls in CSF and plasma respectively.

**Effect of the induction of chronic stress on cellular models of Amyotrophic Lateral Sclerosis**

Niccolò Candelise^a,b^, Henri Zenuni^c^, Silvia Scaricamazza^b^, Illari Salvatori^a,b^, Valentina Nesci^b^, Tina Garofalo^a^, Vincenzo Mattei^a,d^, Maurizio Sorice^a^, Alberto Ferri^b,e^, Cristiana Valle^b,e^, and Roberta Misasi^a^

^a^Department of Experimental Medicine, Faculty of Medicine, ‘Sapienza’ University of Rome, Rome, Italy; ^b^IRCCS Fondazione Santa Lucia, Rome, Rome, Italy; ^c^Unit of Neurology, Department of Systems Medicine, University of Roma Tor Vergata, Rome, Italy; ^d^Biomedicine and Advanced Technologies Rieti Center, Sabina Universitas, Rieti, Italy; ^e^Institute of Translational Pharmacology (IFT), Consiglio Nazionale delle Ricerche (CNR), Rome, Italy

**Aims**: Amyotrophic Lateral Sclerosis (ALS) is a neurodegenerative disease affecting the upper and lower motor neurons. The hallmark of ALS is the presence of inclusions made of TDP-43 formed through prion-like misfolding, a common process in neurodegenerative diseases. TDP-43 is a nuclear protein that localizes in the cytosol upon acute stress insults. However, little is known about TDP-43 biology upon milder and prolonged insults, a condition closer to pathology compared to acute stress. Although metabolic dysfunctions and TDP-43 aggregates are present in ALS patients before neurodegeneration, the interplay between TDP-43 aggregation and bioenergetic derangement is poorly understood. Hence, we aim to set a chronic stress paradigm on neuronal cell lines that would better mirror the long-lasting events leading to TDP-43 proteinopathy.

**Material and Methods**: Different paradigms of chronic stress were applied to neuronal SH-SY5Y cultures for 72 hours and tested for cell viability by MTS assay. Stressors included: Serum deprivation (Sd, 1% – 0,1% FBS, inducing environmental stress); Sodium Arsenite (Ars, 10–20 µM, inducing oxidative stress); Paraquat (PQ, 0,1–0,2 mM, source of oxidative stress); Sorbitol (Sorb, 40–120 mM, causing osmotic stress). Cell viability from chronic stress conditions were compared with results obtained with established acute stress paradigms (0,5 mM Ars, 1 h; 1–2 mM PQ, overnight; 0,6–1,2 M Sorb, 2 h). Cell viability data were combined with bioenergetic parameters measured by Seahorse analyzer. Western Blot analyses were performed after protein extraction with RIPA buffer to assess the solubility of TDP-43 under acute and chronic stressful conditions. TDP-43 localization after stress induction was observed by immunofluorescence.

**Results**: Sub-lethal concentrations of Sd, Ars, PQ and Sorb were identified by MTS assay. Bioenergetic analyses suggested a decrease in functionality of major mitochondrial complexes upon both acute and chronic treatment. Western Blot analyses revealed the formation of RIPA-insoluble species of TDP-43 upon chronic treatment. Immunofluorescence experiments showed different aggregation patterns of TDP-43, which may imply that different pathways may be involved in response to prolonged stress.

**Conclusions**: Here we propose a novel cellular paradigm based on prolonged and mild treatment for the study of TDP-43 pathology. By extension, this system may further be suitable for the study of other proteins involved in neurodegenerative disorders such as alpha-synuclein, prion protein and Tau.

**Funded by**: ‘*Giovani@RicercaScientifica’* Fondazione Cassa di Risparmio di Pistoia e Pescia.

**Grant number**: CUP B89J22001910007

**Conformational shift as the evolutionary mechanism for classical BSE emergence from atypical scrapie**

Sara Canoyra, Alba Marín-Moreno, Juan Carlos Espinosa, Natalia Fernández- Borges, and Juan María Torres

Centro de Investigación en Sanidad Animal, CISA-INIA-CSIC, Valdeolmos, Madrid, Spain

**Aims**: New prion strains emerge when the prion conformational characteristics change during intra- or cross-species transmission. There are two main theories, non-mutually exclusive, that could explain this phenomenon: the ‘deformed templating’ and the ‘conformational selection model’. According to the ‘deformed templating’ or mutation model, when the prion is unable to replicate in a new host there is a shift to a new PrP^Sc^conformation. On the other hand, the ‘conformational selection’ theory postulates that prion isolates are a conglomerate of conformations and during cross-species transmission the species barrier acts as a filter.

In previous studies, we showed the emergence of the bovine spongiform encephalopathy agent (C-BSE) due to the transmission of atypical scrapie (AS) onto bovine PrP. This work will elucidate the evolutionary dichotomy in the AS transmission, providing supporting evidence on the hypothesis of the origin of the epidemic C-BSE prion from AS.

**Material and Methods**: A panel of AS isolates with different genotypes and geographical distribution was analyzed. To differentiate between AS and C-BSE two strain typing features were used: thermostability and PMCA propagation. The AS isolates underwent a heat treatment of 98°C during 2 h and were amplified *in vitro* by PMCA in bovine PrP^C^substrate. The templating activity with or without heat was determine after 10 amplification rounds by western blot characterization.

In addition, we analyzed an artificial mixture of AS and C-BSE generated by diluting C- BSE in a constant amount of AS.

**Results**: We observed a drastic loss in the C-BSE emergence due to the heat treatment. The AS is a thermolabile prion. Hence, the inactivation of the AS conformers with the ability to shift the conformation will slow down the emergence of the C-BSE.

In contrast, when we analyzed the artificial mixture C-BSE prions emerge even with the heat treatment. Therefore, if the AS isolates had contained a minoritarian C-BSE conformer (defended by the conformational selection model) the emergence wouldn’t have been affected by the heat.

**Conclusions**: Mutation is the main evolutionary mechanism responsible for the C-BSE emergence. The species barrier forces the shift to a possible structure (C-BSE in this case) in a thermodynamically unfavorable process.

This discovery reenforces the origin hypothesis of the epidemic C-BSE as a contact of the cattle with feed contaminated with AS. Where the AS will evolve shifting to a C-BSE stable conformation. This also has implications in the control of farmed animals and humans’ exposure to the AS.

**Funded by:/Grant number**: Project PID2019-105837RB-I00 MCIN/ AEI /10.13039/501,100,011,033 Fundación La Marató de TV3 Enfermedades

**The chronic wasting disease agent from white-tailed deer is infectious to humanized mice after passage through raccoons**

Eric Cassmann^a^, Xu Qi^b^, Qingzhong Kong^b^, and Justin Greenlee^a^

^a^National Animal Disease Center, Agricultural Research Service, US Department of Agriculture, Ames, IA, USA

^b^Departments of Pathology, Neurology, National Center for Regenerative Medicine, and National Prion Disease Pathology Surveillance Center, Case Western Reserve University, Cleveland, Ohio, USA

**Aims**: Evaluate the zoonotic potential of the raccoon passaged chronic wasting disease (CWD) agent in humanized transgenic mice in comparison with the North American CWD agent from the original white-tailed deer host.

**Material and Methods**: Pooled brain material (GG96) from a CWD positive herd was used to oronasally inoculate two white-tailed deer with wild-type prion protein genotype and intracranially inoculate a raccoon. Brain homogenates (10% w/v) from the raccoon and the two white-tailed deer were used to intracranially inoculate separate groups of transgenic mice that express human prion protein with methionine (M) at codon 129 (Tg40h). Brains and spleens were collected from mice at experimental endpoints of clinical disease or approximately 700 days post-inoculation. Tissues were divided and homogenized or fixed in 10% buffered neutral formalin. Immunohistochemistry, enzyme immunoassay, and western blot were used to detect misfolded prion protein (PrP^Sc^) in tissue.

**Results**: Humanized transgenic mice inoculated with the raccoon passaged CWD agent from white-tailed deer exhibited a 100% (12/12) attack rate with an average incubation period of 605 days. PrP^Sc^was detected in brain tissue by enzyme immunoassay with an average optical density of 3.6/4.0 for positive brains. PrP^Sc^also was detected in brain tissue by western blot and immunohistochemistry. No PrP^Sc^was detected in the spleens of mice inoculated with the raccoon passaged CWD agent. Humanized mice inoculated with the CWD agent from white-tailed deer did not have detectable PrP^Sc^using conventional immunoassay techniques.

**Conclusions**: The host range of the CWD agent from white-tailed deer was expanded in our experimental model after one passage through raccoons.

**Funded by**: This research was funded in its entirety by congressionally appropriated funds to the United States Department of Agriculture, Agricultural Research Service. The funders of the work did not influence study design, data collection and analysis, decision to publish, or preparation of the manuscript.

**Acknowledgement**: We thank Quazetta Brown, Lexi Frese, Rylie Frese, Kevin Hassall, Leisa Mandell, and Trudy Tatum for providing excellent technical support to this project.

**Beta-endoproteolysis of the cellular prion protein by dipeptidyl peptidase-4 and fibroblast activation protein**

Andrew R. Castle^a,b^, Sang-Gyun Kang^a,b^, Ghazaleh Eskandari-Sedighi^a,c^, Serene Wohlgemuth^a,b^, My-Anh Nguyen^f,g^, Daniel J. Drucker^d,e^, Erin E. Mulvihill^f,g^, and David Westaway^a,b,c^

^a^Centre for Prions and Protein Folding Diseases, University of Alberta, Edmonton, Canada; ^b^Department of Medicine, University of Alberta, Edmonton, Canada; ^c^Department of Biochemistry, University of Alberta, Edmonton, Canada; ^d^Lunenfeld-Tanenbaum Research Institute, Mt. Sinai Hospital, Toronto, Canada; ^e^Department of Medicine, University of Toronto, Toronto, Canada; ^f^University of Ottawa Heart Institute, Ottawa, Canada; ^g^Department of Biochemistry, Microbiology and Immunology, University of Ottawa, Ottawa, Canada

**Aims**: β-Endoproteolysis, which splits PrP^C^into the N-terminal fragment N2 and the C-terminal fragment C2, is of interest because a protease-resistant, C2-sized fragment (‘C2^Sc^’) accumulates in the brain during prion infections, seemingly comprising the majority of PrP^Sc^at disease endpoint in mice. However, candidates for the underlying proteolytic mechanism remain unconfirmed *in vivo*. We therefore set out to investigate this important phenomenon.

**Materials and Methods**: We performed a cell-based screen of protease inhibitors using capillary westerns to study PrP^C^fragmentation. Hits were rescreened to obtain dose-response curves and were pursued further by the following approaches: *i)* acute co-transfections of PrP alleles and protease genes; *ii)* analyses of recombinant PrP substrates exposed to recombinant proteases; *iii)* Edman sequencing to assign N-termini; *iv)* analyses of C2 fragment levels in protease-knockout mice; *v)* testing of relevant protease inhibitors in cell-based models of prion infections.

**Results**: The protease inhibitor screen unexpectedly linked type II membrane proteins of the S9b serine peptidase subfamily to PrP^C^β-cleavage. Co-transfection experiments in cells and assays with recombinant proteins confirmed that fibroblast activation protein (FAP) and its paralog, dipeptidyl peptidase-4 (DPP4), cleave directly at multiple sites within the N-terminal domain of PrP^C^. For wild-type mouse and human PrP^C^substrates expressed in cells, the rank orders of activity were human FAP ~ mouse FAP > mouse DPP4 > human DPP4 and human FAP > mouse FAP > mouse DPP4 ≫ human DPP4, respectively. C2 levels relative to total PrP^C^were reduced in several tissues from FAP-null mice, and, while knockout of DPP4 lacked an analogous effect, the combined DPP4/FAP inhibitor linagliptin, but not the FAP-specific inhibitor SP-13786, reduced C2^Sc^and total PrP^Sc^levels in two murine cell-based models of prion infections.

**Conclusions**: We have identified two closely-related proteases that can perform β-cleavage of PrP^C^. Beyond opening up a potential new avenue for therapeutic intervention against prion diseases, our findings may lead to new axes of investigation, given the published biologies of DPP4 and FAP in neuropeptide processing, cellular senescence and tissue remodelling.

**Funded by**: Canadian Institutes of Health Research; Campus Alberta Neuroscience; Alberta Prion Research Institute

**Grant number**: 165980; CANNEGP; APRIIEP201600033

**Role of APOE vs Aβ in prion models of tau pathology**

P. Chakrabarty^a,b,c^, T. Williams^a,b^, G. Xu^a,b^, B. Moore^a,b^, Q. Vo^a,b^, A J. Ruiz^a^, P. Sullivan^d^, B. Giasson^a,b,c^, and D. Borchelt^a,b,c^

^a^Center for Translational Research in Neurodegenerative Disease, University of Florida, Gainesville, USA; ^b^Department of Neuroscience, University of Florida, Gainesville, USA; ^c^McKnight Brain Institute, University of Florida, Gainesville, USA; ^d^Department of Medicine, Duke University, Durham, USA

**Aims**: Alzheimer’s disease and related tauopathies are characterized by progressive transmission of aggregated tau and amyloid β (Aβ) along neuroanatomically connected brain regions. Our aim was to broadly investigate the relative contribution of Apolipoprotein E (APOE) and Aβ in modulating tau pathology in tau prion-seeded polygenic models of Alzheimer’s disease.

**Material and Methods**: We combined the human P301S mutant tau mice (Line PS19) with human APOE3tr and APOE4tr mice and examined the outcomes of hippocampal seeding of K18-tau aggregates. Mice were injected at 2.5 month of age and analyzed at 7.5 months of age. We also combined PS19 mice with human APPswe/ind mutant transgenic APPsi mice and examined the effects of intracerebral seeding with amyloid-enriched mouse brain homogenates from various sources. Neonatal PS19xAPPsi mice were injected with the seeding homogenates and analyzed at 9 months of age.

**Results**: The first set of experiments allowed us to generate insights into how APOE4, the major risk factor for Alzheimer’s disease, regulates intracerebral propagation of K18-tau prion induced tauopathy. K18-tau seeded PS19xAPOE3tr mice accumulated higher phosphorylated tau and microgliosis relative to PS19xAPOE4tr mice, but neurofibrillary tangle burden was not affected. PS19 mice that were heterozygous for APOE3 showed similar results, albeit to a lesser degree. A focused transcriptomic study revealed modest changes in gene expression associated with tau propagation, with more changes evident in APOE4tr mice compared to APOE3tr mice. In the second set of experiments, we used PS19xAPPsi mice. At 9 months of age, these mice had no Aβ deposits and only 2 of 7 bigenic mice showed MC1 reactive pre-tangle pathology. Seeding with amyloid-enriched homogenates from three different transgenic APP sources robustly induced Aβ deposits in PS19xAPPsi mice (19 of 19 injected mice showed pathology; n = 6–7 for each Aβ source). Most of the animals with induced Aβ pathology (n = 16/19 seeded mice) also showed high burden of MC1 and Gallyas positive tau tangle pathology.

**Conclusions**: We demonstrate that the presence of human APOE3 triggered accumulation of phosphorylated tau in PS19 mice following K18-tau seeding. Seeding did not differentially induce tau tangle in PS19xAPOE3tr and PS19xAPOE4tr mice. In the combined presence of tau and APP overexpression, seeding with amyloid enriched mouse brain homogenates induced both Aβ deposits and tau tangles. Overall, these findings indicate that Aβ is a relatively stronger modulator of tau prion-induced tau misfolding than APOE.

**Funded by:/Grant number**: NIA RF1AG057933 (DRB, PC), NIA T32 AG061892 (TW), Ed & Ethel Moore Alzheimer’s disease Research Grant (GX).

**PrP^Sc^aggregation state does not affect efficiency of peripheral infection in two CWD strains**

Sheng C. Chang, Samia Hannaoui, and Sabine Gilch

Department of Comparative Biology and Experimental Medicine, Faculty of Veterinary Medicine, University of Calgary, Calgary, Canada

**Aims**: PrP^Sc^exists in various strain-specific aggregation states, and PrP^Sc^oligomers have been shown to be the most infectious isoforms upon intracerebral inoculation. We aimed to determine the most infectious particle size upon infection directly into the brain versus through the periphery in an *in vivo* model, and to explore the impact of PrP^Sc^aggregation states on neuroinvasion efficiency using *in vitro* models.

**Material and Methods**: We solubilized and fractionated mouse-adapted Wisc-1 and 116AG CWD prions into thirty different fractions through a sedimentation velocity gradient. Fractions were collected and the quantity of total PrP and PK-resistant PrP^Sc^that each fraction represents as a ratio of the entire gradient were determined through western blot after treatment without or with proteinase K. The same fractions were also inoculated intracerebrally and intraperitoneally into gene-edited mice expressing cervid PrP. The animals were sacrificed upon the onset of terminal illness, and the survival figures were analyzed. To determine degradation kinetics of different PrP^Sc^aggregate, we exposed J774 macrophages to fractionated scrapie or CWD prions. Cells at various time points post-exposure were harvested and cell lysates were subjected to solubilization in anionic detergent and ultracentrifugation, following which the resulting pellet fraction consisting of PrP^Sc^was quantified with western blot to determine the uptake and degradation kinetics of the different prion aggregates in the immune cells.

**Results**: The greatest quantity of PK-resistant PrP^Sc^in Wisc-1 CWD was between fractions 9–12, and for 116AG CWD this was slightly lower in the gradient between fractions 10–14. We found that in both the animals inoculated intracerebrally and intraperitoneally, fractions 10–16 and 24–30 in the middle and the bottom of the gradient that correspond, respectively, to small oligomers and fibrils led to shortest survival. Interestingly, fibrillar PrP^Sc^from fractions 22–30 of the gradient were more resistant to degradation by cultured macrophages.

**Conclusions**: In our CWD model, prion neuroinvasion and infectivity from the periphery are not dictated by the aggregation state of inoculated prions. Rather, these properties are likely determined by intrinsic properties specific to each individual prion strain, despite the different processing of the different aggregates in the periphery by immune cells.

**Funded by**: Alberta Prion Research Institute, Natural Sciences and Engineering Research Council of Canada, University of Calgary

**Acknowledgement**: Dr. Debbie McKenzie and Dr. Trent Bollinger for providing us the cervid prion isolates used to generate the passaged material for this study, Dr. Walker Jackson and Dr. Lech Kaczmarzcyk for providing us the embryonic stem cells used to generate the cervid-PrP-expressing gene-edited mice, and Shranjit Lail and Dr. Robin Yates for providing us with the J774 macrophage cell line.

**GSS A117V and a mouse model expressing bank vole PrP^C^as a fast and versatile model to monitor potential treatments for human prion diseases**

Jorge M. Charco^a,b,l^, Tomás Barrio^c,*^, Hasier Eraña^a,b,l^, Carlos Díaz-Domínguez^a^, Cristina Sampedro-Torres-Quevedo^a^, Enric Vidal^e^, Izaro Kortazar-Zubizarreta^f^, Guiomar Pérez de Nanclares^g^, Steffen Halbgebauer^h^, Mariví Geijo^i^, Glenn Telling^j^, Markus Otto^k^, and Joaquín Castilla^a,d,l^

**^a^**Center for Cooperative Research in Biosciences (CIC bioGUNE), Basque Research and Technology Alliance (BRTA). Bizkaia Technology Park. Derio. Spain; **^b^**ATLAS Molecular Pharma S. L. Bizkaia Technology Park. Derio. Spain; **^c^**UMR ENVT-INRAE 1225 Interactions Hôtes-Agents Pathogènes (IHAP). École Nationale Vétérinaire de Toulouse. Chemin des Capelles, 23. Toulouse. France * These authors contributed equally to this work; **^d^**IKERBASQUE, Basque Foundation for Science, Prion Research Lab. Bilbao. Spain; **^e^**Unitat mixta d’Investigació IRTA-UAB en Sanitat Animal. Centre de Recerca en Sanitat Animal (CReSA). Campus de la Universitat Autònoma de Barcelona (UAB). Bellaterra. Catalonia; **^f^**Department of Neurology, Bioaraba Health Research Institute, Araba University Hospital – Txagorritxu, Vitoria-Gasteiz. Spain; **^g^**Molecular (Epi)Genetics Laboratory, Bioaraba Health Research Institute, Araba University Hospital, Vitoria-Gasteiz. Spain; **^h^**Institute of Experimental Neurology, Ulm University Hospital. Helmholtz Strasse. Ulm. Germany; **^i^**Animal Health Department, NEIKER-Basque Institute for Agricultural Research and Development, Basque Research and Technology Alliance (BRTA), Parque Científico y Tecnológico de Bizkaia, P812, E-48160 Derio, Spain; **^j^**Prion Research Center (PRC), Colorado State University. Fort Collins, Colorado. USA; **^k^**Departament of Neurology, Martin Luther University Halle-Wittenberg, Halle (Saale), Germany; **^l^**Centro de Investigación Biomédica en Red de Enfermedades infecciosas (CIBERINFEC), Carlos III National Health Institute, Madrid, Spain

**Aims**: To develop an animal model of human prion disease that meets the ideal conditions to evaluate future anti-prion therapies.

**Material and Methods**: TgVole (1x) mouse model expressing bank vole (I109I) PrP at approximately physiological levels was used in this project. A brain homogenate from a GSS A117V patient was used for the intraperitoneal and intracerebral inoculation of these animals. For NfL and β-synuclein sera quantification, a SIMOA Immunoassay system was used.

**Results**: GSS A117V propagates intracerebrally into TgVole (1x) with high specific infectivity and an extremely short incubation period of 67 ± 1 dpi. Additionally, GSS A117V also propagates intraperitoneally very efficiently with a period of 87 ± 1 dpi. A kinetic study after intraperitoneal inoculation allowed us to observe increasing levels of NfL and β- synuclein detectable at short times during the pre-clinical stage, which allows an accurate way to monitor the disease process.

**Conclusions**: The combination of a very short incubation period after intracerebral and, more importantly, intraperitoneal inoculation allows monitoring of disease progression by means of serum biomarkers (NfL and β-synuclein). These results make this combination of mice and inoculum an ideal *in vivo* model for preclinical studies focused on future therapeutic approaches for human transmissible spongiform encephalopathies.

**Funded by:/Grant number**: PID2021-122201OB-C21 and CJDF Grant 2022

**Acknowledgement**: Spanish Ministry of Science and Innovation and USA CJD Foundation

**Identification of biomarker panels for differential diagnosis of Neurodegenerative Disorders**

Athanasia Chatziefstathiou^a^, Sezgi Canaslan^b^, Eirini Kanata^c^, Konstantinos Vekrellis^d^, Elisabeth Kapaki^e^, Matthias Schmitz^b^, Inga Zerr^b^, Konstantinos Xanthopoulos^c^, Dimitra Dafou^a^, and Theodoros Sklaviadis^c^

^a^School of Biology, Aristotle University, Thessaloniki, Greece; ^b^Department of Neurology, University Medical Center and the German Center for Neurodegenerative Diseases (DZNE), Göttingen, Germany; ^c^School of Pharmacy, Aristotle University, Thessaloniki, Greece; ^d^Center of Basic Research, Biomedical Research Foundation Academy of Athens (BRFAA), Athens Greece; ^e^Department of Neurology, Neurochemistry Unit National and Kapodistrian University, Athens, Greece

**Aims**: Recent reviews of epidemiological studies and reports from the WHO (World Health Organization), estimated that there were 24.3 million people with dementia in the world in 2001 and predicted that this would rise to 42.3 million in 2020, and 81.1 million patients by 2040. Therefore, it is necessary to exploit already established biomarkers, identify, and establish novel ones to determine appropriate biomarker panels that will allow the early detection and differential diagnosis of Neurodegenerative Disorders. The main goal of the current study is the identification of a suitable combination of different biomarkers in cerebrospinal fluid (CSF) and plasma to either distinguish different types of Neurodegenerative Disorders and controls (healthy and/or neurological).

**Material and Methods**: We analyzed different protein markers such as glial fibrillary acidic protein (GFAP), neurofilament light chain (NF-L), total tau (TAU) and ubiquitin carboxyl-terminal hydrolase L1 (UCH-L1) by using an ultrasensitive test called single-molecule array assay (SIMOA). A cohort of Alzheimer Disease (AD) and Frontotemporal Dementia (FTD) patients were analyzed using plasma and CSF from matched patients.

**Results**: We observed that CSF-NF-L, CSF-TAU & CSF-UCH-L1 were significantly elevated in AD patients compared to control donors. Receiver operating characteristic (ROC) curve analysis resulted in a very good diagnostic accuracy as indicated by the area under the curve (AUC) values of 0.84–0.93 & p < 0.0001. We also observed that CSF-NF-L & CSF-UCH-L1 were significantly elevated in FTD patients compared to controls, with an AUC of 0.76 and 0.82, respectively and p < 0.01. Furthermore, we also observed an increase on CSF-GFAP in AD and control group compared to FTD patients, with an AUC of 0.80 & 0.79 and p < 0.01. Finally, spearman correlation for NF-L showed an association between CSF and plasma samples in patients with AD and FTD, r = 0.4852 & r = 0.6703, p < 0.05, respectively.

**Conclusions**: Our study suggests NF-L and UCH-L1 as promising markers to discriminate between AD or FTD and controls, while GFAP is also a promising marker in discriminating AD and controls from FTD patients.

**Funded by**: This research has been co‐financed by the European Regional Development Fund of the European Union and Greek national funds through the Operational Program Competitiveness, Entrepreneurship and Innovation, under the call RESEARCH – CREATE – INNOVATE (project code:T1EDK-03884)

**Acknowledgement**: The research work was supported by the Hellenic Foundation for Research and Innovation (H.F.R.I) under the 3^rd^Call for H.F.R.I. PhD Fellowships (Fellowship Number: 6325)

**Yeast models for studying aggregation of proteins, involved in Alzheimer’s disease**

Y. Chernoff^a^, Z. Deckner^b^, P. Chandramowlishwaran^a^, A. Hirsch^a^, R. Mezencev^a^, L. Walker^b^, and D. Lynn^c^

^a^School of Biological Sciences, Georgia Institute of Technology, Atlanta, GA, USA; ^b^School of Medicine and Emory University, Atlanta, GA, USA; ^c^Departments of Chemistry and Biology, Emory University, Atlanta, GA, USA

**Aims**: Cross-β fibrous aggregates (amyloids) of human Aβ and tau proteins are linked to Alzheimer’s disease. Mechanisms of amyloid formation and propagation are still poorly understood due to the complexity of the human organism. Heritable endogenous amyloids, found in yeast cells and termed yeast prions, provide a powerful tool for the investigation of these processes. We have developed yeast assays for prion properties of Aβ and tau.

**Material and Methods**: Our constructs employ fusions of Aβ or tau to either various fragments of the yeast prion protein Sup35, or to various fluorescent proteins (FPs). Fusions to Sup35 allow for phenotypic detection of a prion, formed by a chimeric protein. Fusions to FP allow for cytological detection of aggregation and biochemical characterization of aggregates.

**Results**: We have demonstrated the ability of Aβ amyloids (including those from brain extracts) to transfect yeast cells, followed by propagation of various Aβ-based polymorphs (strains) in yeast. Impact of chemicals and Aβ mutations on amyloidogenic properties of Aβ has been studied by using yeast assays. Molecular basis of aggregates formed by Aβ and tau proteins in yeast cells has been characterized. Colocalization between tau and Aβ aggregates has been detected.

**Conclusions**: Our data show that yeast model recapitulates the major features of Aβ and tau aggregation, observed in humans, and is applicable to understanding mechanisms of aggregation.

**Funded by**: NIH

**Acknowledgement**: Support from Emory Alzheimer’s Disease Research Center.

**Loss of Rab7 activation leads to the impairments in cholesterol metabolism in prion infection**

Pearl Cherry^a,b^, Samia Hannaoui^a,b^, SuY Shim^a,b^, Vincent Ebacher^b^, Waqas Tahir^a,b^, Li. Lu^a,b^, Hermann Schätzl^a,b^, and Sabine Gilch^a,b^

^a^Calgary Prion Research Unit, Comparative Biology and Experimental Medicine, Faculty of Veterinary Medicine, University of Calgary, Calgary, Canada; ^b^Hotchkiss Brain Institute, Cumming School of Medicine, University of Calgary, Calgary, Canada

**Aims**: Prion diseases are caused by the accumulation of infectious PrP^Sc^, which is the misfolded isoform of cellular prion protein-PrP^c^. In prion infected neurons, certain cellular impairments including elevating cholesterol levels are observed. The aim of my study was to elucidate the causal mechanism of impaired cholesterol metabolism upon prion infection, focusing on the role of Rab7.

**Material and Methods**: Quantitative analysis of active Rab7 (Rab7.GTP) levels at different stages of prion infection in cerebellar granular neurons (CGN), 22 L-N2a cells and 22 L infected terminal mouse brains, and the trafficking dynamics of low-density lipoprotein (LDL) in 22 L-CAD5 cells were analyzed by confocal microscopy. Free cholesterol levels were visualized by filipin stating. Feedback regulation of cholesterol synthesis in prion infection, was measured via quantitative PCR. Impairments in Rab7 ubiquitination and its interactions with effector proteins upon prion infection were analyzed via immune pull down and subsequent immunoblotting. Over-expression of a constitutively active mutant of Rab7 in 22 L-N2a cells followed by amplex cholesterol assay and detection of PrP^Sc^was used to analyse the effect of Rab7 on cholesterol levels and prion propagation.

**Results**: We found that de novo prion infection of CGN is associated with elevated active-Rab7 levels at the early stages of infection, followed by a gradual loss in its levels, concomitant with increased cholesterol levels and PrP^Sc^accumulation. The loss in active Rab7 levels was also observed in neurons of 22 L-infected terminal mouse brains. The loss in active Rab7 levels upon prion infection causes a delay in LDL trafficking to lysosomes, leading to defective feedback regulation of cholesterol metabolism, which triggers de novo cholesterol synthesis in prion infected neuronal cell lines. Prion infection results in compromised ubiquitination of Rab7, which can subsequently impair its activation process. Rab7 effector interaction is reduced in both prion-infected neuronal cells and terminal mouse brains resulting in defective retrograde lysosomal transport. Over-expression of an active mutant of Rab7 in prion-infected cells restores normal cholesterol levels and reduces prion propagation, demonstrating a central role of Rab7 in regulating cholesterol metabolism and PrP^Sc^propagation.

**Conclusions**: We have demonstrated that loss in Rab7 activation upon prion infection causes dysregulations in cholesterol metabolism. The reduced activation of Rab7 can be linked to its compromised ubiquitination status. Rescuing the LDL trafficking impairments upon loss of active Rab7 reduces elevated cholesterol levels and PrP^Sc^propagation. Our results imply that Rab7 activity is critically important in regulating cholesterol levels and hence attenuating prion propagation.

**Funded by**: This research was funded by the Canada Research Chairs program and grants from the Alberta Prion Research Institute and the Canadian Institutes of Health Research to S.G. P.C. was supported by a University of Calgary Eyes High Graduate Student Fellowship

**Grant number**: 201,600,035

**Inoculation of human traumatic brain injury tissue homogenates induces cognitive deficits and widespread tau pathology in wild-type mice**

Roberto Chiesa^a^, Gloria Vegliante^a^, Ilaria Raimondi^a^, Elena Restelli^a^, Ilaria Lisi^a^, Federica Paredi^a^, Fabrizio Ortolano^b^, Marco Carbonara^b^, and Elisa R Zanier^a^

^a^Department of Neuroscience, Istituto di Ricerche Farmacologiche Mario Negri, Milan, Italy; ^b^Department of Anaesthesia and Critical Care, Fondazione IRCCS Cà Granda Ospedale Maggiore Policlinico, Milan, Italy

**Aims**: Tau pathology has been hypothesized to spread by a prion-like mechanism by which pathological tau conformers seed misfolding of normal tau. We have demonstrated that a single severe traumatic brain injury (TBI) in C57BL/6 J (WT) mice induces a self-propagating tau pathology that progressively spreads in the brain and can be horizontally transmitted to naïve mice, causing synaptic degeneration and cognitive impairment.

The aim of this study was to test whether a transmissible tau pathology is generated also in TBI patients.

**Material and Methods**: Fresh-frozen human traumatic brain injured (hTBI) tissues surgically removed for refractory intracranial hypertension (n = 3 patients, GCS at admission <8) and characterized biochemically for the presence of both total and hyperphosphorylated tau (P-tau), were homogenized (10% w/v in PBS) and inoculated bilaterally in the hippocampus and overlaying cortex of male WT and tau knockout (KO) mice. A glioma brain specimen was used as control (hCT). The cognitive function of inoculated mice was assessed by the novel object recognition (NOR), Radial Arm Water Maze (RAWM) and Y-maze tests up to 14 months post-inoculation (mpi). Synaptic loss and tau deposition were evaluated by immunohistochemistry at sacrifice.

**Results**: Early and persistent memory deficits were observed in mice inoculated with hTBI1 homogenate as assessed by NOR at 4, 8 and 12 mpi (hTBI1 vs hCT, *p < 0.05), associated with widespread tau pathology and synaptic loss (V-GLUT1 and drebrin, hTBI1 vs hCT, *p < 0.05). A similar memory impairment was induced by hTBI2 in NOR and RAWM at 4 and 10 mpi respectively (hTBI2 vs naïve, *p < 0.05). Interestingly, hTBI3, which had lower levels of both total and P-tau than the other homogenates (total tau: hTBI1/2 vs hTBI3, **p < 0.01; CP13: hTBI1/2 vs hTBI3, *p < 0.05), did not significantly impair cognitive function of inoculated mice. Semiquantitative analysis found that hTBI1 induced the largest pathological tau deposition in the hippocampus in terms of PHF1-positive neurons in the CA2-3 regions, and CP13 staining of apical dendrites in the stratum radiatum of the CA1 region. Thus, hTBI1 was selected for additional inoculations in WT and tau KO mice. The latter showed preserved memory function as compared to WT inoculated mice, consistent with a key role of endogenous tau in the spreading of tau proteinopathy.

**Conclusions**: Self-templating pathological tau is induced by a single TBI in humans and plays a key role in exacerbating post-traumatic pathology.

**Funded by**: Alzheimer’s Association, USA

**Grant number**: AARG-17532633

**Acknowledgement**: We thank Ilaria Bertani for participating in the initial phase of this project.

**A Field-Deployable Diagnostic Assay for the Visual Detection of Chronic Wasting Disease Prions**

Peter R. Christenson^a,b^, Manci Li^b,c^, Gage Rowden^b,c^, Marc D. Schwabenlander^b,c^, Tiffany M. Wolf^b,d^, Sang-Hyun Oh^a,b^, and Peter A. Larsen^b,c*^

^a^Department of Electrical and Computer Engineering, University of Minnesota, Minneapolis, MN USA; ^b^Minnesota Center for Prion Research and Outreach, University of Minnesota, St. Paul, MN USA; ^c^Department of Veterinary and Biomedical Sciences, University of Minnesota, St. Paul, MN USA; ^d^Department of Veterinary and Population Medicine, University of Minnesota, St. Paul, MN USA

**Aims**: Chronic wasting disease (CWD), a prion proteinopathy of cervids, is spreading across the United States and Canada, and has been detected in South Korea and Northern Europe. Field-deployable diagnostic tools for the rapid and reliable detection of CWD are limited. Gold nanoparticles (AuNPs) facilitate sensitive and reliable diagnostic techniques via visual color change for the detection of a variety of targets. In parallel, recently developed QuIC assays leverage protein-amplification and fluorescent signaling for the accurate detection of misfolded proteins, especially misfolded prion proteins. To advance field based CWD diagnostics, we combine AuNPs and QuIC technologies to create the Minnesota Quaking Induced Conversion (MN-QuIC™) assay, a field deployable method for the detection of CWD using the naked eye.

**Material and Methods**: In MN-QuIC™, tissue samples are amplified by incubating/shaking in recombinant prion rich solutions for 20–24hrs. After amplification, solutions are injected into an AuNP solution. AuNP solutions from CWD positive tissues appear visually red and while CWD not detected solutions appear visually blue. For laboratory testing, retropharyngeal lymph nodes (RPLN) and tonsil samples from wild white-tailed deer (WTD) were tested. The status of all samples was independently confirmed with ELISA and/or IHC. To demonstrate the utility of MN-QuIC™ outside the laboratory, we deployed to a rural wildlife surveillance field station where we performed MN-QuIC™ on 13 wild WTD in a blind study.

**Results**: Laboratory testing consisted of 60 tissues (RPLNs and tonsils) from 50 animals that were investigated with MN-QuIC™ as well as independently with RT-QuIC, ELISA, and/or IHC . For RPLN testing, MN-QuIC™ correctly identified 18 of 20 CWD positive tissues and 20 of 20 CWD not detected tissues. In tonsil testing, MN-QuIC™ successfully identified all 10 CWD positive and all 10 CWD not detected tissues. For the blinded field test outside of the laboratory, we detected 3 CWD positive animals and 10 CWD negative animals, in which independent ELISA results matched MN-QuIC™ with 100% consistency. All MN-QuIC™ results for both laboratory and field testing were obtained in approximately 24hrs.

**Conclusions**: We successfully demonstrated that MN-QuIC™ is functional in a non-traditional laboratory setting by correctly identifying all CWD positive and CWD not detected (independently confirmed with ELISA and/or IHC tests) animals in a blinded study at a field site, thus documenting the portability of the assay. We conclude that hybrid AuNP and QuIC assays, such as MN-QuIC™, have great potential for sensitive, field-deployable diagnostics of CWD.

**Funded by**: Minnesota Department of Natural Resources; the Minnesota State Legislature through the Minnesota Legislative-Citizen Commission on Minnesota Resources (LCCMR); Minnesota Agricultural Experiment Station Rapid Agricultural Response Fund; Minnesota Agricultural, Research, Education, Extension and Technology Transfer (AGREETT) program; CSE Interdisciplinary Fellowship

**Acknowledgement**: We thank Christopher Ertsgaard and Dong Jun Lee for helpful discussions on experimental results and protocols. Portions of this work were conducted in the Minnesota Nano Center, which is supported by the National Science Foundation through the National Nano Coordinated Infrastructure Network (NNCI) under Award Number ECCS-2025124. W. Zhang provided the expertise for TEM studies. These studies were carried out in the University of Minnesota Characterization Facility, which receives partial support from the NSF through the MRSEC (Award Number DMR-2011401) and the NNCI (Award Number ECCS-2025124) programs. F. Schendel, T. Douville, and staff of the University of Minnesota Biotechnology Resource Center provided critical support concerning the large-scale production of recombinant proteins. We thank the Minnesota Department of Natural Resources, especially M. Carstensen, Lou Cornicelli, E. Hildebrand, P. Hagen, and K. LaSharr, for providing the white-tailed deer tissues used for our analyses and logistical assistance for MN-QuIC field deployment. K. Wilson of the Colorado State University Veterinary Diagnostic Laboratory provided assistance with ELISA and IHC testing of samples reported herein. S. Stone provided valuable logistical assistance with our molecular work. We thank NIH Rocky Mountain Labs, especially B. Caughey, A. Hughson, and C. Orru for training and assistance with the implementation of RT-QuIC and for supplying the rPrP clone.

**Isolation and Characterization of Natural Bioactive Polyphenols with Antioxidant and Anti-Prion Properties**

Nikoletta Christoudia^a^, Eirini Kanata^b^, Korina. Karagianni^a^, Spyros Pettas^a^, Matthias Schmitz^c^, Andreana N Assimopoulou^d^, Konstantinos Xanthopoulos^b^, Dimitra Dafou^a^, and Theodoros Sklaviadis^b^

^a^Department of Genetics, Development and Molecular Biology, School of Biology, Aristotle University of Thessaloniki, Thessaloniki, Greece; ^b^Laboratory of Pharmacology, School of Pharmacy, Aristotle University of Thessaloniki, Thessaloniki, Greece; ^c^Department of Neurology, University Medical Center and the German Center for Neurodegenerative Diseases (DZNE), Göttingen, Germany; ^d^Laboratory of Organic Chemistry, School of Chemical Engineering, Aristotle University of Thessaloniki, Thessaloniki, Greece

**Aims**: Prion diseases, also known as transmissible spongiform encephalopathies (TSEs), are a group of rare, fatal brain diseases that affect both animals and humans – and are caused by the misfolding of normal protein (PrP^C^) into disease-associated protein (PrP^Sc^). Oxidative stress has been found to be associated with the onset and/or progression of neurodegenerative diseases. Natural polyphenol bioactive compounds act as inhibitors of oxidative stress. We aimed to study the neuroprotective biological activities of natural polyphenols.

**Material and Methods**: Carnosic acid (a catechol-type diterpene isolated from *Rosmarinus officinalis*), Carnosol (Carnosic acid’s metabolite), Oenin and Myrtillin chloride (Anthocyanins) and anthocyanin extracts isolated from grape skins using methods such us HPLC-DAD-MS, GC-MS and NMR, were tested for their antioxidant and anti-prion effects.

The assessment of bioactivity of polyphenols was evaluated in an *in vitro* model of Prion disorders (N2a22L cells) and a cell-free Prion amplification assay (RT-QuIC). Cell viability in the presence of different concentrations of polyphenols extracts was estimated by MTT assay. In addition, the assessment of their neuro- and anti-prion protection was evaluated on the N2a22L cell model by determining, via western-blot and RT-QuIC, whether they inhibit the accumulation of the proteinase–resistant protein PrP^Sc^. Their antioxidant role was estimated by the expression measurement of genes linked with the antioxidant response, using real-time PCR and by the measurement of reactive oxygen species (ROS) production.

**Results**: In line with polyphenols’ anti-oxidant properties, the expression of genes linked with anti-oxidant response was elevated when N2a22L cells were treated. In addition, polyphenols demonstrated their effectiveness to neutralize oxidative stress, by decreasing ROS. Treatment of N2a22L cells resulted in a remarkable reduction in the accumulation of the disease-associated form of PrP^Sc^, as detected by immunoblotting. This effect was validated in cell-free assays, demonstrating that polyphenols can independently prevent the formation of PrP^Sc^. Importantly, cell-free assays unveiled that these natural products not only prevent the formation of PrP aggregates but can also disrupt already formed aggregates.

**Conclusions**: Our findings suggest that polyphenols have pleiotropic effects against Prion diseases, suggesting that they could become important preventative and/or therapeutic agents against Prion and other neurodegenerative diseases.

**Funded by**: European Regional Development Fund, 2021–2023. Investment Research Plans for Business Research and Development of Central Macedonia (KMP6-0079465).

**Grant number**: KMP6-0079465

**Longitudinal Profile of Specific Blood Cell Phenotypes Critical to Prionemia in Deer Inoculated with Chronic Wasting Disease**

Brianne M. Coleman, Amy V. Nalls, Erin E. McNulty, Joseph A. Westrich, Audrey M. Sandoval, and Candace K. Mathaison

Department of Microbiology, Immunology, and Pathology, College of Veterinary Medicine and Biomedical Sciences, Colorado State University, Fort Collins, Colorado, USA

**Aims**: Chronic wasting disease (CWD) is a terminal, infectious prion disease endemic within captive and free-ranging cervid populations across North America, Scandinavia, and Korea. It has been established that prion infectivity is present in the blood of prion-infected animals, including deer and humans. Yet to be revealed is the longitudinal profile of specific cell subsets associated with prion infection. Using our white-tailed deer model we have established a reliable, consistent method for isolating blood cell populations throughout disease course; from minutes post inoculation to terminal disease. Throughout CWD disease course we isolate platelets, polymorphonuclear cells (PMNs), total peripheral blood mononuclear cells (PBMCs), and specific blood cell subsets including CD4, CD8, CD14, and B cells. All of these cell subsets are being analyzed for the presence of amyloid seeding activity (prions) by real-time quaking-induced conversion (RT-QuIC) assay.

**Material and Methods**: We employ density gradient and centrifugation processing on fresh whole blood from a cohort of four deer inoculated with 10 mg of prion infected brain material, to isolate cell populations of interest followed by magnetic bead separation for specific blood cell subset extraction. Verification of each cell population is done by flow cytometry utilizing the limited, species-specific antibodies available for cervids. Isolated cell fractions harvested at various times throughout disease course are analyzed for protein seeding activity by our modified amyloid amplification assay, lipase iron-oxide bead extraction RT-QuIC (LIQ). Two animals inoculated with 10 mg of non-infectious prion brain material are processed in the same manner to serve as negative controls.

**Results**: Prion seeding activity has been identified in PBMC and subset populations at or near the time of lymphoid biopsy positivity, with a progressive increase in signal detection as disease course progresses. CD14 and B cell populations express consistently increasing rates of prion amplification with indications of oscillating T cell interactions over time. Evaluation and optimization of prion detection in platelet and PMN populations by LIQ and protein misfolding cyclic amplification assay with RT-QuIC readout (PQ) is currently ongoing.

**Conclusions**: Our studies are revealing the temporal prion seeding activity in bulk PBMC and specific blood cell subsets across the longitudinal course of CWD infection. These findings will provide better understanding of mechanisms associated with intra-host prion trafficking.

**Funded by**: NIH-NIAID, NIH-NINDS

**Grant number**: 2R01AI112956-06, 1R01NS107246-01

**Exposure of non-human primates to low doses of BSE/vCJD prions: an update**

Emmanuel Comoy, Jacqueline Mikol, Jérôme Delmotte, and Jean-Philippe Deslys

Direction of Fundamental Research, Division of Prions and Related Diseases (SEPIA), CEA, Fontenay-aux-Roses, France

**Aims**: The occurrence of a high prevalence of healthy carriers (1/2,000) in UK, as revealed by appendix studies, constitutes a sharp contrast with the limited number of clinical cases of variant Creutzfeldt-Jakob disease (vCJD) and the absence of new cases during the past years.

The high heterogeneity of consumers’ exposure may explain this apparent paradox: a low number of people were exposed to a high amount of infectivity, whereas a high number of people were exposed to a very low amount of infectivity. Our macaque model might help to assess the clinical evolution of these latter ones and their potential as a source of secondary exposure, notably through blood donations.

**Material and Methods**: We exposed cynomolgus macaques to serial dilutions of BSE-infected material or blood products from different sources. Post mortem histological and biochemical analyses were performed on clinically-affected animals.

**Results**: High dose-inoculated animals developed typical clinical vCJD disease with all the pathognomonic hallmarks after incubation periods ranging from 3 to 8 years. Some low-dosed animals developed clinical signs with atypical patterns after extensive incubation periods, exhibiting lesion and biochemical profiles that differed markedly from the typical disease. Despite the presence of neurological signs and neuronal lesions, classical lesions of spongiform change and presence of cerebral PrPres were inconstant, or even absent, whereas prion infectivity was evidenced after successive transmissions.

**Conclusions**: These observations suggest that low-dose exposure, which would have been the most frequent occurrence during the period of risk and would correspond to healthy carriers, could induce non-typical pathologies that may not be recognized as ‘prion disease’.

**Funded by**: European Commission, French Research Funding Agency, Health Canada

**Non-human primates: a renewed gold standard for prion(-like) diseases?**

Emmanuel Comoy, Jacqueline Mikol, Jérôme Delmotte, and Jean-Philippe Deslys

Direction of Fundamental Research, Division of Prions and Related Diseases (SEPIA), CEA, Fontenay-aux-Roses, France

During decades non-human primates (NHP) were considered as gold standard to model human prion diseases until the onset of humanized transgenic mice. The NIH group of Carleton Gajdusek first brought from these models pivotal and founding information about transmissibility, pathogeny and resistance of the different forms of human prion diseases (familial, sporadic or iatrogenic CJD, Kuru …), and in a second time, our primate studies provided the first experimental evidence for a zoonotic potential of BSE.

The BSE crisis opened the field to studies about the zoonotic potential of the other animal prion diseases and the iatrogenic (mainly transfusional) risk of subsequent human prion diseases. Transgenic models of mice expressing human PrP emerged at that time and were widely used to assess these questions, with respect to their numerous advantages (expression of the prion protein of concern, little size, limited cost, ethical considerations, availability of dedicated facilities) in comparison to primates. However, primate (mainly macaque) models persisted since they provided in these two domains complementary answers according to their specific features: their lifespan (25–30 years versus 2 years for mice) is more compatible with long incubation periods (as expected) in humans, and most of all their size, their phylogeny and their physiology make them unique to model the potential natural routes of human contamination (oral, intravenous, accidental).

During the last decade, we and others described, in different independent primate studies, unexpected observations after prion exposure in several non-optimal conditions, mostly through peripheral routes. The animals developed neurological diseases with either incomplete prion phenotypes, or pathological pictures that would not be suspected as linked to prion. These observations that will be updated here question the real expanse of prion diseases and our capacities do detect them. At a time where structural, conjectural and ethical issues hamper the use of NHP in all the areas of scientific research, these observations together with new technical approaches for refined animal monitoring, renew the interest of these large animal models for prion diseases but also for prion-like diseases, with the first description in a non transgenic model of the transmissibility of Alzheimer’s disease.

**Optimization of the RT-QuIC in prion disease diagnostics**

S. Correia, M. Schmitz, and I. Zerr

Neurologie, University Medical Center, Göttingen, Germany

**Aims**: Real-time quaking-induced conversion (RT-QuIC) is a highly sensitive and specific diagnostic test for Creutzfeldt-Jakob disease (CJD). The current sensitivity of CSF RT-QuIC was undertaken at the Germany National Reference Center for TSE is 80–89% and the specificity is 99–100%. We aimed to increase the sensitivity in CSF RT-QuIC. Based on this aim, the following tasks were formulated:

1. Purification of Hamster-sheep, FL Human, FL Human E200K, and FL Human FFI recombinant PrP substrates were performed. They were tested in a set of samples to define assay conditions for reduced self-aggregation.
2. The best suitable substrate for sCJD and gCJD CSF diagnostic via RT-QuIC was investigated to obtain the best diagnostic accuracy. sCJD, gCJD, and control samples were tested. We compared the signal response from each substrate and the best substrate performance was determined.
3. To determine agreement between the different substrates, we performed standard RT-QuIC (Hamster-sheep) in parallel with FL Human and E200K.

**Material and Methods**: Different substrates were purified using AKTÄ system. To determine the sensitivity and specificity of different substrates in sCJD and gCJD we used a cut-off at 10,000 rfu/80 h in 88 sCJD samples, 16 E200K CJD samples and 22 FFI samples. To investigate the agreement between the different substrates in CJD CSF, 134 CSF samples with positive or negative signal response in the standard RT-QuIC (hamster-sheep) were analyzed in parallel with Fl Human and E200K.

**Results**: The E200K subtrate showed the highest sensitivity in sCJD (98%), E200K (100%) and FFI (55%) patients. The specificity in all the substrates was 100 %. Signal-kinetic of the substrates were analyzed and E200K substrate had a significant shorter lag phase and higher AUC in comparison with Hamster-sheep and FL Human substrates. Agreement between substrates was analyzed using the Cohen’s kappa value in a cohort of 134 CSF samples. We obtained an agreement of 86.6% between Hamster-sheep and FL Human and a agreement of 79.1% between Hamster-sheep and E200K substrates. For FL Human and E200K substrates we obtained 71.6% agreement.

**Conclusions**: The results suggest that E200K substrate increases the sensitivity of the RT-QuIC in patients with sCJD to 98% (Hamster-sheep 80% and FL Human 75%) and in CJD E200K and GSS patients to 100% (Hamster-sheep 88% and 50%, FL Human 88% and 50%). In FFI samples the E200K substrate showed the best sensitivity (55%) in comparison to FL Human (36% sensitivity) and Hamster-sheep (36% sensitivity).

**A non-radioactive cell-free assay for detection of direct PERK activators**

Márcia Costa^a,b,c^, Thomas W. Rösler^a,b^, and Günter U. Höglinger^a,b,c^

^a^Department of Translational Neurodegeneration, German Center for Neurodegenerative Diseases, Munich, Germany; ^b^Department of Neurology, School of Medicine, Technical University of Munich, Munich, Germany; ^c^Department of Neurology, Hannover Medical School, Hannover, Germany

**Aims**: Activation of the PERK pathway, a major branch of the unfolded protein response has shown to be promising therapeutic approach in tauopathies and possibly other neurodegenerative proteinopathies. In the present study, we aimed to develop a cell-free assay for the discovery of novel direct PERK activators.

**Material and Methods**: We measured the activity of human recombinant PERK in the presence of a test compounds (activators or inhibitors) by detecting the amount of consumed ATP in a cell-free setup. We developed a novel assay protocol and carried out cell-free protein reactions to optimize the assay parameters.

**Results**: We were able to determine suitable assay conditions, including parameters such as optimal PERK concentration, reaction temperature, reaction time and type of substrate protein. We successfully detected PERK activation and inhibition by selected modulators, using SMAD3 as a phosphorylation-accepting protein. The developed assay showed adequate stability and robustness to estimate an activating EC_50._ We also provided novel insights into the mechanism of PERK activation by a distinct activator. Lastly, we assessed the assay applicability by testing MK-28, a recently described PERK activator.

**Conclusions**: Our data shows that the developed cell-free assay can be applied for a high-throughput screening setup to discover novel direct PERK activators from large compound libraries. Novel direct activators of PERK will be useful to deepen the understanding of the PERK signaling pathway particularly in neurodegeneration, and may lead to the identification of new therapeutic drug candidates.

**Funded by/Grant number**: Deutsche Forschungsgemeinschaft under Germany’s Excellence Strategy within the framework of Hannover Cluster RESIST (EXC 2155 – project number 39,087,428)

Niedersächsisches Ministerium für Wissenschaft und Kunst (MWK, ZN3440.TP): REBIRTH – Forschungszentrum für translationale regenerative Medizin

VolkswagenStiftung (Niedersächsisches Vorab)

Petermax-Müller Foundation (Etiology and Therapy of Synucleinopathies and Tauopathies)

**Acknowledgement**: The authors thank Luciana Fernandes for helping with the preliminary setup experiments

**Assessing the effect of inoculation route on pathogenesis in CWD-susceptible gene targeted mice**

Joseph DeFranco, Sehun Kim, Jenna Crowell, Jifeng Bian, Bailey Huser, EmmaKate Raisley, and Glenn C. Telling

Prion Research Center (PRC), the Department of Microbiology, Immunology and Pathology, Colorado State University, Fort Collins, Colorado, USA, Program in Cell and Molecular Biology, Colorado State University, Fort Collins, Colorado, USA

**Aims**: This study aims to explore the effects of inoculation route on disease outcomes following infection of gene targeted (Gt) mice expressing either deer or elk PrP with North American CWD prions. In doing so, we will explore the hypothesis that accurately controlled physiological expression of cervid PrP (CerPrP) by *Prnp* transcriptional elements in Gt mice provides an authentic model in which to study how adaptation, evolution, and selection of CWD strains in non-CNS compartments influences CWD pathogenesis.

**Material and Methods**: Whereas North American deer or moose PrP encodes glutamine at residue 226 (CerPrP-Q226), North American elk PrP encodes glutamate (CerPrP-E226). To precisely assess the effects of this difference on CWD pathogenesis, we created Gt mice in which the murine PrP coding sequence was targeted and replaced with CerPrP-Q226 or CerPrP-E226, referred to as GtQ and GtE mice. This study builds on our preliminary findings that GtQ and GtE mice accurately recapitulate deer and elk CWD including peripheral pathogenesis. Here, we assessed the development of disease in GtQ and GtE following various routes of inoculation. We compared the transmission properties of elk CWD with deer CWD. CWD prions were delivered by various inoculation routes including intracerebrally, intraperitoneally, by gavage, perorally by feeding prion contaminated food, intranasally, intravenously, intramuscularly, and subcutaneously. Iterative passages were also performed. Primary outcome measures included kinetics of disease onset and neuropathological assessments. We assessed PrP^Sc^accumulation in various tissues by standard methods using various mAbs, IHC, and assessed the conformational properties of prions in different tissues by conformational stability analysis. We are assessing CWD prion titers by cell based cervid prion titration (CPCA). We also assessed the transmission properties by peripheral routes of emergent Nordic CWD prion strains that are either lymphotropic or non-lymphotropic.

**Results**: Different inoculation routes produced different transmission efficiencies and mean incubation times to disease. Disease outcomes were influenced by host polymorphism at residue 226 and the properties of the inoculum. All peripheral routes of inoculation had substantially lower levels of PrPSc in the CNS than intracerebrally inoculated mice. In peripheral challenges using Norwegian CWD strains, mice inoculated with lymphotropic strains developed disease, while the mice inoculated with non-lymphotropic strains did not.

**Conclusions**: Our findings illustrate that different routes of CWD inoculation produce various disease outcomes in Gt mice. Our future studies will address the postulate that different routes of inoculation propagate different prion strains that alter pathogenesis and produce unique infectious conformers.

**Funded by**: The National Institutes of Health (NIH) and the Anschutz Foundation (AF)

**Grant number**: NIH grants R01NS121682 and PO1-0011877A and AF grant 6,476,430

**Effects of Montmorillonite Clay Adsorption on Chronic Wasting Disease Prion Seeding Activity and Infectivity in Deer**

Nathaniel D. Denkers^a^, Shannon Bartelt-Hunt^b^, Jason C. Bartz^c^, Candace K. Mathiason^a^, and Edward A. Hoover^a^

^a^Prion Research Center, College of Veterinary Medicine and Biological Sciences, Department of Microbiology, Immunology, and Pathology; Colorado State University, Fort Collins, CO, USA; ^b^Department of Civil Engineering, University of Nebraska-Lincoln, Omaha, Nebraska, USA; ^c^Department of Medical Microbiology and immunology, Creighton University, Omaha, Nebraska, USA

**Aims**: Chronic wasting disease (CWD) is unique in its facile spread in nature, attributable to indirect transmission from environmental exposure. Infected cervids shed prions through excreta/bodily fluids into the landscape–potentially generating a substantial prion reservoir. Prions bound to soil may have altered infectivity, nevertheless, the impact of environmental contamination remains unclear. These studies examined: (1) the binding capacity of CWD prions to montmorillonite (MTE), as assessed by real-time quaking-induced conversion (RT-QuIC), and (2) the effect of CWD prions, unbound or bound to MTE, when orally inoculated into white-tailed deer.

**Material and Methods**: For *in vitro* experiments, concentrations of montmorillonite (3.12–25 mg) were incubated with serial dilutions (10^−5^– 10^−7^) of CWD-positive or negative brain homogenates, whereafter bound and unbound fractions were analyzed by RT-QuIC. For *in vivo* studies, eight (8) white-tailed deer were inoculated with 10 mg CWD-positive brain, either unbound (n = 4) or bound (n = 4) to 5 grams of MTE. Serial longitudinal tissue biopsies and excreta have been collected every 3 months post-inoculation (MPI) and are being analyzed for CWD prion seeding activity and PrP^CWD^by RT-QuIC and immunohistochemistry. Deer are also being monitored for development of clinical disease.

**Results**: *In vitro* results demonstrated that MTE binds CWD prions and removes 90–100% prion seeding activity in a concentration-dependent manner. Prion seeding activity was detectable even when bound to MTE. From these studies, we estimated prion binding capacity to be ~100-200ng CWD brain per milligram of MTE. *In vivo* results from deer inoculated with prions either unbound or bound to MTE demonstrated positive seeding activity in tonsil biopsies by RT-QuIC from 2 of 4 deer in each cohort at 3 MPI. By 6 MPI, 3 deer in the MTE cohort and 2 in the unbound cohort were positive by both RT-QuIC and IHC. At 9 MPI, 3 deer in each cohort were positive by both assays. All 4 animals in each cohort became CWD-positive and have remained so at the time of this writing (15MPI). None have signs of clinical disease.

**Conclusions**: These studies demonstrate: (1) MTE efficiently binds CWD prions and that bound prions maintained seeding activity, inferring a potential durable environmental reservoir and vehicle for horizontal transmission; and (2) While MTE binding removed CWD prion seeding activity *in vitro, in vivo* assessment in deer has not demonstrated a discernible difference in infectivity after 15 months. Animals will be observed until clinical disease onset and terminal tissue prion infectivity assayed.

**Funded by**: National Institutes of Health (NIH)

**Grant number**: RO1-NS061902-09 R to EAH, PO1-AI077774 to EAH, and R01-AI112956-06 to CKM

**Acknowledgement**: We abundantly thank Sallie Dahmes at WASCO and David Osborn and Gino D’Angelo at the University of Georgia Warnell School of Forestry and Natural Resources for their long-standing support of this work through provision of the hand-raised, CWD-free, white-tailed deer used in these studies.

**Bioassay of Chronic Wasting Disease Prions Derived from Brain and Lymph Node in White-tailed Deer**

Nathaniel D. Denkers, Caitlyn N. Kraft, Lindsay E. Parrie, Erin E. McNulty, Amy V. Nalls, Candace K. Mathiason, and Edward A. Hoover

Prion Research Center, Department of Microbiology, Immunology and Pathology, College of Veterinary Medicine and Biomedical Sciences, Colorado State University, Fort Collins, Colorado, USA

**Aims**: Chronic wasting disease (CWD) is the most transmissible prion disease and the only one to affect a free-ranging population. Evidence is accruing that prions produced in the lymphoreticular system (LRS) may differ from those produced in the central nervous system (CNS). Studies of transmissible spongiform encephalopathy (TSE) strains in hamsters have illustrated that prion stain conformations differ. CWD is transmitted predominantly by shed rather than CNS prions, and thereby could vary in their conformation and/or tissue tropism. In attempt to further elucidate the transmission and pathogenesis of CWD, we orally exposed white-tailed deer to equivalent low doses of prions present in either brain or retropharyngeal lymph node to determine whether the differences may exist in infection efficiency, tissue tropism, conformation, or clinical disease.

**Material and Methods**: Eight (8) white-tailed deer were orally inoculated with 0.5 mg (equated by reaction rates from real-time quaking induced conversion (RT-QuIC)) of either CWD-positive obex (n = 4) or retropharyngeal lymph node (RPLN) (n = 4) originating from a single source animal. Longitudinal tissue biopsies and excreta were collected every 3 months post-inoculation (MPI) with brain, peripheral lymph nodes, and other tissues and body fluids collected at necropsy (12 MPI). All samples were analyzed by RT-QuIC and immunohistochemistry.

**Results**: Longitudinal tonsil biopsies revealed CWD positivity by RT-QuIC at 3 MPI in 1 of 4 deer (25%) in both cohorts. At six months, 4 of 4 (100%) deer in the obex cohort and 3 of 4 (75%) deer in the RPLN cohort were positive by both assays. By 9 months, the last deer in the RPLN cohort was tonsil biopsy positive by RT-QuIC only. Terminal tissue samples analyzed by RT-QuIC revealed widespread distribution of prions in 8 peripheral lymph nodes of all (100%) obex and 3 of 4 (75%) RPLN inoculated deer. Positivity was demonstrated in the obex of 2 of 4 (50%) deer in each cohort. Excreta results demonstrated 1 of 16 (6%) saliva samples were positive in each cohort with only 1 urine sample positive in the obex cohort. Negative controls remained negative in all samples by both assays.

**Conclusions**: Negligible differences were observed between cohorts infected with comparable doses of CWD prions derived from brain and lymph node over the duration of the study, inferring no discernable variance in CWD pathogenesis from either inoculum source. Ongoing studies are investigating the biochemical differences within the brain and lymph node of the point-source inoculum and matched terminal tissues of inoculated deer.

**Funded by**: National Institutes of Health (NIH)

**Grant number**: RO1-NS061902-09 R to EAH, PO1-AI077774 to EAH, and R01-AI112956-06 to CKM

**Acknowledgement**: We abundantly thank Sallie Dahmes at WASCO and David Osborn and Gino D’Angelo at the University of Georgia Warnell School of Forestry and Natural Resources for their long-standing support of this work through provision of the hand-raised, CWD-free, white-tailed deer used in these studies.

**Shedding of Chronic Wasting Disease Prions in Multiple Excreta Throughout Disease Course in White-tailed Deer**

Nathaniel D. Denkers^a^, Erin E. McNulty^a^, Caitlyn N. Kraft^a^, Amy V. Nalls^a^, Joseph A. Westrich^a^, Wilfred Goldmann^b^, Candace K. Mathiason^a^, and Edward A. Hoover^a^

^a^Prion Research Center, College of Veterinary Medicine and Biological Sciences, Department of Microbiology, Immunology, and Pathology; Colorado State University, Fort Collins, CO, USA; ^b^Division of Infection and Immunity, The Roslin Institute and the Royal Dick School of Veterinary Studies, University of Edinburgh, Midlothian, UK

**Aims**: Chronic wasting disease (CWD) now infects cervids in South Korea, North America, and Scandinavia. CWD is unique in its efficient transmission and shedding of prions in body fluids throughout long course infections. Questions remain as to the magnitude of shedding and the route of prion acquisition. As CWD continues to expand, the need to better understand these facets of disease becomes more pertinent. The purpose of the studies described was to define the longitudinal shedding profile of CWD prions in urine, saliva, and feces throughout the course of infection in white-tailed deer.

**Material and Methods**: Twelve (12) white-tailed deer were inoculated with either 1 mg or 300ng of CWD. Urine, saliva, and feces were collected every 3-month post-inoculation (MPI) throughout the study duration. Cohorts were established based on PNRP genotype: codon 96 GG (n = 6) and alternate codons 96 GS (n = 5) & 103NT (n = 1). Urine and saliva were analyzed using iron-oxide magnetic extraction (IOME) and real-time quaking induced conversion (RT-QuIC)(IQ). Feces were subjected to IOME, followed by 4 rounds protein misfolding cyclic amplification (PMCA) with products analyzed by RT-QuIC (IPQ). To determine whether IPQ may be superior to IQ, a subset of urine and saliva were also tested by IPQ. Results were compared with clinical disease status.

**Results**: Within the 96 GG cohort, positive seeding activity was detected in feces from all deer (100%), in saliva from 5 of 6 (83%), and in urine from 4 of 6 (66%). Shedding in all excreta occurred at, or just after, the first positive tonsil biopsy result. In the 96 GS/103NT cohort, positive seeding activity could be detected in feces from 3 of 6 (50%) deer, saliva in 2 of 6 (33%), and urine in 1 of 6 (16%). Shedding in excreta was detected >5 months after the first tonsil positive result. Four of six 96 GG deer developed clinical signs of CWD, whereas only 2 of the 96 GS/103NT did. Shedding was more frequently detected in deer with clinical disease. The IPQ protocol did not significantly improve detection in saliva or urine samples, however, it significantly augmented detection in feces by eliminating non-specific background commonly experienced with IQ. Negative control samples remained negative in samples tested.

**Conclusions**: These studies demonstrate: (a) CWD prion excretion occurs throughout infection; (2) PRNP genotype (GG≫GS/NT) influences the excreta shedding; and (3) detection sensitivity in excreta can vary with different RT-QuIC protocols. These results provide a more complete perspective of prion shedding in deer during the course of CWD infection.

**Funded by**: National Institutes of Health (NIH)

**Grant number**: RO1-NS061902-09 R to EAH, PO1-AI077774 to EAH, and R01-AI112956-06 to CKM

**Acknowledgement**: We abundantly thank Sallie Dahmes at WASCO and David Osborn and Gino D’Angelo at the University of Georgia Warnell School of Forestry and Natural Resources for their long-standing support of this work through provision of the hand-raised, CWD-free, white-tailed deer used in these studies

**Study of sporadic Creutzfeldt-Jakob disease mortality in France between 1992 and 2016 using an Age-Period-Cohort model**

A. Denouel^a^, J-P. Brandel^a,b^, A. Elbaz^c†^, and S. Haik^a,b†^

^a^CNRS UMR 7225, INSERM U1127, Institut du cerveau et de la moelle épinière, Sorbonne Universités, Paris, France; ^b^AP-HP, Centre National de Référence des Maladies de Creutzfeldt-Jakob, Groupe Hospitalier Pitié-Salpêtrière, Paris, France; ^c^INSERM U1018, CESP, Equipe ‘Exposome and heredity’, Villejuif, France ^†^These authors share senior authorship.

**Aims**: Sporadic form of Creutzfeldt-Jakob disease (sCJD) is the most common form of prion diseases. Its origin is still unknown and the role of exogenous factors remains possible. We aimed to study sCJD mortality from data collected over 25-years of active surveillance in France in order to better understand the origin of this form.

**Material and Methods**: Cases reported to the French CJD Surveillance Network between 1992 and 2016 with a diagnosis of probable or definite sCJD were included to the study. Crude and age- and sex-standardized mortality rates were calculated as well as male-to-female mortality ratio. Variations over time of sCJD mortality were analyzed using an Age-Period-Cohort (APC) model. Age effect reflects a biological process linked to aging, effect of period of death shows an overall trend such as diagnosis evolution, and a cohort of birth effect reflects an environmental exposure change.

**Results**: We included 2475 cases of probable or definite sCJD aged 45–89 years. The overall mortality rate was 4.58 per million person-years with a peak in 75–79 years age group. The APC model showed effects of age, period and cohort on sCJD mortality. We also observed an age-dependent sex effect. Men-to-women mortality ratio was <1 before 55 years and >1 after 80 years.

**Conclusions**: Study of sCJD mortality rates revealed that several factors influence its evolution over time. Indeed, APC analyses highlighted processes linked to aging through an age effect, improvement of surveillance system especially during first years through the period effect, and unexpectedly, showed a cohort effect in favour of unknown environmental risk factors (zoonoses, diet, pesticides, antibiotics?). In addition, an age-dependent gender effect was shown with a shift in men-to-women mortality ratio at the age peak.

This statistical approach based on 25 years of surveillance in France brings results that support the role of environmental factors that could influence sCJD susceptibility.

**Evaluation of naturally occurring polymorphic variants of the PrP from cervids as RT- QuIC substrates for the detection of multiple CWD strains**

Carlos M. Díaz-Domínguez^a^, Nuno Gonçalves-Anjo^a,b^, Jorge M. Charco^a,b,d^, Tram Thu Vuong^e^, Leire Fernández-Veiga^a^, Linh Tran^e^, Cristina Sampedro-Torres-Quevedo^a^, Estela Bastos^b^, Hasier Eraña^a,c,d^, Sylvie L. Benestad^e^, and Joaquín Castilla^a,d,f^

**^a^**Center for Cooperative Research in Biosciences (CIC bioGUNE), Basque Research and Technology Alliance (BRTA), Bizkaia Technology Park, Derio, Spain; **^b^**Centre for the Research and Technology of Agro‐Environmental and Biological Sciences (CITAB), University of Trás‐os‐Montes and Alto Douro (UTAD), Vila Real, Portugal; **^c^**ATLAS Molecular Pharma S. L. Bizkaia Technology Park, 800, Derio, Spain; **^d^**Carlos III National Health Institute, Madrid, Spain; **^e^**Norwegian Veterinary Institute, Norway; **^f^**IKERBASQUE, Basque Foundation for Science, Prion Research Lab, Bilbao, Spain Centro de Investigación Biomédica en Red de Enfermedades Infecciosas (CIBERINFEC)

**Aims**: Due to the emergence of new Chronic Wasting Disease strains in different cervid species in Europe and the large amount of polymorphic variants being detected worldwide, which could give rise to other strains, refining CWD surveillance methods is vital. Here, we propose to improve RT-QuIC detection of North American and European CWD strains by testing all known polymorphic variants of the PrP within the *Cervidae* family as substrate.

**Material and Methods**: For that purpose, we have generated plasmids containing every polymorphic variant of the PrP (from the amino acids 90 to 230) within the *Cervidae* family that were produced in bacteria, purified and used to generate new RT-QuIC substrates. Bank vole 109I rec-PrP, hamster rec-PrP, and hamster-sheep chimeric rec-PrP were selected as reference RT-QuIC substrates.

Brain homogenates from a variety of CWD-infected cervids that presented different strain and/or genetic properties were selected as seed to challenge all the previous substrates. Red deer normal brain homogenate was used as a negative control.

**Results**: All tested substrates challenged with serially diluted CWD seeds were ranked according to their time to threshold, obtaining a value for each inoculum-substrate combination. These revealed the influence of every polymoprhic variant of the cervid PrP in the propagation and detection of distinct strains, and allowed to highlight the regions of the protein where the most relevant polymophisms are. Furthermore, we assessed the more promising polymorphic variants for the detection of each different CWD strain by RT-QuIC after equilizing the concentration of PrP^Sc^in every inoculum tested.

**Conclusions:**

- Some polymorphic variants of the cervid rec-PrP can be considered as better substrates for CWD detection by RT-QuIC than the reference substrates for the different strains tested.

- Polymorphic variations located in certain regions of the recombinant cervid PrP alter the CWD detection by RT-QuIC.

**Funded by**: Spanish ministry of science and innovation

**Grant number**: PID2021-122201OB-C21

**Single-cell transcriptomics of mammalian prion diseases**

Athanasios Dimitriadis^a^, Fuquan Zhang^a^, Thomas Murphy^a^, Thomas Trainer^a^, Zane Jaunmuktane^b,c^, Christian Schmidt^a^, Malin Katarina Sandberg^a^, Tamsin Nazari^a^, Jackie Linehan^a^, Stephanie Canning^a^, Azadeh Khalili-Shirazi^a^, Mark Kristiansen^d^, John Collinge^a^, Simon Mead^a^, and Emmanuelle Vire^a^

^a^MRC Prion Unit at University College London (UCL), UCL Institute of Prion Diseases, UCL, London, W1W 7FF, UK; ^b^Division of Neuropathology, National Hospital for Neurology and Neurosurgery, University College London NHS Foundation Trust, London, UK; ^c^Department of Clinical and Movement Neurosciences and Queen Square Brain Bank for Neurological Disorders, Queen Square Institute of Neurology, University College London, London, UK; ^d^UCL Genomics, UCL Great Ormond Street Institute of Child Health and Zayed Centre for Research into Rare Disease in Children, London, UK

**Aims**: Despite substantial research aiming to elucidate prion disease pathogenesis, the underlying mechanisms of cellular toxicity and neurodegeneration remain poorly characterized. The human brain comprises numerous cell populations with heterogenous transcriptional landscape, complicating the interpretation of transcriptomic studies. To untangle this complexity, we performed longitudinal single-cell transcriptomics studies on case-control mouse and human brain. We aimed to transcriptionally characterise mammalian prion diseases and elucidate its mechanisms.

**Materials and Methods**: We utilized a single-nucleus sequencing technique based on split-pool barcoding called SPLiT-seq where random and unique nucleotide barcodes are attached to each transcript through multiple rounds of combinatorial indexing. Our pipeline combined tissue disruption, nuclei isolation, RNA barcoding, library preparation, Illumina sequencing and bioinformatic analysis. Our bioinformatics pipeline is based on Seurat, used published open-source software, and followed the latest guidelines of the community. We applied this experimental pipeline to a time-course case-control study of RML-and control brain homogenate inoculated FVB mice (N = 95, timepoints: 20, 40, 80, 120 dpi and disease end-stage), and a human case-control study in post-mortem and biopsied brain samples (N = 26). We performed standard pathological characterisation of our samples and used RNA SCOPE to visualise transcript expression.

**Results**: We generated 210,000 high-quality cell transcriptomes across 5 time points in mice. After quality control steps and clustering of the dataset based on transcriptomic similarity, we identified 26 subclusters of cortical neurons, interneurons, mature oligodendrocytes, oligodendrocyte precursor cells, vascular and leptomeningeal cells, and astrocytes. Glial activation was evident from 80 dpi, while our data suggested a selective transcriptomic response of individual cell clusters to disease. We identified a pattern of neuronal transcriptomic change shortly after RML-brain inoculation that quickly resolved, despite rapidly increasing prion titres in brain, only to return at later stages when the neuropathology of prion disease was evident. Subsequent pathway analyses identified common perturbed biological pathways associated with synaptic dysfunction and ion homeostasis. Correlations of these findings with infectivity and validated transcriptomic targets using RNA SCOPE will be discussed.

**Conclusions**: Here we provide the first single-cell transcriptomics study of prion diseases in mouse and human brain which found cell-type and time-specific patterns. Taken together, findings suggest that prion replication itself does not produce a transcriptomic signature in brain, rather, a transient pattern of toxicity can be seen immediately following inoculation of prion disease brain homogenate, which becomes re-established as prion disease neuropathology develops. Our human data is being analysed and new findings will be discussed to provide a more complete characterization of the molecular mechanisms of prion diseases.

**Funded by**: Medical Research Council and Alzheimer’s Research UK

**Grant numbers**: ARUK-PPG2020A-030 (ARUK)

**Acknowledgements**: We are very grateful to all patients and family who consented their samples to be used in the study, to the National Prion Clinic for their help acquiring patient’s meta-data, and to the Queen Square Brain Bank and BRAIN UK for providing human control samples. The ‘Genetics, Epigenetics and Bioinformatics’ group is core funded by the MRC Prion Unit at UCL.

**Development of a high-throughput system for the screening of anti-prion molecules**

Katherine Do, Uffaf Khan, Carlos Kramm, Rebeca Benavente, Claudio Soto, Rodrigo Morales

Department of Neurology, McGovern Medical School, University of Texas Health Science Center at Houston, Texas, USA

PrP^Sc^ (a misfolded form of the physiological prion protein, PrP^C^) is the main effector in TSEs. Extensive evidence suggests that preventing or decreasing PrP^Sc^ replication is a promising target for therapeutic intervention. Unfortunately, the existence of different PrP^Sc^strains provide challenges for the identification of anti-prion compounds as molecules modifying a particular prion agent may not be effective on a second one. The search of appropriate anti-prion therapies is limited by the lack of platforms allowing the screening of compound libraries in a high throughput manner. Although cell-based systems have been adapted for such purpose, they are not available for any relevant prion strains. We have shown that the Protein Misfolding Cyclic Amplification (PMCA) technology faithfully replicate the conformational properties of prions, maintaining all relevant biological features such as their infectivity and particular strain properties.

**Aims:** Standardization of a high throughput PMCA (HT-PMCA) platform for rodent prions in a 96-well format and screening of a small library of anti-prion compounds.

**Material and Methods:** We implemented a 96-well plate PMCA system to efficiently replicate six strains of prions from rodents (mice and Syrian hamsters) and reduced the time of analysis by the use of dot blots. We then tested a small library of known protein misfolding inhibitors for all six prion strains.

**Results:** We were able to reduce PMCA times to 24 hours (compared to conventional 48 or 72 hours per PMCA round) and volumes to 50 μL (compared to conventional 100 μL) without losing sensitivity. Dot blots were adapted in replacement of conventional western blotting, fact that substantially reduced the time to visualize PrP^Sc^ signals. Other materials and reagents were also reduced. Regarding the protein misfolding inhibitors, we observed strain-specific patterns of prion misfolding interference with specific compounds. Specifically, our data shows that while some drugs were active for some prion strains, they were inactive in others. Our system showed great reproducibility as judged by replicates run in different plates.

**Conclusions:** Our HT-PMCA system is suitable to test chemical libraries for putative anti-prion molecules. The data presented in this work demonstrate that the anti-aggregation activity of certain compounds is prion-strain specific.

**Funded by:** Creutzfeldt-Jakob disease Foundation

**Phagocytic glia mediate prion-like spreading of mutant huntingtin aggregates in *Drosophila* brains**

Kirby M. Donnelly, Aprem D. A. Zaya, Graham H. Davis, David S. Tomlinson, and Margaret M. Panning Pearce

Department of Biological Sciences, University of the Sciences, Philadelphia, PA

**Aims**: A key pathological feature of neurodegenerative disease is appearance of insoluble aggregates that arise due to protein misfolding in cells of the central nervous system (CNS). The healthy brain has the capacity to clear toxic protein aggregates via multiple mechanisms, including the ubiquitin-proteasome pathway, autophagy, and phagocytic engulfment by glia, but defects in these pathways lead to progressive accumulation of aggregates over time. A growing body of evidence supports the hypothesis that pathogenic aggregates associated with many neurodegenerative diseases behave similarly to infectious prions–they spread from cell-to-cell and nucleate the aggregation of natively-folded versions of the same protein, events which can contribute to aggregate propagation in the brain. Here, we explored the hypothesis that mutant huntingtin (mHTT) aggregates associated with Huntington’s disease (HD) spread in a ‘prion-like’ manner between synaptically-connected neurons and glia in the *Drosophila* CNS.

**Methods and Results**: In a *Drosophila* model of HD, mHTT aggregates formed in presynaptic olfactory receptor neuron (ORN) axons nucleated the aggregation of soluble, cytoplasmic wild-type HTT (wtHTT) proteins expressed in post-synaptic partner projection neurons (PNs) or in nearby phagocytic glia. ORN-to-PN and ORN-to-glia transfer of mHTT aggregates was enhanced when ORN activity was silenced, slowed when caspase-dependent apoptosis in ORNs was blocked, and remarkably, required expression of the conserved glial scavenger receptor Draper/MEGF10. Further, mHTT aggregate transmission between synaptically-connected ORNs and PNs involves a transient visit to the glial cytoplasm, suggesting that phagocytic glia act as obligatory intermediates in trans-synaptic aggregate spreading in vivo. Forward genetic screens have revealed that entry of phagocytosed neuronal mHTT aggregates into the glial cytoplasm involves Rab GTPases with proposed roles in phagosome maturation, suggesting that inefficient or incomplete progression of engulfed material through the phagolysosomal system could generate seeding-competent mHTT aggregate species. In support of this, mHTT aggregate formation in neurons accelerates age-related decline in glial phagocytic clearance of axonal debris and increases markers of late phagosome and lysosomes in the fly CNS.

**Conclusions**: Our findings suggest that while phagocytic glia regulate the load of mHTT aggregates in the brain, they also promote formation of mHTT aggregate seeds capable of spreading the misfolded phenotype to other cells in the brain. Together, these data add to a growing understanding of activated glial cells as double-edged players in neurodegenerative diseases such as HD.

**Funded by**: NIH/NIA, Pittsburgh Foundation, W.W. Smith Charitable Trusts

**Grant number**: R03 AG063295

**Prion infectivity accumulation in CJD patients peripheral tissues and its implication for public health**

Jean-Yves Douet^a^, Alvina Huor^a^, Hervé Cassard^a^, Severine Lugan^a^, Naima Aron^a^, Chloe Mesic^a^, Tomas Barrio^a^, Joaquin Castilla^b^, Hasier Eraña^b^, Delisle Marie-Bernadette^c^, Péran Patrice^c^, Vincent Beringue^d^, James W. Ironside^e^, and Olivier Andreoletti^a^

^a^UMR INRAE ENVT 1225, Toulouse, France; ^b^CIC Biogune, BilBao, Spain; ^c^INSERM U 1214 TONIC, Toulouse, France; ^d^INRAE VIM, Jouy-en-Josas, France; ^e^National Creutzfeldt-Jakob Disease Research & Surveillance Unit, Edinburgh UK

**Aims**: In marked contrast to vCJD, previous investigations in sCJD revealed either inconsistent levels or an absence of PrP^Sc^in peripheral tissues. These findings contributed to the consensus that risks of transmitting sCJD as a consequence of non-CNS invasive clinical procedures were low. Recently, the presence of prion infectivity in the plasma and bone marrow of several sCJD patients was reported. These results raised questions about the overall distribution of prion infectivity and transmission risks associated with other peripheral tissues from sCJD patients.

**Material and Methods**: we systematically assessed the distribution of prion infectivity in the peripheral tissues of sCJD (from different subtypes), vCJD and a specific subtype of gCJD (E200K) patients. Prion quantification/detection was carried out by bioassay (in PrP transgenic mice) and/or by *in vitro* amplification (PMCA).

**Results**: we found that, like in vCJD, prions are largely distributed in the peripheral tissues of sCJD and gCJD-affected patients from various subtypes. Although strong inter-individual heterogeneity exists between sCJD-affected individuals, the overall infectivity distribution and levels observed in sCJD were not significantly different from those found in vCJD patients. These findings naturally raise a question: how early prion infectivity starts accumulating in peripheral organs in sCJD.

To answer this question, we propose that studying certain forms of genetic CJD could represent an interesting approach. Transmission experiments using CJD cases associated with E200K mutation demonstrated that these form of gCJD are caused by the same strains than sCJD. They also indicated that both E200K gCJD and sCJD-affected patients display the same infectivity distribution patterns in peripheral tissues.

**Conclusions**: These results could impact on our perception of the possible transmission risks associated with sCJD. They also support the contention that E200K carriers could allow major advances in the understanding of CJD disease pathogenesis.

**Funded by**: EU FEDER/INTERREG

**Grant number**: EFA282/13 TRANSPRION, EFA148/16 REDPRION

**Acknowledgement**: All contributors and funders

**Peripheral prion accumulation in CWD-infected animals**

Camilo Duque Velasquez^a,c^, Alicia Otero^d^, Chiye Kim^a,c^, Judd Aiken^b,c^, and Debbie McKenzie^a,c^

^a^Department of Biological Science, University of Alberta, Edmonton, AB T6G 2G8, Canada; ^b^Department of Agriculture, Food & Nutritional Science, University of Alberta, Edmonton, AB T6G 2G8, Canada; ^c^Centre for Prions and Protein Folding Diseases, University of Alberta, Edmonton, AB T6G 2M8, Canada; ^d^Centro de Encefalopatias y Enfermedades Transmisibles Emergentes, University of Zaragoza, Zaragoza, Spain

**Aims**: The main host factor affecting the likelihood of cervids of becoming infected and developing chronic wasting disease (CWD) is the primary structure of the cellular prion protein (PrP^C^). Single changes in the amino acid composition of PrP^C^(i.e., polymorphisms) can affect misfolding into PrP^C^conformers. The serine polymorphism at amino acid 96 (S96) of white-tailed deer PrP^C^slows the progression of CWD in deer. In transgenic mice, S96-PrP^C^ protects against prion disease following oral, intraperitoneal and intracranial infection with different CWD strains. In PMCA assays, using S96-PrP^C^ as substrate for different CWD strains, Wisc-1 and CWD2 (the most common CWD strains) lose their replication capacity. We will present data on the seeding activity of PrP^C^prions from peripheral tissues of deer expressing different *PRNP* genotypes.

**Material and Methods**: PMCA is being used to estimate seeding activity present in peripheral tissues from terminally-ill orally inoculated white tailed deer expressing different *PRNP* genotypes.

**Results**: Preliminary analysis of various peripheral tissues (muscle, heart, kidney pancreas, salivary gland) from deer demonstrated that PrP^C^polymorphisms correlate with lower prion seeding activity as determined by the number of serial PMCA rounds required for detection. In addition, seeding of PMCA substrates with tissue homogenates from deer heterozygous for *PRNP* polymorphisms resulted in co-amplification of different PrPres glycotypes.

**Conclusions**: Deer expressing PrP^C^polymorphisms accumulate less prions as compared to deer expressing the *PRNP* genotype (wt/wt; G96/G96) most commonly associated with CWD infection and rapid disease progression. Understanding the dynamics of prion accumulation in hosts with PrP^C^molecules impairing prion propagation would provide information fundamental for developing strategies to reduce the spread of CWD.

**Funded by**: Alberta Prion Research Institute

**Grant number**: # 212200712

**Adaptation of chronic wasting disease (CWD) prion strains in hosts with different *PRNP* genotypes**

Camilo Duque Velasquez^a,c^, Elizabeth Triscott^a,c^, Chiye Kim^a,c^, Diana Moreno^a,c^, Judd Aiken^b,c^, and Debbie McKenzie^a,c^

^a^Department of Biological Science, University of Alberta, Edmonton, AB T6G 2G8, Canada; ^b^Department of Agriculture, Food & Nutritional Science, University of Alberta, Edmonton, AB T6G 2G8, Canada; ^c^Centre for Prions and Protein Folding Diseases, University of Alberta, Edmonton, AB T6G 2M8, Canada

**Aims**: The contagious nature of CWD epizootics and the PrP^C^amino acid variation of cervids (and susceptible sympatric species) guarantee the expansion of prion conformational diversity and selective landscapes where new strains can arise. CWD strains can have novel transmission properties including altered host range that may increase zoonotic risk as circulating strains diversify and evolve. We are characterizing the host adaptability of characterized CWD strains as well as CWD isolates from different cervid species in various enzootic regions.

**Material and Methods**: Characterized CWD strains as well as a number of isolates from hunter-harvested deer were bioassayed in our rodent panel (transgenic mice expressing cervid alleles G96, S96 and H95-PrP^C^, elk PrP^C^, bovine PrP^C^, and both hamsters and non-transgenic laboratory mice). Strain characteristics were compared using computer based scoring of brain pathology (e.g. PrP^CWD^brain distribution), western blot and protein misfolding cyclic amplification (PMCA).

**Results**: Transmission of various isolates resulted in the selection of strain mixtures in hosts expressing similar PrP^C^, particularly for polymorphic white-tailed deer and for Norwegian reindeer. As of the second passage, transmission of P153 moose prions from Norway has not resulted in emergence of strains with properties similar to any North American CWD strains in our taxonomic collection (Wisc-1, CWD2, H95^+^and 116AG).

**Conclusions**: Our data indicates polymorphic white-tailed deer can favor infection with more than one strain. Similar to transmission studies of Colorado CWD isolates from cervids expressing a single PrP^C^primary structure, the isolate from Norway reindeer (V214) represents a strain mixture, suggesting intrinsic strain diversity in the Nordfjella epizootic. The diversity of CWD strains with distinct transmission characteristics represents a threat to wildlife, sympatric domestic animals and public health.

**Funded by**: Genome Canada and Genome Alberta (Alberta Prion Research Institute and Alberta Agriculture & Forestry); NSERC

**Grant number**: #LSARP 10205; NSERC RGPIN-2017-05539

**Acknowledgement**: We would like to thank Margo Pybus (Alberta Environment and Parks) Trent Bollinger (University of Saskatchewan) for providing us with tissue samples from hunter-harvested deer and Sylvie Benestad for providing moose and reindeer samples.

**Combination of seeded aggregation and sFIDA for diagnostics of neurodegenerative diseases**

Alexandra Dybala^a^, Marlene Pils^b,c^, Lara Blömeke^b,c^, Oliver Bannach^a,b,c^, and Detlev Riesner^a^

^a^Institut für Physikalische Biologie, Heinrich-Heine-Universität Düsseldorf, Düsseldorf, Germany; ^b^Institute of Biological Information Processing (Structural Biochemistry: IBI-7), Forschungszentrum Jülich, Jülich, Germany; ^c^attyloid GmbH, Düsseldorf, Germany

**Aims**: The aim of the present study was to increase the sensitivity of the sFIDA assay for quantification of beta-amyloid (Aβ) and alpha-synuclein (aSyn) aggregates by introducing seeded aggregation.

**Material and Methods**: The technology of surface-based fluorescence intensity distribution analysis (sFIDA) was originally developed to detect and count prion protein particles prepared from scrapie-infected sheep brain and blood. More recently, sFIDA was applied to analyze Aβ and aSyn aggregates from CSF samples of Alzheimer’s and Parkinson’s Disease patients, respectively. In sFIDA, particles are immuno-captured to a glass surface, labeled with fluorescent antibody probes and imaged by fluorescence microscopy at the single particle level.

**Results**: In the present work, additional monomers were added to aggregates of Aβ or aSyn by seeded aggregation prior to microscopic analysis. The attached monomers were labelled either intrinsically with fluorescent dyes or after seeded aggregation by specific, fluorescently labelled antibodies. Seeded aggregation was carried out either after binding the seeds to the surface, or in solution, i.e. before binding to capture antibodies. Enhanced detection of Aβ peptides by seeded aggregation could not be achieved reproducibly with sufficient improvement of sensitivity. This was due to the very high tendency of self-aggregation of Aβ peptides, even if the length of the peptide was varied.

In contrast, the limit of detection for aSyn particles could be improved by two to three orders of magnitude. Seeded aggregation in solution was superior over aggregation on the surface. Optimized conditions included adjustment of pH, temperature and monomer concentration. Self-aggregation of aSyn did not interfere with the analysis.

**Conclusions**: Seeded aggregation in solution, binding to the capture antibodies on the surface, labelling seeded aggregates with antibody probes, and counting the aggregates in the fluorescence microscope was found as the optimal procedure for sensitive analysis of aSyn aggregates.

**Funded by**: iBrain – Interdisciplinary Graduate School for Brain Research and Translational Neuroscience, Heinrich-Heine-Universität Düsseldorf, and Forschungszentrum Jülich, Germany

**Understanding the key features of the spontaneous formation of *bona fide* recombinant prions through a new method allowing their consistent generation within hours**

Hasier Eraña^a,b,c^, Jorge M. Charco^a,b,c^, Carlos M. Díaz-Domínguez^a^, Miguel A. Pérez- Castro^a^, Enric Vidal^d^, Cristina Sampedro-Torres-Quevedo^a^, Rafael López-Moreno^a^, Sierra Espinar^d^, Ezequiel González-Miranda^a^, Sandra García-Martínez^a^, Aileen Santini^a^, Melisa Lázaro^a^, Leire Fernández-Veiga^a^, Juan Tasis-Galarza^a^, Nuria López-Lorenzo^e^, Mikel Valle^a^, Glenn Telling^f^, Mariví Geijo^g^, Jesús R. Requena^e^and Joaquín Castilla^a,c,h^

^a^Center for Cooperative Research in Biosciences (CIC bioGUNE), Basque Research and Technology Alliance (BRTA), Derio, Spain; ^b^ATLAS Molecular Pharma S. L., Derio, Spain; ^c^Centro de Investigación Biomédica en Red de Enfermedades infecciosas (CIBERINFEC), Carlos III National Health Institute, Madrid, Spain; ^d^Centre de Recerca en Sanitat Animal (CReSA) – Institut de Recerca i Tecnologia Agroalimentàries (IRTA), Barcelona, Catalonia; ^e^CIMUS Biomedical Research Institute & Department of Medicine, University of Santiago de Compostela-IDIS, Santiago de Compostela, Spain; ^f^Prion Research Center (PRC), Colorado State University, Fort Collins, Colorado, USA; ^g^Animal Health Department, NEIKER-Basque Institute for Agricultural Research and Development. Basque Research and Technology Alliance (BRTA), Derio, Spain; ^h^IKERBASQUE, Basque Foundation for Science, Bilbao, Spain

**Aims**: Different methodologies developed to achieve the spontaneous generation of infectious prions *in vitro* have given rise to a large spectrum of misfolded proteins and amyloid aggregates with distinct properties. From those unable to propagate and cause disease *in vivo* or those requiring overexpressing animal models and multiple serial inoculations, to infectious prions with high titers, able to cause disease in wild-type animals. Herein, we break down a novel and easily scalable methodology that consistently leads to the spontaneous misfolding of recombinant PrP into infectious, high titer, *bona fide* prion preparations, allowing the understanding of the minimal requirements for such an event.

**Material and Methods**: Using recombinant bank vole PrP and Protein Misfolding Shaking Amplification (PMSA) technique, derived from the well-known PMCA, we analyzed all potentially relevant parameters leading to consistent spontaneous prion misfolding. Among others, are bead material and size, shaking parameters, protein concentration, presence of cofactors, and process temperature.

**Results**: Apart from developing a methodology to obtain infectious synthetic prions with high titers within minutes, we achieved the formation of a variety of strains with specific and differential features. Additionally, the critical parameters for consistent spontaneous *bona fide* prion formation were determined, providing relevant information on the mechanism promoting this event, central in prion pathogenesis.

**Conclusions**: Through fine-tuning of PMSA operational conditions, we can consistently produce distinct strains of highly infectious recombinant prions in a spontaneous manner, offering an invaluable tool to study every aspect contributing to spontaneous prion formation. Although protein concentration, shaking parameters, and process temperature are required to be within a specific range, the most critical parameter for consistent spontaneous formation of *bona fide* recombinant prions is the glass surface, provided in this system by determined amounts of glass beads.

**Funded by**: Spanish Ministry of Science and Innovation

**Grant number**: This study was funded by MINECO research project references RTI2018-098515-B-I00 and PID2021-122201OB-C21, and by RedPRION (Interreg POCTEFA EFA148/16).

**Prion Protein Gene Diversity in German Cervids**

Sonja Ernst^a^, Martin H. Groschup^a^, Balal Sadeghi^a^, Markus Keller^a^, Dolores Gavier-Widén, Jörn Gethmann^a^, Fiona Houston^c^, and Christine Fast^a^

^a^Friedrich-Loeffler-Institut, Institute of Novel and Emerging Diseases, Isle of Riems, Germany; ^b^National Veterinary Institute (SVA), Uppsala, Sweden; ^c^Division of Infection and Immunity, The Roslin Institute and The Royal Dick School of Veterinary Studies, University of Edinburgh, Edinburgh, UK

**Aims**: Chronic Wasting Disease (CWD), a Transmissible Spongiform Encephalopathy (TSE) affecting multiple Cervid species, is caused by a cellular prion protein (PrP^C^) misfolding into its pathological isoform (PrP^CWD^). While the disease has been endemic since the 1960s in North America and still continues to spread, only six years ago the first European case was described in Norway. However, since then additional cases, not only in its contagious but also in a novel ‘atypical’ form in moose *(Alces alces)* and red deer *(Cervus elaphus)*, have been discovered in Scandinavia, now endangering all European Cervids. Susceptibility to CWD strongly depends on the structure of the prion protein gene *(PRNP)*, thus this study aims to investigate the diversity of the *PRNP* of the German Cervid population.

**Methods**: We reached out to hunters, forest offices and deer keepers to sample red deer, roe deer *(Capreolus capreolus)* and sika deer *(Cervus nippon)* throughout Germany during the past hunting season (2021/2022). After dividing Germany into 12 regions, from each region and species, a sample of 149 animals will be genotyped. This sample size is suitable to detect polymorphisms with an occurrence of more than 2% in the population. When less than 149 samples are collected, all animals will be genotyped.

Sanger Sequencing the Open Reading Frame *(ORF)* on exon three of the *PRNP* provides detailed information on possible amino acid exchanges, single nucleotide polymorphisms and silent mutations. This will allow us to define different genotypes under the aspect of recently published data from similar studies in North America and Europe.

**Results**: Samples from a total of 834 red deer, 2170 roe deer and 47 sika deer throughout Germany were sent back to FLI. The different numbers of samples per species reflect the composition of the natural population, as there live far more roe than red deer in Germany. Since there are only five regions where they are native, Sika deer are rare in Germany. The distribution of the samples covers most parts of Germany, with only smaller regions that have not been sampled yet. Preliminary results of roe and red deer show genotype variation within the red deer population, whereas the examined roe deer are homologous so far.

**Conclusion**: This study aims to get new detailed insights into the vulnerability of German Cervid population to CWD, to improve the European CWD surveillance and control measures and to estimate the threat of a CWD introduction into Germany.

**Seed Amplification Assay accurately detects misfolding α-Synuclein in CSF samples from PD and iRBD patients of the DeNoPa cohort**

Carly Farris^a^, Luis Concha^a^, Sandrina Weber^b^, Mohammed Dakna^b^, Elisabeth Lang^b^, Yihua Ma^a^, Maritta Starke^b^, Claudio Soto^a,c^, and Brit Mollenhauer^b^

^a^R&D Unit, Amprion, San Diego, US; ^b^Paracelsus-Elena-Klinik, Kassel, Germany; ^c^University of Texas McGovern Medical School, Houston, TX, USA

**Aims**: Diagnostic biomarkers are most valuable in the earliest stage of Parkinson’s disease (PD). Because of the high rate of misdiagnosis, early clinical diagnosis cannot be used as gold standard to evaluate accuracy of a diagnostic test. In addition, access to pathologically confirmed samples can be challenging. Our goal was to determine sensitivity and specificity of the αSyn Seed Amplification Assay (αS-SAA) for manifest and prodromal PD using the longitudinal DeNoPa cohort. Thus, we compared the αS-SAA results of baseline samples (collected weeks after diagnosis) versus their 8y clinical diagnosis.

**Material and Methods**: We blindly analyzed samples from 113 PD, 29 isolated REM sleep behavior disorder (iRBD), and 64 healthy control (HC) patients. Patients were categorized based on baseline (BL) assessment and underwent biannual clinical follow-ups at a single center. CSF samples were analyzed in triplicate, using recombinant αSyn substrate. αS-SAA conditions used in this study have been described elsewhere.

**Results**: Of the 113 BL-PD cases, 99 (87.6%) had a final synucleinopathy diagnosis, including 95 PD (84.1%), 2 DLB (1.8%), and 2 MSA (1.8%). The other 14 (12.4%) BL-PD patients had a non-synucleinopathy final diagnosis, including 3 (2.7%) vascular PD (vPD), 5 (4.4%) essential tremor (ET) and/or dystonia (DT), 2 (1.8%) restless leg syndrome (RLS), and 4 (3.5%) progressive supranuclear palsy (PSP).

The assay was highly sensitive, as 94 (95%) of the 99 patients with final synucleinopathy diagnosis were αS-SAA-positive, including 91 (92%) PD, 1 (1%) DLB, and 2 (2%) MSA. One of the MSA samples presented the low fluorescence signature previously reported for MSA αSyn seeds. Of the 14 cases without a synucleinopathy, 12 were found αS-SAA-negative. Of the 64 HC subjects, 62 (96.9%) were αS-SAA-negative. Considering BL samples only, the assay performed with a sensitivity of 94.6% (95%CI 86.7–98.5%) and a specificity of 98% (95%CI 89.4–100%).

Of the 56 iRBD CSF samples, 53 (94.6%) were αS-SAA-positive and 27 (93.1%) of the 29 patients had an αS-SAA(+) sample at BL or follow-up visit. This level of detection at BL is 64.8% higher than previously reported for a sub-cohort of these samples.

**Conclusions**: αS-SAA enables the sensitive and specific detection of misfolded αSyn in early PD and even early iRBD. Compared to baseline clinical assessment, αS-SAA was 10% more specific. Remarkably, CSF from iRBD was αS-SAA-positive up to 8 years before phenoconversion and 2 cases were found positive only months after the first iRBD symptoms.

**Funded by**: Michael J Fox Foundation

**Grant number**: MJFF-021233

**Semi-quantitative αS-SAA detects no difference in αSyn seeds in CSF from prodromal to phenoconversion in longitudinal samples**

Carly Farris^a^, Yihua Ma^a^, Claudio Soto^c^, Brit Mollenhauer^b^, and Luis Concha^a^

^a^R&D Unit, Amprion, San Diego, US; ^b^Paracelsus-Elena-Klinik, Kassel, Germany; ^c^University of Texas McGovern Medical School, Houston, TX, USA

**Aims**: To determine if CSF levels of αSyn seeds increase in subjects that progress from prodromal to Parkinson’s disease (PD) or dementia with Lewy bodies (DLB).

**Material and Methods**: We developed a semi-quantitative αSyn seed amplification assay (SemiQ αS-SAA) that estimates the seeding activity (SD50) of misfolded αSyn in CSF. SD50 was estimated by means of serial dilutions and the Spearman-Kärber model. The method was evaluated with immunoprecipitated PD-CSF samples and also by spiking synthetic seeds in healthy control CSF. CSF samples from patients with isolated REM sleep behavior disorder (iRBD) were longitudinally collected and evaluated by SemiQ αS-SAA. Some of these subjects phenoconverted to full blown disease and CSF samples were semi-quantified at baseline iRBD evaluation and after phenoconversion. We also developed a synthetic CSF matrix that can replace healthy control (HC) CSF as diluent and enables semi-quantitation of large quantities of samples.

**Results**: The SemiQ αS-SAA estimated seeding activities (SD50) that were proportional to the number of immunoprecipitations (IPs) performed on PD-CSF. Compared to the control samples (no-IP and no-antibody), 2 serial IPs reduced the seeding activity by ~35%, while 3 serial IPs showed a ~ 60% reduction. Thus, the assay correctly estimated lower levels of PD αSyn seeds in human CSF. When spiking 2 levels of synthetic αSyn seeds (10-fold difference) in HC CSF, the SemiQ αS-SAA estimated the difference to be ~13-fold. This result suggests that the estimated seeding activity does correlate with the mass of seeds in a sample.

We did not find significant differences in SD50 during progression from iRBD to full blown PD in a case with longitudinal samples collected over 60 months. Comparable results were found in 4 more cases that showed no difference in SD50 for samples collected at BL and final visit. Two of these cases included a final sample collected after phenoconversion to PD and DLB, while the other 2 remained iRBD. Lastly, SD50 estimations using synthetic CSF increased the separation window, probably because it lacks CSF inhibitors that reduce amplification of low levels of seeds.

**Conclusions**: αS-SAAs are perceived as non-quantitative because no correlation between levels of αSyn seeds and disease progression has been shown to date. Our results indicate that αSyn behaves like amyloid-β and prions, where misfolding and accumulation occurs before the onset of first symptoms and there is no correlation with disease severity. Moreover, our data suggest that levels of αSyn seeds in CSF reach a plateau during the prodromal stage of the disease.

**Funded by**: Michael J Fox Foundation

**Grant number**: MJFF-021233

**First detection of classical scrapie confirmed by immunoblot in sheep in Eastern Libya.**

Christine Fast*^a^, Fawzia F. Abdalla*^b^, Sonja Ernst^a^, Martin H. Groschup^a^, and Monier Sharif^b^* Both authors are considered as first authors

^a^Friedrich-Loeffler Institut, Isle of Riems, Germany; ^b^Department of Pathology and Clinical Pathology, Faculty of veterinary medicine, Omar Al-Mukhtar University, Al-Bayda, Libya

**Aims**: Scrapie was first reported by the Libyan national centre for animal health in 2014, but the distribution of this disease in Libya is still unknown and neglected. Here we report several classical scrapie cases from different herds in Eastern Libya submitted between 2018 and 2022.

**Methods**: In total 10 sheep from 10 herds were submitted to the Veterinary Teaching Hospital at Omar Al-Mukhtar University, Al-Bayda, Libya for post-mortem examination. Further protein biochemical examination includes a PTA-Immunoblot to detect the pathological prion protein (PrP^Sc^), which was done by FLI – Isle of Riems, Germany.

**Results**: The sheep were between 2 and 5 years of age. The clinical signs, which progressed slowly over one to three months included weight loss and behavioural abnormalities as well as neurological disorders. Among others tremors, bruxism, repeated licking of the lips, typical down and upward movements of the head, hesitant and uncertain gait, ataxia of the hind limbs, occasional falls, blindness, and bunny hopping were described by the owners. Some animals were found dead without any previous clinical signs. The histomorphological examination revealed, most prominent in the brain stem, characteristic signs of a spongiform encephalopathy, with neuronal vacuolation (single or multiple vacuoles) and spongiform change in the gray matter neuropil as well as gliosis. Additionally, mild perivascular infiltration and astrocytosis were observed in vacuolated areas. In one animal no histomorphological alterations was seen. By PTA-immunoblot the presence of classical scrapie has been confirmed.

**Conclusions**: Classical scrapie exists in Libya and is widely distributed. Moreover, almost all cases were easily diagnosed by a combination of clinical signs and histopathological examination, indicating a long lasting disease history in Eastern Libya. Therefore, the results of the study presented here could not only serve to raise awareness of the disease among sheep and goat farmers, but also should lead to the implementation of control measures in the future.

**SFPQ as a plasma biomarker to distinguish Creutzfeldt – Jakob disease and rapidly progressive Alzheimer’s disease**

Leticia C. Fernandez#, Neelam Younas#, Stefan Goebel, Kathrin Dittmar, Peter Hermann, and Inga Zerr

# equal contribution Prion Research Group, National Reference Center for Surveillance of TSE, Department of Neurology, University Medical Center Göttingen, Germany

**Aims**: Measuring the SFPQ (splicing factor proline and glutamine rich) levels in body fluids, specifically blood, as a potential blood-based biomarker for rapid progressive dementias.

**Material and Methods**: A cohort of 40 patients (10 subjects per group) was evaluated. The patients were diagnosed with either Alzheimer´s disease (AD), rapidly progressive Alzheimer´s disease (rpAD) or Sporadic Creutzfeldt – Jakob disease (sCJD), and healthy patients as control (HC). Enzyme-linked immunosorbent assay (ELISA) was performed to quantify SFPQ levels in plasma.

**Results**: SFPQ levels were observed and measured in plasma samples of neurodegenerative diseases. Here, we found that the levels of SFPQ protein in the plasma were significantly different between rapidly progressive dementias, specifically CJD and rpAD.

**Conclusions**: Since SFPQ levels in plasma differs from AD and rpAD from HC and CJD we propose that SFPQ levels in Plasma can be used as a diagnostic tool to differentiate CJD from rpAD. Additionally levels of SFPQ in plasma could be a biomarker for differential diagnosis of rapidly progressive dementias.

**The cellular prion protein as a potential receptor in neurodegenerative diseases**

Anna-Lisa Fischer, and Matthias Schmitz

Inga Zerr Neurology, Universitätsmedizin Göttingen, Göttingen, Germany

**Aims**: Neurodegenerative diseases such as Alzheimer´s disease (AD) or Parkinson´s disease (PD) are associated with the accumulation of aggregated proteins. These diseases demonstrated phenotypic diversity and propagation of pathology that is reminiscent of prion diseases. It is suggested that amyloid-β, alpha synuclein, and tau proteins share common structural, biological, and biochemical features, as well as similar mechanisms of aggregation and self-propagation in a prion-like manner. Propagation of protein misfolding in these diseases may therefore occur via mechanisms similar to those underlying prion pathogenesis. In this work, we focused on cellular prion protein (PrPC) as a potential receptor protein for misfolded proteins, supporting internalization and interaction.

**Material and Methods**: To examine a possible direct interaction we subjected recombinant human PrPC as well as recombinant misfolded proteins (aSyn, tau) to surface plasmon resonance spectroscopy (SPR). SH-SY5Y (SHWT) and stable PRNP transfected SH-SY5Y PrP (SHPrP) cells were treated with different recombinant misfolded proteins under same conditions.

**Results**: We observed aneffect of PrPC on the internalization of misfolded proteins and the interaction between PrPC and misfolded proteins. SPR results presented misfolded proteins as direct interaction partners of PrPC. SHPrP cells showed a significantly higher amount of internalized misfolded proteins compared to SHWT cells.

**Conclusions**: In conclusion, our experiments indicate PrPC as a receptor for misfolded proteins, promoting the internalization and interaction. These findings contribute to a better understanding of the pathological mechanism in neurodegenerative diseases, which is important for future diagnostics or therapies.

**Optimizing prion vaccination in a transgenic mouse model of Gerstmann-Sträussler-Scheinker**

Madeleine Fleming^a,b^, Andrew Fang^a,b^, Brian Tancowny^a, b^, Glenn C. Telling^c^, Holger Wille^a,b,d^

^a^Department of Biochemistry; ^b^Centre for Prions and Protein Folding Diseases; ^c^Prion Research Center, Colorado State University, Fort Collins, Colorado, USA; ^d^Neuroscience and Mental Health Institute, University of Alberta, Edmonton, Alberta, Canada

**Aims:** Gerstmann–Sträussler–Scheinker disease (GSS) is a rare, genetic prion disease in humans that is characterized by dementia and ataxia and is caused by point mutations such as the proline to leucine mutation at codon 102 (P102L). The TgP101L transgenic mouse line overexpresses mutant PrP carrying the murine equivalent of the P102L mutation and develops GSS disease symptoms at 177 days of age (±17 days). Previously we developed a model-based prion vaccine by using the proposed β-solenoid structure of PrP^Sc^ and modified the innocuous fungal prion HET-s to mimic a surface epitope of PrP^Sc^. The goal of this study was to optimize the immunization protocol in vaccine efficacy trials using TgP101L transgenic mice.

**Material and Methods:** In this study, we compared alum, saponin-based QS21, and Freund’s adjuvant in prion vaccination trials using TgP101L transgenic mice and a prime-boost immunization schedule (primary dose followed by three boosts at two-week intervals). We collected pre- and post-immune sera and measured antibody titres following each immunization using an indirect ELISA. We also observed symptom onset and disease progression relative to non-immunized controls. Lastly, we conducted an experiment where the animals received additional prion vaccine boosters every 100 days following the initial prime-boost schedule.

**Results and Conclusions:** The observed antibody titres illustrate that alum adjuvant leads to a higher antibody production than both Freund’s and QS21 adjuvants. Mice immunized with the prion vaccine and either alum, QS21, or Freund’s adjuvant show a significant delay in symptom onset at 495 days of age (±42 days), 479 days of age (±54 days), or 448 days of age (±39 days), respectively. Relative to the disease onset for unimmunized TgP101L mice, this represents an increase in health-span of ~280%, ~270%, and ~250%, respectively, emphasizing the protective effect of the model-based prion vaccine. Additional vaccine boosts every 100 days helped to maintain the antibody titre, however there was an earlier onset of prion disease symptoms at 421 days of age (±105 days), compared to mice on the regular prime-boost schedule. This observation suggests T-cell exhaustion, due to persistent antigen stimulation and prolonged exposure to inflammatory cues, as a potential cause for the reduced vaccine efficacy. Moreover, this finding points towards a prominent role of cellular immune response elements in the observed protection against neurodegeneration. Optimizing the administration of the model-based prion vaccine is essential for its potential use in the prevention of prion diseases.

**Funded by:** The CJD Foundation, Alberta Innovates/Alberta Prion Research Institute

**Grant number:** 201900006

**Fatal Familial Insomnia in a cerebral organoid model**

Simote T. Foliaki^a^, Anna Smith^a^, Benjamin Schwarz^b^, Eric Bohrnsen^b^, Catharine M. Bosio^b^, Katie Williams^a^, Hailey Lachenauer^c^, Bradley R. Groveman^a^, and Cathryn L. Haigh^a^

^a^Laboratory of Persistent Viral Diseases; ^b^Laboratory of Bacteriology; ^c^Research Technologies Branch, National Institute of Allergy and Infectious Diseases, Division of Intramural Research, Rocky Mountain Laboratories, National Institutes of Health, Hamilton, MT, USA

**Aims**: Fatal familial insomnia (FFI) is a rare neurodegenerative disease caused by a dominantly inherited single amino acid substitution (D178N) within the prion protein (PrP). No *in vitro* human brain tissue model for this disease has previously been available. As a result, the cellular and molecular pathogenesis of FFI in the human brain and how this mutation exerts its damaging effect on brain cells are still unknown. This study aims to investigate this damaging effect in human brain tissue using a human cerebral organoid model.

**Material and Methods**: CRISPR-Cas9 engineering was used to introduce the D178N mutation into the PRNP gene in human induced pluripotent stem cells. We made FFI cerebral organoids using these cells and compared them with their isotype control organoids to investigate disease-associated cellular changes. We assessed the mutant organoids for prion disease pathology including prion seeding activity by RT-QuIC, Proteinase-K resistant prion deposition, altered PrP post-translational processing, and astrogliosis. The efficacy of neuronal network communication and the associated changes in neurotransmitters were measured by Multi-electrode arrays and Liquid Chromatography Mass-Spectrometry (LCMS). Oxidative stress levels and mitochondrial activity were assessed by Flow cytometry, Immuno-blotting, and Seahorse assay. Autophagy and mitophagy were assessed by Flow cytometry and Transmission electron microscopy. Changes in cellular metabolites and lipid compositions were determined by LCMS.

**Results**: We found that, in the absence of other hallmarks of FFI, the D178N organoids exhibited astrogliosis with cellular oxidative stress. Abnormal post-translational processing of PrP was evident but no tissue deposition or propagation of mis-folded PrP isoforms were observed. In addition, the neuronal electrophysiological function was compromised and neurotransmitter levels altered. Underlying these dysfunctions were changes in cellular energy homeostasis, with substantially increased glycolytic and Krebs cycle intermediates, and greater mitochondrial activity. This increased energy demand in D178N organoids was associated with increased mitophagy and depletion of lipid droplets, resulting in shifts in cellular lipid composition. Using a double mutation (178 NN) we could confirm that most changes were caused by the presence of the mutation rather than interaction with PrP molecules lacking the mutation

**Conclusions**: Our data strongly suggests that shifting biosynthetic intermediates and oxidative stress, caused by an imbalance of energy supply and demand, results in astrogliosis with compromised neuronal activity in FFI organoids. They further support that many of the disease-associated changes are due to a corruption of PrP function because of the mutation and do not require propagation of PrP mis-folding.

**Funded by**: This work was supported by the Division of Intramural Research, National Institute of Allergy and Infectious Disease.

**Modulation of PrP^C^expression affects cancer progression *in vivo***

Natalia Fortunato de Miranda^a^, Shana Portelli^a^, Frederic Hollande^b^, Theo Mantamadiotis^a^, and Victoria A Lawson^a^

^a^Department of Microbiology and Immunology and Peter Doherty Institute for Infection and Immunity, The University of Melbourne, Melbourne, Australia; ^b^Department of Clinical Pathology and The University of Melbourne Centre for Cancer Research, The University of Melbourne, Melbourne, Australia

**Aims: An increase in tumor-specific** PrP^C^expression is associated with poor patient outcome. However, the role of PrP^C^as either a cause or consequence of malignant transformation and the consequences of therapeutic PrP^C^modulation in the context of neurodegeneration on tumor initiation and progression are yet to be established. In this study we investigated the effect of PrP^C^expression on tumor initiation and progression in two mouse models of cancer.

**Material and Methods**: Tumors were induced in *Prnp* knock-out *(Prnp^KO^)* and congenic *Prnp* wildtype *(Prnp^WT^)* mice in a colitis model of colorectal cancer using the carcinogen azoxymethane and inflammatory reagent, dextran sulfate sodium salt. Tumors were induced in *Prnp^KO^* mice and their *Prnp^WT^* littermates in a glioblastoma model by constitutive activation of the PI3K pathway via expression of a *Pik3ca^H1047R^* oncogenic mutant and deletion of phosphatase and tensin homolog (*Pten^Δ^*) tumour suppressor gene.

**Results**: In the colitis and glioblastoma models of cancer both *Prnp^KO^* and *Prnp^WT^* mice developed tumors. However, in both cancer models, tumors in *Prnp^KO^*mice had a greater tumor burden than *Prnp^WT^*mice. These *in vivo* observations suggest that PrP^C^expression is not required for the development of tumors, but rather appears to modulate tumor progression and malignancy.

**Conclusions**: These observations suggest that in cancer cells, the modulation of PrP^C^expression may regulate tumor progression.

**Funded by**: SP is supported by a Carol Willessee PhD scholarship and we are grateful for the support from the CJD Support Group Network in Australia.

**The chronic wasting disease agent from white-tailed deer fails to adapt to sheep upon second passage**

Alexis J. Frese, Eric D. Cassmann, M. Heather West Greenlee, and Justin J. Greenlee

Department of Agriculture, Virus and Prion Research Unit, National Animal Disease Center, Agricultural Research Service, United States Ames, IA, USA

**Aims**: Interspecies transmission of prion disease is highly variable and dependent upon multiple factors. The chronic wasting disease (CWD agent) of mule deer is transmissible to sheep after intracranial inoculation, with similar clinical signs and incubation periods to scrapie. This study used sheep and transgenic mice to investigate the susceptibility of sheep to the CWD agent from white-tailed deer and to characterize subsequent passages of the resulting disease agent.

**Material and Methods**: Suffolk sheep (n = 15) with PRNP genotypes VRQ/ARQ, ARQ/ARQ, or ARQ/ARR were inoculated intracranially with CWD prions from white-tailed deer. Western blots and ELISA assays were performed on brain and lymphoid tissues to analyze PrP^Sc^accumulation. Brain material from one positive sheep with the ARQ/ARQ genotype (#424) was used to inoculate mice expressing the mouse (C57BL/6; 20 ul of 10% homogenate) or sheep VRQ (Tg338; 20 ul of 1% homogenate) PRNP.

**Results**: PrP^Sc^was detected in 4/15 sheep in the brainstem at the level of the obex, with an average incubation period of 41 months. Sheep #424 (the animal whose brain was passaged to mice) also had PrP^Sc^in the cerebrum, but PrP^Sc^was not detectable in any lymphoid tissues.

Inoculum from the CWD-positive sheep did not cause disease or result in detectable PrP^Sc^in ovinized mice (Tg338) after incubation of 800 days. However, upon passage to C57BL/6, 4/20 mice were positive with an average incubation period of 684 days. Upon second passage to C57BL/6 mice, the attack rate increased to 14/15 with a mean incubation period of 380 dpi.

**Conclusions**: The CWD agent from white-tailed deer transmits to sheep via intracranial inoculation demonstrating that there is not an absolute species barrier between sheep and white-tailed deer. In affected sheep, distribution of PrP^Sc^was limited to the central nervous system, suggesting that environmental shedding of CWD prions from sheep would be negligible. CWD isolates from cervids usually do not transmit to wild-type mice, but after passage through sheep, this isolate readily passed to C57BL/6 mice. It was unexpected that the CWD agent from sheep did not further adapt in Tg338 mice. This data suggests that the CWD agent from white-tailed deer is unlikely to present a major risk to sheep, but further assessments should be conducted in mice expressing the ARQ ovine prion protein.

**Funded by**: The United States Department of Agriculture

**Grant number**: N/A

**Acknowledgement**: The authors wish to thank Leisa Mandell and Kevin Hassall for technical assistance.

**Prion disease in TgMHu2ME199K mice skeletal muscle**

Kati Frid^a^, Orli Binyamin^a,b^, Areen Usman^a,b^, and Ruth Gabizon^a^

**^a^**Department of Neurology, The Agnes Ginges Center for Human Neurogenetics, Hadassah University Hospital, Jerusalem, Israel; **^b^**Medical School, The Hebrew University, Jerusalem, Israel

**Aims**: Myositis and PrP accumulation were observed in muscles of diverse prion affected human and animal models. While in transmissible prion disease muscles are most likely infected by the corresponding brain prions, in genetic CJD muscle pathology may also result from a spontaneous mechanism related to the individual properties of each mutant PrP protein. In this work, we looked for morphological and pathological abnormalities in the muscles of TgMHu2ME199K mice, a model of genetic CJD linked to the E200K PrP mutation. Next, we investigated whether mutant PrP in these mice muscles accumulates as a PK resistant form in an age and disease dependent way, as is the case for PrP in the brains of TgMHu2ME199K mice. Based on the results from this project, we aim to consider whether a muscle biopsy from humans can contribute to early disease diagnosis and treatment follow-up in gCJD.

**Material and Methods**: Muscle tissue from young (asymptomatic) and adult (sick) TgMHu2ME199K mice were tested by immunoblotting and immunohistochemistry for a line of parameters. In some experiments, we also examined muscles from adult TgMHu2ME199K mice treated with Granagard, a nano-formulation of PSO which significantly delays disease progression in these mice. First, we tested the morphology and pathology of muscle tissue in TgMHu2ME199K mice as compared to wt mice. Next, we tested the aggregation and PK resistance of muscle PrP as compared to brain PrP at both ages. Also, we compared by immunofluorescence for MBP and NeuN the location of aggregated PrP. We also examined the expression of PAX7, a marker of muscle stem cells, in muscle from wt as well as treated and untreated TgMHu2ME199K mice.

**Results**: Histological results revealed morphological abnormalities in the muscles of sick TgMHu2ME199K mice, including central nucleus and fat cell infiltrations. Contrarily to PrP in brains of adult TgMHu2ME199K mice, in skeletal muscles PrP was aggregated but not PK resistant. Immunofluorescence with NeuN suggest reduced enervation in adult mice muscles. Most important, PAX7 immunofluorescence demonstrated reduced generation of stem cells in adult mice muscles, which was mostly corrected by administration of Granagard.

**Conclusions**: We conclude that the pathological findings in muscles of adult TgMHu2ME199K mice may result from both reduced enervation, aberrant accumulation of mutant PrP and reduced generation of stem cells. Interestingly, Granagard restored generation of endogenous stem cells in these mice. It remains to be established whether results from muscle biopsies obtained from gCJD patients can provide similar results.

**Funded by**: Granalix biotechnologies

**Evaluation of the seeding activity of alpha-synuclein in brain and cerebrospinal fluid tissue samples**

Soňa Galušková^a^, Tibor Moško^a^, Radoslav Matěj^b,c^, Petr Dušek^d^, and Karel Holada^a^

^a^Institute of Immunology and Microbiology of the 1^st^Faculty of Medicine, Charles University and General University Hospital, Prague, Czech Republic; ^b^Department of Pathology of the 3^rd^Faculty of Medicine, Charles University and University Hospital Královské Vinohrady, Prague, Czech Republic; ^c^National Reference Laboratory for Human Prion Diseases, Department of Pathology and Molecular Medicine of the 3^rd^Faculty of Medicine, Charles University and University Thomayer Hospital, Prague, Czech Republic; ^d^Department of Neurology and Center of Clinical Neuroscience of the 1^st^Faculty of Medicine, Charles University and General University Hospital, Prague, Czech Republic

**Aims**: Early and specific diagnosis of synucleinopathies is essential for the prognosis and the life quality of the patient. However, diagnosis remains difficult with up to 25% cases being misdiagnosed. Real-Time Quaking-Induced Conversion (RT-QuIC) assay represents a new promising diagnostic method of synucleinopathies.

We evaluated the ability of RT-QuIC to detect the presence of α-synuclein seeding activity in *post-mortem* brain homogenate (BH) and cerebrospinal fluid (CSF) samples.

**Material and Methods**: Total of 15 BH and 14 CSF samples were obtained *post-mortem* from patients with autopsy confirmed Dementia with Lewy bodies (DLB, n = 6), Alzheimer disease with Amygdala Lewy Bodies (AD/ALB, n = 3) and Creutzfeldt-Jakob disease with α-synuclein deposits (CJD/αSyn = 6). Control group (n = 14 for BH and n = 17 for CSF) consisted of other neurodegenerative diseases.

End point seeding activity of samples was analyzed using RT-QuIC developed by Groveman *et al*., 2018. Reaction mix (98 or 85 µl) consisted of 40 mM phosphate (pH = 8.0), 170 mM NaCl, 10 µM Thioflavin T (ThT) and 0.1 mg/ml human recombinant (WT) α-synuclein, was seeded with 2 µl of serially diluted BH or 15 µl of CSF sample. The mixture was supplemented with six 800-micron silica beads. Reaction was incubated 60 hours at 42°C with incubation (1 min) and shaking (400 rpm, 1 min) cycles.

**Results**: Seeding activity of aggregated α-synuclein was detected in 13 out of 15 BH samples. Two CJD/αSyn samples were classified as negative. Samples gave the highest fluorescence signal at 10^−2^– 10^−3^dilutions. At 10^–5^all samples, except one, gave negative results. Samples with DLB gave the highest signal with the mean max fluorescence value (MMFV) 12.9 × 10^4^. The group of samples with CJD/αSyn gave the lowest signal, with the MMFV 9 × 10^4^.

Analyzing CSF samples, the seeding activity was detected in all samples except one with AD/ALB diagnosis. 100x diluted CSF samples gave generally better results, with the highest MMFV 23 × 10^4^for DLB samples. Similarly, as for the BH, samples with CJD/αSyn gave the lowest signal with the MMFV 15 × 10^4^.

**Conclusions**: Our pilot retrospective study confirmed the ability of RT-QuIC to detect seeding activity of α-synuclein in the patient samples. Interestingly, CSF samples gave overall better results than BH. Samples with DLB diagnosis gave the best RT-QuIC performance, either BH or CSF. The lowest ThT signal in CJD/αSyn samples may corresponds to younger age of the patients and suggests that α-synuclein deposits might be less developed than in other groups of patients.

**Funded by**: The Ministry of Health Czech Republic and Czech Health Research Council/ Charles University Grant Agency/ General University Hospital Grant Agency

**Grant number**: NU21-04-0053/ 36,252/ GIP-20-L-13-212

**Acknowledgement**: We would like to thank Dr. Byron Caughey for providing bacterial plasmids with recombinant α-synuclein.

**The VM1 subtype of sporadic Creutzfeldt-Jakob disease: phenotypic and molecular characterization of a novel subtype of human prion disease**

Ellen Gelpi*^a,b^, Simone Baiardi*^c,d^, Carlos Nos^e^, Sofia Dellavalle^c^, Iban Aldecoa^b,f^, Raquel Ruiz^g^, Lourdes Ispierto^h^, Domingo Escudero^h^, Virgina Casado^i^, Elena Barranco^j^, Anuncia Boltes^k^, Laura Molina-Porcel^b^, Nuria Bargalló^l^, Marcello Rossi^c^, Angela Mammana^c^, Anna Ladogana^m^, Elisabeth Stoegmann^n^, Ingrid Simonitsch-Klupp^o^, Gregor Kasprian^p^, Sigrid Klotz^a^, Romana Höftberger^a^, Herbert Budka^a^, Gabor G. Kovacs^q,r,s^, Isidre Ferrer^t^, Sabina Capellari^c,u^, Raquel Sanchez-Valle^b,v^, and Piero Parchi^c,d^ *contributed equally

^a^Division of Neuropathology and Neurochemistry, Department of Neurology, Medical University of Vienna and Austrian Reference Center for Human Prion Diseases (ÖRPE); ^b^Neurological Tissue Bank of the Biobank-Hospital Clinic-IDIBAPS, Barcelona, Spain; ^c^IRCCS, Istituto delle Scienze Neurologiche di Bologna, Bologna, Italy; ^d^Department of Experimental, Diagnostic and Specialty Medicine (DIMES), University of Bologna, Bologna, Italy; ^e^General Subdirectorate of Surveillance and Response to Emergencies in Public Health, Department of Public Health in Catalonia, Barcelona, Spain; ^f^Department of Pathology, Center for Biomedical Diagnosis, Hospital Clinic de Barcelona, University of Barcelona, Barcelona, Spain; ^g^Department of Immunology, Center for Biomedical Diagnosis, Hospital Clinic, Barcelona, Spain; ^h^Cognitive and Movement Disorders Unit, Hospital Germans Trias i Pujol de Badalona, Barcelona, Spain; ^i^Neurology Department, Hospital de Mataró, Barcelona, Spain; ^j^Department of Geriatrics, Hospital General de Granollers, Barcelona, Spain; ^k^Department of Neurology, Hospital General de Granollers, Barcelona, Spain; ^l^Radiology Department, Image Diagnosis Center, Hospital Clínic, Barcelona, Spain and Magnetic Resonance Image core facility of IDIBAPS, Barcelona, Spain; ^m^Department of Neuroscience, Istituto Superiore di Sanità, Rome, Italy; ^n^Department of Neurology, Medical University of Vienna, Vienna, Austria; ^o^Department of Pathology, Medical University of Vienna, Vienna, Austria; ^p^Department of Biomedical Imaging and Image-Guided Therapy, Medical University of Vienna, Vienna, Austria; ^q^Tanz Centre for Research in Neurodegenerative Disease, University of Toronto, Toronto, Ontario, Canada; ^r^Department of Laboratory Medicine and Pathobiology and Department of Medicine, University of Toronto, Toronto, Ontario, Canada; ^s^Laboratory Medicine Program & Krembil Brain Institute, University Health Network, Toronto, Ontario, Canada; ^t^Department of Pathology and Experimental Therapeutics, University of Barcelona; Bellvitge University Hospital-IDIBELL; CIBERNED; Barcelona; Spain; ^u^Department of Biomedical and Neuromotor Sciences (DIBINEM), University of Bologna, Bologna, Italy; ^v^Neurology Department, Alzheimer disease and other cognitive disorders unit, Hospital Clinic de Barcelona, Spain

**Aims**: To describe a novel sporadic Creutzfeldt-Jakob disease (sCJD) subtype.

**Material and Methods**: Comprehensive clinical, histopathological, immunohistochemical and molecular characterisation of human post-mortem brain tissue. Case identification from the Spanish and Italian CJD surveillance Centres.

**Results**: Six sCJD cases with a new phenotype were identified (4 in Catalonia and 2 in Italy). Patients carried the methionine (M)-valine (V) genotype at PRNP codon 129 combined with PrPSc type 1. The clinical and neuropathological profile was reminiscent of the VV1 sCJD subtype rather than the typical MM1/MV1. Patients had relatively long disease duration (mean of 20.5 vs. 3.5 months of MM1/MV1 patients) and typical MRI findings with cortical hyperintensities, but no typical EEG features for CJD. Histological findings included prominent spongiform change with larger vacuoles than those observed in sCJD MM1/MV1 (as in VV1), and a lesion profile with prominent cortical and striatal involvement. The PrPSc deposition pattern was characterized by faint synaptic deposits that contrasted with the prominent spongiform change (as happens in VV1) combined with coarse, patch-like deposits in the cerebellar molecular layer, which is unusual for VV1. Western blot analysis revealed a PrPSc type 1 profile with physicochemical properties similar to those of the type 1 protein linked to the VV1 sCJD subtype.

**Conclusions**: We have identified a novel subtype of sCJD affecting mainly elderly patients with a unique clinicopathological phenotype likely representing the V1 sCJD strain propagation in the 129 MV host genotype.

**Funded by**: The Austrian Reference Center for Human Prion diseases (OERPE) is funded by the Austrian Federal Ministry of Social Affairs, Health, Care and Consumer Protection. This study was supported by a grant to E.G. from the ‘Medizinisch-Wissenschaftlichen Fonds des Burgermeisters der Stadt Wien’, and from the Italian Ministry of Health (Ricerca corrente) S.K. holds a grant from the ‘Hochschuljubiläumsfondsder Stadt Wien’.

**Grant number**: Medizinisch-Wissenschaftlichen Fonds des Burgermeisters der Stadt Wien (project no. 18,097), Hochschuljubiläumsfondsder Stadt Wien (project no. H-283459/2019).

**Acknowledgement**: We are indebted to the Neurological Tissue Bank of the IDIBAPS Biobank, the Prion Disease Tissue collection at IRCCS-ISNB, and the Neurobiobank of the Medical University of Vienna for sample and data procurement. We particularly thank brain donors and their families for generous tissue donations for research purposes. Without them, this research would not have been possible. We also thank the Austrian Ministry of Social Affairs, Health, Care, and Consumer Protection for supporting the Austrian Reference Center for Human Prion Diseases (OERPE). We are also grateful to Sara Charif, Veronica Santiago (IDIBAPS Biobank, Barcelona), Benedetta Carlà (IRCCS, ISNB), and Judith Ludwig (Medical University of Vienna) for excellent technical assistance.

**New anti-prion compounds able to reduce the pathologic aggregation of alpha-synuclein and PABPN1 and to lessen ER stress**

L. Gentile^a,*^, PH. Nguyen^a,*,#^, M. Sinane^a,#^, J. Dhiab^b,#^, K. Nguyen^a^, C. Page^a^, P. Conan^a^, A. Nasir^a^, G. Friocourt^a^, C. Trollet^b^, H. Galons^c^, N. Oumata^c,#^, and C. Voisset^a,#^

^a^Inserm, Univ Brest, EFS, UMR 1078, GGB, Brest, France; ^b^Inserm, Institut de Myologie, Centre de Recherche en Myologie, Sorbonne Université, Paris, France; ^c^Laboratoire de chimie organique 2, Inserm U1022, Université Paris Descartes, Paris, France ^*^Present address: Host Parasite Interactions Section, Laboratory of Intracellular Parasites, NIAID, NIH, Rocky Mountain Laboratories, Hamilton, MT, USA ^#^These authors contributed equally to this work.

**Aims**: In an effort to identify compounds able to reduce the adverse effects of the pathogenic aggregation of proteins responsible for protein misfolding diseases (PMDs) such as Prion diseases, a series of 26 original compounds were synthetized and screened for their activity against PrP^Sc^Prion. The 5 best anti-prion compounds identified were further challenged for their capacity to reduce misfolded protein aggregation and endoplasmic reticulum (ER) stress, two major hallmarks of PMDs.

**Material and Methods**: Anti-prion activity was evaluated using MovS6 cells, PABPN1 protein aggregation was evaluated using Ala17 OPMD muscle cells, CHO-KI and PC12 differentiated cells were used to evaluate cytoprotection against ER stress.

**Results**: 4 out of 5 anti-prion compounds were able to reduce the toxic aggregation of WT and mutant A53T alpha-synuclein proteins in yeast-based models. 3 out of 5 anti-prion compounds were able to reduce the pathologic nuclear aggregation of PABPN1 protein in a cellular model of oculopharyngeal muscular dystrophy, and 5 out of 5 were shown to protect CHO cells and neuronal cells from ER stress-induced cell death.

**Conclusions**: This study showed that molecules selected for their anti-prion activity also possess capacities to reduce the pathogenic aggregation of other proteins, decrease the adverse effects of protein aggregation like ER stress and promote cell survival.

**Funded by**: Fondation NRJ, CECAP, Fondation Grand Ouest, Inserm, MESR, association G. Saleun.

**Acknowledgement**: Fondation NRJ, CECAP, Fondation Grand Ouest, Inserm

**In vivo assessment of Lewy body copathology in idiopathic normal pressure hydrocephalus: Prevalence and associations with clinical features and surgery outcome**

Giulia Giannini^a,b^, Sofia Dellavalle^a^, Simone Baiardi^a,c^, Corrado Zenesini^a^, Sabina Cevoli^a,b^, Nils Danner^d,e^, Henna-Kaisa Jyrkkänen^d, e^, Marcello Rossi^a^, Barbara Polischi^a^, Corinne Quadalti^a^, Camilla Stefanini^b^, Pietro Cortelli^a,b^, David Milletti^a^, Sanna-Kaisa Herruka^f,g^, Giorgio Palandri^a^, Ville Leinonen^d,e^, and Piero Parchi^a,c^

^a^IRCCS, Istituto delle Scienze Neurologiche di Bologna, Bologna, Italy; ^b^Department of Biomedical and Neuromotor Sciences (DIBINEM), University of Bologna, Bologna, Italy; ^c^Department of Experimental, Diagnostic and Specialty Medicine (DIMES), University of Bologna, Bologna, Italy; ^d^Department of Neurosurgery, Kuopio University Hospital, Kuopio, Finland; ^e^Neurosurgery, Institute of Clinical Medicine, University of Eastern Finland, Kuopio, Finland; ^f^Department of Neurology, Kuopio University Hospital, FI-70029 Kuopio, Finland; ^g^Neurology, Institute of Clinical Medicine, University of Eastern Finland, FI-70210 Kuopio, Finland

**Aims**: Idiopathic normal pressure hydrocephalus (iNPH) is a clinical-radiological syndrome of elderly individuals characterized by gait disturbances, cognitive decline and urinary dysfunction. Given the overlapping age distribution and clinical phenotypes with common age-related neurodegenerative disorders such as Alzheimer’s disease (AD) and Lewy body disease (LBD), exploring the influence of these co-pathologies on clinical features and surgery outcome represents a crucial step. The recent introduction of the alpha-synuclein (α-syn) real-time quaking-induced conversion (RT-QuIC) assay, an ultrasensitive technique that detects misfolded α-syn using an amplification strategy, has recently provided a robust in vivo biomarker for Lewy body (LB) pathology.

**Material and Methods**: We measured CSF α-syn seeding activity by RT-QuIC in 293 iNPH patients from two independent cohorts from Italy and Finland. In participants who showed a positive α-syn seeding profile, we evaluated the number of positive replicates, the peak of the fluorescence response (Imax), and the lag phase (time to threshold). We also compared the results obtained in iNPH participants with those of two control groups. They included 89 age-matched individuals who died of Creutzfeldt-Jakob disease (CJD), as representative of the general population, and 45 patients with dementia of Lewy bodies (DLB), representing a full-blown LBD. Finally, in the Italian cohort, we investigated the association between positive α-syn RT-QuIC result, baseline clinical features, and shunt surgery outcome at six months.

**Results**: Sixty (20.5%) iNPH patients showed α-syn seeding activity with no significant difference of frequency between cohorts. In contrast, the prevalence observed in CJD was only 6.7% (p = 0.002). The comparison of the number of positive replicates and the RT-QuIC kinetic parameters between the iNPH and DLB groups showed reduced seeding activity in the former group (fewer positive replicates, longer lag phase, and lower Imax (all p < 0.001)). In the Italian cohort, α-syn RT-QuIC positivity was associated with higher axial and upper limb rigidity (p = 0.003 and p = 0.011, respectively) and lower MMSEc scores (p = 0.003) at baseline. There were no significant associations between positive CSF α-syn seeding activity and surgical outcome at six months.

**Conclusions**: LB pathology affects many iNPH patients and contributes to clinical phenotype by worsening cognitive and motor (parkinsonian type) impairment but does not significantly influence the surgical outcome at six months. The comparison with the DLB cohort indicates that LB pathology is, on average, less severe in the iNPH than in the DLB. The effect of LB pathology on the clinical benefit after surgery over a more extended period remains to be determined.

**Funded by**: Grants from the Italian Ministry of Health (‘Ricerca corrente’). Kuopio University Hospital VTR Fund, Sigrid Juselius Foundation and Academy of Finland

**Acknowledgement**: We wish to thank the members of the BOLOGNA PRO-HYDRO Study Group (Raffaele Agati, Luca Albini-Riccioli, Giovanna Calandra-Buonaura, Lorenzo Chiari, Emanuele La Corte, Elena Magelli, Paolo Mantovani, Alessandro Pirina, Vito Antonio Piserchia, Luciano Romano, Michelangelo Stanzani-Maserati, Luisa Sambati), and Marita Parviainen for their contributions.

**Modeling Creutzfeldt-Jakob Disease using human iPSC-derived Neurons and Brain Organoids**

Aldana D. Gojanovich^a#^, Nhat T.T. Le^b#^, Robert C.C. Mercer^b#^, Alice Anane^c^, Seonmi Park^a^, Bei Wu^b^, David A. Harris^b^ and Gustavo Mostoslavsky^a,d,e^

^a^Center for Regenerative Medicine of Boston University and Boston Medical Center, Boston, Massachusetts, USA; ^b^Boston University School of Medicine, Department of Biochemistry, Boston, Massachusetts, USA; ^c^Creutzfeldt-Jakob disease Foundation, Israel; ^d^Boston University School of Medicine, Department of Microbiology, Boston, Massachusetts, USA; ^e^Boston University School of Medicine, Department of Medicine Boston, Massachusetts, USA #equal contributions

**Aims**: To develop a new model of hereditary Creutzfeldt-Jakob Disease (CJD) using induced Pluripotent Stem Cells (iPSC) from a cohort of E200K CJD individuals, both carriers and non-carriers and their differentiated neuronal, astrocyte and brain organoid progeny to characterize the role of mutant prions in neural injury.

**Material and Methods**: We report the establishment of the largest CJD E200K-specific iPSC library and their differentiation towards cortical neurons to characterize the mutant PrP. Samples for reprogramming were obtained from 22 individuals of a large family including carriers and non-carriers of the E200K mutation. Some of these iPSC lines were selected for differentiation into pyramidal cortical neurons, a brain region highly affected by this mutation as well as astrocytes and brain organoids. Utilizing CRISPR-based gene editing we generated isogenic corrected vs non-corrected lines. We performed the first ever sc-RNA-Seq analysis comparing the corrected vs mutant iPSC-derived brain organoids.

**Results**: By analyzing the biochemical properties of PrP and Tau protein, we found that the E200K hiPSC-derived neurons accumulate pathological forms of PrP which co-localize with paired helical filaments of tau protein. At the postsynaptic site, NMDR and PSD95 protein colocalization is disrupted in neurons expressing E200K PrP. Besides, through differentiation of these iPSC lines into brain organoids we were able to obtain a stable 3D platform for disease modeling and characterize their transcriptional signature at single cell resolution.

**Conclusions**: Our study shows, for the first time, that hiPSC-derived neurons and brain organoids expressing endogenous levels of mutant PrP can model certain aspects of human prion disease, offering a powerful platform for investigating subtype pathologies and testing putative therapeutics.

**Funded by**: NIH

**Grant number**: R21NS111499-01

**Structure-defined Aβ polymorphs promote different pathological changes in susceptible mice**

Ruben Gomez-Gutierrez^a,b^, Ujjayini Gosh^c^, Katherine Do^a^, Wai-Ming Yau^c^, Nazaret Gamez^a,b^, Carlos Kramm^a^, Hamid Shirani^d^, Jonathan Schulz^a^, Antonia Gutierrez^b,f^, Peter R. Nilsson^d^, Robert Tycko^c^*, Claudio Soto^a^*, and Rodrigo Morales^a,f^*

^a^Department of Neurology. The University of Texas Health Science Center at Houston. Houston, Texas, USA; ^b^Dpto. Biología Celular, Genética y Fisiología, Instituto de Investigación Biomédica de Málaga-IBIMA, Facultad de Ciencias, Universidad de Málaga. Málaga, Spain; ^c^Laboratory of Chemical Physics, National Institute of Diabetes and Digestive and Kidney Diseases, National Institutes of Health. Bethesda, Maryland, USA; ^d^Department of Physics, Chemistry and Biology, Linköping University, Linköping, Sweden; ^e^Centro de Investigación Biomédica en Red sobre Enfermedades Neurodegenerativas (CIBERNED). Madrid, Spain; ^f^Centro Integrativo de Biologia y Quimica Aplicada (CIBQA). Universidad Bernardo O’Higgins. Santiago, Chile.

**Aims**: Compelling evidence in humans and experimental rodents suggest that Alzheimer’s disease (AD) -associated Aβ exists in a variety of conformational strains. Unfortunately, current research has not addressed the biological significance of Aβ strain variation in AD. This issue acquires additional relevance considering that mixtures of Aβ strains seems to exist in the brains of patients. Aβ strain variation may explain the pathological and clinical differences observed among people afflicted by AD.

**Material and Methods**: Here, we used brain-derived and synthetic Aβ strains to assess for potential differences in propagation and pathological manifestations.

**Results**: In a first set of experiments, two synthetic-Aβ40 strains (2F and 3F) that have been thoroughly studied for their structural motifs, were biochemically characterized and injected in the brains of 50 days old Tg2576 mice. We assessed prion-like transmission of these materials by analyzing Aβ deposition 250 days later. A second set of experiments involved the administration of AD brain homogenates from individuals displaying diverse amyloid pathology. These human brain extracts were intra-cerebrally injected into 30 days old APP/PS1 mice that were sacrificed 150 days later. Pathological differences in both experiments were found at different levels, including the type and anatomical distribution of the aggregates, Aβ40/Aβ42 ratios, reactivity of amyloid deposits to dyes able to discriminate among misfolded protein conformations, among others. Importantly, differences in astro- and micro-glial activation were also observed.

**Conclusions**: Our data support the concept and biological relevance of conformational strain variation in non-prion protein misfolding disorders. Our findings may help to identify the most deleterious particles responsible for AD and design conformation-specific strategies for diagnosis and treatment.

**Funded by: NIH/NIA and Alzheimer’s Association**

**Grant number**: R56AG061878, RF1AG059321, and AARGD-18-566,576

**Cattle with the EK211 *PRNP* polymorphism are susceptible to the H-type bovine spongiform encephalopathy agent from either E211K or wild type donors after oronasal inoculation**

Justin J. Greenlee^a^, Eric D. Cassmann^a^, S. Jo Moore^a,b^, and M. Heather West Greenlee^c^

^a^Virus and Prion Research Unit, National Animal Disease Center, ARS, United States Department of Agriculture, Ames, IA, USA; ^b^Oak Ridge Institute for Science and Education (ORISE), U.S. Department of Energy, Oak Ridge, TN, US; ^c^Department of Biomedical Sciences, Iowa State University College of Veterinary Medicine, Ames, IA, US

**Aims**: In 2006, a case of H-type bovine spongiform encephalopathy (H-BSE) was reported in a cow with a previously unreported prion protein polymorphism (E211K). The E211K polymorphism is heritable and homologous to the E200K mutation in humans that is the most frequent *PRNP* mutation associated with familial Creutzfeldt-Jakob disease. Although the prevalence of the E211K polymorphism is low, cattle carrying the K211 allele develop H-type BSE with a rapid onset after experimental inoculation by the intracranial route. The purpose of this study was to investigate whether the agents of H-type BSE or H-type BSE associated with the E211K polymorphism transmit to wild type cattle or cattle with the K211 allele after oronasal exposure.

**Material and Methods**: Wild type (EE211) or heterozygous (EK211) cattle were oronasally inoculated with the H-BSE agent from either the US 2004 case (wild type donor; n = 3) or from the US 2006 case with the E211K polymorphism (n = 4). Cattle were observed daily throughout the course of the experiment for the development of clinical signs. When signs were noted, animals were euthanized and necropsied. Cattle were confirmed positive for abnormal BSE prions by enzyme immunoassay (EIA; Idexx HerdChek BSE Ag Test), anti-PrP immunohistochemistry (IHC) on brainstem, and microscopic examination for vacuolation.

**Results**: Three-out-of-four (75%) calves with the EK211 genotype developed clinical signs of H-BSE including inattentiveness, loss of body condition, weakness, ataxia, and muscle fasciculations and were euthanized. Two of the positive EK211 steers received H-BSE US 2004 inoculum (Incubation Period (IP): 59.3 and 72.3 months) while the other positive steer received the E211K H-BSE inoculum (IP: 49.7 months). EIA confirmed that abundant misfolded protein (O.D. 2.57–4.0) in the brainstem, and IHC demonstrated PrP^Sc^throughout the brain. All wild type recipient cattle and a single EK211 steer remained asymptomatic for the duration of the experiment (approximately 7 years post-inoculation) and no abnormal prion protein was detected in these cattle by EIA.

**Conclusions**: This study demonstrates that the H-type BSE agent is transmissible by the oronasal route. Cattle with the EK211 genotype are oronasally susceptible to small doses of the H-BSE agent from either EK211 or EE211 (wild type) donors. Wild-type EE211 cattle remained asymptomatic for the duration of the experiment with this small dose (0.1 g) of inoculum. These results reinforce the need for ongoing surveillance for classical and atypical BSE to minimize the risk of potentially infectious tissues entering the animal or human food chains.

**Funded by**: US Department of Agriculture

**Acknowledgement**: This research was funded in its entirety by congressionally appropriated funds to the United States Department of Agriculture, Agricultural Research Service. The funders of the work did not influence study design, data collection and analysis, decision to publish, or preparation of the manuscript. This research was supported in part by an appointment to the Agricultural Research Service (ARS) Research Participation Program administered by the Oak Ridge Institute for Science and Education (ORISE) through an interagency agreement between the U.S. Department of Energy (DOE) and the U.S. Department of Agriculture (USDA). ORISE is managed by ORAU under DOE contract number DE-SC0014664. All opinions expressed in this paper are the author’s and do not necessarily reflect the policies and views of USDA, ARS, DOE, or ORAU/ORISE.

**Prion Disease in Human Cerebral Organoids**

Bradley R. Groveman^a^, Simote T. Foliaki^a^, Katie Williams^a^, Brent Race^a^, Chase Baune^a^, Gianluigi Zanusso^b^, and Cathryn L. Haigh^b^

^a^Laboratory of Persistent Viral Diseases, Rocky Mountain Laboratories, National Institute for Allergy and Infectious Diseases, National Institutes of Health, Hamilton, Montana, USA; ^b^Department of Neurosciences, Biomedicine and Movement Sciences, University of Verona, Verona, Italy

**Aims**: Human cerebral organoids (COs) are three-dimensional self-organizing cultures of cerebral brain tissue generated from induced pluripotent stem cells (iPSCs). COs have been used to model different aspects of human neurodegenerative diseases such as in Alzheimer’s disease, Parkinson’s disease, and Down’s syndrome dementia. Our recent application of this CO model to the fatal, transmissible, neurodegenerative family of human prion diseases has opened new avenues for study that were previously inaccessible due to limitations in available disease models. Here we further validate and characterize the CO model for human prion disease.

**Material and Methods**: COs were generated and maintained following the Lancaster protocol (Lancaster MA, Knoblich JA (2014) Nat Protoc). After 5 months of maturation, brain homogenates from different prion disease subtypes were diluted into organoid maintenance media and applied to the organoid cultures for 1 week with a 1:1 dilution after 24 hours. Uptake, clearance, and de novo propagation of prion infection were monitored over several months using Real-Time Quaking Induced Conversion (RT-QuIC) assays and western blot analysis. For animal studies organoid homogenates were diluted to 1% in PBBS/FBS and 30 µL were inoculated intracerebrally into Tg66 transgenic mice that overexpress the homologous human 129 M prion protein. The mice were evaluated for clinical, neuropathological, and biochemical evidence of prion infection.

**Results**: Here we show that organoids derived from different iPSC donors with different genetic background, namely at codon 129, can be infected with various subtypes of human brain derived prions and maintain many aspects of the original prion subtype. Pairing the CO model with transgenic mice we have shown that the prions generated within the organoids maintain and propagate the characteristics of the original inoculum.

**Conclusions**: These studies demonstrate and validate the utility of COs to model different aspects of human prion diseases and provide a new platform for investigating the function of the prion protein and its mutants, prion subtype pathologies, and even testing putative therapeutics.

**Funded by**: This research was supported by the Intramural Research Program of the NIH (NIAID)

**Romanian goats’ genetic variability of PRNP gene**

Maria Rodica GURĂU, Elena NEGRU, Teodor IONESCU, Anca Amalia UDRISTE, Petruța CORNEA, and Stelian BĂRĂITĂREANU

University of Agronomic Sciences and Veterinary Medicine of Bucharest, Faculty of Veterinary Medicine, Clinical Sciences, Bucharest, Romania

**Aims**: Genotyping of Romanian goats in order to establish the PRNP polymorphism and the potential impact of genetic selection over goat farming.

**Material and Methods**: Whole blood and root hair samples from Carpathian, French Alpine and Banat White goat breeds were used to DNA extraction by using the PureLink Genomic-DNA Kits. The PRNP gene was amplified using Amplitaq Gold 360-DNA Polymerase-250 U kit and the primers F1 (5`-CATTTATGACCTAGAATGTTTATAGCTGAT-3`) and R1 (5`-TTGAATGAATATTATGTGGCCTCCTTCCAGAC-3`). The sequencing reactions were performed by outsourcing, CEMIA using the Big Dye Terminator Cycle sequencing Kit v3.1 and detected with ABI PRISM 3130 (Applied Biosystems). Sequence alignment and processing was performed using the Mega 5.0 Software and BioEdit.

**Results**: The following alleles were identified in our study: G37G, W102W, T110T, P110T, P110S, G127G, I142I, I142M, T142I, H143H, P143P, R143 R, N146 N, R151R, R154 R, H154 R, P168P, Q168Q, S173S, N173S, R211R, I218I, Q222Q, K222 K, P240P, S240P, P240S, and S240S. The PRNP alleles proved to confers decreased susceptibility to scrapie were rare in some breeds and with a uncreased incidence in other breed.

**Conclusions**: A future genetic selection program seems to have a negative impact over goat farming for some breed so that the selection genetic programs must be adapted to the breed.

**Funded by**: University of Agronomic Sciences and Veterinary Medicine of Bucharest

**Grant number**: 2021–0004/13.07.2021 CaPriRo, within IPC 2021.

**Acknowledgement**:

This work was supported by a grant of the University of Agronomic Sciences and Veterinary Medicine of Bucharest, project number 2021–0004/13.07.2021 CaPriRo, within IPC 2021.

**Germ-line transmission and generation of PRNP mutated cattle using CRISPR-Cas9**

Gyeong-Min Gim^a,b^, Dong-Hyeok Kwon^a,b^, Kyeong-Hyun Uhm^a,b^, Min-Ji Kim^a,b^, Ji-Hyun Park^c^, Kil-Young Song^c^, Won-Wu Lee^c^, Dae-Jin Jung^d^, Dae-Hyun Kim^d,e^, Jun-Koo Yi^d^, and Goo Jang^a,b,c^

^a^Department of Veterinary Clinical Sciences, College of Veterinary Medicine and the Research Institute of Veterinary Science, Seoul National University, Seoul 08826, Republic of Korea; ^b^BK21 Plus program, College of Veterinary Medicine, Seoul National University, Seoul, Republic of Korea; ^c^LARTBio Inc., Seoul, Republic of Korea; ^d^Gyeongsangbukdo Livestock Research Institute, Yeongju, GyeongSang Buk-Do 36,052, Republic of Korea; ^e^Department of Biotechnology, College of Agriculture & Life Science, Hankyong National University, Gyeonggi, Republic of Korea

**Aims**: Even though occurrence of bovine spongiform encephalopathy (BSE) has been decreased, developing bovine models is an important point for basic understanding of prion in cattle. The objective of this study investigated the possibility that a PRNP knockout cattle (F0) were born by microinjecting all-in-one CRISPR-Cas9 DNAs into in vitro fertilized zygotes and its mutation was transmitted into the germ cells and next generation.

**Material and Methods**: All-in-one plasmid including spCas9, GFP and sgRNA for PRNP was constructed and microinjected into in vitro fertilized zygotes. GFP expressing blastocysts were selected and transferred to recipients. Mutation analysis from the tissue of the offspring using T7E1 assay and deep sequencing was carried out. Oocytes and semen from the founder (female and male) were collected and mutation of PRNP was confirmed.

**Results**: A blastocyst was transferred 18 recipients and 7 calves were born as shown. There were PRNP mutation in five calves and transgene integration was confirmed by genomic PCR. The oocytes of a female were collected, fertilized with wild type sperm and developed to pre-implantation stage. Semen from a male were collected, frozen for long-term storage, fertilized with in vitro matured oocytes, and developed to blastocyst. Those blastocysts have PRNP mutation, were transferred into recipients. In six recipients, pregnancy has been going on to date.

**Conclusions**: In conclusion, these data demonstrated that PRNP mutation cattle was born, germline transmitted to next generation and survived to up to date. Those PRNP mutation cattle and their germ cells will be valuable resources for studying the prion disease.

**Funded by**: National Research Foundation of Korea and Seoul National University (SNU) grant

**Grant number**: #2017R1A2B3004972 and **#**550–2,020,005

**Acknowledgement**: We thank the members of the Goo Jang lab for their valuable comments and the National Agricultural Cooperative Federation Bucheon Livestock Market and National livestock institute the technical support.

**Selective breeding for rare PRNP variants in farmed whitetail deer in the management of chronic wasting disease**

Nicholas J. Haley^a^, Rozalyn Donner^b^, Kahla Merrett^b^, Matthew Miller^b^, and Kristen Senior^a^

^a^Department of Microbiology and Immunology, College of Graduate Studies, Midwestern University, Glendale, Arizona USA; ^b^College of Veterinary Medicine, Midwestern University, Glendale, Arizona USA

**Aims**: We sought to eliminate highly susceptible prion (PRNP) genotypes in farmed whitetail deer (WTD) in favor of animals carrying less susceptible PRNP variants, and reduce the prevalence of chronic wasting disease (CWD) on several endemic properties.

**Material and Methods**: The project focused on a farmed deer herd made up of two CWD-negative captive breeding properties and two fully enclosed hunting properties where CWD was endemic – with historical prevalence >50%. Animals were screened for PRNP genotypes using PCR and sequencing. Those carrying alleles coding for 95 H, 96S, and 226 K were selected for breeding. Deer were released at 2 years of age on CWD-endemic properties for progressively longer durations of time and monitored for CWD status when harvested 12–24 months later.

**Results**: The frequency of animals homozygous for the highly susceptible 96 G PRNP allele was reduced to near zero after 4 years of selective breeding, while animals carrying less susceptible alleles increased in frequency. In the first two years of harvest, CWD prevalence on both endemic properties decreased significantly, with only 96 G homozygous animals testing positive for CWD.

**Conclusions**: Deer with rare PRNP variants can be effectively propagated, and this may be useful in reducing CWD prevalence in endemic areas. Though additional work is necessary, selective breeding focusing on PRNP genotype alone may be sufficient to effectively eliminate CWD in farmed WTD populations.

**Funded by**: Midwestern University Start-up Funds

**Grant number**: NA

**Acknowledgement**: We would like to acknowledge the deer farm involved in this project, who would like to remain anonymous.

**Infection of Neuronal Cells by extracellular PrP fibrils**

Hazim A. Halim^a,b^, Juan M. Ribes^b^, Mitali Patel^b^, George Thirlway^b^, John Collinge^b^, and Peter- C. Kloehn^b^

^a^Universiti Sains Islam Malaysia, Persiaran Ilmu, Bandar Baru Nilai, Nilai, Negeri Sembilan, Malaysia; ^b^Medical Research Council Prion Unit at UCL, Institute of Prion Diseases, University College London, London W1W 7FF, UK

**Aims**: Extracellular assemblies of amyloid plaques, composed of disease-associated prion protein (PrP^d^) are a pathological hallmark of prion diseases, but the pathogenicity of such amyloid plaques is less well studied. We generated cell-free assemblies of amyloid PrP^d^scaffolds (PrP^d^scaffolds), an experimental model that mimics extracellular amyloids *in vivo*. PrP^d^scaffolds are formed in the extracellular matrix (ECM) of infected neuronal cells and consist of abundant, micrometer long full-length PrP^d^fibrils which remain immobilized on the surface of culture dishes following decellularization. To address whether infection is established by surface contact or by uptake of PrP^d^fibrils, we analyzed the cellular site of *de novo* formed PrP using myc-*Prnp* expressing reporter cells. We further silenced *Prnp* in reporter cells prior to contact with PrP^d^scaffolds to ascertain that infection can be blocked by gene perturbation, a prerequisite for investigating the cellular underpinning of infection.

**Material and Methods**: Decellularized ECM was prepared by culturing persistently prion-infected or uninfected cells for 5 days, followed by trituration of cells under hypoosmolar conditions. Uninfected myc-*Prnp* expressing reporter cells were plated onto PrP^d^scaffolds and infection of cells was assessed by prion titer determination and by labeling of PrP^d^aggregates, respectively. For gene perturbation experiments, reporter cells were transfected with pools of siRNA against selected gene targets.

**Results**: Infection of reporter cells, plated onto PrP^d^scaffolds is unexpectedly fast and efficient. At two weeks after exposure of reporter cells with cell-free PrP^d^scaffolds, infectious titers were equivalent to 2,440 LD50 units per well of a 96-well plate, when calculated from infections with titered prion-containing brain homogenates. Mice inoculated with 9 × 10^5^infected reporter cells succumbed to disease at 171 days post inoculation, only about 10 days later than an equivalent amount of persistently prion-infected cells. Transient transcriptional silencing of *Prnp* blocks infection of reporter cells by at least 80%, when compared with non-targeting controls, confirming that the established reporter cell assay is amenable to a gene perturbation.

**Conclusions**: PrP^d^scaffolds are an experimental tool to study how neuronal cells are infected by contact with PrP^d^amyloid. Unexpectedly, extracellular PrP^d^scaffolds are highly infectious. Blocking infection of reporter cells by *Prnp* silencing confirms that this model can be used to investigate how neuronal cells are infected by PrP^d^amyloid.

**Funded by**: Ministry of Higher Education of Malaysia and UK Medical Research Council (MC_UU_00024/4)

**Acknowledgement**: We would like to thank Christian Schmidt, Parvin Ahmed and George Thirlway for conducting the Automated Scrapie Cell Assays.

**Transmission of prion infectivity from CWD-infected macaque tissues to rodent models demonstrates the zoonotic potential of chronic wasting disease.**

Samia Hannaoui^a^, Ginny Cheng^a^, Wiebke Wemheuer^b^, Walter J. Schulz-Schaeffer^b^, Sabine Gilch^a^, and Hermann M. Schätzl^a^

^a^Department of Comparative Biology and Experimental Medicine, Faculty of Veterinary Medicine & Hotchkiss Brain Institute; University of Calgary, Calgary, Canada; ^b^Institute of Neuropathology, Medical Faculty, Saarland University, Homburg/Saar, Germany

**Aims**: Chronic wasting disease (CWD) is a prion disease of cervids. Its rapid geographic expansion, shedding of infectivity and persistence in the environment for many years are of concern for humans. Here, we provide the first evidence by transmission experiments to different transgenic mouse models and bank voles that *Cynomolgus macaques* inoculated via different routes with CWD-positive cervid tissues harbor infectious prions that elicit clinical disease in rodents.

**Material and Methods**: We used tissue materials from macaques inoculated with CWD to inoculate transgenic mice overexpressing cervid PrP^C^followed by transmission into bank voles. We used RT-QuIC, immunoblot and PET blot analysis to assess brains, spinal cords, and tissues of the gastrointestinal tract (GIT) for the presence of prions.

**Results**: Our results show that of the macaque materials that induced clinical disease in transgenic mice,73% were from the CNS (46% spinal cord and 27% brain), and 27% were from the spleen, although attack rates were low around 20%. Clinical mice did not display PK-resistant PrP^Sc^(PrP^res^) in immunoblot, but showed low-levels of prion seeding activity. Transmission into bank voles from clinical transgenic mice led to a 100% attack rate with typical PrP^res^signature in immunoblot, which was different from that of voles inoculated directly with CWD or scrapie prions. High-level prion seeding activity in brain and spinal cord and PrP^res^deposition in the brain were present. Remarkably, we also found prion seeding activity in GIT tissues of inoculated voles. Second passage in bank voles led to a 100% attack rate in voles inoculated with brain, spinal cord and small intestine material from first round animals, with PrP^res^in immunoblot, prion seeding activity, and PrP^res^deposition in the brain. Shortened survival times indicate adaptation in the new host. This also shows that prions detected in GIT tissues are infectious and transmissible. Transmission of brain material from sick voles back to cervidized mice revealed transmission in these mice with a 100% attack rate, and interestingly, with different biochemical signature and distribution in the brain.

**Conclusions**: Our findings demonstrate that macaques, considered the best model for the zoonotic potential of prions, were infected upon CWD challenge, including oral one. The disease manifested as atypical in macaques and transgenic mice, but with infectivity present at all times, as unveiled in the bank vole model with an unusual tissue tropism.

**Funded by**: The National Institutes of Health, USA, and the Alberta Prion Research Institute/Alberta Innovates Canada.

**Grant number**: 1R01NS121016-01; 201,600,023

**Acknowledgement**: We thank Umberto Agrimi, Istituto Superiore di Sanità, Rome, Italy, and Michael Beekes, Robert-Koch Institute Berlin, Germany, for providing the bank vole model. We thank the University of Calgary animal facility staff and Dr. Stephanie Anderson for animal care.

**Transmission of Cervid Prions to Humanized Mice Demonstrates the Zoonotic Potential of CWD**

Samia Hannaoui^a^, Irina Zemlyankina^a^, Sheng Chun Chang^a^, Maria Immaculata Arifin^a^, Vincent Béringue^b^, Debbie McKenzie^c^, Hermann M. Schatzl^a^, and Sabine Gilch^a^

^a^Department of Comparative Biology and Experimental Medicine, Faculty of Veterinary Medicine; Hotchkiss Brain Institute; University of Calgary, Calgary, Canada; ^b^Université Paris-Saclay, INRAE, UVSQ, VIM, Jouy-en-Josas, France; ^c^Department of Biological Sciences, Center for Prions and Protein Folding Diseases, University of Alberta, Edmonton, Canada

**Aims**: Chronic wasting disease (CWD), a prion disease of cervids, spreads efficiently among wild and farmed animals. Potential transmission to humans of CWD is a growing concern due to its increasing prevalence. Here, we aimed to determine the zoonotic potential of CWD using a mouse model for human prion diseases.

**Material and Methods**: Transgenic mice overexpressing human PrP^C^homozygous for methionine at codon 129 (tg650) were inoculated intracerebrally with brain homogenates of white-tailed deer infected with Wisc-1/CWD1 or 116AG CWD strains. Mice were monitored for clinical signs and were euthanized at terminal disease. Brains were tested by RT-QuIC, western blot upon PK digestion, and immunohistochemistry; fecal homogenates were analyzed by RT-QuIC. Brain/spinal cord and fecal homogenates of CWD-inoculated tg650 mice were inoculated into tg650 mice or bank voles. Brain homogenates of bank voles inoculated with fecal homogenates of CWD-infected tg650 mice were used for second passage in bank voles.

**Results**: Here, we provide the strongest evidence supporting the zoonotic potential of CWD prions, and their possible phenotype in humans. Inoculation of mice expressing human PrP^C^with deer CWD isolates (strains Wisc-1 and 116AG) resulted in atypical clinical manifestations in > 75% of the mice, with myoclonus as leading clinical sign. Most of tg650 brain homogenates were positive for seeding activity in RT-QuIC. Clinical disease and presentation was transmissible to tg650 mice and bank voles. Intriguingly, protease-resistant PrP in the brain of tg650 mice resembled that found in a familial human prion disease and was transmissible upon passage. Abnormal PrP aggregates upon infection with Wisc-1 were detectable in thalamus, hypothalamus, and midbrain/pons regions.

Unprecedented in human prion disease, feces of CWD-inoculated tg650 mice harbored prion seeding activity and infectious prions, as shown by inoculation of bank voles and tg650 with fecal homogenates.

**Conclusions**: This is the first evidence that CWD can infect humans and cause disease with a distinctive clinical presentation, signature, and tropism, which might be transmissible between humans while current diagnostic assays might fail to detect it. These findings have major implications for public health and CWD-management.

**Funded by**: We are grateful for financial support from the Natural Sciences and Engineering Research Council of Canada, the National Institutes of Health, Genome Canada, and the Alberta Prion Research Institute. SG is supported by the Canada Research Chairs program.

**Acknowledgement**: We thank Dr. Trent Bollinger, WCVM, University of Saskatchewan, Saskatoon, Canada, for providing brain tissue from the WTD-116AG isolate, Dr. Stéphane Haïk, ICM, Paris, France, for providing brain tissue from vCJD and sCJD cases, and Dr. Umberto Agrimi, Istituto Superiore di Sanità, Italy, for the bank vole model. We thank animal facility staff for animal care, Dr. Stephanie Anderson for veterinary oversight, and Yo-Ching Cheng for preparing recombinant PrP substrates. Thank you to Dr. Stephanie Booth and Jennifer Myskiw, Public Health Agency of Canada, Canada.

**No evidence of uptake or propagation of reindeer CWD prions in environmentally exposed sheep**

Erez Harpaz^a^, ״yvind Salvesen^a^, Geir Rune Rauset^b^, Aqsa Mahmood^a^, Linh Tran^c^, Bjרrnar Ytrehus^b,d^, Sylvie Lafond Benestad^c^, Michael Andreas Tranulis^e^, Arild Espenes^e^, and Cecilie Ersdal^a^

^a^Department of Production Animal Clinical Sciences, Faculty of Veterinary Medicine, Norwegian University of Life Sciences, Sandnes, Norway; ^b^Norwegian Institute for Nature Research (NINA), Trondheim, Norway; ^c^Norwegian Veterinary Institutes, Norway; ^d^Department of Biomedical Science and Veterinary Public Health, Swedish University of Agricultural Sciences, Uppsala, Sweden; ^e^Department of Preclinical Sciences and Pathology, Faculty of Veterinary Medicine, Norwegian University of Life Sciences, Norway.

**Background and aim**: Chronic wasting disease (CWD) is a prion disease of cervids originally reported in North America in the 1960s. CWD was diagnosed in 2016 in a wild reindeer in Nordfjella mountain area in Norway, the first rapport of natural infection in reindeer, and the first reported CWD in Europe. Detection of more cases in the same area led to the complete culling and testing of this partially confined reindeer population of about 2400 animals. Of these, 19 CWD positive reindeer were identified. The affected area is extensively used as a summer pasture for sheep, with hundreds of installed mineral licks that attract both sheep and cervids. This overlap in area use raised concerns for cross-species prion transmission between reindeer and sheep. The aim of the study was to investigate potential uptake and propagation of CWD prions in sheep under natural conditions, and to investigate spatial and time-relevant overlaps between reindeer and sheep.

**Material and methods**: Global positioning system (GPS) data from sheep and reindeer was collected and analyzed, including tracking of one of the CWD positive reindeer. Since prions can accumulate in lymphoid follicles following oral uptake, samples of gut-associated lymphoid tissue (GALT) from 425 lambs and 78 adult sheep, which had grazed in the region during the relevant timeframe, were analyzed for the presence of prions. The recto-anal mucosa associated lymphoid tissue (RAMALT) from all the animals were examined by immunohistochemistry (IHC) and enzyme-linked immunosorbent assay (ELISA), and the ileal Peyer’s patch (IPP) from a subsample of 37 lambs were examined by IHC, for the detection of prions. All samples were analyzed histologically with respect to lymphoid follicles.

**Results**: GPS data showed an overlap in area use between the infected reindeer herd and the sheep. In addition, the GPS positions of an infected reindeer and some of the sampled sheep showed temporospatial overlap. No prions were detected in the 12,746 lymphoid follicles found in the GALT of all the investigated sheep. The mean number of lymphoid follicles was 22.6 for RAMALT and 37.8 for IPP.

**Conclusion**: The absence of prions in the GALT of sheep that have shared pasture with CWD-infected reindeer, may suggest that transmission of this novel CWD strain to sheep does not easily occur under the conditions found in these mountains. We also document that the lymphoid follicle rich RAMALT could be a useful tool to screen for prions in sheep.

**Funded by**: The Fund for Research Fees of Agricultural Products and the Agricultural Agreement Research Fund, canalized through the Research Council of Norway

**Grant number**: 294,885

**mGluR5 inhibition delays cognitive decline and incubation time in a mouse model for prion disease, but only if applied before onset of symptoms**

Kristin Hartmann^a^, Christiane Hartmann^a^, Cheng Fang^b^, David Harris^b^, Diego Sepulveda-Falla^a^, Markus Glatzel^a^, and Susanne Krasemann^a^

^a^Institute of Neuropathology, University Medical Center Hamburg-Eppendorf, Hamburg, Germany; ^b^Department of Biochemistry, Boston University School of Medicine, Boston, Massachusetts, USA

**Aims**: Toxic signaling of oligomeric protein species via binding to the cellular prion protein (PrP) have been implicated in a variety of neurodegenerative diseases including Alzheimer’s (AD) and Parkinson disease (PD). Metabotropic glutamate receptor 5 (mGluR5) is one of the receptors that were identified as PrP signaling partner and pharmacologic inhibition of mGluR5 was successfully shown to improve cognitive performance in mouse models of AD and LTP impairment in *in vitro* models for PD. While broad inhibition of mGlu-receptors led to prolonged survival in a mouse prion model, when delivered directly with prion infection, the sensitive time frame for therapeutic intervention during disease progression was not determined. Here, we investigate the ability of the highly specific mGluR5 inhibitor CTEP to stall disease when applied at different time points during disease progression.

**Material and Methods**: We investigated the impact of chronic oral treatment with the selective mGluR5 inhibitor CTEP in a C57Bl/6 mouse model of prion diseases after cerebral infection with RML5.0. Mice were adapted to the chow and treatment was started at different time points post infection. Control chow did not contain CTEP but was otherwise prepared identically. Animals were monitored daily and behavior of mice was documented. One cohort was taken at a preclinical time point. While the others were taken at clinical disease, matching animals were also taken from all groups for better comparison. Brains of all cohorts were examined in detail via Western Blot, immunohistochemical staining, and expression analysis. Primary neurons were used to dissect effects on synaptotoxicity of mGluR5 inhibition by CTEP after treatment with amyloid versus PrP^Sc^.

**Results**: Whereas treatment starting before onset of symptoms resulted in significant improvement in survival and cognitive performance, the application of CTEP after onset of symptoms did not show such effects. Of note, the amount of misfolded PrP^Sc^in brain tissue as determined by Western Blot was comparable in all groups. Interestingly, subacute treatment of primary neurons with CTEP blocked amyloid-induced, but not PrP^Sc^-mediated synaptotoxicity.

**Conclusions**: Oral treatment with CTEP to inhibit the mGluR5 significantly prolonged and improved survival in a prion disease mouse model. However, treatment must start before the onset of clinical symptoms.

**Prion genotypes in Icelandic scrapie flocks: The effect of removing rams with aVRQ allele from Icelandic breeding stations**

Eva Hauksdóttir, and Stefanía Thorgeirsdóttir

Department of Virology and molecular biology, The Institute for Experimental Pathology at Keldur, University of Iceland, Reykjavík, Iceland

**Aims**: Iceland does not have a fixed protocol to reduce the number of risk associated VRQ genotypes in the Icelandic sheep population. However, since 2008, there have been no VRQ-allele-carrying rams in the Icelandic sheep breeding stations. This study assessed the effect that removal of VRQ-allele-carrying rams from the breeding stations has had on the genetic variance of *PRNP*.

**Material and Methods**: The assessment was performed by comparing classical scrapie flocks, as well as their clinical suspect index samples, from the years 2010–2019(experimental group, n = 1450 and 10, respectively) to 1998–2007 (control group, n = 1081 and 32, respectively). The process for genotyping samples was DNA isolation, PCR, RFLP and electrophoresis. The age of the index cases was obtained from their ear tags. When comparing the distribution of *PRNP* genotypes between the groups, for both the flocks and the index samples, a Chi square test was used, and a Mann-Whitney test was used for age comparison.

**Results**: A significant difference was detected in the frequency of the corresponding amino acids at codons 136 and 154 (p < 0.0001 for both) in the classical scrapie flocks. However, there was no difference when comparing codon 136, in the clinical suspect index samples, between the groups (p = 0.9784), and no polymorphism was found at codon 154. There was no difference in the comparison of the age of index samples (p = 0.2808) between the experimental group (median: 36 months; range: 24–48 months) and the control group (33 months; 12–108 months).

**Conclusions**: The results imply that the removal of VRQ-allele-carrying rams from the breeding stations has brought about a change in the genetic variance of *PRNP*, as there has been a decrease in the frequency of the risk genotypes that contain VRQ alleles, in classical scrapie flocks. As there was no difference in the genetic variance of *PRNP* or age in the clinical suspect, classical scrapie index samples, we hypothesize that not enough time has passed for the full effect of this action to be noticeable.

**Acknowledgement**: We thank Ástríður Pálsdóttir for her contribution in initiating the *PRNP* genotyping studies in sheep at Keldur in 1995.

**Adipose-Derived Mesenchymal Stromal Cells Decrease Prion-Induced Glial Inflammation**

Arielle Hay^a,b^, Tanner Murphy^a,b,c^, Katriana Popichak^a,b^, Sean Boland^a,b^, Mark D. Zabel^a,b^, and Julie A. Moreno^a, b^

^a^Prion Research Center, Colorado State University, Fort Collins, CO, USA; ^b^Department of Microbiology, Immunology and Pathology, College of Veterinary Medicine and Biomedical Sciences, Colorado State University, Fort Collins, CO, USA; ^c^Department of Environmental and Radiological Health Sciences, College of Veterinary Medicine and Biomedical Sciences, Colorado State University, Fort Collins, CO, USA

**Aims**: Prion diseases are characterized by the cellular prion protein, PrP^C^, misfolding and aggregating into the infectious prion protein, PrP^Sc^, which leads to neurodegeneration and death. An early pathology of disease is glial inflammation with a shift of resting glial cells to A1 reactive astrocytes and M1 microglia. Few current therapeutics target this stage of disease. The aim of this research is to develop a safe treatment that decreases inflammation and reprograms glials cells to a neuroprotective phenotype. Mesenchymal stromal cells can migrate to sites of inflammation where they produce anti-inflammatory molecules in response to inflammatory signals and damaged tissue. We assessed the therapeutic potential of adipose-derived mesenchymal stromal cells (AdMSCs) in an in vitro and murine model of prion disease.

**Material and Methods**: Primary mixed glia and BV2 microglia were infected with mouse-adapted scrapie and co-cultured with AdMSCs. RNA was isolated and analyzed for markers of prion-induced inflammation and reactive glia. Prion-infected mice received intranasally-delivered AdMSCs twice monthly from 10 weeks post-infection (wpi) to 18 wpi. Behavioral assays, clinical signs and survival of the mice was assessed. Upon sacrifice, brains were analyzed for number of vacuoles and glial cells, inflammatory cytokines and chemokines, and amount of PrP^Sc^. Migration of AdMSCs to prion-infected brains was assessed in an in vitro assay as well as through tracking intranasally-delivered labeled AdMSCs with live animal imaging in a mouse model.

**Results**: AdMSCs migrate toward prion-infected brain homogenate and produce the anti-inflammatory molecules transforming growth factor b (TGFβ) and tumor necrosis factor-stimulated gene 6 (TSG-6). In an in vitro model of prion infection of both primary mixed glia and BV2 microglial cell line, co-culturing with AdMSCs led to a significant decrease in inflammatory cytokines and markers of A1 astrocytes and M1 microglia. This protection against in vitro prion-induced inflammatory responses is independent of PrP^Sc^replication. In a murine scrapie model, AdMSCs migrate to the brain when delivered intranasally. Although intranasal deliveries of AdMSCs did not increase survival or improve behavioral signs in mice, it did reduce the number of microglia and vacuole counts in the brain.

**Conclusions**: These data support a role for AdMSCs as a beneficial therapeutic for decreasing the early onset of glial inflammation and reprogramming glial cells to a protective phenotype.

**Funded by**: Webb-Waring Biomedical Research Award and Murphy Turner Fund

**Acknowledgement**: The authors thank Lab Animal Resources for their animal husbandry. Our funding sources for this manuscript include the Murphy Turner Fund, CSU College of Veterinary Medicine and Biomedical Sciences College Research Council, Peak Serum, and the Boettcher Webb-Waring Biomedical Research award.

**Proteomic analysis of cerebral spinal fluid and plasma from white-tailed deer infected with CWD**

Allen Herbst^a^, Judd M. Aiken^b^, Anthony Ness^c^, and Debbie McKenzie^c^

^a^US Geological Survey National Wildlife Health Center, Madison, United States of America; ^b^Agricultural, Food and Nutritional Sciences, University of Alberta, Edmonton, Canada; ^c^Biological Sciences, University of Alberta, Edmonton, Canada

**Background**: Chronic Wasting Disease (CWD) is a contagious prion disease of cervids that is spreading geographically and increasing in prevalence. The molecules that respond to CWD prion infection in deer are poorly described.

**Aim**: We hypothesized that protein biomarkers may be present in the CSF and plasma of white-tailed deer infected with CWD and that these biomarkers might help describe the host response to disease.

**Material and Methods**: We used Cerebrospinal Fluid (CSF) and plasma from white-tailed deer experimentally infected with CWD. Protein samples were digested with trypsin and labelled with isobaric tags. The labelled peptides were pooled and then fractionated by high pH reverse phase HPLC. The abundance and identity of labelled peptides in the fractions were determined by an additional phase of liquid chromatography coupled to an electrospray ionization mass spectrometer for peptide mass and sequence determination. Differential protein expression analysis was performed to identify proteins deregulated by CWD. Plasma was also collected from elk suspected of infection with CWD at slaughter and assayed by sandwich ELISA.

**Results**: We obtained 2,883,600 peptide spectra from the CSF of control and prion infected deer that mapped to 112,617 peptides with a 95% probability. Using thresholds of 2 peptides per protein and a 99% probability, 1200 proteins were identified for differential analysis. We found 10 proteins upregulated in the CSF greater than 1.5-fold. The upregulated proteins included haptoglobin, serum amyloid A, lipopolysaccharide binding protein and serine protease inhibitors, suggesting an acute-phase response to CWD infection in the CSF.

In the white tailed-deer plasma, we obtained 2,601,930 spectra which mapped to 98,292 peptides with 95% probability. Using thresholds of 2 peptides per protein and a 99% probability, 340 proteins were identified for differential analysis. Haptoglobin, serpin A3-8 and serum amyloid A were significantly up-regulated greater than 1.5-fold. In the elk plasma, analysis by sandwich ELISA found an upregulation of lipopolysaccharide-bind protein.

**Conclusions**: These data indicate that an acute phase response and inflammation is associated with CWD in white-tailed deer. Elk may also elicit an acute phase response to CWD.

**Funded by**: Genome Canada, Genome Alberta, the Alberta Prion Research Institute, Alberta Agriculture and Forestry and the University of Alberta

**Grant number**: Systems Biology and Molecular Ecology of Chronic Wasting Disease

**Susceptibility of ovine bone marrow-derived mesenchymal stem cell spheroids to scrapie prion infection**

Adelaida Hernaiz^a^, Paula Cobeta^a^, Belén Marín^b^, Francisco J. Vázquez^a,c^, Laura García-Mendívil^d^, Juan J. Badiola^b^, Pilar Zaragoza^a^, Laura Ordovás^d^, Rosa Bolea^b^, and Inmaculada Martín-Burriel ^a,b^

^a^Laboratorio de Genética Bioquímica (LAGENBIO), Facultad de Veterinaria, Universidad de Zaragoza-IA2, IIS, Zaragoza, Spain; ^b^Centro de Encefalopatías y Enfermedades Transmisibles Emergentes (CEETE), Facultad de Veterinaria, Universidad de Zaragoza-IA2, IIS, Zaragoza, Spain; ^c^ Departamento de Patología Animal, Facultad de Veterinaria, Universidad de Zaragoza, Zaragoza, Spain; ^d^Biomedical Signal Interpretation and Computational Simulation (BSICoS), Institute of Engineering Research (I3A), University of Zaragoza & Instituto de Investigación Sanitaria (IIS), Zaragoza, Spain.

**Aims:** Cellular in vitro models are essential tools for the study of different pathologies including prion diseases. However, only a few cell lines can be infected and replicate prions, being the majority of them murine lines that show limited susceptibility to certain prion strains due to the species barrier. This problem could be solved using cells from naturally susceptible species. We tried to overcome this problem using mesenchymal stem cells from a naturally susceptible species and with the purpose of creating a cellular ambient more similar to the physiological state, these cells were cultivated in three dimension conditions (spheroids). The aim of this work was to evaluate the viability and susceptibility of ovine bone marrow-derived mesenchymal stem cell (oBM-MSC) spheroids to scrapie prion infection.

**Material and Methods:** Spheroids from oBM-MSCs carrying the ARQ/ARQ genotype were created. Some spheroids were maintained in growth conditions and others were subjected to neurogenic differentiation. To evaluate the viability, a MTT assay was performed in differentiated and undifferentiated spheroids subjected to three conditions: spheroids inoculated with scrapie-infected sheep brain homogenate, spheroids inoculated with non-infected sheep brain homogenate and control spheroids without inoculum. The inoculum was removed after 48h in contact with the spheroids. The viability was studied at three infection times: 2 dpi (days post-inoculation), 5 dpi and 8 dpi. In addition, to study the capacity of spheroids to be infected and replicate prions, differentiated and undifferentiated spheroids were infected with scrapie-infected sheep brain homogenate. The inoculum was also removed after 48 h in contact with the spheroids and the levels of pathological prion protein (PrP^Sc^) were measured by ELISA at three infection times: 2dpi, 5dpi and 8 dpi. The PrP^Sc^ presence was also confirmed by immunocytochemical analysis.

**Results:** The viability results showed the survival of spheroids after inoculation. Regarding to the ELISA assay, the levels of PrP^Sc^ in both undifferentiated and differentiated spheroids decreased initially at 5 dpi but, afterwards, an increase of PrP^Sc^ signal was observed at 8 dpi showing the ability of spheroids to maintain prion infection. This infection capacity was also observed in the immunocytochemical analysis.

**Conclusions:** These results show that oBM-MSC spheroids constitute a useful in vitro cellular model with potential for the study of prion diseases.

**Funded by:** Gobierno de Aragón and the European Social Fund co-financed predoctoral grant Order IIU/2023/2017 and RTI2018-098711-B-I00: Transmission, replication, toxicity and therapeutic targets for prion diseases in cell models and bioassays (BIOPRIONCELL).

**Transcriptomic analysis of scrapie-infected mesenchymal stem cells**

Adelaida Hernaiz^a^, Belén Marín^b^, Francisco J. Vázquez ^a,c^, Juan J. Badiola^b^, Pilar Zaragoza^a^, Rosa Bolea^b^, and Inmaculada Martín-Burriel ^a,b^

^a^Laboratorio de Genética Bioquímica (LAGENBIO), Facultad de Veterinaria, Universidad de Zaragoza-IA2, IIS, Zaragoza, Spain; ^b^Centro de Encefalopatías y Enfermedades Transmisibles Emergentes (CEETE), Facultad de Veterinaria, Universidad de Zaragoza-IA2, IIS, Zaragoza, Spain; ^c^Departamento de Patología Animal, Facultad de Veterinaria, Universidad de Zaragoza, Zaragoza, Spain

**Aims**: Cellular in vitro models have been proven as useful tools to study many aspects of neurodegenerative diseases, including prion diseases. Previous studies have shown that mesenchymal stem cells (MSCs) are susceptible to prion infection and toxicity, decreasing their proliferation potentical. The aim of the present study was to elucidate the molecular pathways and biological routes involved in the prion infection of MSCs.

**Material and Methods**: Ovine bone marrow mesenchymal stem cells carrying the ARQ/ARQ genotype were seeded and cultured in three different conditions: control cells (without inoculum), MSCs infected with positive-scrapie brain inoculum and MSCs infected with negative-scrapie brain inoculum. The inocula were removed after 48 h in contact with the cells and two infection times were studied; 2 dpi (days post-inoculation) corresponding to the time of inocula removal and 4 dpi. Each condition was analyzed per triplicate in each time. Total RNA of cell cultures (n = 18) was purified to perform a RNA sequencing assay with subsequent enrichment and differential expression analyses.

**Results**: The greatest differences regarding to differentially expressed genes (DEGs) were found at 2 dpi comparing cultures infected with scrapie and control brain with a total of 4,183 DEGs, being 2,112 upregulated and 2,071 downregulated. The number of DEGs decreased at 4dpi (1,665 DEG, 826 upregulated and 839 downregulated). Among the DEGs found between positive-infected and negative-infected MSCs at 2 dpi and 4 dpi, only 792 were common between the two infection times. On the other hand, GO enrichment revealed several significant biological processes enriched in positive-infected MSCs in the two infection times such as oxidoreductase activity, cytoplasmic part, structural molecule activity, protein kinase activity and protein phosphorylation. Moreover, KEGG analysis also identified enriched pathways in scrapie-infected MSCs associated with Alzheimer, Parkinson and Huntington diseases, lysosome, ferroptosis and autophagy.

**Conclusions**: Genes differentially regulated in cultures inoculated with scrapie brains at early phases post inoculation were enriched in genes involved in pathways related with Alzheimer disease, Parkinson disease and Hungtinton disease, all of them considered prion-like neurodegenerative diseases. Those genes were also involved in pathways known to be dysregulated in prion diseases like the lysosome pathway, confirming the susceptibility of ovine MSC to prion toxicity and the reproduction of molecular mechanisms occurring in vivo.

**Funded by**: Gobierno de Aragón and the European Social Fund co-financed predoctoral grant Order IIU/2023/2017 and RTI2018-098711-B-I00: Transmission, replication, toxicity and therapeutic targets for prion diseases in cell models and bioassays (BIOPRIONCELL).

**Two new decontamination process effective against the variant- and the sporadic-VV2 CJD prion strains**

L. Herzog^a^, F. Reine^a^, M. Moudjou^a^, H. Rezaei^a^, V. Béringue^a^, and A. Igel^a,b^

^a^VIM, INRA, UVSQ, Université Paris-Saclay, Jouy-en-Josas, France; ^b^FB Product, Torcé viviers en charnie, France

**Aims**: Prions are a class of pathogens with a high risk of transmission that defy standard inactivation and sterilisation processes and therefore require special vigilance. Thanks to the development of prion-detection tools, it has been demonstrated that the high majority of prionicidal process validated at that time against only 263 K hamster prion strain, barely eliminate human prion strain, requiring The European and French Medicine agencies to re-examine their standardized protocol for validation of inactivation process. We previously discussed methodologies and technical issues raised by these new guidelines with respect to the choice of the cell-free assay, the human prion strain type and the nature of the prion-containing biological material.

The two objectives of this work were: i) to developpe a robust protocol which may be served as a standard for human prion decontamination assay and ii) to validate new decontamination process efficient against human prion strains.

**Material and Methods**: To assess effectiveness of decontamination procedures, stainless steel wires were contaminated with vCJD, sCJD-VV2 and 263 K prion-infected brain homogenates. After washing with different prionicidal treatments (TFD Premium, Anilon, and preconized treatments) the residual prion seeding-activity present in the surface of the wire or in the treated-brain homogenate were quantified using relevant bioassay and PMCA.

**Results**: Thanks to our approaches, we are now able to detect with a high sensitivity the presence of vCJD and sCJD-VV2 prion particles attached to a surface by PMCA and by bioassays. Most importantly, we highlighted that all human prion strain does not show the same susceptibility to inactivation treatments. The sCJD-VV2 prion strain appears more resistant to the inactivation process tested than vCJD prion. We demonstrated that the formulation of TFD Premium and the Anilon used in washing machine are completely efficient to inactivate human prion strains tested in our study. We also demonstrated that TFD Premium used in a soaking bath is more efficient than the 1 M sodium hydroxide during one hour.

**Conclusions**: For the first time we validated two new prionicidal formulations against a panel of human prion strains. The use of these formulations in washing machine or in soaking made these processes accessible to all medical’s facilities and laboratories. The French Agency for Medicines and Health Products Safety (ANSM) just validate the use of the TFD premium as a prionicidal process efficient against human prion strain.

**Funded by: FB Product**

**Characterization of miRNA changes in Chronic Wasting Disease in Relation to Developing Early Detection Models**

Nicholas R. Heyer^a^, Mark D. Zabel^a^ and Jason E. Bruemmmer^b^

^a^Microbiology Immunology and Pathology, Colorado State University, Fort Collins, USA; ^b^National Wildlife Research Center, United States Department of Agriculture, Fort Collins, USA

**Aims**: One aim of this project is to find a curated list of the most significant differences in miRNA in a Chronic Wasting Disease (CWD) infected cervids and a negative control to aid in subsequent longitudinal studies in transgenic mice.

**Material and Methods**: Brain homogenizes from 70 animals (35 CWD+, 35 CWD-) will be confirmed for a prion infection via three rounds of PMCA and then a western blot. RNA extraction and then size selection was then performed in order to enrich the sample for the miRNAs that are of interest. After this next generation sequencing was performed, the reads were cleaned with Trimmomatic then mapped using minimap2 to the GCF_019320065.1 genome, as it is the only cervid with a chromosome level assembly in order to remove any possible contaminating reads. The reads then must be remapped to the mouse reference genome (GRCm39) as there are no miRNAs annotated on a cervid genome, and since we will be doing the longitudinal study in transgenic mice. The reads could then be normalized off of the most stable gene across all samples as determined by NormFinder. The normalized data was then input into R, then using the ‘glmnet’ package and using Elastic-Net Logistic regression the best predictive miRNAs were found as determined by finding the maximum value for the lambda penalty that keeps the false discovery rate under 1% in a 20-fold cross-validation test. To test the accuracy of the model we will then generate ROC curves for the model and find an average AUC.

**Expected Results**: Based on a paper by Jessy A. Slota et. al. in 2019 we expect to find at least 21 miRNAs that differ significantly between CWD+ and CWD- samples. Moreover, since we are using the same sample size we also expect that the best predictive model will only include ~6 miRNAs specifically if our results are consistent we expect mmu-miR-185-5p, mmu-miR-500-3p, mmu-miR99a(−5p), mmu-miR-125b-5p, mmu-miR-181c-5p and mmu-miR-181d-5p to be included, the orthologs in mouse of what was found in the previous paper.

**Conclusions**: The results of this study serve to confirm the findings of the previously mentioned paper so that we have a set of punitive miRNA indicators of CWD and a pipeline for testing the effectiveness of them. Subsequently, we will longitudinally assess at transgenic mice inoculated with CWD with regular sequencing of miRNA to see when or if it could be a reliable, early, and non-lethal detection tool for CWD.

**Funded by**: United States Department of Agriculture Cooperative Agreement

**Grant number**: APP-18407

**Knockout Mice for the Sporadic CJD Risk Gene *STX6* are Overtly Healthy, but have Extended Incubation Times to Mouse Prions**

Elizabeth Hill^a^, Emma Jones^a^, Jacqueline Linehan^a^, Sebastian Brandner^b^, Michael Farmer^a^, Thomas Coysh^a^, Emmanuel Asante^a^, Emmanuelle Viré^a^, John Collinge^a^, and Simon Mead^a^

^a^MRC Prion Unit at UCL, UCL Institute of Prion Diseases, Courtauld Building, 33 Cleveland Street, London W1W 7FF, United Kingdom; ^b^Institute of Neurology, UCL, Queen Square House, London, WC1N 3BG

**Background**: *STX6*, which encodes syntaxin-6, a SNARE protein primarily involved in early endosome to *trans*-Golgi network retrograde transport, was recently proposed as a risk gene for sporadic Creutzfeldt-Jakob disease (sCJD). Specifically, increased brain expression of *STX6* may increase susceptibility to prion disease. Interestingly, the underlying molecular mechanisms may be common to other neurodegenerative diseases with syntaxin-6 also being identified as a genetic risk factor for progressive supranuclear palsy and modified protein expression of syntaxin-6 has been causally associated with Alzheimer’s disease.

**Aims**:

1. Explore the effect of syntaxin-6 (*Stx6*) expression on incubation time and neuropathology in prion-infected mice.
2. Perform physiological and behavioural analysis on the *Stx6*^−/−^mice to inform the feasibility of targeting syntaxin-6 as a novel, genetically validated therapeutic target in multiple neurodegenerative diseases.

**Material and Methods**: *Stx6*^−/−^, *Stx6*^±^and *Stx6*^+/+^C57BL/6 N mice were intracerebrally inoculated with RML (n = 20/group) or ME7 (n = 20/group) prions or PBS (n = 5/group). Mice were culled following definite scrapie sick diagnosis and incubation period calculated. Immunohistochemical characterisation of neuronal loss, spongiform vacuolation, astrogliosis, microgliosis and PrP deposition was performed. PBS-inoculated mice were additionally phenotypically characterised in terms of weight and mass of white adipose tissue and underwent preliminary behavioural analysis.

**Results**: Knockout of *Stx6* in mice extended incubation time by 12 days following inoculation with RML or ME7, representing an 8% and 7% increase in survival relative to wildtype controls respectively. Heterozygous *Stx6^±^* mice showed an 8% prolongation of incubation time following RML inoculation, although no extension in survival was seen with ME7-inoculated animals.

Although no differences in neuronal loss, spongiform change or PrP deposition were noted between the different genotypes, a modest increase in astrogliosis was seen in ME7-inoculated *Stx6^−/−^* animals and a variable effect of *Stx6* expression on microgliosis was observed at endpoint.

*Stx6*^−/−^mice are viable and fertile with no gross neurological impairments. Interestingly, investigation of physiological phenotypes in *Stx6*^−/−^mice revealed a subtle metabolic defect and preliminary behavioural analysis suggested minor motivation and motor defects.

**Conclusions**: The statistically significant prolongation in incubation period in mice with reduced *Stx6* expression with two different prion strains provides evidence for a pathological role of *Stx6* expression in prion disease. Lowering syntaxin-6 levels could be explored as a therapeutic strategy, for example, by testing the effects of lowering expression later in the course of prion disease and related neurodegenerative disorders.

**Funded by**: Medical Research Council

**Grant number**: MC_UU_00024/1

**Acknowledgement**: MRC Harwell for the generation of the mouse model. The Transgenics group at the MRC Prion Unit at UCL for assistance with the characterisation of the mouse lines as well as Malin Sandberg, Huda Al-Doujaily and Michael De Oliveira for support with generating inocula and measuring PrP expression. All the staff at our animal facility including Lucy Draper, Ami Woodcock and Thomas Horan for breeding the lines; as well as Nick Kaye, Craig Fitzhugh and Gavin Graham for work in the inoculation experiment. Tamsin Nazari, Fabio Argentina, Florin Pintilli and Helena Costa for help with histology.

**alpha-Synuclein as a surfactant of synaptic condensates**

Christian Hoffmann^a^, Roberto Sansevrino^a^, Johannes Tromm^a^, Gwendolin Schneider^a^, Silvio O. Rizzoli^b^, Tiago Outeiro^c^, and Dragomir Milovanovic^a^

^a^Laboratory of Molecular Neuroscience, German Center for Neurodegenerative Diseases (DZNE), Berlin, Germany; ^b^Institute of Neuro- and Sensory Physiology, University Medical Center Göttingen, Göttingen, Germany; ^c^Department of Experimental Neurodegeneration, Center for Biostructural Imaging of Neurodegeneration, University Medical Center Göttingen, Göttingen, Germany

**Aims**: Compelling evidence suggests that liquid-liquid phase separation is emerging as a major mechanism for organizing macromolecules, particularly proteins with intrinsically disordered regions, in compartments non-limited by a membrane or a scaffold. Hundreds of synaptic vesicles (SVs) form biomolecular condensates through the interaction with synapsins, the highly abundant family of synaptic phosphoproteins. Another major family of disordered proteins at the presynapse includes synucleins, most notably alpha-synuclein. The precise physiological role of alpha-synuclein in synaptic physiology remains elusive, albeit its role has been implicated in nearly all steps of the SV cycle. Here, we aim to dissect the role of alpha-synuclein on the physiological synapsin/SV condensates and pathological inclusions made of synphilin 1, another synaptic protein implicated in Parkisnon’s Disease.

**Material and Methods: T**o determine the effect of α-synuclein on the synapsin phase, we employ the reconstitution approaches using natively purified SVs from rat brains and the heterologous cell system to generate synapsin condensates. We employed a battery of assays to characterize liquid-liquid phase separation properties such as FRAP, live-cell imaging, pharmacological treatments and determining the phase diagram of condensates.

**Results**: We demonstrate that synapsin condensates recruit alpha-synuclein, and while enriched into these synapsin condensates, alpha-synuclein still maintains its high mobility. The presence of SVs enhances the rate of synapsin/ alpha-synuclein condensation, suggesting that SVs act as catalyzers for the formation of synapsin condensates. Notably, at physiological salt and protein concentrations, alpha-synuclein alone cannot trigger the phase separation of SVs. The excess of alpha-synuclein attenuates the kinetics of synapsin/SV condensate formation, indicating that the molar ratio between synapsin and alpha-synuclein is important in assembling the functional condensates of SVs. alpha-Synuclein can be depleted from synapsin condensates by synphilin 1, another intrinsically disordered, scaffold protein at the presynapse implicated in Parkinson’s Disease. Interestingly, synphilin 1 can form fluid condensates by itself, and alpha-synuclein shows the ability to fully wet synphilin condensates in a salt-dependent manner.

**Conclusions**: alpha-Synuclein acts as a surfactant modulating the packing physiological SV condensate in a concentration-dependent manner. Furthermore, alpha-synuclein is wetting the surface of the aberrant synphilin inclusions recapitulating the architecture of Lewy Bodies. Understanding the molecular mechanism of alpha-synuclein interactions at the nerve terminals is crucial for clarifying the pathogenesis of synucleinopathies, where alpha- synuclein, synaptic proteins, and lipid organelles all accumulate as insoluble intracellular inclusions.

**Funded by**: DM is supported by the start-up funds from DZNE and the German Research Foundation (SFB 1286/B10 and MI 2104). CH is supported by the IMP grant of Deutsche Demenzhilfe.

**Grant number**: SFB 1286/B10 and DFG Sachbeihilfe MI 2104

**Acknowledgement**: The authors thank the funding sources for the support.

**Calcium-dependent serine-threonine phosphatase and calcineurin inactivation mediated by baicalein attenuates prion protein-mediated neuronal cell damage**

Jeong-Min Hong, Jong-Hoon Kim, Ji-Hong Moon, Jae-Won Seol, and Sang-Youel Park

Biosafety Research Institute, College of Veterinary Medicine, Jeonbuk National University, Gobong ro, Iksan, Jeonbuk Korea

**Aims**: Prion diseases are a group of incurable and fatal neurodegenerative disorders characterized by neuronal cell death. Calcineurin and autophagy mediate prion-induced neurodegeneration, suggesting that the inhibition of calcineurin and autophagy may have a therapeutic benefit. Baicalein has been previously reported to exert neuroprotective effects against calcium-dependent neuronal cell death.

**Material and Methods**: SK-N-SH cells were used to determine calcineurin phosphatase activity and calcium contents.

**Results**: In the present study, we investigated whether baicalein attenuates prion peptide-mediated neurotoxicity and reduces calcineurin. We found that baicalein treatment inhibits prion protein-induced apoptosis. Baicalein inhibited calcium up-regulation and protected cells against prion peptide-induced neuron cell death by calcineurin inactivation. Furthermore, baicalein increased p62 protein levels and decreased LC3-II protein levels, suggesting autophagic flux inhibition and related baicalein inhibition of prion protein-induced neurotoxicity.

**Conclusions**: These data demonstrated that baicalein attenuates prion peptide-induced neurotoxicity via calcineurin inactivation and autophagic flux reduction. This conclusion suggests that baicalein may be an effective therapeutic drug against neurodegenerative diseases, including prion diseases.

**Funded by**: National Research Foundation of Korea (NRF) funded by Ministry of Education

**Grant number**: 2019R1A6A1A03033084

**ARR/ARR genotype sheep show no resistance to ovine adapted c-BSE infection by the oral route**

Alvina Huor^a^, Frédéric Lantier^b^, Jean-Yves Douet^a^, Séverine Lugan^a^, Naïma Aron^a^, Chloé Mesic^a^, Hervé Cassard^a^, Tomás Barrio^a^, Hugh Simmons^c^, Isabelle Lantier^b^, and Olivier Andreoletti^a^

^a^UMR IHAP 1225, INRAe/ENVT, Toulouse, France; ^b^ISP, INRAe, Nouzilly, France; ^c^APHA, Weybridge, UK

**Aims**: In sheep, the susceptibility to Prion diseases is strongly determined by the polymorphisms at codons 136 (A or V), 154 (R or H) and 171 (R, Q or H) of the *PRNP* gene. Several studies reported the absence of transmission of classical Bovine Spongiform Encephalopathy (c-BSE) in A_136_R_154_R_171_/ARR *PRNP* genotype sheep following oral challenge. On this basis, selection of the ARR allele has been promoted as a mean to prevent c-BSE propagation in sheep population. However, some data suggested that ARR/ARR resistance to TSE agent infection might be lower than initially described. This study aims at (i) documenting the transmissibility by the oral route of ovine adapted c-BSE agent in sheep (including ARR/ARR sheep) and (ii) the risk for human health that could result from such transmission.

**Material and Methods**: ARQ/ARQ and ARR/ARR lambs were orally challenged using c-BSE agent previously adapted in sheep. Animal groups from each genotype were killed at 4 and 10 months post-inoculation (mpi) and at the clinical stage of the disease. Blood samples were regularly collected in each animal. The c-BSE agent (seeding activity) levels in the blood and tissues samples were quantified by PMCA endpoint titration. Bioassays in human PrP- expressing mice (Valine_129_ and Methionine_129_ variants) were used to gauge the capacities of the propagated Prions to cross the human species barrier.

**Results**: In both ARR/ARR and ARQ/ARQ c-BSE orally challenged animals, a 100% efficient transmission was observed. Although incubation period in ARR/ARR sheep were significantly longer than in ARQ/ARQ animals, both groups displayed a similar distribution of the c-BSE agent in their organism. At the clinical stage of the disease, c-BSE seeding activity titers measured by PMCA were similar in tissues from ARQ/ARQ and ARR/ARR sheep.

Bioassays in bovine and human PrP-expressing mice indicated that the passage of c-BSE in ARR/ARR sheep did not alter its strain properties or apparent zoonotic potential.

**Conclusions**: ARR/ARR genotype does not confer resistance against oral infection by c-BSE agent adapted in ovine. This finding highlights the limitations of the ARR/ARR allele selection policy as a mean to prevent and control c-BSE occurrence and spread in farmed sheep populations.

Beyond this, our results also illustrate the fact that the crossing of transmission barriers can deeply alter TSE agents’ biological properties without affecting the phenotypic traits classically used to identify strains.

**Funded by**: EU grant^a^, EU FEDER/INTERREG^b,^REDPRION^c^

**Grant number**:^a^QLK-CT 2001–309 (‘BSE in sheep’), ^b^EFA282/13 TRANSPRION, ^c^EFA148/16

**Acknowledgement**: All contributors and funders

**Serial RT-QuIC to increase sensitivity and specificity for CWD**

Soyoun Hwang^a^, Konstantin Alekseev^a,b^, Danielle Beckley^a,b^, and Eric M. Nicholson^a^

^a^Virus and Prion Research Unit, National Animal Disease Center, Agricultural Research Service, United States Department of Agriculture, Ames, IA, USA; ^b^Oak Ridge Institute for Science and Education (ORISE) through an interagency agreement between the U.S. Department of Energy (DOE) and the U.S. Department of Agriculture (USDA)

**Aims**: The study goal is to evaluate the potential to enhance diagnostic sensitivity and specificity for CWD through the use of serial RT-QuIC of fecal samples from white-tailed deer. The basis for this is supported by αobservations of more rapid and sensitive detection of tissue based RT-QuIC detection of prion disease.

**Material and Methods**: Tissue and fecal samples used in this work are from previously completed animal studies at National Animal Disease Center. Serial RT-QuIC assay conditions were were initially optimized using brain homogenate samples to ascertain optimal first-round reaction times, with this first round reaction time applied to RT-QuIC reactions seeded with fecal samples from CWD inoculated white-tailed deer. The solvent conditions for fecal and brain samples are independently determined.

**Results**: Serial RT-QuIC resulted in faster overall detection times with better ability to discriminate positive and negative samples fundamentally increasing both the sensitivity and specificity for CWD affect animals.

**Conclusions**: Serial RT-QuIC can be useful for rapid detection of CWD in white-tailed deer using fecal samples. Such an enhancement should be considered in the advancement of RT-QuIC as an approved assay for detection of CWD.

**Funded by**: USDA appropriated funds

**Acknowledgement**: All samples used in this study were provided by Dr. Justin Greenlee at National Animal Disease Center, USDA, Ames, IA, USA.

**Chemical Optimization of Cellular Prion Protein Degraders**

Nicole Innocenti^a,b^, Valerio Bonaldo^a^, Giovanni Spagnolli^c^, Tania Massignan^c^, Dino Gasparotto^a,b^, Giorgia Susin^a,b^, Giorgia Contessi^a,b^, Cecilia Perrucci^a,b^, Laura Copat^a,b^, Eleonora Parolin^a,b^, Massimo Casagranda^b^, Samuele Brugnara^b^, Giacomo Ambrosino^a,b^, Andrea Astolfi^d^, Maria Letizia Barreca^d^, Ines Mancini^b^, and Emiliano Biasini^a^

^a^Department CIBIO & University of Trento; ^b^Department of Physics, University of Trento; ^c^Sibylla Biotech S.R.L; ^d^Department of Pharmaceutical Sciences, University of Perugia

**Aims**: We have recently developed an approach for selectively reducing the level of target proteins by impairing their folding process rather than targeting their native conformations. This method, called Pharmacological Protein Inactivation by Folding Intermediate Targeting (PPI-FIT), is made possible by computational algorithms allowing the full atomistic reconstruction of folding and misfolding processes of polypeptides. The rationale underlying PPI-FIT is that targeting a folding intermediate with small ligands could promote its degradation by the cellular quality control machinery, which recognizes such artificially stabilized intermediates as improperly folded species. We have applied PPI-FIT to target the cellular prion protein (PrP), a key player in prion diseases, and identified a pharmacological degrader (named SM875) capable of dose-dependently suppressing the expression of the protein (1). We capitalized on a unique rationale, solid preliminary data, key experimental tools, and a highly cross-disciplinary team to move SM875 along the preclinical drug discovery pipeline. This effort could bring the first pharmacological degrader against prion diseases to the clinical phase.

**Material and Methods**: We designed and performed a synthesis scheme for SM875. The correct structure of the molecule was verified by nuclear magnetic resonance (NMR), mass spectrometry (MS), and infrared (IR) spectroscopy. Such a scheme allowed us to introduce modifications into the chemical scaffold of the compound, leading to tens of different analogs. Each molecule was then tested in vitro to assess the ability to suppress PrP.

**Results**: We tested 35 synthetic analogs differing from the parent compound SM875 for one or more chemical substitutions. Dose-dependent analysis of each molecule in an imaging-based cellular assay allowed us to draw a first structure-activity relationship for SM875, which was used to refine the docking model of the compound-pocket interaction. These results represent fundamental steps along the SM875 optimization pipeline and encourage searching for additional analogs with improved pharmacological and pharmacokinetic properties.

**Conclusions**: Our data represent the first step to move SM875 along the drug-discovery pipeline, which could ultimately lead to a drug administrable in vivo and the subsequent validation in animal models of prion diseases, fundamental steps to translate this new approach to the clinical phase.

**References**:

1. Spagnolli et al. Commun Biol. 2021

**Funded by**: Fondazione Telethon, Italy

**Grant number**: GGP20043

**Funded by**: CJD Foundation, USA

**The fidelity of prion templating *in vitro* depends on the identity of the prion strain**

Kezia Jack, Mark Batchelor, John Collinge, and Jan Bieschke

UCL Institute of Prion Diseases/MRC Prion Unit, London, UK

**Aims**: Although prions are all formed from the same single protein sequence, different prion strains cause consistent disease symptoms and characteristics, meaning phenotypic information is contained in the physical structure of the prion. The fidelity of templating can be influenced by seed structure and folding environment. In my project, I aim to investigate *in vitro* which of these two factors has a stronger influence on the structural templating of prions.

**Material and Methods**: I have used a native state aggregation assay to fibrilise two different PrP monomers (23–231 and 91–231) using *ex vivo* prions (RML, ME7) and synthetic PrP fibrils as seeds. The resulting fibrils were propagated over multiple rounds of seeding. To probe the structures of the resulting fibrils in a quantitative and high-throughput way, I have developed a spectral fingerprinting method using luminescent conjugated oligothiophene dyes.

**Results**: Fibrils seeded by RML prions, ME7 prions and synthetic fibrils had significantly different structures. Over subsequent seeding rounds, the seeding fidelity varied depending on which strain was used as initial seed, and whether the PrP substrate included or lacked the N-terminal amino acids (23–89).

**Conclusions**: The N-terminal region is not incorporated into genuine prions, and so it presence or absence alters the folding environment of the amino acids that are incorporated into the fibril. This suggests that the interplay between seed structure and folding environment is complex, and the relative strengths vary with different strains.

**Funded by: UKRI, MRC**

**Grant number**: MRC MC_UU_00024/6

**Synthetic prions with high specific infectivity generated from recombinant PrP.**

Graham S, Jackson, Adam Wenborn, Jemma Betts, Ines Whitworth, Christian Schmidt, Jacqueline Linehan, Jonathan DF Wadsworth, and John Collinge

MRC Prion Unit, UCL Institute of Prion Diseases, UK

**Aims**: To generate synthetic prions from recombinant PrP with high specific infectivity to facilitate their isolation and subsequent characterisation, including a determination of their atomic structure.

**Material and Methods**: We have developed a modified method of Protein-Misfolding by Cyclic Amplification (PMCA) which utilises recombinant prion proteins (rPrP) and an extract of brain homogenate from PrP-knockout mice. When used in a serial format synthetic prions can be replicated *de novo* to very high titres in combination with extreme dilution of the intiating seeds. The resultant synthetic prions have been purified to near homogenity with respect to protein and characterised using a variety of methods. prions have been quantitatively titred and possess a specific infectivity equal to or in excess of those extracted *ex vivo*. In common with the propagation of prions in mammalian hosts our synthetic prions can encode distinct strains, dependent upon the original initiating ‘seed’.

**Results**: The purity of isolated, synthetic prions has been assessed using silver-stained SDS-PAGE and mass spectrometry which has confirmed the absence of significant protein contaminants. Quantification of PrP content by ELISA in conjunction with cell-culture measurements of infectivity have established the purified prions have a specific infectivity equivalent to those isolated from mammalian brain which was confirmed by transmission in rodent bioassay. The overall architecture of the isolated prions visualized by negative stain electron microscopy appeared closely similar to that of prion rods isolated from rodent brain. Cryo-electron microscopy confirms the structures seen by negative stain and the determination of an atomic structure is currently underway.

**Conclusions**: Synthetic prions with high specific infectivity can be generated from recombinant PrP in the presence of a brain homogenate extract. The resulting prions can be purified to homogeneity and do not contain any proteinaceous component other than PrP. Following digestion with proteinase K and analysis by mass spectrometry a core of residues 85–231 remain which is in common with prions obtained ex vivo. The infectious titre or concentration of synthetic prions generated is higher than that contained within typical brain homogenates and following purification, prion rods visualized by cryo-electron microscopy closely resemble those obtained from mammalian brain. Thus post-translational modifications such as glycosylation are not a prerequisite for the formation of infectious prion rods.

**Funded by**: UK Medical Research Council

**Grant number**: MC_U123170362

**Citrullinated GAPDH and vimentin in the pathology of prion diseases**

B. Jang^a^, MJ. Kim^b^, YJ. Lee^a^, MJ. Choi^a^, SJ. Park^a^, A. Ishigami^c^, YS. Kim^a,d^, and EK. Choi^a,b^

^a^Ilsong Institute of Life Science, Hallym University, Seoul, Korea; ^b^Department of Biomedical Gerontology, Graduate School of Hallym University, Chuncheon, Korea; ^c^Molecular Regulation of Aging, Tokyo Metropolitan Institute of Gerontology, Tokyo, Japan; ^d^Department of Microbiology, College of Medicine, Hallym University, Chuncheon, Korea

**Aims**: Protein citrullination, the calcium (Ca^2+^)-dependent peptidylarginine deiminase (PAD)-mediated conversion of a protein arginine residue to a citrulline residue, has emerged as a pathophysiologic outcome in neurodegeneration. However, the roles, functions, and expression of citrullinated proteins have not yet been elucidated because available antibodies are limited.

**Material and Methods**: We developed mouse monoclonal IgG1 or IgM specific for citrullinated GAPDH and vimentin to investigate the pathogenesis of prion diseases in animal models, in patients with prion diseases, and *in vitro*.

**Results**: Glycolytic enzyme GAPDH is citrullinated at R13 and R200. Citrullination did not regulate GAPDH enzyme activity. Full-lenth 37 kDa and fragments 25 kDa in size were mainly citrullinated at R200 residue but not at R13 residue, and were highly detected in sporadic Creutzfeldt-Jakob disease (sCJD). The type III intermediate filament protein vimentin citrullinated at R450 was specifically detected in scrapie-infected mice. Vimentin was also highly citrullinated and was mainly expressed in reactive astrocytes and in neuro-inflammatory marker YKL-40-positive cells in the brain tissues of sCJD patients. Citrullination led to increased cytoplasmic and integral membrane/organelle vimentin enrichment. Caspase sensitivity of vimentin was also changed by citrullination.

**Conclusions**: Our findings suggest that these newly developed antibodies may be useful for identifying disease activity and the pathogenesis of human disorders.

**Funded by**: National Research Foundation of Korea (NRF) grant funded by the Korea government (Ministry of Science and ICT)

**Grant number**: 2022R1A2C1006085; NRF-2020R1A2C2009566

**Acknowledgement:**

**Neuropathology of 8 patients of the New Brunswick cluster of Neurological Syndrome of Unknown Cause; human Chronic Wasting Disease or blue-green algae?**

Gerard H. Jansen^a^, Dean A. Fergusson^b^, John M. J. Woulfe^a^, and Alexander S. Easton^c^

^a^Department of Pathology and Laboratory Medicine, University of Ottawa, Ottawa, Ontario, Canada; ^b^Clinical Epidemiology Program, The Ottawa Hospital Research Institute, Ottawa, Ontario, Canada; ^c^Department of Pathology, QEII Health Science Centre, Halifax, Nova Scotia, Canada

**Aims**: Since late 2019 patients were reported from one Canadian province to the Canadian CJD surveillance, and it was suggested these suffered from a new unknown neurological disease, possibly human CWD. In March 2021 this cluster became world news. 48 of the patients of this cluster of Neurological Syndrome of Unknown Cause were investigated by the Oversight Committee established by the New Brunswick government. 96% of cases had been referred by one local neurologist. Ten patients had died at the time of writing of this abstract, and eight came to autopsy. In April 2021, following the results of the first autopsies, suggestions for other environmental factors causing this cluster were made: aberrant amino acid BMAA from blue-green algae. The Oversight Committee was briefed in August 2021 of the results of our study, and partly based on those, on 24 February 2022 the province of New Brunswick concluded that there was no evidence to support a new mysterious illness.

**Material and Methods**: Brain tissue was obtained at autopsy as formalin fixed (8/8) and frozen (7/8). Routine stains were performed, and PrP (12F10), TDP43, β-amyloid, Tau, P62, α-Synuclein. Slides were reviewed by 2 independent neuropathologists. Western blotting for PrP was performed (7/8).

**Results**: In none of these 8 cases microscopical or immunohistochemical features of a prion disease were found. No misfolded prion protein was detected by WB. Pathology showed that the group of 8 consisted of: one case of vascular dementia; one case without morphological changes (consistent with clinical history); one case of neoplasia; and 5 cases of neurodegenerative disease. The latter group showed in each case mixed dementia with as main component Alzheimer disease (2/5), neocortical Lewy body pathology (2/5), or frontotemporal lobe dementia TDP43 (1/5).

**Conclusions**:

1. No prion disease was found in any of the 8 autopsy cases.
2. An inhomogeneous group of diagnoses was found in the 8 cases. Considering the 48 total cases and a conservative diagnostic acumen, the probability of having all 8 consecutive autopsy cases representing a new mystery illness is exteremly remote (p = 0.0001 using classical probability theorem). Therefore, the cluster constitutes not a new disease, but a cluster of clinical misdiagnoses.
3. The suggestion that blue-green algae BMAA would be causing known misfolding diseases, initiating this cluster, can be dismissed as 3/8 patients did not suffer from a misfolding disease. Moreover, the original presentation in this cluster is not consistent with this hypothesis as all 8 were initially suggested to have a ‘new-unknown’ disease, not a ‘known’ neurodegenerative disease.

**Funded by**: Pathology work-up and Western blotting was funded by Public Health Agency of Canada. No other funding/support was received.

**Acknowledgement**: The University of Ottawa Neuropathology Laboratory Services staff; Olga Agah, Eric Labelle, and Sharlene Faulkes. The National Microbiology Laboratory in Winnipeg staff: Dr. J. David Knox and Anne Peterson.

**RAD51 demonstrates amyloid properties *in vivo* and *in vitro***

Daniel Kachkin^a^, Julia Sopova^a,b^, Kirill V. Volkov^c^, Alexandr G. Bobylev^d^, Sergey Fedotov^a^, Ivan I. Kostroma^e^, Aleksandr A. Rubel^a^, and Anna Yu. Aksenova^a^

^a^Laboratory of Amyloid Biology, St. Petersburg State University, St. Petersburg, Russia; ^b^Center of transgenesis and genome editing, St. Petersburg State University, St. Petersburg, Russia; ^c^Research Resource Center ‘Molecular and Cell Technologies’, Research Park, St. Petersburg State University, St. Petersburg, Russia; ^d^Institute of Theoretical and Experimental Biophysics, Russian Academy of Sciences, Moscow, Russia; ^e^Russian Research Institute of Hematology and Transfusiology, St.Petersburg, Russia

**Aims**: Amyloids are highly ordered fibrillar protein polymers capable of self-assembly and possessing intermolecular cross-β structure. The revelation of the role of amyloids in the development and progression of cancer has been one of the important developments in recent years.

During the screening of potentially amyloidogenic proteins, we discovered that the human protein RAD51 has the potential for amyloid aggregation. RAD51 is one of the key proteins of homologous DNA double-strand break repair responsible for genome integrity and stability. In the course of our study, we demonstrated the amyloid properties of human RAD51 protein and proposed the role of its aggregation on the progression of cancer.

**Material and Methods**: We have purified the recombinant protein from *E. coli*, aggregated it *in vitro* and studied its amyloid properties. We stained RAD51 aggregates with amyloid-specific dyes such as Thioflavin T and Congo red. Aggregation dynamics and seeding potential of RAD51 aggregates were studied by using Thioflavin T assay. The resistance of RAD51 aggregates to the action of ionic detergent (1% SDS) was examined. Fibrils of RAD51 were visualized on a transmission electron microscope. X-ray diffraction analysis was used to confirm the cross-beta structure of the fibrils formed by RAD51. Tissues of patients with various forms of hematologic malignancies were analyzed for the presence of an aggregated form of the RAD51 protein with SDD-AGE assay.

**Results**: In this work, we demonstrated that a key protein that maintains genome stability, RAD51, has amyloid properties *in vitro*. We have shown that RAD51 *in vitro* forms detergent-resistant aggregates that have an unbranched fibrillar structure. RAD51 aggregates bind with amyloid-specific dyes. Aggregates of RAD51 dyed with Congo red glow in polarized light. Analysis of the RAD51 fibrils by X-ray diffraction demonstrated a cross-β structure that characterizes amyloid proteins. Moreover, in the tissues of patients with multiple myeloma and acute leukaemia SDS-resistant aggregates of the RAD51 protein were detected.

We propose here that amyloid aggregation of RAD51 may be relevant to cancer development.

**Conclusions**: To our knowledge we have demonstrated the amyloid properties of the human RAD51 protein for the first time.

**Funded by**: Saint Petersburg State University and Russian Science Foundation

**Grant number**: Pure ID: 93,025,998, 92,561,695 (SPSU) and № 20–14-00148 (RSF)

**Cell type-specific translatome signatures in pre-onset prion disease mice**

Lech Kaczmarczyk^a,b^, and Walker S. Jackson^a,b^

^a^Wallenberg Center for Molecular Medicine, Department of Biomedical and Clinical Sciences, Linköping University, Linköping, Sweden; ^b^German Center for Neurodegenerative Diseases, Bonn, Germany

**Aims**: To identify potential therapeutic targets we aimed to identify molecular responses of specific cell types to early-stage prion disease.

**Material and Methods**: We employed RiboTag translational profiling to identify molecular responses of five cell types at pre-onset and onset disease stages of mice infected with the RML strain of mouse adapted scrapie prions. The two disease stages were identified from serial electroencephalography (EEG). The cell types included astrocytes plus four neuron subtypes with varying vulnerabilities to prion disease.

**Results**: Serial EEG showed that theta frequency waves gradually increased in RML infected mice starting at 13 weeks post injection (WPI) and became significant at 18 WPI, although typical features of mouse scrapie (e.g., ataxia, kyphosis) were still absent. At this stage, translatome responses were surprisingly strong in all cell types studied. At the pre- onset stage of 10 WPI, parvalbumin and somatostain neurons showed no coordinated response but the other three cell types showed coordinated responses, each with a uniquesignature. Specifically, astrocytes downregulated genes needed for ribosome biogenesis, glutamatergic neurons altered genes needed for the cytoskeleton, and GABAergic neurons altered several core circadian rhythm genes. In a follow-up study, interference of the circadian rhythm only slightly enhanced disease of RML infected mice

**Conclusions**: The strong response seen at 18 WPI indicates that during early signs of diminishing brain function (e.g., EEG theta), brain cells are already severely altered and therapies may need to tackle multiple problems. At 10 WPI, well before the emergence of EEG theta increase, clinical signs and neuropathological changes, cells make specific and disparate molecular responses. Since parvalbumin neurons have been reported to be especially vulnerable in human and animal prion disease, the lack of response at 10 WPI suggests they are unable to detect the ensuing disease and provides no obvious molecular target in these cells to engage therapeutically. Furthermore, the minimal enhancement of disease severity following circadian rhythm interference suggests there may be little therapeutic benefit from rigorously controlling it. Nonetheless, a few interesting genes were identified, one of which we found activated by other aggregated proteins such as amyloid beta and alpha synuclein. To test for any therapeutic potential, a novel knock-in mouse line to control its expression has been created.

**Funded by**: Internal funding of the Wallenberg Center for Molecular Medicine and the German Center for Neurodegenerative Diseases

**Liquid-liquid phase separation of the prion protein promotes the formation of neurotoxic aggregates; a critical role of the N-terminal domain**

Janine Kamps^a,b^, Nuwin Mohamad^a^, Verian Bader^c^, Konstanze F. Winklhofer^b,c^, and Jörg Tatzelt^a,b^

^a^Department Biochemistry of Neurodegenerative Diseases, Institute of Biochemistry and Pathobiochemistry, Ruhr University Bochum, Germany; ^b^Cluster of Excellence RESOLV, Bochum, Germany; ^c^Department Molecular Cell Biology, Institute of Biochemistry and Pathobiochemistry, Ruhr University Bochum, Germany

**Aims**: Formation of biomolecular condensates through liquid-liquid phase separation (LLPS) has been described for several pathogenic proteins linked to neurodegenerative diseases and is discussed as an early step in the formation of protein aggregates with neurotoxic properties.

We have demonstrated that the unstructured N-terminal domain of the prion protein (PrP) is necessary and sufficient to promote LLPS, emphasizing the important role of intrinsically disordered low-complexity domains (LCD) in phase separation. The liquid-like state of the PrP condensates was proven by fluorescence recovery after photobleaching (FRAP) and droplet fusion assays. Mechanistically, LLPS of PrP is driven by a highly conserved polybasic motif in the N-terminal domain, previously identified as binding site for neurotoxic Aβ oligomers, presumably largely via cation-π interactions (Kamps *et al*., 2021). Previous studies revealed that LCDs not only promotes LLPS but also the formation of different types of assemblies, including reversible amyloid-like fibrils (hydrogels) and irreversible amyloids, depending on the chemical milieu. We therefore tested whether the LCD of PrP is also implicated in a conformational transition of natively folded PrP into aberrant protein assemblies with neurotoxic activity.

**Material and Methods**: We analyzed the formation of different types of protein assemblies by various methods, including super-resolution structured illumination microscopy (SR-SIM), fluorescent recovery after photobleaching (FRAP), Thioflavin T staining, circular dichroism spectroscopy, dynamic light scattering and atomic force microscopy (AFM). The cytotoxic activity of the protein assemblies formed were tested in our established cell culture models, including primary neurons.

**Results**: We have identified specific conditions *in vitro* under which biomolecular condensates formed by full length prion protein either dissolve or transform into undynamic assemblies with cytotoxic activities.

**Conclusions**: Our experiments provided evidence for the concept that the unstructured N-terminal domain of PrP can modulate the conformational transition of PrP into protein assemblies with distinct biological activities.

**Funded by**: Deutsche Forschungsgemeinschaft

**Grant number**: EXC 2033 – 390,677,874 – RESOLV, TA 167/6-3, TA 167/11-1

**Prion photocatalytic inactivation**

Eirini Kanata^a^, Ioannis Paspaltsis^a^, Sotirios Sotiriadis^b^, Matthias Schmitz^c^, Athanasios Arsenakis^d^, Christina Niovi Papanikolaou^a^, Zoi Patsani^a^, Dafni Dodopoulou^a^, Dimitra Paschaloudi^a^, Chrysanthi Berberidou^e^, Sofia Tsoumachidou^e^, Ioannis Poulios^e^, Dimitra Dafou^b^, Konstantinos Xanthopoulos^a^, and Theodoros Sklaviadis^a^

^a^School of Pharmacy, Aristotle University of Thessaloniki, Thessaloniki, Greece; ^b^School of Biology, Aristotle University of Thessaloniki, Thessaloniki, Greece; ^c^Department of Neurology University Medical School Göttingen, Germany; ^d^Sterimed S.A., Thessaloniki, Greece; ^e^School of Chemistry, Aristotle University of Thessaloniki, Thessaloniki, Greece

**Aims**: Prions are infectious agents extremely resilient to standard decontamination methods. Advanced oxidation processes generate strong oxidizing agents which non-selectively attack and minearilize organic compounds, previously applied for pathogen inactivation, including prions. We evaluated heterogenous and homogenous photocatalytic oxidation on prion inactivation, aiming to simulate prion as a possible contaminant in liquid waste and assess the feasibility of photocatalytic prion inactivation within this context.

**Material and Methods**: We treated final stage RML-mice brain homogenate (0.1% w/v) with TiO_2_ P25 nanoparticles and UV-A illumination (heterogenous) or with the photo-Fenton reagent, Fe^3+^/H_2_O_2_/UV-A (homogenous photocatalytic oxidation). For increased efficiency, H_2_O_2_ (1000 mg/l) was included. UV-A light lamps were used as light source, whereas controls (brain homogenate and catalyst without H_2_O_2_ and UV-A illumination) were included. Material treated for 6 or 12 h was administered intraperitoneally to C57Bl/6 J mice, while control mice received control material. Animals were examined for the appearance of symptoms and sacrificed at terminal stage or >1 year after challenge if no symptoms had developed. Brain tissue was sampled for neuropathological evaluation, WB and RT-QuiC analysis.

**Results**: Our bioassay indicated that the heterogenous photocatalytic treatment (catalyst: TiO_2_) efficiently deactivates RML prions. Animals receiving material treated for 12 h did not develop clinical symptoms and were sacrificed at 400 dpi (9/9). Treatment for 6 h reduced significantly the titer as 78% of mice (7/9) did not develop symptoms (and were sacrificed at 400 dpi). In contrast, animals receiving material treated with homogenous photocatalysis (catalyst: FeCl_3_) were not protected and succumbed to disease. In this case a longer treatment moderately reduced infectivity, as evidenced by the longer survival interval and the smaller penetration (8/9 mice for 6 h treatment and 7/9 mice for 12 h treatment developed symptoms and were sacrificed). WB and RT-QuiC analyses are underway to investigate residual PrP^Sc^in **Conclusions**: Our data highlight the feasibility of utilizing heterogenous photocatalytic oxidation for prion inactivation. Prions may occur in medical liquid waste, and although we tested simulated material carrying substantially higher prion loads than expected in real medical liquid waste, efficient prion inactivation was achieved within a reasonable timeframe (12 h), significantly reducing the risk of prion infectivity. We are currently developing an industrial scale reactor for efficient medical liquid waste management.

**Funded by**: The work was implemented in the framework of «Competitiveness, Entrepreneurship and Innovation» co-financed by the European Regional Development Fund (ERDF) of the European Union and national resources through the Operational Programme Competitiveness, Entrepreneurship and Innovation (EPAnEK).

**Grant number**: T1EDK-02678asymptomatic animals.

**Investigation of the role of RNA editing in immunoregulation in Creutzfeldt – Jakob disease pathogenesis**

Korina. Karagianni^a^, Alba M. Moreno^b^, Spyros. Pettas^a^, Eirini Kanata^c^, Juan C. Espinosa^b^, Konstantinos Xanthopoulos^c^, Juan M. Torres^b^, Dimitra Dafou^a^, and Theodoros Sklaviadis^c^

^a^Department of Genetics, Development and Molecular Biology, School of Biology, Aristotle University of Thessaloniki, Thessaloniki, Greece; ^b^Centro de Investigación en Sanidad Animal (CISA-INIA-CSIC), Valdeolmos, Madrid, Spain; ^c^Laboratory of Pharmacology, School of Pharmacy, Aristotle University of Thessaloniki, Thessaloniki, Greece

**Aims**: This study aims to investigate in depth the role of RNA editing alterations in molecular mechanisms involved in sporadic Creutzfeldt–Jakob disease (sCJD) initiation and progression, emphasizing on immunoregulation, through targeted investigation of microglial populations. Our central hypothesis is that altered RNA editing in microglial cells provides epitranscriptomic changes representing a novel mechanism contributing to transcriptome diversity and subsequent translational changes triggered by or resulting in protein misfolding. Thus, our study focused on *in vivo* and *in silico* studies in resident (microglia) and peripheral (macrophages) immune cell populations from a humanized CJD *in vivo* animal model in early, middle and later disease stages, to reveal transcriptomic and RNA editome signatures that specifically relate to increasing levels of pathology, from minimal PrP^Sc^accumulation to massive neuronal loss. Emphasis was given to parallel comparison of microglia and peripheral macrophages under physiological and neuroinflammatory conditions.

**Material and Methods**: We utilized Tg340-PRNP129 MM mice infected with postmortem material from sCJD patients of the most susceptible genotype (MM1 subtype), a sCJD model that faithfully recapitulates the molecular and pathological alterations of the human disease. Hu-sCJD Tg340 mice and corresponding aged-matched controls, were sampled in four critical time points (0, 60, 120 & 180 days post infection -dpi). Microglia and peripheral macrophages were isolated using CD11b antibody-coupled microbeads. Transcriptomic and epitranscriptomic profiles were obtained through deep RNA sequencing analysis using the IonTorrent platform. RNAseq data were subjected to an advanced ‘in house’ QC pipeline, followed by gene expression and RNA editing analyses based on the DESeq and REDItools/SPRINT algorithms.

**Results**: Global microglial RNA editing profiles (editomes) were established during disease progression. RNA editing events mediated by both ADAR (A-I) and APOBEC (C-U) were identified. Similar to bulk tissue analysis (cortex) of the same animal model (https://doi.org/10.1073/pnas.1803521116), reduced global RNA editing was detected with disease progression. Differential RNA editing in microglial transcripts, identified several differentially edited transcripts (e.g. (*B2m, Paqr8*), replicating and further extending our previous bulk tissue analyses, thus allowing a cell-type specific RNA editing analysis. Pathway analysis identified molecular processes affected by RNA editing alterations during disease progression. Further functional prediction *in silico* analyses are conducted for selection of the most potent targets to be used for subsequent experimental verification of RNA editing perturbations and for functional assays. Comparative analysis between microglia and peripheral macrophages are conducted to investigate whether RNA editing perturbations in the brain are reflected in the periphery.

**Conclusions**: Our data shed light into microglial RNA editing changes occurring during sCJD progression, enabling elucidation of disease-associated microglial processes. Similar analysis on peripheral macrophages are expected to provide information regarding the biomarker potential of peripheral macrophages, provided that microglia-macrophages editome correlations are identified.

**Acknowledgement**:

This research has been co‐financed by the European Regional Development Fund of the European Union and Greek national funds through the Operational Program Competitiveness, Entrepreneurship and Innovation, under the call RESEARCH – CREATE – INNOVATE (project code:T1EDK-03884)

Fundació La Marató de TV3, Grant/Award Number: 201,821–31.

Project PID2019-105837RB-I00 from MCIN/AEI/10.13039/501,100,011,033

**Long double stranded RNA is detected in 22 L scrapie infected mouse br**

Yervand E. Karapetyan, and Sonia Frasquilho

Integrated Biobank of Luxembourg, Dudelange, Luxembourg

**Aims**: The nature of the infectious agent causing scrapie and other TSEs remains an enigma. The protein-only prion hypothesis posits host glycoprotein’s abnormal conformer as the primary or sole component of the infectious agent. On the other hand, viral nucleic acids have long been sought in scrapie to explain the existence of multiple agent strains. Despite a plethora of different approaches to the search, no scrapie-specific nucleic acid sequences have been found in infected tissues. Most viruses induce the synthesis of long double stranded RNA (dsRNA) during their replication in cells of infected tissues, and thus the detection of long dsRNA would be an indication of viral infection.

**Material and Methods**: J2 monoclonal antibody against long dsRNA (longer than 40 base pairs) is a useful tool for screening of cells and tissues for the presence of suspected viral infection; however, this antibody has not previously been used for testing of scrapie infected tissues. We have validated and optimized the method for the immunohistochemical detection of long double stranded RNA using J2 antibody in formalin fixed and paraffin embedded tissues with Ventana Benchmark XT automated stainer. Then we applied this method to test two CD1 mouse brains terminally infected with 22 L scrapie strain and three CD1 age and sex matched uninfected mouse brains.

**Results**: Using J2 IHC we detected strong immunostaining for long dsRNA in the cytoplasm of neurons in two out of two 22 L scrapie infected mouse brains. We observed the strongest staining in the neurons of the brainstem and thalamus of the infected mice. No staining was observed in the neurons of the corresponding regions of all three uninfected mouse control brains.

**Conclusions**: For the first time we present direct evidence of viral infection in mouse scrapie brains demonstrated by immunohistochemical detection of long dsRNA only in infected tissues and not in controls.

The sequencing of these long dsRNAs using J2 immunoprecipitation of total RNA extracts from infected brains and applying specific dsRNA-seq methods will shed light on the issue of the nature of the infectious agent in scrapie and other TSEs.

**Funded by**: Integrated Biobank of Luxembourg

**Acknowledgement**: We thank Dr. Daisuke Ishibashi for providing the 22 L mouse brains blocks and Dr. Michel Mittelbronn and his group for providing the control mouse brains.

**Immunological role of cellular prion protein (PrP^C^) during cytomegaloviral infection**

Dubravka Karner^a^, Daria Kveštak^a^, Paola Kučan Brlić^a^, Maja Cokarić Brdovčak^a^, Berislav Lisnić^a^, Ilija Brizić^a^, Vanda Juranić Lisnić^a^, Mijo Golemac^a^, Jelena Tomac^b^, Giuseppe Legname^c^, Hermann C Altmeppen^d^, Milena Hasan^e^, Stipan Jonjić^a^, and Tihana Lenac Roviš^a^.

^a^Center for Proteomics, Faculty of Medicine, University of Rijeka, Rijeka, Croatia; ^b^Department of Histology and Embryology, Faculty of Medicine, University of Rijeka, Rijeka, Croatia; ^c^Prion Biology Laboratory, Department of Neuroscience, Scuola Internazionale Superiore di Studi Avanzati (SISSA), Trieste, Italy; ^d^Institute of Neuropathology, University Medical Center Hamburg-Eppendorf, Hamburg, Germany; ^e^Cytometry and Biomarkers UTechS, Center for Technological Resources and Research, Institut Pasteur, Paris, France

**Aims**: Human cytomegalovirus (HCMV) infection is the most common congenital viral infection and the leading cause of lasting perinatal brain damage, with the inflammatory response being the primary cause of pathogenic manifestations. Accordingly, it has been shown that anti-inflammatory drugs can reduce abnormalities in newborn mice infected with mouse cytomegalovirus (MCMV). Since contemporary antiviral drugs have inadequate efficiency, finding new therapeutic targets that can reduce brain damage is of utmost importance. There are growing indications that cellular prion protein (PrP^C^) dampens the immune response in various organs and prevents collateral, immune response-mediated pathologies. In addition to being expressed on immune cells, PrP^C^can also bind to immune cells, suggesting its interaction with immune receptors. Our primary goal is to characterize the role and impact of PrP^C^on the course and severity of congenital cytomegalovirus infection and associated brain pathology.

**Material and Methods**: Intraperitoneal infection of newborn mice with MCMV recapitulates the major hallmarks of human congenital CMV infection: virus dissemination to the brain parenchyma, neuroinflammation and altered brain development. To determine the correlation between PrP^C^expression, inflammation and developmental and functional changes, we compare the following parameters in infected newborn WT and PrP^C^-KO mice: i) viral load; ii) cytokines (FACS, xMAP technology); iii) activation status and transcriptional profiles of immune cells (FACS, scRNA-seq); iv) CMV-related brain anomalies (IHC). In addition, we monitor the impact of CMV on PrP^C^biology at the cellular level *in vitro* (antibody development, FACS, confocal microscopy, immunoblot).

**Results**: We show that CMV infection in different cell lines and primary cells affects the amount of PrP^C^. After initial strong induction of PrP^C^expression at early time points of infection, cell-associated PrP^C^levels are largely reduced, partially by triggering its ADAM10-mediated cell surface shedding. Intriguingly, PrP^C^-KO mice have significantly lower virus titers in multiple organs, including the brain. Immune cell subsets and mechanisms responsible for more efficient virus clearance in the absence of PrP^C^protein are still being investigated.

**Conclusions**: Obtained data indicate that PrP^C^is involved in the immune response to CMV infection in newborn mice. Absence of PrP^C^improves control of the virus due to enhanced immune system activation.

**Funded by**: Croatian Science Foundation, University of Rijeka, Strengthening the capacity of CerVirVac for research in virus immunology and vaccinology

**Grant number**: IP-2020-02-6617, uniri-biomed-18-233, KK.01.1.1.01.0006

**Acknowledgement**: This work has been supported in part by Croatian Science Foundation under the project (IP-2020-02-6617) and by the University of Rijeka under the project number (uniri-biomed-18-233) and by the grant ‘Strengthening the capacity of CerVirVac for research in virus immunology and vaccinology’, KK.01.1.1.01.0006, awarded to the Scientific Centre of Excellence for Virus Immunology and Vaccines and co-financed by the European Regional Development Fund.

**Ex vivo target engagement of the Abeta oligomer disassembling compound RD2 in patient derived brain homogenates**

Bettina Kass^a^, Sarah Schemmert^a^, Christian Zafiu^a,c^, Marlene Pils^a,b,c^, Oliver Bannach^a,b,c^, Janine Kutzsche^a^, Tuyen Bujnicki^a^, and Dieter Willbold^a,b,c,d^

^a^Institute of Biological Information Processing, Structural Biochemistry (IBI-7), Forschungszentrum Jülich, Jülich, Germany; ^b^Institut für Physikalische Biologie, Heinrich-Heine-Universität Düsseldorf, Düsseldorf, Germany; ^c^attyloid GmbH, Düsseldorf, Germany; ^d^Priavoid GmbH, Düsseldorf, 40,225, Germany

**Aims**: Soluble Aβ oligomers are highly toxic and may be responsible for development and progression of Alzheimer’s disease (AD). Elimination of toxic and prion-like Aβ oligomers is therefore a promising therapeutic strategy. Inhibition of Aβ aggregation is too late in patients having prion-like replicating Aβ oligomers in their brain. Thus, direct disassembly of already existing Aβ oligomers into native Aβ monomers in their physiological conformation is the most promising mode of action (MoA). This MoA is not relying on the immune system and independent of the conformation of the Aβ oligomer strain/polymorph to be disassembled. To realize this MoA, the compound RD2 was developed to stabilize Aβ monomers in their native intrinsically disordered protein (IDP) conformation. RD2 has demonstrated already to directly eliminate soluble Aβ oligomers in vitro and in vivo and to improve cognition or decelerate neurodegeneration in four different animal models in four different laboratories. Here, we wanted to test, if RD2 is able to disassemble Aβ oligomers obtained from AD patient derived brain tissue.

**Material and Methods**: Aβ oligomers in AD patient derived brain tissue homogenates were incubated with different concentrations of RD2 and control compounds. Aβ oligomer concentrations were determined longitudinally by the sFIDA assay, a single-particle sensitive method for quantitating protein oligomers and aggregates.

**Results**: RD2 concentration and incubation time-dependent reduction of Abeta-oligomer concentration in AD patient brain derived homogenates was observed. Control compounds did not reduce Aβ oligomer concentrations.

**Conclusions**: Incubation time and concentration dependent RD2 disassembly of Aβ oligomers from human brain homogenate suggest a very efficient ability of RD2 to fulfill its mode of action, namely the direct disassembly of Aβ oligomers into their Aβ monomer building blocks. Kinetic analysis and the mode of action resemble very much that of catalytic chaperones.

**Altered expression of glymphatic system-related proteins in prion diseases: Implications for the role of the glymphatic system in prion disease**

Yong-Chan Kim, and Byung-Hoon Jeong

Korea Zoonosis Research Institute, Jeonbuk National University, Iksan, Republic of Korea

**Aims:** Prion diseases are fatal and irreversible neurodegenerative diseases caused by a pathogenic form of prion protein (PrP^Sc^) derived from a cellular form of prion protein (PrP^C^). Although several cellular mechanisms, including endoplasmic reticulum (ER)-mediated degradation (ERAD), unfolded protein response (UPR) and autophagy, can be activated to remove PrP^Sc^, overactivation of these cellular mechanisms exacerbates disease progression. Thus, identification of a novel therapeutic target is necessary. Recent studies have reported that aquaporin 4 (AQP4) is dramatically upregulated in the brains of several different prion diseases. Since AQP4 is a key protein of the glymphatic system, which is the perivascular waste clearing system of the brain, and since altered expression of AQP4 has been observed in prion diseases, the glymphatic system may be associated with prion diseases. Thus, investigation of the association between the glymphatic system and prion diseases is important.

**Material and Methods:** We investigated the expression pattern of glymphatic system-related molecules in prion-infected mice at 7 months postinjection and sporadic Creutzfeldt-Jakob disease (CJD) patients by western blotting and immunohistochemistry (IHC). In addition, we evaluated the protective effect of glymphatic system-activated drugs, including dexmedetomidine and clonidine, in prion-infected mice by western blotting and survival analysis.

**Results:** We identified altered band patterns of cleaved agrin and upregulation of neurotrypsin in prion-infected mice and sporadic CJD patients. We found dramatic clearance of PrPSc and amelioration of astrocytosis in dexmedetomidine- and clonidine-treated prion-infected mice at 5 months postinjection. In addition, we observed a significantly delayed incubation period in dexmedetomidine- and clonidine-treated prion-infected mice.

**Conclusions:** To the best of our knowledge, this is the first report of novel glymphatic system-related biomarkers and potential therapeutic substances in prion diseases.

**Funded by:** National Research Foundation of Korea

**Grant number:** 2021R1A2C1013213, 2022R1C1C2004792

**Acknowledgement:** Yong-Chan Kim was supported by the BK21 plus program in the Department of Bioactive Material Sciences.

**Large-scale lipidomic profiling identifies novel potential biomarkers for prion diseases and highlights lipid raft-related pathways**

Yong-Chan Kim, and Byung-Hoon Jeong

Korea Zoonosis Research Institute, Jeonbuk National University, Iksan, Republic of Korea

**Aims:** Prion diseases are transmissible spongiform encephalopathies induced by the abnormally-folded prion protein (PrPSc), which is derived from the normal prion protein (PrPC). Previous studies have reported that lipid rafts play a pivotal role in the conversion of PrPC into PrPSc, and several therapeutic strategies targeting lipids have led to prolonged survival times in prion diseases. In addition, phosphatidylethanolamine, a glycerophospholipid member, accelerated prion disease progression. Although several studies have shown that prion diseases are significantly associated with lipids, lipidomic analyses of prion diseases have not been reported thus far.

**Material and Methods:** We intraperitoneally injected phosphate-buffered saline (PBS) or ME7 mouse prions into mice and sacrificed them at different time points (3 and 7 months) post-injection. To detect PrPSc in the mouse brain, we carried out western blotting analysis of the left hemisphere of the brain. To identify potential novel lipid biomarkers, we performed lipid extraction on the right hemisphere of the brain and liquid chromatography mass spectrometry (LC/MS) to analyze the lipidomic profiling between non-infected mice and prion-infected mice. Finally, we analyzed the altered lipid-related pathways by a lipid pathway enrichment analysis (LIPEA).

**Results:** We identified a total of 43 and 75 novel potential biomarkers at 3 and 7 months in prion-infected mice compared to non-infected mice, respectively. Among these novel potential biomarkers, approximately 75% of total lipids are glycerophospholipids. In addition, altered lipids between the non-infected and prion-infected mice were related to sphingolipid, glycerophospholipid and glycosylphosphatidylinositol (GPI)-anchor-related pathways. In the present study, we found novel potential biomarkers and therapeutic targets of prion disease.

**Conclusions:** To the best of our knowledge, this study reports the first large-scale lipidomic profiling in prion diseases.

**Funded by:** National Research Foundation of Korea

**Grant number:** 2021R1A2C1013213, 2022R1C1C2004792

**Acknowledgement:** Yong-Chan Kim was supported by the BK21 plus program in the Department of Bioactive Material Sciences.

**Increasing incidence of Creutzfeldt-Jakob-disease in Austria – An epidemiological Update**

S. Klotz^a,b^, Günther Regelsberger^a,b^, Thomas Ströbel^a,b^, Romana Höftberger^a,b^, and Ellen Gelpi^a,b^

^a^Division of Neuropathology and Neurochemistry, Department of Neurology, Medical University of Vienna, Vienna, Austria; ^b^Austrian Reference Center for Human Prion diseases (Österreichisches Referenzzentrum zur Erfassung und Dokumentation menschlicher Prionen-Erkrankungen, ÖRPE)

**Aims**: To report epidemiological facts on human transmissible spongiform encephalopathies (TSEs) in Austria between 1996 and 2021.

**Material and Methods**: The Austrian Reference Centre for Human Prion Diseases (Österreichisches Referenzzentrum zur Erfassung und Dokumentation menschlicher Prionen-Erkrankungen) registers all cases of human TSEs in Austria since its establishment in 1996. The collected data was evaluated to give epidemiological information.

**Results**: Over 6800 CSF samples have been analyzed for 14-3-3 protein between 1996 and 2021. Since 2017 the Real-Time Quaking Induced Conversion (RT-QuIC) assay was added. The number of referrals for 14-3-3 protein analysis in CSF showed a constant increase since 1996 with a mean of 122 referrals per year from 1996 to 2008, and of 375 from 2009 until 2021. More than 700 brains were examined neuropathologically due to a suspicion of prion disease. The total number of definite human prion disease cases in Austria between 1996 and 2021 was 391. The average yearly incidence from 1996 until 2021 was 1.91 per million inhabitants. When comparing the average yearly incidence in the years from 1996–2008 and the more recent years from 2009 until 2021 we observed an increase of the yearly incidence from 1.49 to 2.33 cases per year. In 2021 the incidence was at an all times high with 4.14 cases per 1 million inhabitants per year. 92% of the cases were sporadic cases of Creutzfeldt-Jakob Disease (sCJD), 8% were genetic prion disease cases, and 5 cases of iatrogenic CJD have been registered. There are no cases of variant CJD in Austria. The mean age at death from 1996 to 2021 was 67.8 years. 51.41% of patients were female and 48.59% were male. Frequent alternative neuropathological diagnoses in suspect cases were Alzheimer’s disease, Lewy body disorders, vascular encephalopathy, metabolic encephalopathies and viral or limbic encephalitis. No false positive RT-QuIC results have been found.

**Conclusions**: The numbers of referrals for CSF analysis and also the yearly incidence of prion disease cases showed an increase over time between 1996 and 2021. This is likely due to an active surveillance of TSEs in Austria which is made possible by the good collaboration between the treating neurologists, pathologists and neuropathologists. Other contributing factors are an increase in awareness in the community and the implementation of the RT-QuIC assay. The peak of a yearly incidence of 4.14 cases per million inhabitants in 2021 needs further investigation.

**Funded by**: The **‘**Austrian Reference Center for Human Prion diseases’ (Österreichisches Referenzzentrum zur Erfassung und Dokumentation menschlicher Prionen-Erkrankungen) is funded by the Austrian Federal Ministry for Social Affairs, Health, Nursing and Consumer Protection

**Grant number: /**

**Acknowledgement**: We thank Prof. Gabor Kovacs and Prof. Herbert Budka, and all colleagues for their involvement in CJD surveillance.

**Scratch a downer cow: improving clinical diagnosis of atypical BSE in cattle**

T. Konold, L. Phelan, L. Read, A. McKenna, J. Hills, and H. Abdu

Department of Pathology & Animal Sciences, APHA Weybridge, Addlestone, UK

**Aims**: Atypical Bovine Spongiform Encephalopathy (BSE) is a supposedly spontaneous prion disease further classified as H-type or L-type BSE, which occurs sporadically in older cattle and is usually confirmed in fallen stock cattle. Studies to investigate tissue distribution of the agent are based on experimental disease because of the lack of reported clinical suspects in the field. Although the clinical presentation has been described in the past in experimentally inoculated cattle, the subsequent studies presented here aimed to find consistent clinical markers that may help farmers and veterinarians to consider atypical BSE in the suspect diagnosis of diseased cattle so that they can be presented for more detailed postmortem examination and tissue collection.

**Material and Methods**: In two separate experiments, two groups with two and three Holstein-Friesian calves respectively were each inoculated intracerebrally with H-type and L-type BSE brain inoculum and monitored clinically until clinical end-stage. Disease was confirmed by Western Immunoblot. A blood sample was collected from five animals for blood biochemistry.

**Results**: All cattle developed atypical BSE, confirmed by Western immunoblot, with a survival time of 14 months (H-type) and 15 months (L-type) respectively. Eight cattle displayed the nervous syndrome, and only two cattle (both L-type) developed the dull syndrome. Most consistent clinical signs were difficulty getting up (four of five H-type and all five L-type BSE cases) leading to recumbency in three cattle, ataxia/ dysmetria in all cattle and a positive scratch test (four of five H- and L-type BSE cases each, also in recumbent cattle) whereby scratching of the tail head resulted in head or lip movement. All five cattle (three H-type, two L-type), which had a blood sample taken, presented with a creatine kinase level above the upper normal limit of 211 IU/l.

**Conclusions**: Naturally occurring atypical BSE cases, if experimentally produced disease mimics natural disease, will present as ‘downer cows’, with animals unable to get up and creatine kinase levels likely to be increased suggestive of muscle trauma. Neurological gait abnormalities are likely to precede recumbency. In the absence of a clear cause, such as hypocalcaemia, and the presence of a positive scratch test, atypical BSE should be considered in the differential diagnosis of downer cows.

**Funded by**: Grant number: SE1961, SE1962

**Acknowledgement**: We thank the Department of Food, Environment and Rural Affairs for funding and staff at the Department of Pathology and Animal Sciences for inoculation, animal husbandry and tissue collection at necropsy.

**Aggregation and misassembly of the Disrupted-in-schizophrenia 1 (DISC1) protein defines a subset of patients with schizophrenia and recurrent affective disorders**

Carsten Korth

Department Neuropathology, University of Düsseldorf, Germany

**Aims**: To investigate the biological significance of protein aggregation and misassembly in major psychiatric diseases.

**Material and Methods**: Cell-based and cell-free techniques of recombinant protein expression. Immunological detection assays. Generation of a transgenic rat modestly overexpressing the full-length, non-mutant DISC1 protein (tgDISC1 rat), its behavioral, neurochemical, neuropathological and biochemical analysis. Analysis of post mortem brains and ante mortem cerebrospinal fluid from patients with schiophrenia and healthy controls with immunological techniques.

**Results**:

1. DISC1 aggregates were demonstrated in a subset of patients with schizopohrenia both ante mortem in cerebrospinal fluid (CSF) in fM concentrations, and in post mortem brains
2. the tgDISC1 rat models DISC1 protein aggregates and displays changes in dopamine homeostasis, behavioral and neuroanatomical changes consistent with schizophrenia
3. the tgDISC1 rat displays a immunological signature translatable to patients with schizophrenia.

**Conclusions**: Protein misassembly and aggregation is not limited to neurodegenerative diseases and can be, although in a subtle way, associated to chronic mental illness without causing massive cell death. DISC1 protein misassembly or aggregation is a specific feature for a subset of patients with schizophrenia („DISC1opathies“), and possibly other patients with recurrent affective disorders. The tgDISC1 rat is a valid model of this subset allowing more extensive direct translational studies in sporadic DISC1opathies and the development of causative pharmacological treatments.

**Funded by**: DFG (KO1679/3-1), BMBF (01EW1003), EU-FP7 (#607616), NARSAD (#20350)

**Acknowledgements**: Coauthors Svenja Trossbach, Marlene Pils, Oliver Bannach, Julia Rutsch, Dieter Willbold, Sophie Erhardt, Jospeh Huston, Ruter Leliveld, Philipp Ottis, An-Li Wang, Hannah Hamburg, Hans-Jürgen Bidmon, Peter Falkai, Andrea Schmitt, Ovidiu Popa, Philip Seeman, Laura Hecher, Verian Bader, Ingrid Prikulis, et al.

**Abeta dimers are antiprions that interfere with seeded nucleation *in vitro* and *in vivo***

Carsten Korth^a^, Else van Gerresheim^a^, Andreas Müller-Schiffmann^a^, Arne Herring^b^, Kathy Keyvani^b^, Lothar Gremer^c^, and Sandra Schäble^d^

^a^Departement Neuropathology, University of Düsseldorf, Germany; ^b^Department Neuropathology, University of Essen, Germany; ^c^Department of Physical Biology, University of Düsseldorf, Germany; ^d^Department of Comparative Psychology, University of Düsseldorf, Germany

**Aims**: To investigate the interaction of soluble Abeta oligomers with insoluble Abeta, and on Abeta prion spreading, in vitro and in vivo.

**Material and Methods**: The tgDimer mouse which is transgenic for Thy1-APPS679C/Swe and expresses exclusively Abeta S8C dimers was crossed to tgCRND8 mice, as well as to tgGFAP-luciferase mice. Mice were analyzed by immunohistochemistry (IHC), stereology and biochemistry (post mortem) as well as bioluminescence imaging (using luciferin) and behavior (CognitionWall; ante mortem). For cell-free in vitro experiments, Abeta42 wildtype and Abeta42 S8C were synthesized and purified, before subjection to a thioflavinT test.

**Results**:

1. Genetic crossing of tgDimer with tgCRND8 mice led to a decrease number of plaques whereas the size of the plaques remained similar.
2. Inoculation of tgDimer/GFAP-luc mice with Abeta prions purified from tgCRND8 mice did not lead to any evidence of Abeta prion spreading or astrogliosis as seen by absent bioluminescence or IHC, whereas the positive control, tgAPP23 mice, displayed Abeta prion spreading oafter inoculation with the same inoculum as previously established.
3. in the cell-free thioflavin T assay, Abeta S8C dimers inhibited Abeta wildtype aggregation at substochiometric concentrations.

**Conclusions**: Abeta S8C dimers inhibit nucleation/seeding of Abeta plaques, but not their growth. The variable interaction of Abeta dimers with insoluble Abeta can therefore be a contributing factor in the heterogeneity of Abeta neuropathology in Alzheimer’s disease. Abeta S8C dimers are homologous antiprions, adapting a concept established in the yeast prion field to mammalian prions. Likely, more homologous or heterologous antiprions exist that modulate actively the neuropathology and disease course of Alzheimer’s disease

**Funded by**: DFG KO1679 10–1

**Fast Axonal Transport of PrP^Sc^**

Sam M. Koshy^a^, Ronald A. Shikiya^a^, Anthony E. Kincaid^b^, and Jason C. Bartz^a^

^a^Medical Microbiology and Immunology, Creighton University, Omaha, NE, USA; ^b^Department of Pharmacy Science, School of Pharmacy Science and Health Professions, Creighton University, Omaha, NE, USA

**Aims**: Current studies indicate PrP^Sc^slow axonal transport based on immunohistochemistry, immunoblot analysis of tissue homogenates, detection of spongiosis, or the presence of prion infectivity using animal bioassay. A shortcoming of these methods is that they have low sensitivity and measure both inoculum PrP^Sc^and newly replicated PrP^Sc^, confounding the observed PrP^Sc^transport rates. In this study, we aim to:

1. Utilize highly sensitive PMCA to measure transport of inoculum PrP^Sc^in a replication deficient system to more accurately calculate PrP^Sc^transport rate.
2. Investigate the role of PrP^C^in PrP^Sc^axonal transport.

**Material and Methods**: Hamster-adapted 263 K scrapie PrP^Sc^was unilaterally inoculated into mouse sciatic nerve (ScN). At specified timepoints post-inoculation (p.i.), ScN, spinal cord (SC) segments, and midbrain were harvested. 10% tissue homogenates or HY TME brain homogenate dilutions were seeded into uninfected hamster or mouse brain substrate, underwent PMCA, and analyzed by immunoblot. The above tissue structures were dissected from an adult mouse and the distance between structures was determined. PrP^Sc^transport rate was determined based on the distance between structures and the timepoint of PrP^Sc^signal appearance in those structures.

**Results**: PMCA was able to detect down to 5 × 10^–12^μg eq. of HY hamster PrP^Sc^seed after two rounds while negative control unseeded PCMA reactions failed to amplify PrP^Sc^. After two PMCA rounds, we failed to detect PrP^Sc^in the uninoculated ScN, cervical SC, or midbrain at 24 hours p.i. We detected PrP^Sc^in the inoculated ScN, lumbar SC, and thoracic SC at 24 hours p.i. Based on a distance of 25 mm from the inoculation point to the lumbar SC, PrP^Sc^transport rate was calculated as greater than 25 mm/day.

**Conclusions**: PMCA is more sensitive than animal bioassay and in combination for its specificity for amplification of hamster PrP^Sc^allowed for detection of inoculum PrP^Sc^within tissues at earlier timepoints compared to previous studies. PMCA detection of PrP^Sc^in the lumbar SC at 24 hours p.i. indicates a rate of PrP^Sc^transport of at least 25 mm/day, well above established slow transport rates (0.3–8 mm/day). Based on this, we hypothesize that PrP^Sc^uses fast axonal transport, having implications for treatment and mitigation.

**Funded by**: NIH NINDS

**Grant number**: 1R01NS107246

**Acknowledgement**: We thank Creighton University Animal Resource Facility Staff for excellent animal care.

**Photodynamic inactivation of prions reduces infectivity in mouse bioassay but not seeding activity in RT-QuIC.**

Marie Kostelanska, Tibor Mosko, Zdenka Backovska Hanusova, and Karel Holada

Institute of Immunology and Microbiology, First Faculty of Medicine, Charles University in Prague, Czech Republic

**Aims**: Prions are the most resistant infectious agents to the conventional sterilization procedures. Effective techniques recommended for prion sterilization include highly corrosive chemicals like 2% NaClO or 1 M NaOH. These are incompatible with most delicate medical tools. We have previously demonstrated that nontoxic disulfonated hydroxyaluminium phtalocyanine (AlPcOH(SO_3_)_2_) can be used to inactivate prions by photodynamic inactivation (PDI). The aim of this study was to compare the effectiveness of prion inactivation in the mouse model and in RT-QuIC assay.

**Material and Methods**: The PDI was performed on 1% RML strain of prions or control CD1 mouse brain homogenates (BH) treated with 25 µg ml^−1^of AlPcOH(SO_3_)_2_ and exposed to red light (18 min, 4 × 2.5 W LED). Control RML inoculums was prepared similarly but was kept in the dark. In mouse bioassay, CD1 female mice (n = 10) were intracerebrally inoculated with a 25 µl of AlPcOH(SO_3_)_2_-light treated RML BH or control RML BH containing AlPcOH(SO_3_)_2_ but not irradiated by light. Other controls included mice (n = 5) inoculated with untreated, uninfectious CD1 BH or CD1 BH containing AlPcOH(SO_3_)_2_ either irradiated or nonirradiated. The animals were sacrificed after reaching the terminal disease phase. The presence of PrP^res^in mouse brains was detected by WB and seeding activity by second generation RT-QuIC assay using end point dilution.

**Results**: Mice inoculated with PDI-treated RML BH (at 10^–2^dilution) survived significantly longer (204 ± 23 days) than mice inoculated with the BH containing an identical amount of AlPcOH(SO3)_2_, that was not exposed to light (165 ± 11 days). Their survival was also significantly longer than that of mice inoculated with straight 10^–2^, 10^–3^and 10^–4^dilutions of the RML BH, which survived 169 ± 11, 165 ± 12 and 176 ± 15 days respectively. Comparison of survival time based on the regression line suggested a decrease in the prion infectivity titer of the RML BH of more than 4 orders of magnitude after PDI treatment. In contrast, seeding activity of PDI-treated RML BH in RT-QuIC assay decreased less than half log_10_. AlPcOH(SO3)_2_ did not have any effect on seeding activity of control CD1 or RML BH (light/dark).

**Conclusions**: We have proved the effectiveness of the PDI of prions by bioassay (4 log_10_ decrease) and by WB. The seeding activity in RT-QuIC assay did not correspond to decrease of infectivity or loss of PrP^res^analyzed by WB after PDI. This suggests that infectivity and seeding activity may not be the properties of prions tied strongly together.

**Funded by**: Czech health research council (AZV ČR)

**Grant number**: NV18-04-00179

**Detection of Chronic Wasting Disease Muscle Tissue by PMCA RT-QuIC**

Caitlyn N. Kraft^a^, David W. Bissinger^b^, Clare E. Hoover^c^, Nathaniel D. Denkers^a^, Candace K. Mathiason^a^, and Edward A. Hoover^a^

^a^Prion Research Center, Department of Microbiology, Immunology and Pathology, College of Veterinary Medicine and Biomedical Sciences, Colorado State University, Fort Collins, Colorado, USA; ^b^School of Veterinary Medicine, University of California, Davis, Davis, CA, US; ^c^AstraZeneca Inc., Waltham, Massachusetts, USA

**Aims**: Prion diseases affecting animals that are consumed by humans pose a significant public health risk – most notable being bovine spongiform encephalopathy, or Mad Cow Disease. Chronic Wasting Disease (CWD) affects moose, elk, and deer, all of which are consumed by humans. While evidence of CWD transmission to humans has not been demonstrated, establishing which muscles contain CWD prions is of high interest. Conventional assays, such as immunohistochemistry (IHC) and enzyme-linked immunosorbent assay (ELISA), are inadequate in detecting the low concentration of prions found outside neural or lymphatic tissues. Here we combined two prion amplification assays–protein misfolding cyclic amplification (PMCA) and real-time quaking induced conversion (RT-QuIC) to demonstrate the presence of prion seeding activity in muscles throughout the body of white-tailed deer.

**Material and Methods**: Hamstring and tongue were collected post-mortem from 31 CWD-inoculated white-tailed deer. Five additional muscles (neck, backstrap, tenderloin, calf, rump) were collected from a subset of deer (n = 7). All tissues were homogenized at 10% (w/v) in 1X PBS. For PMCA, 5 rounds of amplification were run using cervidized transgenic mice (Tg(CerPrP) 5037) normal brain homogenate as substrate. PMCA products were diluted in 0.1% SDS and analyzed by RT-QuIC. Samples were also analyzed by RT-QuIC, with and without an iron oxide bead (IOB) extraction. Truncated recombinant Syrian hamster prion protein (rhaPrP; 90–231) was used as substrate. In attempt to detect PrP^CWD^, IHC was performed on select samples using the BAR-224 antibody.

**Results**: Hamstring muscles were analyzed using PMCA/RT-QuIC where seeding activity was detected in 16 of 31 (51.6%) samples. Only 3 of 31 (9.7%) samples tested positive by IOB or standard RT-QuIC with no prion deposition detectable by IHC. Tongue from select deer was then analyzed by PMCA-RT-QuIC, which demonstrated prion seeding activity in 12 of 13 (92.31%) samples. IOB and standard RT-QuIC each detected positivity in 8 of 13 (61.5%) samples yet IHC remained negative. Other muscles analyzed by PMCA-QuIC revealed seeding activity in 4 of 7 (57.1%) backstrap and tenderloins, 3 of 7 (42.8%) rump, 2 of 5 (40%) calf, and 1 of 5 (20%) neck samples. Negative deer (n = 4) muscles remained negative in all assays.

**Conclusions**: We conclude that PMCA combined with RT-QuIC allowed for better detection of prions in the muscles of CWD-positive deer, with tongue exhibiting the most consistent seeding activity. Overall, this data provides added knowledge to the extent of CWD prion distribution within white-tailed deer muscles.

**Funded by**: This work was supported by the National Institutes of Health (NIH) grants RO1-NS061902-09 R to EAH, PO1-AI077774 to EAH, and R01-AI112956-06 to CKM

**Acknowledgement**: We abundantly thank Sallie Dahmes at WASCO and David Osborn and Gino D’Angelo at the University of Georgia Warnell School of Forestry and Natural Resources for their long standing support of this work through provision of the hand-raised, CWD-free, white-tailed deer used in these studies.

**Nasal swab detection of prion shedding in CWD-infected white-tailed**

Caitlyn N. Kraft, Nathaniel D. Denkers, Candace K. Mathiason, and Edward A. Hoover

Prion Research Center, Department of Microbiology, Immunology and Pathology, College of Veterinary Medicine and Biomedical Sciences, Colorado State University, Fort Collins, Colorado, USA

**Aims**: The continuing spread of chronic wasting disease (CWD) reinforces the pressing need for rapid detection of CWD prions shed by live cervids with minimally invasive methods. Prion replication in the nasal olfactory mucosa has been demonstrated by studies in deer, elk, and hamsters, yet the temporal profile of CWD prion shedding in nasal secretions has not been documented. Here we report a longitudinal study of nasal prion shedding using serial nasal swabs correlated to longitudinal tonsil biopsies and terminal tissues from white-tailed deer orally exposed to low doses of CWD prions.

**Material and Methods**: White tailed deer were inoculated per os (PO) with either 10 mg, 1 mg, or 300ng of CWD positive brain or saliva containing equivalent amounts of prion seeding activity assessed by real-time quaking induced conversion (RT-QuIC). Tonsil biopsies and nasal swabs were collected every 2 or 3 months post-inoculation throughout the duration of the study. Four swabs were collected at each timepoint (2 per nostril), and suspended in 1 mL 1X PBS at time of collection, and then spun to collect fluids and nasal mucosal cells. Samples were assayed by iron-oxide magnetic extraction and real-time quaking induced conversion (IOME RT-QuIC) using truncated Syrian hamster prion protein (rhaPrP; 90–231) as substrate. We correlated the RT-QuIC findings with the results of longitudinal tonsil biopsies and terminal tissue assays.

**Results**: We first detected nasal shedding of prion seeding activity 10 of the 18 deer (3–16 months; mean = 9.1) shortly after the first detection of RT-QuIC positivity in tonsil biopsies. Detectable nasal shedding persisted thereafter in 9 of the 10 animals. Nasal swabs remained negative in remaining 8 deer, even though all were CWD-infected as determined by tonsil biopsies and terminal tissue assays. Positive seeding activity was demonstrated in obex, frontal cortex, and olfactory bulb of all deer that had positive nasal swab results. Furthermore, deer near the endpoint of clinical disease shed more frequently. Nasal shedding frequency did not appear to vary among the narrow range of CWD prion dose administered. Control material inoculated deer (n = 2) remained negative in all assays at all time periods.

**Conclusions**: CWD prion seeding activity can be detected in nasal secretions from CWD infected deer using minimally invasive nasal swab sampling and IOME RT-QuIC. Nasal shedding of prion seeding activity occurred shortly after seeding activity was detected in tonsil in over half the animals studied, and became most consistent in later, near clinical phases of CWD infection when brain infection was present.

**Funded by**: This work was supported by the National Institutes of Health (NIH) grants RO1-NS061902-09 R to EAH, PO1-AI077774 to EAH, and R01-AI112956-06 to CKM.

**Acknowledgement**: We abundantly thank Sallie Dahmes at WASCO and David Osborn and Gino D’Angelo at the University of Georgia Warnell School of Forestry and Natural Resources for their long standing support of this work through provision of the hand-raised, CWD-free, white-tailed deer used in these studies.

**High resolution structures of infectious mammalian prions reveal a common prion fold**

Allison Kraus^a^, Forrest Hoyt^b^, Heidi G. Standke^a^, Efrosini Artikis^c^, Cindi L. Schwartz^b^, Bryan Hansen^b^, Kunpeng Li^a^, Andrew G. Hughson^c^, Matteo Manca^a^, Olivia R. Thomas^a^, Gregory J. Raymond^c^, Brent Race^c^, Gerald S. Baron^c^, Byron Caughey^c^

^a^Department of Pathology and Cleveland Center for Membrane and Structural Biology, Case Western Reserve University School of Medicine, Cleveland, OH, USA; ^b^Research Technologies Branch, Rocky Mountain Laboratories, National Institute of Allergy and Infectious Diseases, National Institutes of Health, Hamilton, MT, USA; ^c^Laboratory of Persistent Viral Diseases, Rocky Mountain Laboratories, National Institute of Allergy and Infectious Diseases, National Institutes of Health, Hamilton, MT, USA

**Aims**: Protein-based self-propagation is a shared characteristic of the disease-related misfolded proteins of prion, Alzheimer’s and Parkinson’s diseases, among others. High resolution structures are now available for disease-specific tau and -synuclein fibrils, and here, we describe high resolution structures of ex vivo and highly infectious PrP prions.

**Material and Methods**: We derived 263 K and aRML prions from the brains of clinically ill animals, and determined their titres using mouse bioassays. We used cryogenic-electron microscopy (cryo-EM) and helical reconstruction techniques to achieve high resolution cryo-EM maps with which to build de novo atomic models.

**Results**: High resolution structures of 263 K and aRML prion fibrils indicated surprisingly conserved organizational topologies with significant and strain-specific variability of motifs therein. Shared features between 263 K and aRML strains include the cross-section of the fibril being comprised of a single polypeptide backbone, with monomers being stacked 4.9 Å apart to form the parallel in register intermolecular -sheet fibril architecture. To accommodate the up to 137 amino acid sequence in the core, the polypeptide backbone is organized as a series of -sheets and loops arranged as three -arches, with a central -strand dividing N and C-terminal lobes. While a single monomer comprises the cross-section, variations in the planarity of the monomer leads to uneven exposed surfaces at the fibril ends where incoming monomers will be refolded. Additional densities were also observed outside the protein amyloid core for both 263 K and aRML prions, and outside of those attributable to glycan and glycolipid anchor placement in 263 K prions, being most often directly adjacent to cationic residues. The nature of these densities is as of yet unclear, however, such densities also occur adjacent to the polybasic cluster at amino acids 100–110, a region previously implicated as a potential cofactor coordination site, and critical modulator in the generation of de novo infectious prions.

**Conclusions**: Our evidence, together with other recent descriptions of murine prion structures, indicates that infectious prions are amyloids with large protease-resistant cores organized as parallel in register intermolecular -sheet architectures. Collectively, rodent prions share a common modular arrangement of their amyloid cores, with motif variations that are specific to the strain. This is suggestive that key structural domains of ex vivo prions are not inherently encoded in the occurrence of high -sheet, parallel in register assemblies – instead – key structural domains of disease causing prions underpin the replication and propagation characteristics of prion strains in ways that are linked to the divergent pathogenic outcomes.

**Funded by**: This work was supported by the Intramural Research Program of the NIAID; Mary Hilderman Smith, Zoë Smith Jaye, and Jenny Smith Unruh in memory of Jeffrey Smith; and the Britton Fund, and CWRU School of Medicine.

**Tau seeds precede earliest Alzheimer’s changes and are prevalent in synucleinopathies and other neurodegenerative diseases**

Matteo Manca^a*^, Heidi G. Standke^a*^, Mikayla L. Huntley^a^, Olivia R. Thomas^a^, Christina D. Orrú^b^, Andrew G. Hughson^b^, Yongya Kim^c^, Annie Hiniker^c^, David G. Coughlin^c^, Douglas Galasko^c^, and Allison Kraus^a^

^a^Department of Pathology, Case Western Reserve University School of Medicine, Cleveland, OH, United States 44,106; ^b^Rocky Mountain Laboratories, National Institute of Allergy and Infectious Diseases, National Institutes of Health, Hamilton, MT 59840; ^c^Department of Neurosciences, University of California San Diego

**Aims**: Neurofibrillary tau tangles are a hallmark of Alzheimer’s disease neuropathological change. However, it remains largely unclear how distinctive Alzheimer’s disease tau seeds (i.e. 3 R/4R) correlate with histological indicators of tau accumulation. Furthermore, AD tau co-pathology is thought to influence features and clinical progression of other neurodegenerative diseases including Lewy body disease; yet direct and reliable measurements of different types of tau seeds in such diseases is an unmet need.

**Material and Methods**: Here, we use tau real-time quaking-induced conversion (RT-QuIC) assays to selectively quantitate 3 R/4R tau seeds in the frontal lobe as a brain region that accumulates histologically identifiable tau pathology at late-stage disease.

**Results**: Seed quantitation across a spectrum of neurodegenerative disease cases and controls indicate tau seeding activity can be detected well before accompanying histopathological indication of tau deposits and even prior to the earliest evidence of Alzheimer’s related tau accumulation anywhere in the brain. In addition, Alzheimer’s tau seeds occur in all cases evaluated here inclusive of primary synucleinopathies, frontotemporal lobar degeneration and age-comparable controls albeit at multi-log lower levels than Alzheimer’s cases. α-synuclein seeding activity confirmed synucleinopathy cases and further indicated the co-occurrence of α-synuclein seeds in some Alzheimer’s disease and primary tauopathy cases. Our analysis indicates that tau seeds in the mid-frontal lobe correlate with the overall Braak tau stage, Alzheimer’s disease neuropathologic change, and frontal lobe digital quantitative immunohistochemistry measurements supporting the quantitative predictive value of tau RT-QuIC assays. Our data also indicates 3 R/4R tau seeds are elevated in female cases compared to males at higher (≥ IV) Braak stages, and that, in the cases of synucleinopathy, 3 R/4R tau seeding levels differ with Lewy body stage.

**Conclusions**: This study suggests 3 R/4R tau seeds are widespread even prior to the earliest stages of Alzheimer’s disease changes, including in normal individuals, and are prevalent across multiple neurodegenerative diseases as specific biomarkers to further define disease subtypes.

**Funded by**: This work was supported by Biomarkers Across Neurodegenerative Diseases (BAND), a partnership between the Alzheimer’s Association, The Michael J. Fox Foundation for Parkinson’s Research, The Weston Brain Institute, Alzheimer’s Research UK (to A.K., D.G.). D.C. is supported by NINDS NS120038 and D.C., D.G. by NIA AG062429, A.H. by an Alzheimer’s Association AACSF Research Grant and A.K. by BAND, NIH/NINDS and NIA (R01NS118760 and R01AG067607), Case Western Reserve University, the Britton Fund, and the Clifford V. Harding and Mina K. Chung Professorship in Pathology. This work was supported in part by the Intramural Research Program of the NIAID, NIH (C.D.O., A.G.H.).

**Grant number**: As indicated above under funding.

**Acknowledgement**: We thank the families and donors who have made this research possible; Jeff Metcalf at the UCSD Shiley-Marcos Alzheimer’s Disease Research Center for their assistance identifying and obtaining the tissue for this study; Dr. Lawrence Hansen for expert neuropathological characterization; Dr. Byron Caughey and Dr. Brent Race for the provision of tau knock-out brain tissue.

**Optimizing inactivation of CWD prions with humic acid**

Alsu Kuznetsova^b^, Isa Dzhabrailov^a,b*^, Anthony Ness^a,b^, Debbie McKenzie^a,b^, and Judd Aiken^b,c^

a Department of Biological Sciences, University of Alberta, Edmonton, Alberta, Canada; b Centre for Prions and Protein Folding Diseases, Edmonton, Alberta, Canada; c Department of Agricultural, Food and Nutritional Sciences, University of Alberta, Edmonton, Alberta, Canada * Presenting Author

**Aims**: Chronic wasting disease (CWD) is an environmentally transmissible prion disease of cervids. The shedding of CWD prions by infected animals presents a challenge for CWD management, as prions can contaminate the environment, remaining bioavailable for years. Soil decontamination protocols used to date are destructive and expensive, often requiring further land reclamation following the procedure, and not readily expandable to large swaths of land. We have shown that one of soil organic matter compounds, humic acids (HA), have anti-prion properties, making them capable of inactivating prions and reducing infectivity. Since the composition of humic substances is variable, we are investigating the biochemical properties of HA, and determining which may enhance prion inactivation. Our work helps elucidate the optimal conditions for CWD prion inactivation, allowing us to better understand the effect of HA outside of controlled laboratory conditions.

**Material and Methods**: CWD infected brain homogenates were incubated with a variety of HA solutions and PrP^CWD^levels detected by immunoblotting. We compared commercially available HA with HA extracted from different soil horizons for their ability to degrade CWD prions. HA was fractioned by molecular weight to determine if an HA fraction optimally inactivates prions. Prion inactivation was compared at varying temperatures and incubation lengths, both in solution and following prion adsorption to a surface.

**Results**: HA incubation with CWD brain homogenates reduces PrP^CWD^signal irrespective of temperature and whether PrP is adsorbed to soil or wood chips. The HA effect was immediate but increased with exposure times. Natural soils showed better prion inactivation, likely due to a higher carbon content, and our fractionation studies revealved higher molecular weight HA to be most efficient at inactivating PrP^CWD^.

**Conclusions**: Humic substances present a unique soil remediation strategy for CWD prion infected soil, providing the opportunity to inactivate prions while simultaneously improving soil properties as HA is commonly used in fertilizer. We demonstrate conditions which may serve to enhance HA prion inactivation. Our work helps understand the optimal conditions for CWD prion inactivation and may be a steppingstone for environmentally compatible interventions, aimed at reducing prion contamination of endemic soils.

**Funded by**: NFRFE**Grant number**: 2019–00943

**PrP^CWD^detection in soils from CWD endemic regions**

Alsu Kuznetsova^a,b^, Erin Moffat^b^, Trent Bollinger^b^, Debbie McKenzie^c^, and Judd M. Aiken^c^

^a^Department of Renewable Resources, University of Alberta, Edmonton, AB, Canada; ^b^University of Saskatchewan, Saskatoon, SK, Canada; ^c^Centre for Prions and Protein Folding Diseases, University of Alberta, Edmonton, AB, Canada

**Aims**: Chronic wasting disease (CWD) is a fatal, transmissible prion disease affecting free ranging and farmed cervids. One remarkable property of CWD prions is their persistence in external environments and their ability to remain infectious for years. Soils are a natural environmental reservoir of shed CWD prions. Prion detection in soils is challenging as recovery of soil-bound PrP^CWD^becomes more difficult with time. PrP^CWD^detection in soils is necessary for identifying CWD contaminated lands. The aim of this study is to develop a reliable method to detect PrP^CWD^in soils from CWD-endemic regions.

**Materials and Methods**: Soil samples were collected from CWD-endemic regions with high prevalence level of disease (South Saskatchewan, Canada); sites were distinguished as with or without recent deer activity based on camera monitoring. Prions were extracted from soils with an SDS-buffer and used as a seed in uninfected brain homogenates (from transgenic cervidized mice) for serial PMCA. After 4–5 rounds of PMCA, the amplification products were PK-digested and analyzed by immunoblot for PrP^CWD^. To define the sensitivity of the assay, dilutions of CWD infectious brain homogenates were spiked into soils (CWD-negative region). PrP^CWD^spiked into soil was detectable after 3 rounds of PMCA at 10^–6^dilution after 1-day of incubation with soil and at 10^–3^after 15-weeks of incubation with soil.

**Results**: Soils from the high prevalence CWD-endemic region (prairie Chernozems and alluvial soils) were collected near the stations for deer activity monitoring. A total of 15 soils from farmlands and natural landscapes sites with and without recent deer activity were sampled. PrP^CWD^was detected in 11 of the 15 soils sampled. Sites known to have recent deer activity accounted for most of the positive soils.

**Conclusions**: We can detect CWD prions from soils from CWD-endemic regions. This represents a significant improvement in soil-bound PrP^CWD^detection benefiting both surveillance and mitigation approaches.

**Funded by**: North Dakota Fish and Game Department, RDAR (#2022N067RC), APRI-AB Innovates (CWD-RP-2122007_30), Genome Canada and Genome Alberta

**Acknowledgement**: We thank our funding agencies for supporting this research

**Comparison of PMCA performance using identical sets of vCJD tissue homogentes spiked into blood components**

Kaetan Ladhani, and Jillian K Cooper

The National Institute for Biological Standards and Controls (NIBSC), Blanche Lane, SouthMimms, United Kingdon, EN6 3QG

**Aims**: Directly compare the performance of laboratories performing PMCA for the detection of vCJD in blood using a) Standard tissue homogenates b) Tissue homogenates spiked into normal donor blood components.

Assess the impact of different seed concentration steps used by laboratories on assay sensitivity and specificity.

Investigate different methods used for seed concentration ‘in house’.

**Material and Methods**: We have generated standards from vCJD tissues (brain and spleen) that have been widely used for the assessment of methods used to detect PrPSc. Using non-UK sourced blood spiked with serially diluted vCJD tissues, provided blinded to tester, we have established test sensitivity with these common sample sets. A large number of individual normal blood donor blood components have been generated for the assessment of specificity.

Different approaches are used for initial concentration of the seed material used for the PMCA assay. We have compared these both externally through the analysis of spiked panels as well as in house.

**Results**: Using panels of vCJD tissues spiked into blood components we have independently assessed the performance of laboratories using PMCA for the detection of vCJD and compared sensitivity to laboratories using direct detection methods.

**Conclusions**: Standard sample sets allow direct comparison of method performance and play an important role in monitoring the progress of method establishment. These reagents are available to laboratories for independent assessment.

**Funded by:/Grant number**: National Institute for Health Research Policy Research Programme Project PR-R17-0916-13,007

**Second passage of scrapie in white-tailed deer is discernable from chronic wasting disease.**

Zoe J. Lambert^a,b,c^, M. Heather West Greenlee^a^, Eric D. Cassmann^b^, and Justin J. Greenlee^b^

^a^Department of Biomedical Sciences, Iowa State University College of Veterinary Medicine, Ames, USA; ^b^Virus and Prion Research Unit, National Animal Disease Center, Agricultural Research Service, United States Department of Agriculture, Ames, USA; ^c^Oak Ridge Institute for Science and Education, Oak Ridge, USA

**Aims**: White-tailed deer (WTD) are susceptible to the scrapie agent from sheep after oronasal inoculation. Upon initial passage from sheep, 100% of inoculated deer developed disease. Western-blot profiles were tissue dependent: profiles from cerebrum and retina were similar to the scrapie inoculum, whereas brainstem and lymph node appeared CWD-like. The purpose of this study was to examine how the agent of scrapie in WTD may adapt following subsequent passage in WTD and determine if it can be differentiated from CWD.

**Material and Methods**: The inoculum was brain homogenate from a WTD that was challenged with classical scrapie from a sheep (ARQ/ARQ genotype) and developed clinical signs at 32.5 months post inoculation. Three WTD received 1 mL of 10% w/v obex inoculum oronasally, four received 1 mL of 10% w/v cerebrum intranasally. The negative control animal received no inoculum. Deer were euthanized and necropsied following the development of clinical signs, including weight loss, hair loss, excessive salivation, diarrhea, and progressive weakness. Enzyme immunoassay (IDEXX), western blot, and immunohistochemistry using the primary antibodies Sha 31, 12B2 and F99 were used. Cervidized mice (Tg12) were inoculated with material from WTD in each group.

**Results**: All inoculated WTD displayed clinical disease and were positive for abnormal prion protein by enzyme immunoassay. The average survival time of the obex-inoculated group was 24.1 months post-inoculation, while that of the cerebrum-inoculated group was 29.2 months post-inoculation. Both groups had shorter incubation times compared to the inoculum donor (32.5 months). Western blotting of retinas from all WTD (second pass) resulted in a similar molecular profile as the retinas of WTD that were inoculated with the agent of scrapie from sheep (first pass). Immunohistochemical staining also was similar between inoculation groups and the initial passage from sheep, but different from WTD inoculated with the agent of CWD. Following bioassays in cervidized mice (Tg12), all incubation periods were over 300 days, substantially longer than the approximately 150 day incubation period typical with CWD isolates.

**Conclusions**: Based upon analysis of retinal tissues, it is possible to differentiate the agents of scrapie and CWD in WTD by both western blot and immunohistochemistry. Bioassay in cervidized mice further supports this based on incubation periods of WTD-scrapie being approximately twice that of WTD CWD.

**Funded by**: This research was funded in its entirety by congressionally appropriated funds to the United States Department of Agriculture, Agricultural Research Service. The funders of the work did not influence study design, data collection and analysis, decision to publish, or preparation of the manuscript. This research was supported in part by an appointment to the Agricultural Research Service (ARS) Research Participation Program administered by the Oak Ridge Institute for Science and Education (ORISE) through an interagency agreement between the U.S. Department of Energy (DOE) and the U.S. Department of Agriculture (USDA). ORISE is managed by ORAU under DOE contract number DE-SC0014664. All opinions expressed in this paper are the author’s and do not necessarily reflect the policies and views of USDA, ARS, DOE, or ORAU/ORISE.

**Grant number**: DOE contract number DE-SC0014664

**Doxycycline rescues recognition memory and circadian motor rhythmicity but does not prevent terminal disease in fatal familial insomnia mice**

Giada Lavigna^a^, Antonio Masone^a^, Ihssane Bouybayoune^a^, Ilaria Bertani^a^, Jacopo Lucchetti^b^, Marco Gobbi^b^, Luca Porcu^c^, Stefano Zordan^d^, Mara Rigamonti^d^, Luca Imeri^e^, Elena Restelli^a^, and Roberto Chiesa^a^

^a^Department of Neuroscience, Istituto di Ricerche Farmacologiche Mario Negri, Milan, Italy; ^b^Department of Molecular Biochemistry and Pharmacology, Istituto di Ricerche Farmacologiche Mario Negri, Milan, Italy; ^c^Department of Oncology, Istituto di Ricerche Farmacologiche Mario Negri, Milan, Italy; ^d^Tecniplast SpA, Buguggiate, Italy; ^e^Department of Health Sciences, University of Milan, Milan, Italy

**Aims**: Fatal familial insomnia (FFI) is a dominantly inherited prion disease linked to the D178N mutation in the gene encoding the prion protein (PrP). Symptoms, including insomnia, memory loss and motor abnormalities, appear around 50 years of age, leading to death within two years. No treatment is available. A ten-year clinical trial of doxycycline (doxy) is under way in healthy individuals at risk of FFI to test whether presymptomatic doxy prevents or delays the onset of disease. To assess the drug’s effect in a tractable disease model, we used Tg(FFI-26) mice, which accumulate aggregated and protease-resistant PrP in their brains and develop a fatal neurological illness highly reminiscent of FFI.

**Material and Methods**: Mice were treated daily with 10 mg/kg doxy starting from a presymptomatic stage for twenty weeks. Motor function was assessed periodically by the beam walking and accelerating rotarod tests; memory was assessed by the novel object recognition test; onset and progression of neurological illness were scored with a set of objective criteria; circadian motor activity was measured using Digital Ventilated Cages. Detergent insolubility and protease resistance were used to assess the effect of the treatment on the biochemical properties of mutant PrP. Immunohistochemistry was used to assess gliosis.

**Results**: Doxy rescued memory deficits and restored circadian motor rhythmicity in Tg(FFI-26) mice. However, it did not prevent the onset and progression of motor dysfunction, clinical signs and progression to terminal disease. Doxy did not change the amount of aggregated and protease-resistant PrP, but reduced microglial activation in the hippocampus.

**Conclusions**: Presymptomatic doxy treatment rescues cognitive impairment and the motor correlates of sleep dysfunction in Tg(FFI-26) mice but does not prevent fatal disease.

**Funded by**: Fondazione Telethon Italy, the EC and the Italian Ministry of Health.

**Grant number**: GGP10208, ERARE14-fp-097 – CHAPRION, RF-2016-02362950

**Characterizing inhibitory effects of metal ions on CWD prion amyloid formation using RT-QuIC**

Manci Li^a,b^, and Peter A. Larsen^a,b^

^a^Minnesota Center for Prion Research and Outreach, University of Minnesota, St. Paul, MN 55108, USA; ^b^Department of Veterinary and Biomedical Sciences, University of Minnesota, St. Paul, MN 55108, USA

**Aims**: Chronic Wasting Disease (CWD) is an emerging infectious prion disease with zoonotic potential and is now found in several Nordic countries, South Korea, Canada, and 27 U.S. states. CWD is caused by misfolded prion proteins (PrP^CWD^) that are resistant to degradation due to their β-sheet-rich structures (amyloids). PrP^CWD^can induce native cellular prion protein (PrP^C^) to adopt the misfolded conformation through direct contact. PrP^C^participates in maintaining metal homeostasis in mammals; for example, PrP^C^can mediate the internalization of both zinc and copper. Several papers have identified links between copper availability and CWD disease incidence and progression. Real-time quaking-induced conversion (RT-QuIC) is a highly sensitive assay to detect PrP^CWD^seeding activity in vitro. In addition to assisting future diagnostics of protein misfolding diseases, RT-QuIC has been suggested for drug pre-screening, especially in assessing the ability of compounds to inhibit amyloid formation. Among many candidates for controlling CWD in cervids, nutritional additives with metal ions known to interact with PrP^C^and have enhanced cellular uptake, such as copper amino acid complex (Cu-AA) and zinc amino acid complex (Zn-AA), are ideal candidates to explore potential inhibitory effects on prion amyloid formation. Moreover, effective delivery of such nutritional additives can be easily achieved through cervid feed. Here, we aim to investigate the potential impact that Cu-AA and Zn-AA have on amyloid formation in vitro using RT-QuIC.

**Material and Methods**: We adapted RT-QuIC to characterize the inhibitory effects of Cu-AA and Zn-AA on CWD prion amyloid formation in RT-QuIC and compared such effects with those produced by metals salts alone.

**Results**: We found that Cu-AA and Zn-AA can more readily inhibit amyloid formation in vitro than metal salts and the timing of addition affects the effectiveness of inhibitory effects.

**Conclusions**: Our results suggest that metal ion additives may exhibit inhibitory effects on amyloid formation through interactions with PrP^C^. Future research efforts aimed at advancing therapeutics for prion diseases using metal complexes are needed.

**Funded by**: Lyssy & Eckel Feeds

**Acknowledgement**: We would like to thank Zinpro Corporation for providing us with Performance Minerals®.

**Influence of Cobalamin levels on Prion protein expression**

Ewald Lindner

Medical University Graz, Graz, Austria

**Aims**: In animal models it has been demonstrated that cobalamin deficiency increases normal cellular prion levels in spinal cord and cerebrospinal fluid. Here we have investigated whether cobalamin levels affect the expression of prion protein in humans.

**Material and Methods**: Patients presenting to the department of ophthalmology of the Medical University of Graz for reasons unrelated to prion diseases were enrolled. Cobalamin was measured by routine laboratory tests. Surface prion protein on CD14+ monocytes, CD8+ and CD4 + T cells was analyzed by fluorescence activated cell sorting.

**Results**: 85 patients were enrolled. Serum Cobalamin levels correlated significantly with prion protein levels on CD14+ POM1+ monocytes (r = −0.23, p = 0.036), CD8+ POM1+ (r = −0.38, p = 0.001) and CD4+ POM1 + T cells (r = −0.40, p < 0.001).

**Conclusions**: Our findings suggest an association between cobalamin levels and prion protein expression in humans.

**Funded by**: No funding

**De-repression of endogenous retroviruses promotes prion-like spreading of proteopathic seeds**

S. Liu^a,b*^, S.-E. Heumüller^a*^, A. Hossinger^a^, S.A. Müller^c^, O. Buravlova^a^, S.F. Lichtenthaler^c,d,e^, P. Denner^a^, and I.M. Vorberg^a,f^

^a^German Center for Neurodegenerative Diseases Bonn (DZNE), Bonn, Germany; ^b^Present address: German Federal Institute for Risk Assessment (BfR), German Centre for the Protection of Laboratory Animals (Bf3R), Berlin, Germany; ^c^German Center for Neurodegenerative Diseases (DZNE), Munich, Germany; ^d^Neuroproteomics, School of Medicine, Klinikum rechts der Isar, Technical University of Munich, Germany; ^e^Munich Cluster for Systems Neurology (SyNergy), Munich, Germany; ^f^Rheinische Friedrich-Wilhelms-Universität Bonn, Germany

*Contributed equally to this manuscript

**Aims**: Endogenous retroviruses, remnants of viral germline infections, make up a substantial proportion of the mammalian genome. While usually epigenetically silenced, retroelements can become upregulated in neurodegenerative diseases, such as amyotrophic lateral sclerosis and tauopathies. Here we investigate how de-repression of endogenous retroviruses affects the prion-like spreading of misfolded proteins between cells.

**Material and Methods**: We use murine and human cell models to study the dissemination of proteopathic seeds composed of a yeast prion domain or human Tau. Methods include high-content cell-based assays and proteomics.

**Results**: We demonstrate that spontaneous upregulation of endogenous retrovirus gene expression drastically affects the dissemination of protein aggregates between cells in culture. Viral glycoprotein Env mediates membrane association between donor and recipient cells and also promotes the intercellular transfer of protein aggregates packaged into extracellular vesicles. Proteopathic seed spreading can be inhibited by neutralizing antibodies targeting Env as well as drugs inhibiting viral protein processing. Importantly, we show that also overexpression of a human endogenous retrovirus Env elevates intercellular spreading of pathological Tau.

**Conclusions**: Our data highlight the potential influence of endogenous retroviral proteins on protein misfolding diseases and suggest that antiviral drugs could represent promising candidates for inhibiting protein aggregate spreading.

**Funded by: Funding information**

This work was funded by the Helmholtz Portfolio ‘Wirkstoffforschung’, the ‘Deutsche Forschungsgemeinschaft’ (DFG, German Research Foundation) under Germany’s Excellence Strategy within the framework of the ‘Munich Cluster for Systems Neurology’, by the German Ministry for Education and research. The funders had no role in study design, data collection and analysis, decision to publish, or preparation of the manuscript. S. Liu, S.A. Müller, S.F. Lichtenthaler, P. Denner and I.M. Vorberg hold pending patent applications for ‘HERV inhibitors for use in treating tauopathies’: “US Patent Application No. 17/640,119 based on PCT International application No. PCT/EP2020/074809, claiming priority to ‘European Application No. 19,195,304.1’.

**Grant numbers**: EXC 2145 SyNergy – ID 390857198, CLINSPECT-M (FKZ161L0214C) and JPND PMG-AD (01ED2002B)

**Acknowledgement**: We thank Leonard Henry Evans for generously sharing anti-MLV antibodies. The light microcopy (LMF) and laboratory automation facilities (LAT) of the DZNE Bonn were used for image acquisition.

**A non-PrP^Sc^ PrP prion**

Nuria López-Lorenzo^a^, Davy Martin^b^, Enric Vidal^c^, Sonia Veiga^a^, Maite Freire Delgado^a^, Yaiza B.Codeseira^a^, Vincent Beringue^b^, Human Rezaei^b^, and Jesús R. Requena^a^

^a^CIMUS Biomedical Research Institute & Department of Medicine, University of Santiago de Compostela-IDIS, Santiago de Compostela, Spain; ^b^INRAE, UVSQ, VIM, Université Paris-Saclay, Jouy-en-Josas, France; ^c^Centre de Recerca en Sanitat Animal (CReSA) – Institut de Recerca i Tecnologia Agroalimentàries (IRTA), Campus de UAB, 08193 Bellaterra, Barcelona, Catalonia.

**Aims**: Prions are responsible for transmissible spongiform encephalopathies, also known as prion diseases. The most notorious prion is PrP^Sc^, nonetheless, prion protein (PrP) amyloids can also cause prion diseases, such as the N-terminal amyloid 145Stop, which is responsible for Gerstmann-Straussler-Scheinker (GSS) disease.

While infectivity is the *sine qua non* condition to define a *bona fide* prion, some recombinant full-length amyloids also propagate and accumulate in the brain but cannot cause clinical signs, at least in the first passage.

Recently some of us have shown that full-length PrP amyloids are infectious to transgenic (Tg)Tg7 mice.

**Material and Methods**: Here Tg mice expressing ~1x of the bank vole I109 PrP sequence were intracerebrally inoculated with a bank vole 23–231 PrP amyloid.

**Results**: All the mice developed clinical signs consisting of ataxia, kyphosis, lethargy and body weigh lost with an average incubation time of 198 ± 8 days. Brains of clinically sick mice accumulate PK-resistant PrP fragments of ~20, ~16 and ~13 kDa. These fragments, where recognized by the C-terminal antibody SAF84 (160–170), but not by SAF83 (126–164). Moreover, when brain homogenates were treated with PNGaseF, all the mentioned bands collapsed in a ~ 13 kDa band, corresponding to the ~9.5 kDa PK-resistant amyloid core plus the GPI anchor.

**Conclusions**: These results demonstrate the existence of an infectious recombinant full-length PrP amyloid that has an attack rate of 100% in its first passage in a ~ 1x expression model and presents period of incubation even shorter than some recombinant PrP^Sc^strains.

In view of the recent results of cryo-EM, showing that PrP^Sc^and PrP amyloids share a PIRIBS architecture, it is no wonder that both conformers can be infectious, despite their different size. Thus, PrP amyloids must be also considered *bona fide* prions.

**Funded by:**

**Grant number**: This study was funded by research grants MINECO (PID2020-117465GB-I00) and the Spanish CJD Association (Sergio Rubio).

**Identification of biomarkers associated with endoplasmic reticulum stress and proteasome impairment in natural scrapie**

Jenny Lozada, Marina Betancor, Sonia Pérez Lázaro, Rosa Bolea, Juan J. Badiola, and Alicia Otero

Centro de Encefalopatías y Enfermedades Transmisibles Emergentes, Universidad de Zaragoza-IA2, Zaragoza, Spain

**Aims**: The accumulation of misfolded proteins such as PrP^Sc^can alter endoplasmic reticulum homeostasis triggering the unfolded protein response (UPR). In this pathogenic event, the molecular chaperones play an important role and are overexpressed in prion diseases. Several reports in humans and animals have suggested that neurodegeneration is related to endoplasmic reticulum stress in diseases caused by the accumulation of misfolded proteins.

These proteins may contribute as biomarkers of ER stress in prion diseases.

**Material and Methods**: In this study, we investigated the expression of three endoplasmic reticulum stress markers: PERK (endoplasmic reticulum kinase), BiP (binding immunoglobulin protein) and PDI (Protein Disulfide Isomerase). In addition, we evaluated the accumulation of ubiquitin as a marker for protein degradation mediated by the proteasome. These proteins were studied by immunohistochemistry and western blot in brain tissue of sheep affected by natural scrapie in clinical and preclinical stages of the disease. Protein accumulation was semiquantitatively evaluated. Results were compared with those observed in healthy controls.

**Results**: PERK accumulation was more intense in clinical and preclinical sheep compared with healthy animals. PERK immunolabeling revealed significant differences between groups. The thalamus and hippocampus were the regions showing the most intense PERK accumulation in clinical sheep. BiP expression levels were higher in clinical and preclinical sheep compared with controls in several brain areas. PDI was the most altered marker. Scrapie-infected sheep showed significantly higher levels of PDI than healthy animals in almost all evaluated brain areas.

Significantly increased intraneuronal and neuropil ubiquitinated deposits in the form of granules were observed in certain brain areas such as the obex and hippocampus in scrapie-affected animals compared to controls.

**Conclusions**: Our results suggest that the neuropathological and neuroinflammatory phenomena that develop in prion diseases cause endoplasmic reticulum stress in brain cells triggering the UPR. In addition, the significantly higher accumulation of ubiquitin aggregates in scrapie-affected animals suggests an impairment of the ubiquitin-proteasome system in natural scrapie.

**Funded by:/ Grant number**: RTI2018-098711-B-I00, POCTEFA EFA148/16

**Widespread search for potentially protective prion protein variants in the Icelandic sheep population delivers promising results**

Gesine Lühken^a^, Karólína Elísabetardóttir^b^, Eyþór Einarsson^c^, Vilhjálmur Svansson^d^, and Stefanía Thorgeirsdottir^d^

^a^Department of Animal Breeding and Genetics, Justus-Liebig University of Giessen, Giessen, Germany; ^b^Hvammshlíð, Skagafjörður, Iceland; ^c^Icelandic Agricultural Advisory Centre, Sauðárkrókur, Iceland; ^d^Department of Virology and molecular biology, Institute for Experimental Pathology at Keldur, University of Iceland, Reykjavík, Iceland

**Aims**: Breeding for scrapie resistance in Iceland has not been a real option, since ARR, the main protective allele combination, has in the past not been found in the Icelandic sheep breed (1). Instead, culling whole scrapie flocks has been the main approach to fight this disease in Iceland, with additional means of cleaning of sheep pens and a sheep free period of 2–3 years. The aim of this research project was to search for potential scrapie protective prion protein variants in a large group of Icelandic sheep originating from different regions of the country, with a specific attention to areas which had not been affected by large scale culling of sheep as part of an eradication program against various imported infectious diseases in the past.

**Material and Methods**: Ear tag or brain samples were collected from ca. 17,000 sheep, DNA isolated and the full coding region of the prion protein gene (*PRNP*) sequenced, or genotyped at codons 136, 137, 138, 151, 154, 171 by pyrosequencing.

**Results**: Unexpectedly, 14 sheep (0.08%), carrying ARR, were found at one farm in the far eastern part of Iceland. In addition, a potentially protective polymorphism at codon 137 (T137), was found in 39 sheep (0.23%) from a total of seven farms located in different regions of the country. The T137 polymorphism, found earlier at a low frequency in Icelandic sheep, has been shown to be protective against scrapie in Sarda sheep in Italy (2).

**Conclusions**: Although found at a very low frequency, the presence of ARR and T137 offer the possibility of a careful breeding program for scrapie resistance in the Icelandic sheep breed, the best by including deep pedigree and genomic data in order not to decrease the diversity of the population. Especially, the use of sheep with T137 is promising, because in contrast to ARR, they are not all from one ancestry. An ongoing project should prove the protective effect of the identified variants against the Icelandic scrapie strains, e.g. with the PCMA and RT-QuIC tests. The number of scrapie cases has drastically decreased since the height of the epidemic in the 1980s, but there are still a few cases detected each year, mostly in the North. That area should therefore be targeted first for breeding for resistance.

**References**: (1) Thorgeirsdottir et al. 1999. J. Gen. Virol. 80: 2527–2534 (2) Vaccari et al. 2009. Vet. Res. 40:19

**Funded by**: Local fund for development of sheep farming in Iceland.

**Mortality surveillance of persons potentially exposed to chronic wasting disease**

R.A. Maddox^a^, R.F. Klos^b^, L.R. Will^b^, S.N. Gibbons-Burgener^b^, A. Mvilongo^a^, J.Y. Abrams^a^, B.S. Appleby^c^, L.B. Schonberger^a^, and E.D. Belay^a^

^a^National Center for Emerging and Zoonotic Infectious Diseases, Centers for Disease Control and Prevention (CDC), Atlanta, USA; ^b^Wisconsin Department of Health Services (WDHS), Division of Public Health, Madison, USA; ^c^National Prion Disease Pathology Surveillance Center (NPDPSC), Case Western Reserve University, Cleveland, USA

**Aims**: It is unknown whether chronic wasting disease (CWD), a prion disease of cervids, can infect people, but consumption of meat from infected animals would be the most likely route of transmission. Wisconsin Department of Health Services, Division of Public Health (WDHS) personnel maintain a database consisting of information collected from hunters who reported eating, or an intention to eat, venison from CWD-positive cervids. These data, collected since 2003, allow for the evaluation of causes of mortality in individuals potentially exposed to CWD.

**Material and Methods**: The WDHS database contains the name, date of birth, when available, year of CWD-positive deer harvest, and city and state of residence for each potentially exposed individual. The database also includes information on how the deer was processed (self-processed or by a commercial operator) and when applicable, names of others with whom the venison was shared. Duplicate entries (i.e., those who consumed venison from CWD-positive deer in multiple hunt years) are determined by first name, last name, and date of birth. All names in the database are cross-checked with reported cases of human prion disease in Wisconsin and cases in the National Prion Disease Pathology Surveillance Center (NPDPSC) diagnostic testing database. Persons with date of birth available are also cross-checked with prion disease decedents identified through restricted-use national multiple cause-of-death data via a data use agreement with the National Center for Health Statistics (NCHS).

**Results**: The database currently consists of 1561 records for hunt years 2003–2017 and 87 additional records for 2018–2019. Of these, 657 records have accompanying date of birth; 15 entries were removed as duplicates leaving 642 unique individuals. Of these individuals, 278 of 426 (66%) who ate venison from a CWD-positive deer and provided processing information reported self-processing. No matches were found among any persons in the database cross-checked with WDHS human prion disease surveillance data, NPDPSC data (February 2022 update), and NCHS data through 2020.

**Conclusions**: Because of the linkage of person and CWD-positive animal in the WDHS database, reviewing the cause of mortality in potentially exposed persons is possible. The number of individuals cross-checked so far is likely only a small percentage of those potentially exposed to CWD in Wisconsin, and many more years of vital status tracking are needed given an expected long incubation period should transmission to humans occur. Nevertheless, the findings of this ongoing review are thus far reassuring.

**Prion disease incidence, United States, 2003–2020**

R.A. Maddox^a^, M.K. Person^a^, K. Kotobelli^b^, A. Mvilongo^a^, B.S. Appleby^b^, L.B. Schonberger^a^, T.A. Hammett^a^, J.Y. Abrams^a^, and E.D. Belay^a^

^a^National Center for Emerging and Zoonotic Infectious Diseases, Centers for Disease Control and Prevention (CDC), Atlanta, USA; ^b^National Prion Disease Pathology Surveillance Center (NPDPSC), Case Western Reserve University, Cleveland, USA

**Aims**: Mortality data, in conjunction with neuropathological and genetic testing results, are used to estimate prion disease incidence in the United States.

**Material and Methods**: Prion disease decedents for 2003–2020 were identified from restricted-use U.S. national multiple cause-of-death data, via a data use agreement with the National Center for Health Statistics, and from the National Prion Disease Pathology Surveillance Center (NPDPSC) database. NPDPSC decedents with neuropathological or genetic test results positive for prion disease for whom no likely match was found in the NCHS multiple cause-of-death data were added as cases for incidence calculations, while those with negative neuropathology results but with cause-of-death data indicating prion disease were removed. Unmatched cases in the NPDPSC database lacking neuropathological testing but with a positive real-time quaking-induced conversion (RT-QuIC) test result were additionally assessed. Age-specific and age-adjusted average annual incidence rates were calculated from the combined data; the year 2000 as the standard population and the direct method were used for age-adjustment.

**Results**: A total of 7,921 decedents were identified as having prion disease during 2003–2020 for an age-adjusted average annual incidence of 1.2 per million population. The age-adjusted incidence between males and females (1.3 and 1.1 per million, respectively) differed significantly (p < 0.0001). The age-specific average annual incidence among those <55 and ≥55 years of age was 0.2 and 4.8 per million, respectively; incidence among those ≥65 was 6.1 per million. Eighteen cases were <30 years of age for an age-specific incidence of 8.0 per billion; only 6 of these very young cases were sporadic (3 sporadic CJD, 3 sporadic fatal insomnia), with the rest being familial (9), variant (2), or iatrogenic (1). The age-adjusted annual incidence for the most recent year of data, 2020, was 1.3 per million. However, assessment of RT-QuIC positive cases lacking neuropathology in the NPDPSC database suggested that approximately 20% more cases may have occurred in that year; the addition of a subset of these cases that had date of death information available (n = 44) increased the 2020 rate to 1.4 per million.

**Conclusions**: Mortality data supplemented with the results of neuropathological, CSF RT-QuIC, and genetic testing can be used to estimate prion disease incidence. However, the identification in the NPDPSC database of RT-QuIC-positive cases lacking date of death information suggests that this strategy may exclude a number of probable prion disease cases. Prion disease cases <30 years of age, especially those lacking a pathogenic mutation, continue to be very rare.

**Biomarker-driven phenotyping for Alzheimer’s disease and related dementia**

Nour Majbour^a^, Zane Jaunmuktane^b^, Sebastian Brandner^b^, John Collinge^a,c^, Simon Mead^a,c^

^a^UCL Institute of Prion Diseases, MRC Prion Unit at UCL, London, UK; ^b^Division of Neuropathology, UCL Queen Square Institute of Neurology, and The National Hospital for Neurology and Neurosurgery, University College Hospitals NHS Foundation Trust, Queen Square, London; ^c^National Hospital for Neurology and Neurosurgery, University College London Hospitals NHS Foundation Trust, National Prion Clinic, London, UK

**Aims**: Structural transformation of certain proteins from the native conformation to β-sheet-rich pathogenic conformation, often in aggregated form, has been implicated in many neurodegenerative diseases. In patients with a clinical diagnosis of Alzheimer’s disease dementia, such structural transition is thought to occur initially with amyloid-beta and subsequently tau proteins, but is commonly also seen at post-mortem examination in alpha-synuclein (αSyn) and TAR DNA binding protein 43 (TDP-43) proteins, resulting in a more complex situation that doesn’t easily align with canonical clinical diagnoses. Dementia conceptualised as a multi-proteinopathy might also explain the failure of therapeutic approaches that target just one misfolded protein. Therefore, detection and quantitation of minute quantities of multiple protein assemblies with specific beta-sheet content (or proteopathic ‘seeds’) in human biofluids, would be a logical approach towards more personalized diagnosis. Here, we establish seed amplification- and immuno- assays that allow rapid and specific quantitation of disease-associated misfolded protein seeds in Alzheimer’s and related dementia.

**Material and Methods**: We present the development of a panel of multi-protein quantitative in vitro seed amplification assays against the proteins Tau, αSyn, and TDP-43. We then explore the diagnostic power of the panel in a cohort of post-mortem human brain tissues, including tissues of frontal and temporal cortices from cases with Parkinson’s disease, dementia with Lewy bodies, frontotemporal lobar degeneration, and Alzheimer’s disease (with/without αSyn and TDP-43 pathology) (n = 5 per group). We also investigate potential correlations between biomarkers and pathology data.

**Results**: We have developed quantitative seed amplification assays (SAAs) that preferentially detect full-length αSyn or 3 R/4R tau aggregates in human CSF and brain tissues. Low amounts of given self-propagating protein aggregate (the seed) were robustly quantitated using ELISA-post-SAA inferring more information about the initial seed quantity compared to SAA kinetics alone. Our analysis also showed a correlation between seeding kinetics and disease stage assessed on histopathologic scoring system. Along with amyloid beta and TDP-43 immunoassays, our multi-protein panel strongly highlighted the overlooked heterogeneity in subjects with clinical diagnosis of Alzheimer’s disease, suggesting the need for more personalised diagnostic approaches.

**Conclusions**: Our findings provide deeper understanding of the clinico-pathological associations of common neuropathologies in Alzheimer’s disease and related dementia disorders. Our proposed multi-protein panel could potentially improve future studies and clinical trials aiming at developing target-specific treatments for dementia.

**Funded by**: MRC Prion Unit at UCL

**Tau seeds precede earliest Alzheimer’s changes and are prevalent in synucleinopathies and other neurodegenerative diseases**

Matteo Manca^a*^, Heidi G. Standke^a*^, Mikayla L. Huntley^a^, Olivia R. Thomas^a^, Christina D. Orrú^b^, Andrew G. Hughson^b^, Yongya Kim^c^, Annie Hiniker^c^, David G. Coughlin^c^, Douglas Galasko^c^, and Allison Kraus^a^

^a^Department of Pathology, Case Western Reserve University School of Medicine, Cleveland, OH, United States 44,106; ^b^Rocky Mountain Laboratories, National Institute of Allergy and Infectious Diseases, National Institutes of Health, Hamilton, MT 59840; ^c^Department of Neurosciences, University of California San Diego

**Aims**: Neurofibrillary tau tangles are a hallmark of Alzheimer’s disease neuropathological change. However, it remains largely unclear how distinctive Alzheimer’s disease tau seeds (i.e. 3 R/4R) correlate with histological indicators of tau accumulation. Furthermore, AD tau co-pathology is thought to influence features and clinical progression of other neurodegenerative diseases including Lewy body disease; yet direct and reliable measurements of different types of tau seeds in such diseases is an unmet need.

**Material and Methods**: Here, we use tau real-time quaking-induced conversion (RT-QuIC) assays to selectively quantitate 3 R/4R tau seeds in the frontal lobe as a brain region that accumulates histologically identifiable tau pathology at late-stage disease.

**Results**: Seed quantitation across a spectrum of neurodegenerative disease cases and controls indicate tau seeding activity can be detected well before accompanying histopathological indication of tau deposits and even prior to the earliest evidence of Alzheimer’s related tau accumulation anywhere in the brain. In addition, Alzheimer’s tau seeds occur in all cases evaluated here inclusive of primary synucleinopathies, frontotemporal lobar degeneration and age-comparable controls albeit at multi-log lower levels than Alzheimer’s cases. α-synuclein seeding activity confirmed synucleinopathy cases and further indicated the co-occurrence of α-synuclein seeds in some Alzheimer’s disease and primary tauopathy cases. Our analysis indicates that tau seeds in the mid-frontal lobe correlate with the overall Braak tau stage, Alzheimer’s disease neuropathologic change, and frontal lobe digital quantitative immunohistochemistry measurements supporting the quantitative predictive value of tau RT-QuIC assays. Our data also indicates 3 R/4R tau seeds are elevated in female cases compared to males at higher (≥ IV) Braak stages, and that, in the cases of synucleinopathy, 3 R/4R tau seeding levels differ with Lewy body stage.

**Conclusions**: This study suggests 3 R/4R tau seeds are widespread even prior to the earliest stages of Alzheimer’s disease changes, including in normal individuals, and are prevalent across multiple neurodegenerative diseases as specific biomarkers to further define disease subtypes.

**Funded by**: This work was supported by Biomarkers Across Neurodegenerative Diseases (BAND), a partnership between the Alzheimer’s Association, The Michael J. Fox Foundation for Parkinson’s Research, The Weston Brain Institute, Alzheimer’s Research UK (to A.K., D.G.). D.C. is supported by NINDS NS120038 and D.C., D.G. by NIA AG062429, A.H. by an Alzheimer’s Association AACSF Research Grant and A.K. by BAND, NIH/NINDS and NIA (R01NS118760 and R01AG067607), Case Western Reserve University, the Britton Fund, and the Clifford V. Harding and Mina K. Chung Professorship in Pathology. This work was supported in part by the Intramural Research Program of the NIAID, NIH (C.D.O., A.G.H.).

**Grant number**: As indicated above under funding.

**Acknowledgement**: We thank the families and donors who have made this research possible; Jeff Metcalf at the UCSD Shiley-Marcos Alzheimer’s Disease Research Center for their assistance identifying and obtaining the tissue for this study; Dr. Lawrence Hansen for expert neuropathological characterization; Dr. Byron Caughey and Dr. Brent Race for the provision of tau knock-out brain tissue.

**A pipeline for atomic structure determination of infectious *ex vivo* prion fibrils by cryo-EM**

Szymon W. Manka^a^, Adam Wenborn^a^, Jemma Betts^a^, Susan Joiner^a^, Helen R. Saibil^b^, John Collinge^a^and Jonathan D.F. Wadsworth^a^

^a^MRC Prion Unit at UCL, Institute of Prion Diseases, University College London, 33 Cleveland Street, London W1W 7FF, UK; ^b^Institute of Structural and Molecular Biology, Department of Biological Sciences, Birkbeck College, University of London, Malet Street, London WC1E 7HX, UK

**Aims**: Despite fundamental advances in our understanding of prion biology, key knowledge gaps remain. These include precise delineation of prion replication mechanisms and detailed explanation of the molecular basis of prion strains and inter-species transmission barriers. Key to addressing these questions is the determination of prion structure. We have sought to address this using highly purified *ex vivo* prion preparations in combination with cryogenic electron microscopy (cryo-EM).

**Material and Methods**: Recent technical and computational advances in our laboratory have now enabled determination of the first authentic, *ex vivo* mouse prion fibril structures by cryo-EM. Key methodological advances in this area include: 1) the development of a highly efficient and simple method for obtaining high-specific infectivity preparations of *ex vivo* prion fibrils from mammalian brain; 2) optimization of the distribution of infectious prion fibrils on cryo-EM grids; and 3) application of novel methods for 3D reconstruction of amyloid fibrils from cryo-EM images within the Relion software package.

**Results**: We have established a pipeline for *ex vivo* infectious prion fibril structure determination that combines multiple recent advances in methodology from sample preparation through to computational 3D reconstruction. These advances have now enabled us to solve the near-atomic resolution structures of infectious prion fibrils from two biologically distinct mouse-adapted prion strains. These data provide the first insight into how divergent prion strains can emerge from an identical prion protein substrate. The fibrils of both mouse prion strains share the same underlying modular architecture, but with markedly altered topology.

**Conclusions**: We have just entered a very exciting era for prion research, where methods for prion fibril purification and near-atomic structure determination by single particle cryo-EM are established. Definition of *ex vivo* prion fibril structures from multiple prion strains can now be expected.

**Chronic Wasting Disease Interaction with Agricultural Crops**

Diana A. Martinez^a,b^, Elizabeth Triscott^a,b^, Emma Funk^a,b^, Alsu Kuznetsova^b,c^, Camilo DuqueVelasquez^b,c^, Debbie McKenzie^a,b^, Malinda Thilakarathna^b^, and Judd M. Aiken^b,c^

^a^Department of Biological Science, University of Alberta, Edmonton, AB T6G 2G8, Canada; ^b^Centre for Prions and Protein Folding Diseases, University of Alberta, Edmonton, AB T6G 2M8, Canada; ^c^Department of Agricultural, Food & Nutritional Science, University of Alberta, Edmonton, AB T6G 2G8, Canada

**Aims**: Chronic wasting disease (CWD) is a fatal neurodegenerative disease affecting cervids. The pathogenesis of CWD is characterized by the misfolding of the normal prion protein into an infectious isoform. CWD prions enter the environment via body secretions and excretions shed by infected cervids, contributing to transmission, persistence, and accumulation of CWD infectivity in the environment. CWD endemic regions of North America overlap prime agricultural lands. We are investigating the interactions of CWD prions with crops commonly produced in Alberta.

**Material and Methods**: Plants samples were either grown in the lab (ex. Wheatgrass) or obtained from the ALES greenhouse at the University of Alberta. Brain homogenates from infected deer were applied to the leaves and allowed to incubate for specific lengths of time. A subset of plants were treated with insecticides prior to contamination. To determine the plant-prion interactions, leaves were washed with water, and then Proteinase K (50 ug/ml) digested and analyzed by immunoblot for the presence of PrP^CWD^.

**Results**: Analysis of prion contaminated plant material demonstrated that differential binding capacity of CWD to vegetation is plant species-dependent and affected by temperature and desiccation. Treatment of plants with insecticidal chemicals, prior to experimental contamination, affects the binding efficiency of CWD to plant surfaces.

**Conclusions**: Our data suggest that vegetation serves as a reservoir for CWD; however, environmental changes (seasonality) and mitigation approaches can alter the interaction of CWD with plant material.

**Funded by**: APRI, RDAR

**Grant number**: 212,200,730,2022N067RC

**Generation and characterization of a PrP-HaloTag chimera to study the cellular trafficking and metabolism of PrP**

Antonio Masone, Lorenzo Taiarol, Marvin Oldrati, Ilaria Ortenzi, and Roberto Chiesa

Department of Neuroscience, Istituto di Ricerche Farmacologiche Mario Negri, Milan, Italy

**Aims**: The conformational conversion of the cellular prion protein (PrP^C^) into a β-sheet-rich, infectious isoform (PrP^Sc^or prion), is the key pathogenic event in prion diseases, a group of invariably fatal neurodegenerative disorders. To investigate PrP^C^metabolism in health and disease we have generated a PrP chimera containing a HaloTag derived from a *Rhodococcus rhodochrous* haloalkane dehalogenase that can covalently bind specific fluorescent ligands.

**Material and Methods**: PrP-HaloTag constructs were cloned in pCDNA3.1. HEK293 cells were transfected with Fugene HD (Promega). Transfected cell clones were isolated by limiting dilution in medium containing hygromycin 200 μg/ml (Invitrogen). PrP-HaloTag expression was analyzed by Western blot (WB) or SDS-PAGE after HaloTag labeling. The cellular localization of PrP-HaloTag was studied by confocal and super-resolution microscopy (SIM), after labeling with fluorescent cell-permeant or -impermeant substrates (Promega).

**Results**: We designed two PrP-HaloTag constructs, named PrP_N-Halo and PrP_C-Halo, by inserting the HaloTag sequence at either the N or C terminus of PrP^C^. HEK293 cells were transiently transfected with these constructs. PrP_C-Halo and PrP_N-Halo were efficiently expressed and glycosylated, but PrP_N-Halo showed an abnormal pattern of bands in WB, suggesting protein degradation, and was less efficiently expressed on the cell surface than PrP_C-Halo. Therefore, we used PrP_C-Halo (hereafter PrP-Halo) to generate stable HEK293 cell lines, and studied the protein localization using cell-permeant and -impermeant fluorescent ligands. Cell surface-labeled PrP-Halo was N-terminally cleaved between amino acid 107 and 114, consistent with physiological PrP^C^α-cleavage at site 110/111 during endocytic recycling. Supporting this, a PrP-Halo construct deleted between amino acid 105 and 125 (PrP-Halo ΔCR) was expressed on the cell surface but not α-cleaved. When cells were stimulated with a PrP^C^-degrading compound, cell surface PrP-Halo was internalized by clathrin-coated pits and degraded through the lysosomal pathway, as shown by biochemical analysis and super-resolution (SIM) microscopy.

**Conclusions**: Our analysis indicates that PrP-Halo is correctly expressed on the plasma membrane of HEK293 cells, it is glycosylated and subjected to physiological α-cleavage. The possibility of specifically labeling different subsets of PrP^C^molecules with cell permeant and impermeant fluorescent ligands, and to analyze the protein by imaging and biochemical approaches, will allow to precisely define the cellular trafficking and metabolism of PrP in normal and pathological conditions.

**Funded by**: Telethon Italy, Italian Ministry of Health and the CJD Foundation USA.

**Grant number**: Telethon Italy award GGP15225, Italian Ministry of Health award RF-2016-02362950.

**Acknowledgement**: Stefano Fumagalli for advice on super-resolution microscopy.

**Behavioral deficits, learning impairment, and enhanced hippocampal excitability in co-isogenic*Prnp^ZH3/ZH3^* mice**

A. Matamoros-Angles^a,b§^, A. Hervera ^b^^§^, J. Soriano^c^, E. Martí^d^, P. Carulla^b^, F. Llorens^e^, M. Nuvolone^f,g^, A. Aguzzi^f^, I. Ferrer^h^, A. Gruart^i^, M. Glatzel^a^, JM. Delgado-García^i*^, and JA. Del Río^b*^

^§^Equal contribution * Co-corresponding authors

^a^Institute of Neuropathology, University Medical Center Hamburg-Eppendorf, Hamburg, Germany; ^b^Molecular and Cellular Neurobiotechnology, Institute of Bioengineering of Catalonia, Barcelona, Spain; Department of Cell Biology, Physiology, and Immunology, University of Barcelona, Barcelona, Spain; CIBERNED, Institute of Health Carlos III, Barcelona, Spain; Institute of Neuroscience, University of Barcelona, Barcelona, Spain; ^c^Departament de Física de la Materia Condensada, University of Barcelona, Spain; Institute of Complex Systems, University of Barcelona, Barcelona, Spain; ^d^Department of Biomedicine, University of Barcelona, Barcelona, Spain; Bioinformatics and Genomics, Center for Genomic Regulation, Barcelona, Spain; ^e^Department of Neurology, University Medical School, Göttingen, Germany, Bellvitge Biomedical Research Institute (IDIBELL), L’Hospitalet de Llobregat, Catalonia, Spain; ^f^Institute of Neuropathology, University Hospital of Zürich, Zürich, Switzerland; ^g^Amyloidosis Center, Foundation IRCCS Policlinico San Matteo, Department of Molecular Medicine, University of Pavia, Pavia, Italy; ^h^Bellvitge University Hospital, IDIBELL, L’Hospitalet de Llobregat, Spain, Department of Pathology and Experimental Therapeutics, University of Barcelona, Barcelona, Spain; ^I^Division of Neurosciences, Pablo de Olavide University, Seville, Spain

**Aims**: Decades of studies pointed out the participation of PrP^C^in several physiological roles, but not without controversy. While previous studies have established a neuroprotective role in stroke and epilepsy, conflicting evidence for a synaptic function has revealed both reduced and enhanced long-term potentiation and variable observations on memory, learning, and behavior in mouse knock-out models. Such evidence has been confounded by the absence of an appropriate knock-out mouse model to dissect the biological relevance of PrP^C^, with some functions recently shown to be misattributed to PrP^C^due to the presence of genetic artifacts in mouse models. Here we elucidate the role of PrP^C^in the hippocampal circuitry and its related functions, like learning and memory, using the new strictly co-isogenic *Prnp^0/0^* mouse (*Prnp^ZH3/ZH3^*).

**Material and Methods**: Behavioral and operant conditioning tests were performed to evaluate the memory and learning capabilities of the *Prnp^ZH3/ZH3^* model. Moreover, kainate was administrated to test the epileptical phenotype in this knock-out model. Finally, in vivo electrophysiological recordings were carried out at CA3-CA1 synapses in living behaving mice, and spontaneous neuronal firing and network formation were monitored in primary neuronal cultures of *Prnp^ZH3/ZH3^* vs. wild-type mice.

**Results**: Results showed decreased motility, impaired operant conditioning learning, and anxiety-related behavior in *Prnp^ZH3/ZH3^* animals. PrP^C^absence enhanced susceptibility to high-intensity stimulations and kainate-induced seizures. However, long-term potentiation (LTP) was not enhanced in the *Prnp^ZH3/ZH3^* hippocampus. In addition, we observed a delay in neuronal maturation and network formation in *Prnp^ZH3/ZH3^* cultures.

**Conclusions**: Our results demonstrate that PrP^C^promotes neuronal network formation and connectivity. PrP^C^mediates synaptic function and protects the synapse from excitotoxic insults. Its deletion may underlie an epileptogenic-susceptible brain that fails to perform highly cognitive-demanding tasks such as associative learning and anxiety-like behaviors.

**Funded by**: This research was supported by *PRPSEM* Project with reference RTI2018-099773-B-I00 from MCINN/AEI/10.13039/501,100,011,033/ FEDER ‘Una manera de hacer Europa’, the CERCA Programme, and the Commission for Universities and Research of the Department of Innovation, Universities, and Enterprise of the Generalitat de Catalunya (SGR2017-648), CIBERNED (CMED2018-2) to JADR and IF. The project leading to these results received funding from the ‘la Caixa’ Foundation (ID 100010434) under the agreement LCF/PR/HR19/52,160,007 and the María de Maeztu Unit of Excellence (Institute of Neurosciences, University of Barcelona) MDM-2017-0729 to JADR. JS was supported by FIS2016-78,507-C2-2-P from (MCIU/FEDER/AEI), SGR2017-1061 from the Generalitat de Catalunya, and the European Union’s Horizon 2020 research and innovation program under the grant agreement No. 713,140 (MESOBRAIN). Support was also received from MINECO (BFU2017-82,375-R) and Junta de Andalucía (BIO-122, UPO-1250734, and P18-FR-823) grants to AG and JMDG. F.LL. was supported by Instituto Carlos III (grant PI19‐00144). A.M-A. was supported by the Tatiana Pérez de Guzmán el Bueno Foundation.

**Acknowledgment**: The authors thank Tom Yohannan for editorial advice and Miriam Segura-Feliu, María Sánchez-Enciso, and José M. González-Martín for their technical help.

**Extracellular vesicles in the pathophysiology of Alzheimer’s disease: understanding the role of the prion protein**

A. Matamoros-Angles^a^^§^, E. Karadjuzovic^a^, I. Egger^a^, S. Da Vela^b^, L. Amin^c^, A. Zafar^a^, B. Mohammadi^a^, C. Seuring^d^, B. Siebels^e^, H. Voß^e^, H. Schlüter^e^, M. Schweizer^f^, B. Puig^g^, I. Ferrer^h^, F. Zunke^i^, H. C. Altmeppen^a^, D. A. Harris^c^, D. Svergun^b^, M. Glatzel^a^, and M. Shafiq^a§^

^§^Equal contribution ^a^Institute of Neuropathology, University Medical Center Hamburg-Eppendorf, Hamburg, Germany; ^b^European Molecular Biology Laboratory Hamburg, Germany; ^c^Department of Biochemistry, Boston University School of Medicine, Boston, MA, USA; ^d^Multi-User CryoEM facility, Centre for Structural Systems Biology, Hamburg, Germany; ^e^Institute of Clinical Chemistry and Laboratory Medicine, University Medical Center Hamburg-Eppendorf, Hamburg, Germany; ^f^Core Facility of Electron Microscopy, Center for Molecular Neurobiology, University Medical Center Hamburg-Eppendorf, Hamburg, Germany; ^g^Department of Neurology, Experimental Research in Stroke and Inflammation, University Medical Center Hamburg-Eppendorf, Hamburg, Germany; ^h^Bellvitge University Hospital, IDIBELL, L’Hospitalet de Llobregat, Spain; ^i^Department of Molecular Neurology, University Hospital Erlangen, Friedrich-Alexander University Erlangen-Nürnberg, Erlangen, Germany

**Aims**: Alzheimer’s disease (AD) is the leading cause of dementia worldwide. The trigger(s) is (are) still debated, and available symptomatic treatments show modest efficacy at best. Extracellular vesicles (EVs) have been related to crucial brain functions, such as myelin maintenance and neurotransmission. Cellular prion protein (PrP^C^) is abundantly expressed on the surface of the EVs. In AD, EV-associated PrP^C^, along with proteolytically released extracellular PrP^C^fragments, sequesters Aβ oligomers (Aβo) and thus accelerates Aβ fibrillation thereby reduces the presence of neurotoxic Aβ oligomers load, yet formal evidence is still lacking. Here, we aim to study the role of PrP^C^expressed on Neuro 2a- and human brain-derived EVs on Aβ fibrillization and AD pathophysiology.

**Material and Methods**: PrP^C^-expressing (WT) and -deficient (KO) EVs were obtained from WT and PrP^C^-KO Neuro-2a (N2a) cells, respectively. Moreover, EVs were isolated from frontal cortex of AD patients and age-matched controls by employing a novel protocol without the need of adding proteases (which affects processing of PrP and other EV surface proteins). EVs were characterized using Nanoparticle tracking analysis (NTA), immunoblotting, and electron microscopy (cryo-EM and negative stain TEM). To further the study objectives, small angle X-ray scattering (SAXS), super-resolution microscopy (SRM), Cryo-EM, proteomic and lipidomic profiling, and associative biochemical and biophysical methods were employed. Lastly, additional validations and proof-of-concept experiments will be performed in human iPSC models.

**Results**: SAXS studies along with SRM and aggregation assays helped us to identify potent Aβo-sequestering properties of the N2a-derived WT-EVs, compared to KO-EVs. Lipidomic and proteomic profiling of N2a-derived WT- and KO-EVs pointed towards marked compositional differences (i.e., higher abundance of certain kinases, RNA- and DNA-binding proteins in KO-EVs).

Our novel protease-free EV isolation method for brain tissues resulted in EV subpopulations with conserved ‘native’ surface protein decoration, and highlighted alterations in PrP^C^expression in EV subpopulations specific to AD. ‘Omics’ studies from human brain-derived EVs are underway and will help us to better understand the described neuroprotective role of EV-PrP on AD.

**Conclusions**: Our findings provide new evidence for crucial roles carried out by PrP^C^-expressing EVs in the pathophysiology of AD, i.e., their involvement in Aβ aggregation, a related potential rescue mechanism against Aβo toxicity, and AD-specific EV-mediated intercellular communication. Non-artifactual EVs (with native protein decoration) derived from human brain will be useful to achieve better mechanistic insight into EVs’ pathophysiological roles in AD.

**Funded by**: i) The Joachim Herz foundation, University of Hamburg (PIER Hamburg/Boston seed grant); ii) the PIER seed project, and iii) the European Union’s Horizon 2020 research and the Marie Sklodowska-Curie innovation program.

**Grant number**: i) PHM-2019-03; ii) Project PIF 2020–10); and iii) EXOSOMES_AD (Grant agreement: N°101030402).

**Specific electroencephalogram features in the very early phases of sporadic Creutzfeldt–Jakob disease**

Taiki Matsubayashi^a^, Miho Akaza^b^, Yuichi Hayashi^c^, Tsuyoshi Hamaguchi^d^, Katsuya Satoh^e^, Koki Kosami^f^, Ryusuke Ae^f^, Tetsuyuki Kitamoto^g^, Masahito Yamada^h,i^, Takayoshi Shimohata^c^, Takanori Yokota^a^, and Nobuo Sanjo^a,i^

^a^Department of Neurology and Neurological Science, Tokyo Medical and Dental University Graduate School of Medical and Dental Sciences, Bunkyo-ku, Japan; ^b^Respiratory and Nervous System Science, Biomedical Laboratory Science, Graduate School of Medical and Dental Sciences, Tokyo Medical and Dental University, Bunkyo-ku, Japan; ^c^Department of Neurology, Gifu University Graduate School of Medicine, Yanagido, Japan; ^d^Department of Neurology, Kanazawa Medical University, Uchinada-machi, Japan; ^e^Department of Locomotive Rehabilitation Science, Nagasaki University Graduate School of Biomedical Sciences, Sakamoto, Japan; ^f^Department of Public Health, Jichi Medical University, Shimotsuke-shi, Japan; ^g^Department of Neurological Science, Tohoku University Graduate School of Medicine, Aoba-ku, Japan; ^h^Department of Neurology and Neurobiology of Aging, Kanazawa University Graduate School of Medical Science, Takara-machi, Japan; ^i^Department of Neurology, Kudanzaka Hospital, Chiyoda-ku, Japan

**Aims**: We aimed to analyze the changes in electroencephalography (EEG) findings in patients with early-stage sporadic Creutzfeldt–Jakob disease (sCJD) and clarify their association with clinical symptoms, because very early EEG features prior to the emergence of periodic sharp-wave complexes (PSWCs) in sCJD are sometimes difficult to distinguish from those in non-convulsive status epilepticus (NCSE).

**Material and Methods**: Fourteen patients with sCJD (eight with MM1/classic and six with MM2c) were included in this study. The predominant findings of the first EEG were categorized as 1) lateralized periodic discharges (LPDs), 2) central sagittal sporadic epileptiform discharges (CSSEDs) showing midline predominant generalized spike-and-wave complexes and/or sharp waves in the central sagittal regions, or 3) focal epileptiform discharges. Clinical records, magnetic resonance imaging (MRI), and changes in EEG were compared between three groups (LPD in MM1/classic, CSSED in MM1/classic, and focal epileptiform discharge in MM2c).

**Results**: Three (37.5%) and five (62.5%) patients with MM1/classic sCJD were classified into the LPD and CSSED groups, respectively. Patients in the LPD group were accompanied by cortical hyperintensities at the corresponding areas on MRI, while those in the CSSED group showed hyperintensities on MRI at unassociated cortical areas. The average duration from onset to the first EEG was not significantly different between the LPD and CSSED groups (1.67 months vs. 1.6 months, respectively). Follow-up EEG of three (100%) patients in the LPD group after 2.67 months from onset and four (80%) in the CSSED group after 3.25 months showed transitions to PSWCs. All patients with MM1/classic sCJD showed myoclonus on initial EEG, and the average period between onset and appearance of myoclonus was 1.67 months in the LPD group and 1.6 months in the CSSED group. The symptomatic side was opposite to the hemisphere showing LPDs or higher-amplitude central sagittal epileptiform activity.

**Conclusions**: All patients with MM1/classic sCJD had epileptiform discharge on EEG prior to the emergence of PSWCs, indicating that physicians must distinguish patients with sCJD from those with epilepsy because a rapidly progressive cognitive impairment mimicking CJD may be observed in patients with NCSE. LPDs and CSSEDs on EEG were identified at the area of brain which reflect severe pathological changes including PrPSc deposition and spongiform changes in sCJD. These pathological changes were likely associated with cortical myoclonus before the emergence of PSWCs on EEG. EEG features occurring before PSWCs could contribute greatly to the early diagnosis of MM1/classic sCJD.

**Funded by**: a Grant-in-Aid from the Research Committee of Prion Disease and Slow Virus Infection of the Ministry of Health, Labour, and Welfare of Japan (TH, KS, RA, YN, TK, MY, and NS), a Grant-in-Aid from the Research Committee of Molecular Pathogenesis and Therapies for Prion Disease and Slow Virus Infection of the Ministry of Health, Labour, and Welfare of Japan (TH, TK and MY), and a Grant-in-Aid from the Research Committee of Surveillance and Infection Control of Prion Disease of the Ministry of Health, Labour, and Welfare of Japan (MY, KS, RA, YN, TK, MY, and NS).

**Grant number**: 20FC2001, and 20FC1054

**Acknowledgement**: The authors are grateful to the members of the Department of Neurology and Neurological Science, Tokyo Medical and Dental University Hospital. We would like to sincerely thank the patients and their families for their participation in this study. We would also like to thank the members of the Department of Neurology, Gifu University Graduate School of Medicine, and the Department of Neurology and Neurobiology of Aging, Kanazawa University Graduate School of Medical Science for their collaboration with the study.

**What happened to the pierrot? – painting alterations of a patient with Alzheimer’s disease and Lewy body dementia**

Kacper Mazurkiewicz^a^, Marcin Wojtera^b^, Adrianna Madej^a^, Małgorzata Rózga^c^, Paweł P. Liberski^d^, and Beata Sikorska^d^

^a^Medical University of Lodz, Lodz, Poland; ^b^Department of Old Age Psychiatry and Psychotic Disorders; Central Clinical Hospital of Lodz; Lodz, Poland; ^c^Lodz, Poland; ^d^Department of Molecular Pathology and Neuropathology, Medical University of Lodz, Lodz, Poland

**Aims**: Alzheimer’s disease (AD) and dementia with Lewy bodies (DLB) constitutes leading causes of dementia worldwide. Visuospatial cognition, perceptual functioning, as well as other important for creative work traits have been reported to be impaired in both types of dementias in a disease-specific way. Patients with DLB have all visual perceptual functions affected and in AD predominant change is spatial. However, best to our knowledge, there were no prior reports of such changes in cases with mixed pathology. The study aims to identify the influence of Lewy body dementia with AD co-pathology on patient’s visual art production. To this end we examined artistic works of a patient with autopsy confirmed limbic type of Lewy body pathology and AD created at different stages of the disease.

**Material and Methods**: We examined a collection of artistic works of a patient with neuropathologically confirmed Alzheimer disease and Lewy body dementia. The patient was a female professional artist with a higher artistic education Following qualities of works: aesthetics, composition, evocative impact, novelty, representation-technique, technique, bizarreness, facial features were assessed at three different stages of the disease. Assessment was performed by three evaluators, of whom one was with a higher artistic education. Additionally, the brain slides were re-examined to check for pathology in brain regions responsible for functions essential for creative work. Immunohistochemistry was performed in regions of interest with antibodies against phTau, ph-alpha-synuclein, amyloid-beta, TDP-43, GFAP, p62.

**Results**: We observed changes in patient’s artwork in all of evaluated qualities except for composition, bizarreness and novelty during the progress of the disease. Alzheimer type pathology was consistent with Braak stage 6 and alpha-synuclein pathology matched limbic type of DLB according to McKeith. Apart from the hippocampus and the amygdala abundant tau deposits were present in the occipital cortex. Alpha-synuclein pathology was most severe in the amygdala and the hippocampus.

**Conclusions**: We observed influence of changes in motor ability and visuospatial perception on visual art production throughout the course of the disease. Moreover, we found that brain regions essential for art perception and creative work were affected by the pathological process. Damaged occipitoparietal pathway and hippocampal formation resulting in flawed spatial episodic memory may be associated with observed changes in the artwork.

**Funded by**: Statutory funds of Medical University of Lodz

**Are rapid tests and confirmatory western blot for cattle and small ruminants reliable tools for the diagnosis of Chronic Wasting Disease in Europe?**

Maria Mazza^a^*, Linh Tran^b^*, Daniela Loprevite^a^, Maria C. Cavarretta^a^, Luana Dell’Atti^a^, Jørn Våge^b^, Knut Madslien^b^, Turid Vikøren^b^, Tram T. Vuong^b^, Elena Bozzetta^a#^, and Sylvie L. Benestad^b#^

^* #^These Authors equally contributed to this study

^a^Italian Reference Laboratory for TSEs – Istituto Zooprofilattico Sperimentale del Piemonte, Liguria e Valle d’Aosta, Turin, Italy; ^b^OIE Reference Laboratory for Chronic Wasting Disease ^–^Norwegian Veterinary Institute, Oslo, Norway

**Aims**: The aim of this study was to evaluate the analytical sensitivity of the three commercially available and approved rapid tests for TSE diagnosis in cattle and small ruminants for detecting CWD strains circulating in Europe, and of two different confirmatory western blot methods.

**Material and Methods**: Five moose and two reindeer, detected as positive through the Norwegian surveillance programme, were analysed in this study, as well as negative pools of both moose and reindeer brain tissue. To evaluate the analytical sensitivity of these methods, dilution series of each homogenate were prepared in negative brain material. The samples were analysed in parallel at the Italian Reference Laboratory and Norwegian Veterinary Institute by the following test: TeSeE^TM^SAP Combi (Bio-Rad), TeSeE^TM^Sheep/Goats (Bio-Rad), HerdCheck BSE-Scrapie Ag test (IDEXX) and the two confirmatory western blot methods, one commercially available (TeSeE^TM^Western Blot) and one (SAF-Immunoblot) developed at the Italian NRL for TSEs. For SAF-Immunoblot method five monoclonal anti-PrP antibodies were chosen among the most commonly used for the diagnosis of animal TSEs and raised against different regions of the prion protein.

**Results**: The analyses revealed that all rapid tests are able to identify the different strains of CWD circulating in Europe, although with different analytical sensitivity. Both confirmatory western blot methods showed a good sensitivity and were able to confirm the positive results obtained from rapid tests.

**Conclusions**: Although this study presents a limitation due to the small number of samples analysed, it is conceivable that the rapid and confirmatory diagnostic systems applied in Northern Europe for the CWD surveillance in the cervid populations are reliable tools.

**Performance of second generation CSF RT-QuIC in a clinical CJD Surveillance setting**

Neil McKenzie, Kimberley Burns, Mary Andrews, Hatice Kurudzhu, Johnny Tam, John Centola, Jan Mackenzie, Suvankar Pal, and Alison JE Green

The National CJD Research & Surveillance Unit, Centre for Clinical Brain Sciences, University of Edinburgh, Edinburgh EH16 4SB

**Aims**: The high sensitivity and specificity of CSF RT-QuIC analysis has been a major advance in the diagnosis of sporadic CJD (sCJD) and has led to its inclusion in the revised European CJD Surveillance network sCJD diagnostic criteria. Full-length hamster rPrP is the most widely used RT-QuIC substrate but has an assay time of 80–90 hours (first generation RT-QuIC). The use of truncated hamster rPrP as an alternative substrate (second generation RT-QuIC) has been reported to result in a more sensitive and rapid CSF RT-QuIC assay. Our aim was to compare the performance of the first and second generation RT-QuIC assays in a clinical CJD surveillance setting.

**Material and Methods**: First and second generation RT-QuIC assays were performed as previously described ^a,b^. An initial retrospective study to assess the optimal cut-off time for the second generation CSF RT-QuIC assay was undertaken with 43 neuropathological confirmed or probable sporadic CJD patients, (21 M:22 F; mean age (SD) 65 (9.5) years) and 56 control patients, 31 M:25 F, mean age (SD) 69.5 (11.6) years). A subsequent prospective study was undertaken with 62 neuropathological confirmed or probable sporadic CJD patients, (28 M:34 F; mean age (SD) 66.5 (11.0) years) and 47 control patients (29M:18 F, mean age (SD) 66.3 (13.1) years).

The sensitivity, specificity and accuracy of the first and second generation assays was assessed as well as the overall reaction time.

**Results**: The initial retrospective study showed that the optimal combination of sensitivity and specificity for the second generation CSF RT-QuIC assay was obtained using a cut-off of 40 hours. The sensitivity, specificity and accuracy of the first generation CSF RT-QuIC assay was 94%, 100% and 96% respectively, compared to a sensitivity, specificity and accuracy of 90%, 98% and 94% respectively, for the second generation CSF RT-QuIC assay.

Of the six sCJD patients that were negative for the second generation RT-QuIC assay, four patients had a positive first generation CSF RT-QuIC result. One false positive CSF RT-QuIC result was obtained using the second generation CSF RT-QuIC assay. This patient had a posterior reversible encephalopathy syndrome.

**Conclusions**: This study shows that the second generation CSF RT-QuIC assay is less sensitive than the first generation RT-QuIC assay, however the number of sCJD patients investigated is small. The major advantage of the second generation CSF RT-QuIC assay is the significant reduction in assay time.

1. Orrú CD et al, *Ann Clin Transl Neurol* 2020; **7(11)**:2262–2271
2. McGuire LI et al, *Ann Neurol* 2016;**80**:160–5

**Funded by**: The NCJDRSU, UK is funded by the Department of Health and Social Care Policy Research Programme and the Scottish Government

**Grant number**: PR-ST-0614-00008

**Acknowledgement**: Surveillance of CJD is dependent on the co-operation of neurologists and neuropathologists throughout the UK. The authors are particularly grateful to the relatives of the patients for their co-operation and support.

**Multigenerational Chronic Wasting Disease Mother to Offspring Transmission in Reeves’ muntjac deer**

McNulty EE, Nalls AV, and Mathiason CK

Colorado State University, Fort Collins, CO USA

**Aims**: Chronic wasting disease (CWD) continues to demonstrate geographic expansion, now found in captive and/or free-range cervid populations in North America, Asia, and Scandinavia. CWD mother to offspring transmission has been demonstrated in the experimental Reeves’ muntjac system, as well as in populations of free-ranging elk and white-tailed deer. Our studies further explore the potential for multigenerational transmission as a mechanism to help explain the facile transmission of this fatal prion disease of cervids.

CWD-infected muntjac dams are able to become pregnant, carry, deliver, and rear offspring during the long asymptomatic phase of prion infection. Our studies have shown that CWD prions can be transmitted from mother to first-generation offspring leading to prion infection and subsequent development of TSE disease, and that transmission occurs during gestation (Nalls 2013). We have also demonstrated that there are infection prions within the pregnancy microenvironment by bioassay (uterus, birthing fluids, and placentomes) (Nalls 2017).

The CWD muntjac system has permitted us to further explore the impact of maternal infections by assessing first and second generation offspring born to infected dams for the potential of multigenerational CWD transmission. Here we describe the presence of CWD infectivity within additional tissues of the pregnancy microenvironment, and within tissues of second generation non-viable offspring that harbor protein misfolding cyclic amplification (PMCA) conversion competent amyloid.

**Material and Methods**: We assessed tissues harvested from clinical terminal muntjac dams, in-uterine collections of late term fetuses, and full-term second-generation nonviable muntjac offspring for CWD infectivity by mouse bioassay. Transgenic mice expressing the cervid prion protein, Tg(CerPrP-E226)5037± (n = 6/cohort or n = 9/cohort), were either IC inoculated with 10% ovary or mammary gland/lymph node (LN) homogenate, or IP-inoculated with PMCA-amplified RAMALT, ileum, or obex from first generation fetuses. Additional cohorts of mice were IP-inoculated with lung, mammary gland, kidney or uterus harvested from nonviable 2^nd^generation muntjac offspring (n = 2). Mice from all cohorts were examined for prions by western blot and real time quaking-induced conversion (RT-QuIC).

**Results**: All mice inoculated with muntjac dam tissue (n = 18), all mice inoculated with first generation PMCA-amplified tissue (n = 16) and all mice (n = 20) inoculated with PMCA-amplified tissue from ‘ gestational CWD-exposed’ second-generation offspring developed signs consistent with TSE disease, including severe ataxia and weight loss between 209–373 days pi, and were confirmed CWD positive by western blot and RT-QuIC. Negative control mice (n = 25) receiving negative dam homogenates and PMCA-amplified negative age and tissue-matched homogenates remained healthy and TSE-free for the same duration.

**Conclusions**: Our data indicates that: (1) multigenerational transmission of infectious CWD prions from mother-to-offspring may be possible and (2) early and persistent exposure of the developing embryo to infectious CWD prions in the uterine microenvironment may help explain the facile transmission of CWD in the native host.

**Funded by**: NIH

**Grant number**: 2R01AI112956-06, RO1AI093634-01A1

**Acknowledgement**: We acknowledge LAR for great animal care

**Genome wide association study of survival and age of onset in sporadic Creutzfeldt-Jakob disease**”

Simon Mead, Holger Hummerich for the International CJD GWAS collaboration

MRC Prion Unit at UCL

**Aims**: Sporadic Creutzfeldt-Jakob disease (sCJD) is highly variable in clinical duration and age at onset. We sought to discover genetic factors that modify these phenotypes in a large international collaborative study.

**Material and Methods**: We assembled 4929 cases of probable or definite sCJD by contemporary diagnostic criteria either included in Jones et al. Lancet Neurology 2020, or newly genotyped on Illumina’s Global Screening Array. Genotype doses were imputed using the Michigan Imputation Server, resulting in 6,308,901 SNPs passing quality control. Clinical duration (median:4, IQR:2.5–9 (months)) was available in 3773 and age at onset (median:67, IQR:61–73 (years)) in 3767 cases. Phenotypes were modelled as normally distributed quantitative traits following transformation using methods developed by Box and Cox. Additive and Heterozygous genetic models were run in SNPtest with sex, contributing site and genetic ancestry covariates. Secondary analyses included conditional models, gene-based tests, gene-set analyses and development of polygenetic risk scores.

**Results**: Phenotypes were successfully transformed to normal distributions allowing genome-wide analysis without statistical inflation (lambda = 1.000/1.000 for clinical duration/age). 54 SNPs achieved genome wide significance for the clinical duration phenotype all at the *PRNP* locus (top SNP rs1799990, P = 6.25x10^−38^, beta = 1.417 for additive model; rs1799990, P = 3.94x10^−67^, beta = 2.328 for heterozygous model). *PRNP* was the nearest gene and obvious outstanding genome-wide determinant of clinical duration. There was no effect at eQTL SNPs that associate with brain expression of *PRNP* and no genome-wide significant SNPs after conditioning for codon 129. There were 50 suggestive associated SNPs (P < 1 x 10^–5^, including at regions near to *HDHD5* (chromosome 22), *FHIT* (chromosome 3) and *EREG* (chromosome 4). The top Gene-set-based analysis identified binders of type-5 metabotropic glutamate receptors (GO Molecular Function ontology n = 1738, P = 2x10^−5^). Age-based analysis did not identify any genome-wide associations. We will discuss the utility of polygenetic risk scores in prediction of total survival in CJD.

**Conclusions**: Relative to other loci, *PRNP* is outstanding in modification of the rapidity of progression of CJD, but not the age at clinical onset. This work suggests additional non-PrP mechanisms modestly affect survival including support for the already proposed role of metabotropic glutamate receptor type 5.

**Funded by**: MRC (UK)

**Two-pronged pharmalogical interventions for prion disease targeting propagation and toxicity**

Robert C.C. Mercer^a^, Nhat T.T. Le^a^, Deepthi Yedlapudi^a^, Jenna Crowell^b^, Doug Fraser^c^, Janelle Vultaggio^a^, John Alam^d^, Aaron Beeler^c^, Glenn C. Telling^b^, and David A. Harris^a^

^a^Department of Biochemistry, Boston University School of Medicine, Boston, USA; ^b^Prion Research Center, Department of Microbiology, Immunology and Pathology, Colorado State University, Fort Collins, USA; ^c^Department of Chemistry, Boston University, Boston, USA; ^d^EIP Pharma Inc., Cambridge, USA

**Aims**: The discovery and characterization of two anti-prion compounds that target distinct steps in prion pathogenesis: propagation and toxicity

**Material and Methods**:

- The Psychoactive Drug Screening program (PDSP), a small molecule competition binding assay
- RNA-sequencing
- ScN2a PrP^Sc^clearance assays
- Hippocampal spine retraction assays
- Immunocytochemistry
- CRISPR/Cas9 mediated gene knockout
- Large scale chemical synthesis
- *in vivo* drug treatment of RML infected mice
- Biochemical assays of brain homogenate
- Histopathological analysis

**Results**: Using a high throughput screen for molecules that prevent mutant (ΔCR) PrP toxicity, we discovered a novel class of anti-prion compound; phenethyl piperidines. Further study of this compound class has led, unexpectedly, to the discovery of a number of additional novel anti-prion compounds, some of which have previously identified molecular targets and clinical uses. In addition to inhibiting prion propagation when applied to ScN2a cells, these molecules can prevent the retraction of cultured hippocampal neuron dendritic spines following exposure to purified prion preparations. We have identified central nervous system receptors/channels that bind to these molecules using the Psychoactive Drug Screening Program (PDSP) and explored their role in the observed anti-prion effects through CRISPR/Cas9 mediated gene disruption. In a seperate line of investigation, we have identified a neurotoxic pathway downstream of prion propagation that can be disrupted using inhibitors of p38 MAPK despite prion propagation continuing unabated. We have tested compounds from each group, both alone and in combination, for *in vivo* efficacy against RML prions in C57BL/6 J mice by administration through a liquid diet.

**Conclusions**: Molecules that inhibit PrP^Sc^formation also protect dendritic spines from collapse, suggesting that newly converted PrP^Sc^at the cell surface triggers prion synaptotoxic effects. This inference is supported by the absence of spine retraction following prion exposure of hippocampal neuronal cultures derived from *Prnp^0/0^* mice or from transgenic mice expressing PrP lacking the positively charged N-terminal region, which is known to play a role in PrP^C^-PrP^Sc^ conversion and as well as in certain toxic activities of PrP. Despite well-defined efficacy *in vitro* and brain penetrance *in vivo*, the administration of either molecule alone or in combination had no effect upon incubation time or deposition pattern of PrP^Sc^. Nevertheless, these and related molecules may be useful tool compounds to understand basic mechanisms of prion propagation and toxicity.

**Funded by**: National Institutes of Health (USA), Department of Defense (USA)

**Grant number**: R01 NS065244-11A1, W81XWH-21-1-0141

**Cryo-EM reveals small-molecule binding to the paired helical fillament conformation of tau prions from Alzheimer’s disease**

Gregory E. Merz^a,b^, Matthew J. Chalkley^c^, Nick A. Paras^a,b^, Eric Tse^a^, Joanne Lee^a^, Stanley B. Prusiner^a,b,d^, William F. DeGrado^a,c^, and Daniel R. Southworth^a,d^

^a^Institute for Neurodegenerative Diseases, Weill Institute for Neurosciences, University of California, San Francisco, CA, USA; ^b^Department of Neurology, University of California, San Francisco, CA, USA; ^c^Department of Pharmaceutical Chemistry, Cardiovascular Research Institute, University of California, San Francisco, CA, USA; ^d^Department of Biochemistry and Biophysics, University of California, San Francisco, CA, USA

**Aims**: Recent remarkable efforts in structural biology have led to the determination of numerous high-resolution structures of tau prion filaments purified from the tissues of patients with different tauopathies. At the same time, newly developed small molecules offer promise as positron emission tomography (PET) ligands for diagnosing specific tauopathies. However, a high-resolution structure elucidating the location and mechanism of binding of these small molecules to tau prion filaments has remained elusive.

**Material and Methods**: Tau filaments from an 88 year old male patient with a neuropathologically confirmed Alzheimer’s disease (AD) diagnosis were purified from the frontal cortex using established protocols. These AD filaments were incubated with PET ligand and then plunge frozen on holey carbon grids and imaged on a Titan Krios cryo-electron microscope. Images were analyzed using the helical reconstruction features in Relion 3.1.

**Results**: Here, we present a high-resolution (2.7 Å) structure of a known PET probe in complex with paired helical filaments (PHFs) purified from the brain tissue of a patient with Alzheimer’s disease. Incredibly, the ligand shows a clear stacking arrangement along the filament axis, with a ~ 40° tilt relative to the beta-sheet arrangement. This tilt results in each molecule making extensive contacts, spanning 3 beta sheets of the filament. To further understand the molecular interactions of the stacked arrangement, we used density functional theory (DFT) and Hartree-Fock London Dispersion (HFLD) calculations to generate and evaluate several conformer stacks of ligand molecules and assessed their binding to the protein backbone. These methods reveal the origin of the tilt relative to the backbone and allow us to decompose the binding and stacking energies. Furthermore, while binding of a single ligand is energetically unfavorable, binding of subsequent adjacent ligands (i.e., in a stacked arrangement) is energetically favorable overall.

**Conclusions**: This stacked arrangement of ligands represents a new motif of small-molecule-protein binding. Understanding the energetics of this binding mode holds promise for rapidly advancing the computational development of disease-specific small molecule PET probes and therapeutics for diagnosing and treating neurodegenerative diseases.

**Funded by**: NIH

**Grant number**: AG002132

**Appearance of new scrapie prion strain by the conformational rearrangement of parental scrapie prion strain through serial transmission in wild-type mice**

Kohtaro Miyazawa^a^, Yuichi Matsuura^b^, Morikazu Imamura^c^, and Yoshifumi Iwamaru^b^

^a^Division of Zoonosis Research, National Institute of Animal Health (NIAH), National Agriculture and Food Research Organization (NARO), Tsukuba, Ibaraki, Japan; ^b^Division of Infectious Animal Disease Research, NIAH, NARO, Tsukuba, Ibaraki, Japan; ^c^Division of Microbiology, Department of Infectious Diseases, Faculty of Medicine, University of Miyazaki, Miyazaki, Miyazaki, Japan

**Aims**: Scrapie is a prion disease in sheep and goat. Many biologically different scrapie prion strains have been reported based on the results of wild-type mouse bioassays. However, it remains unclear whether some prion strains are produced by real mutation processes in mice, or if all of them had preexisted in the original scrapie-affected sheep. Thus, we investigate whether new prion strain is generated by conformational mutation of the parental prion strain during serial transmission in mice or not.

**Material and Methods**: Two biologically different prion strains (designated as FuL and FuS, respectively) were isolated from a single natural sheep classical scrapie isolate by CD-1 mouse bioassays. Cloned FuL prion, which meant at least FuS prion-free, was obtained by limiting dilution method through GT1-7 cell culture systems. Then, we demonstrated that FuS prion newly appeared during serial transmission of the cloned FuL prion in CD-1 mice.

**Results**: FuS prion-affected mice showed short incubation time (~150 days), emaciation, marked vacuolation and widespread PrP^Sc^distribution in hippocampus. In contrast, FuL prion-affected mice were characterized by long incubation time (~300 days), polyuria and relatively little vacuolation and limited PrP^Sc^distribution in hippocampus. When GT1-7 cells were exposed to 10^−5^brain homogenate (BH) prepared from a mouse that developed the FuL disease phenotype (termed as GT/FuL), they accumulated PrP^Sc^at passage #30 (P30). However, PrP^Sc^was not detected in GT1-7 cells at P30, which exposed to 10^−5^BH prepared from a tertiary passaged mouse that developed FuS disease phenotype (termed as GT/FuS). Moreover, CD-1 mice inoculated with GT/FuS cell homogenate did not develop any clinical signs of the disease and did not accumulate PrP^Sc^until death (survival range 390–732 days). These results indicate that only FuL prions were amplified and FuS prion is absent in GT1-7 cells exposed to 10^−5^BH prepared from a mouse that developed the FuL disease phenotype. *De novo* generation of FuS prion was confirmed at the tertiary passage of the cloned FuL prion (GT/FuL cell homogenate) in CD-1 mice based on their clinical signs, brain pathology and conformational change of PrP^Sc^detected by seprion-ligand assay using ME7-like prion specific antibody.

**Conclusions**: These results indicate *de novo* generation of FuS prion from FuL prion, due to conformational mutations of PrP^Sc^during serial transmission processes in wild-type mice. Our study may provide an answer to the long-term question of whether some prion strains surfaced by conformational mutations of PrP^Sc^in mice or not.

**Funded by**: Ministry of Health, Labour and Welfare

**Grant number**: 20KA1003

**Acknowledgement**: We appreciate Naoko Tabeta, Naomi Furuya, and Hiroyuki Okada for their technical assistance and constructive comments.

**Fighting prion diseases with released PrP (fragments): transgenic overexpression of N1(Fc) prolongs incubation time in RML-infected mice**

B. Mohammadia F. Song^a^, M. Shafiq^a^, A. Matamoros-Angles^a^, S.Brenna^b^, B.Puig^b^, G. Galliciotti^a^, M. Glatzel^a^, and H. C. Altmeppen^a^

^a^Institute of Neuropathology, University Medical Center Hamburg-Eppendorf (UKE), Hamburg, Germany; ^b^Department of Neurology, Experimental Research in Stroke and Inflammation (ERSI), UKE, Hamburg, Germany

**Aims**: While protective functions of PrP`s main soluble cleavage fragment (N1) have been described against toxic amyloid-beta conformers, little is known on N1`s role in prion diseases. We here investigated neuroprotective effects of an Fc-tagged N1 fragment in murine prion disease.

**Material and Methods**: Since the disordered N1 fragment alone, when transgenically expressed, cannot be secreted due to impaired shuttling into the ER through the translocon, we generated and characterized new transgenic mice overexpressing N1Fc on wild-type background. These mice (TgN1Fc) and their WT littermate controls were intracerebrally inoculated with RML prions. Animals were sacrificed either at matched time points (i.e., 60 and 100 days post-inoculation (dpi)) or at terminal disease stage. Brains were collected and analyzed biochemically and histologically.

**Results**: Overexpression of Fc-tagged N1 does not cause overt immunological or other adverse effects. In contrast, heterozygous TgN1Fc mice showed a moderately, yet significantly, prolonged survival time upon prion inoculation compared to WT controls. PrP^Sc^levels in brain were similar between TgN1Fc and WT mice at terminal disease stage. However, at the preclinical matched time point (100 dpi), lower PrP^Sc^levels were detected in TgN1Fc compared to matched WT mice, and this difference was even more pronounced at 60 dpi, suggesting that transgenic N1(Fc) impairs prion conversion. Detailed histological and biochemical workup (signaling cascades, etc.) is currently under way to further investigate the underlying protective effect(s).

**Conclusions**: Fusion of (unstructured) N1 to an Fc tag (of murine IgG) serving as a structural domain necessary for ER translocation enables secretion (overcoming the previously encountered problem of cytosolic retention of transgenically overexpressed N1 alone) and may improve biostability. Despite a rather mild overexpression of the N1Fc fusion protein, disease progression is slowed down as judged by survival time. Detection of lower PrP^Sc^levels in TgN1Fc mice (compared to matched controls) at preclinical time-points supports a beneficial role of extracellular N1(Fc). Further in vivo studies are ongoing to assess a protective role of N1 in different neurodegenerative diseases as well as in other pathological conditions in the brain, such as ischemic stroke.

**Funded by**: Creutzfeldt-Jakob Disease (CJD) Foundation, Inc.; Alzheimer Forschung Initiative (AFI e.V.); Deutsche Forschungsgemeinschaft (DFG) Collaborative Research Center 877 (SFB877)

**Acknowledgement**: We thank all generous donors enabling the support of this work by the CJD Foundation and AFI e.V.. We thank Dr. Irm Hermans Borgmeyer for her great contribution in generating the transgenic N1Fc mice, and Kristin Hartmann (UKE Mouse Pathology Core Unit) for her help with the immunohistochemistry.

**Defining the onset of prion infection and neurodegeneration in healthy individuals at risk of prion disease**

TH Mok^a,g^, A Nihat^a,g^, D Sequeira^a,g^, T Coysh^a,g^, L Holm-Mercer^a,g^, L Darwent^a^, M Batchelor^a^, J Bieschke^a^, G Jackson^a^, I Swift^b^, J Rohrer^b,d^, A Keshavan^b,d^, J Schott^b,d^, R Paterson^b,d^, E Veleva^b^, R Laban^b^, A Heslegrave^b^, M Chapman^f^, MP Lunn^f^, H Zetterberg^b,c^, C Orru^h^, B Groveman^h^, A Hughson, B Caughey^h^, P Rudge^a,g^, J Collinge^a,g^, and S Mead^a,g^

^a^Medical Research Council Prion Unit at University College London, London, UK; ^b^United Kingdom Dementia Research Institute at University College London, London, ^c^Department of Psychiatry and Neurochemistry, Sahlgrenska Academy at the University of Gothenburg, Mölndal, Sweden; ^d^Dementia Research Centre, Department of Neurodegenerative Disease, University College London Queen Square Institute of Neurology, London, UK; ^e^Institute of Neurology, University College London, London, UK; ^f^Neuroimmunology and CSF laboratory, University College London Hospitals NHS Trust National Hospital of Neurology and Neurosurgery, London, UK; ^g^NHS National Prion Clinic, University College London Hospitals NHS Trust National Hospital of Neurology and Neurosurgery, London, UK; ^h^Laboratory of Persistent Viral Diseases, Rocky Mountain Laboratories, National Institute for Allergy and Infectious Diseases, National Institutes of Health, Hamilton, Montana, USA

**Aims**: The feasibility of preventive strategies in individuals at risk of inherited prion disease and iatrogenic Creutzfeldt-Jakob disease is hampered by the unpredictability of knowing when, or even if, the disease will start. We describe the systematic accrual of a longitudinal biofluid resource, optimisation and detection of cerebrospinal fluid (CSF) prion seeding activity, and measurement of candidate proximity biomarkers in at-risk and symptomatic prion disease cohorts.

**Material and Methods**: We assembled a longitudinal biofluid sample archive from those at-risk of IPD (plasma 217; CSF 67; unique individuals E200K 22, P102L 33 and others 15 (D178N-129 M, D178N-129 V, A117V, 5-OPRI & 6-OPRI)) and iCJD (3;5), symptomatic IPD (62;22), and CJD (40;17) individuals recruited to the National Prion Monitoring Cohort (NPMC). Non-prion (70) and normal CSF controls (24), and normal plasma controls (94) were sourced from NPMC internally and collaborators. IPD CSF (E200K, P102L, P105S, D178N-129 M, Y163X, 6-OPRI) were tested with standard IQ-CSF RT-QuIC, before altering key assay components to optimise for specific mutations before finally, the entire CSF collection was screened with the most sensitive assay. Glial fibrillary acidic protein (GFAP), neurofilament light (NfL), Tau and ubiquitin carboxy-terminal hydrolase L1 (UCH-L1) levels were measured in plasma and CSF using N4PB digital immunoassay platform. Values were log_10_ transformed, and age-normalised for GFAP, NfL and Tau.

**Results**: The IQ-CSF RT-QuIC was 100% sensitive and specific for sCJD, iCJD and familial CJD (E200K + 6-OPRI-CJD); a bespoke Hu P102L RT-QuIC showed partial sensitivity for P102L disease (sensitivity 44.4%; specificity 98.2%). Presymptomatic seeding activity was present in E200K (4) and P102L (1) at-risk samples, between 0.1–7.1 years before estimated/actual disease onset. Of these, an E200K carrier converted, while 2 other E200K carriers (3 samples) remain asymptomatic; the sole positive P102L sample is from an asymptomatic untested individual. No compatible RT-QuIC assay conditions were found for A117V, D178N-129 M, classical 6-OPRI. N4PB analysis revealed statistically significant differences in mean plasma GFAP and NfL, and CSF NfL levels with an increasing trend between normal controls, IPD<2 yrs to onset, sIPD, and CJD cohorts (p < 0.01).

**Conclusions**: Development and application of bespoke RT-QuIC assays compatible with *PRNP* mutation/phenotype, complemented by serial plasma (GFAP & NfL) and CSF (NfL) biomarker measurements, show promise in identifying converting at-risk individuals, which may be useful for therapeutic preventative trial patient selection. Our low conversion numbers however highlight the need to establish longitudinal biofluid archive and protocol standardisation to enable multicentre collaborations.

**Funded by**: Alzheimer’s Society UK and Medical Research Council Core Support for MRC Prion Unit at UCL

**Grant number**: 341

**Acknowledgement**: We wish to thank all the NPMC participants, patients and relatives, and volunteers who have contributed tremendously to this research work over 13 years, a lot of whom did so under very difficult conditions. We wish to also thank the CJD Support Network for a research grant towards purchase of a BMG Labtech FLUOstar Omega Lite microplate reader.

**Detection of misfolded proteins and other biomarkers in the blood and cerebral spinal fluid of the naturally occuring syndrom canine cognitive decline**

Julie A. Moreno^a^, Amelia Hines^a^, Breonna Kusick^b^, Amanda Latham^a^, McKenzie Richards^a^, Savannah Rocha^c^, Brittney MacQuiddy^b^, Ronald B. Tjalkens, and Stephanie McGrath^b^

^a^Department of Environmental and Radiological Health Sciences, Colorado State University, Fort Collins, CO, USA; ^b^Clinical Sciences, Colorado State University, Fort Collins, CO, USA; ^c^Microbiology, Pathology and Immunology, College of Veterinary Medicine and Biomedical Sciences, Colorado State University, Fort Collins, CO, USA

**Aims**: Aged canines develop many features of human aging including cognitive decline and neuropathologies including neuroinflammation and accumulation of misfolded proteins. The only current antemortem diagnostic assay available for veterinarians to diagnose canine cognitive dysfunction (CCD) using clinical signs and magnetic resonance imaging (MRI), mostly to rule out any other causes including brain infections and cancer. The development of antemortem diagnostics using both blood and cerebral spinal fluid (CSF) samples is vital to the early diagnosis of these aged dogs. However, this is challenging due to low concentrations of the common cognitive decline biomarkers found in samples such as misfolded protein amyloid-beta (Aβ), hyperphosphorylation of tau, the inflammatory marker, glial fibrillary acidic protein (GFAP), and neurofilament light chain (NfL).

**Material and Methods**: We have been able to concentrate our samples by extracting the extracellular vesicles to detect Ab, hyperphosphorylation of tau (P-tau), and GFAP in aged dogs and dogs with CCD.

**Results**: We have been able to correlate these changes with pathological brain changes on some of our aged canine tissue, detecting a change in pathologies and blood from the same dog.

**Conclusions**: These sensitive and specific diagnostic assays would improve the ability of veterinarians to accurately diagnose CCD early. Critically, CCD has also been shown by others to be a fantastic translational animal model for Alzheimer’s disease and other dementias in humans.

**Strain Profiles of Sporadic Creutzfeldt-Jakob Disease in Canada**

Jennifer L. Myskiw^a,b^, Lise Lamoureux^a^, Michael Coulthart^c^, Valerie Simd,and Stephanie Booth^a,b^

^a^One Health, Public Health Agency of Canada, Winnipeg, Canada; ^b^Medical Microbiology and Infectious Diseases, University of Manitoba, Winnipeg, Canada; ^c^Canadian CJD Surveillance System, Public Health Agency of Canada, Ottawa; ^d^Division of Neurology, Department of Medicine Centre for Prions and Protein Folding Diseases, University of Alberta, Edmonton

**Aims**: Sporadic Creutzfeldt-Jakob disease (sCJD) is the most widespread human prion disease with an annual prevalence of 1–2 cases per million individuals. sCJD is heterogenous; presenting with diverse clinical signs, neuropathological profiles and molecular subtypes. It is believed this variability is attributed to host genetics and strain properties of the pathogenic prion protein (PrP^Sc^). While the molecular basis of strain diversity is not fully understood, research indicates strain-specific properties are driven by conformational/structural states of PrP^Sc^. In the Canadian population, sCJD strain diversity is poorly understood and remains to be explored experimentally. For these reasons, we are undertaking a systematic analysis of the molecular diversity of CJD cases in Canada. We aim to develop and validate novel methodologies in CJD surveillance research that exploit strain-specific properties of PrP^Sc^. Additionally, by applying these methodologies to analyse PrP^Sc^in a Canadian cohort, we intend to enhance our current understand of the strains affecting the Canadian population.

**Material and Methods**: Firstly, MRIs are being reviewed and pathology information from over 40 cases of CJD are analyzed to select clinically affected areas for investigation. Biochemical analysis will include assessment of the levels of protease sensitive and resistant prion protein, glycoform-ratio analysis, temperature and detergent denaturation profiles, and PrP^Sc^seeding profiles using real-time quaking-induced conversion assays.

**Results**: From the cohort of over 40 Canadian CJD cases, we have identified three atypical CJD cases as well as two cases of variably protease-sensitive prionopathies. One of the atypical CJD cases presented with increased resistance to temperature denaturation as well as a slow seeding pattern. The second atypical CJD case presented with a fast seeding pattern. Finally, the third atypical case presented with an unusual 20kDa protease-resistant core. The two variably protease-sensitive prionopathies identified in this cohort presented with slow seeding profiles as well as a low molecular-weight protease resistant fragment visualized with capillary-based electrophoresis.

**Conclusions**: Throughout this study, we have developed assays that detect and characterize atypical PrP^Sc^profiles. We believe these atypical cases may represent novel strains of CJD, future work involves passaging the atypical isolates into an animal model to further characterize these prion proteins. A deeper knowledge of prion strains within a population has implications for the design and development of future anti-prion therapeutic strategies.

**Funded by**: Public Health Agency of Canada

**An undiagnosed case of prion disease found in donated bodies for anatomical practice of medical students**

Takehiro Nakagaki^a^, Miho Kaneko^a^, Akio Akagi^b^, Yasushi Iwasaki^b^, Katsuya Satoh^c^,and Noriyuki Nishida^a^

^a^Department of Molecular Microbiology and Immunology, Nagasaki University Institute of Biomedical Sciences, Nagasaki, Japan; ^b^Department of Neuropathology, Institute for Medical Science of Aging, Aichi Medical University, Nagakute, Japan; ^c^Health Sciences, Unit of Medical and Dental Sciences, Nagasaki University Institute of Biomedical Sciences, Nagasaki, Japan

**Aims**: Human prion diseases comprise infectious, genetic and sporadic disorders characterized by the accumulation of abnormal prion protein (PrP^Sc^) in the central nervous system. The transmission of prions to patients is known to have occurred by transplantation of infected cadaveric dura mater grafts and corneas, and from exposure to contaminated instruments during neurosurgery. In medical, forensic, and surgical training procedures too, the potential for exposure to prions through accidental contact with tissues from prion-infected cadavers is of concern as prions are not inactivated by formalin fixation. For this reason, we started screening for undiagnosed case of prion disease in the donated bodies received by our institution for anatomical practice from 2020.

**Material and Methods**: The frontal cortex of cadavers were collected and tested by Real Time Quaking Induced Conversion (RT-QuIC) method.

**Results**: In 2020, frontal cortex specimens from 36 cadavers were tested and all were negative. In 2021, a further 44 brains were tested and one was found to be positive. We repeated the test on four additional frontal lobe specimens and all were positive. Samples from the temporal, parietal, and occipital lobes, and these homogenates were also positive wheras samples from the basal ganglia, thalamus, and medulla oblongata were negative. By the hisotopathological analysis, extensive spongiform changes in the neocortex were observed. For these reasons, we concluded that this cadaver is an undiagnosed case of prion diseases.

**Conclusions**: Although our sample size was small, we were able to identify a previously undiagnosed case of prion disease after examination of the brains from only 80 donated cadavers, suggesting that prion carriers could potentially be more common, particularly among the elderly, than would be suggested by the prevalence of prion diseases. Thus, for the safety of surgeons and recipients of transplants, as well as medical personnel and students working with donated cadavers, a systematic prion-screening system for donated bodies and body parts is needed.

**Funded by**:^a^Grant-in-Aid for Scientific Research (C) from Japan Society for the Promotion of Science (JSPS), ^b^Grant-in-Aid of the Research Committee of Prion Disease and Slow Virus Infection from the Ministry of Health, Labour and Welfare of Japan

**Grant number**:^a^JP21K07276, ^b^H29-036

**Acknowledgement**: We thank Prof. Tetsuyuki Kitamoto for giving the advices and discussion.

**Robust hematogenous prion detection in CWD-infected deer throughout disease course**

Amy V. Nalls, Erin E. McNulty, Nathaniel D. Denkers, Edward A. Hoover, and Candace K. Mathiason

Department of Microbiology, Immunology, and Pathology, Colorado State University, Fort Collins, CO USA

**Aims**: There is evidence that infectious prions circulate in the bloodstream of animals infected with prion disease, including cervids (chronic wasting disease), sheep (scrapie), cattle (bovine spongiform encephalopathy), and humans (Creutzfeldt-Jakob disease). The development of rapid, sensitive, and specific assays to detect blood-borne prions has been challenging due to presumed low concentrations of prions in the blood as well as assay inhibitors. Our laboratory has performed extensive chronic wasting disease studies in the native white-tailed deer host, providing a unique repository of serially collected samples from the asymptomatic stage through clinical disease. This repository, in addition to ongoing CWD studies in deer, has permitted us the opportunity to evaluate hematogenous prion load in samples collected minutes, days, weeks and months post exposure using enhanced amplification techniques.

**Material and Methods**: White-tailed deer were orally inoculated with CWD+ or negative deer brain homogenate and blood was collected at 15, 30, and 60 minutes post inoculation, then at 2–4 weeks, and every 3 months until termination in clinical disease. Buffy coat cells were isolated from these samples, counted with a hemacytometer, and assessed for prion seeding activity in an optimized RT-QuIC assay using lipase iron-oxide bead extraction (LIQ). The PMCA/RT-QuIC assay was used for confirmation.

**Results**: Low cell numbers were sufficient to initiate prion seeding activity in LIQ and PMCA/RT-QuIC assays. We detected seeding activity in buffy coat cell fractions harvested as early as 15 minutes post inoculation, throughout asymptomatic disease, peaking at clinical disease.

**Conclusions**: Detection of prion seeding activity within the first hour after inoculation suggests the immediate detection of inoculum after crossing mucosal surfaces and entering the bloodstream. Ongoing detection in serial collections throughout the disease course suggests blood-borne prionemia. These findings allow further assessment of the role hematogenous prions play in the pathogenesis of CWD and provide the tools for examining prionemia in naturally-infected cervid populations.

**Funded by**: HHS-NIH-NIAID

**Grant number**: 2R01AI112956-06

**Acknowledgement**: We thank our undergraduate researchers for their help with sample processing and organization.

**Chronic wasting disease prions in mule deer interdigital glands**

Ness, Anthony^a,b^, Zeng, Doris^c, d^, Kuznetsova, Alsu^b,e^, Otero, Alicia^a,b,f^, Kim, Chiye^a,b^, Saboraki, Kelsey^g^, Lingle, Susan^g^, Pybus, Margo^a,h^, Aiken, Judd^b,e^, Gilch, Sabine^c,d^, and McKenzie, Debbie^a,b^

^a^Department of Biological Sciences, University of Alberta, Edmonton, Alberta, Canada; ^b^Centre for Prions and Protein Folding Diseases, Edmonton, Alberta, Canada; ^c^Department of Comparative Biology and Experimental Medicine, University of Calgary, Calgary, Alberta, Canada; ^d^Hotchkiss Brain Institute, University of Calgary, Calgary, Canada; ^e^Department of Agricultural, Food and Nutritional Sciences, University of Alberta, Edmonton, Alberta, Canada ^f^**Present Address**: Centro de Encefalopatías y Enfermedades Transmisibles Emergentes, Universidad de Zaragoza, Zaragoza, Spain; ^g^Department of Biology, University of Winnipeg, Winnipeg, Manitoba, Canada, ^h^Alberta Environment and Parks, Alberta, Canada

**Aims**: We sought to examine deer skin scent glands for CWD prions. We hypothesized that these glands could contribute to CWD transmission.

**Material and Methods**: Hunter-harvested deer tissues were examined by immunohistochemistry for CWD prions. Interdigital gland homogenates were assayed for prions by real-time quaking-induced conversion (RT-QuIC). Interdigital gland homogenates and soil and was assayed for prions by serial protein misfolding cyclic amplification (sPMCA).

**Results**: We identified CWD prions within the interdigital glands of mule deer, sac-like exocrine structures found between digits of the hooves. Immunohistochemical analysis of interdigital glands from a CWD-infected female mule deer identified disease-associated PrP^CWD^within clusters of infiltrating leukocytes adjacent to sudoriferous and sebaceous glands, and within the acrosyringeal epidermis of a sudoriferous gland tubule. Soil retrieved from between the digits of a clinically affected mule deer amplified proteinase K-resistant PrP material by sPMCA – possibly originating from the interdigital glands. Analysis (blinded) of interdigital glands from 11 mule deer by RT-QuIC accurately identified CWD-infected animals.

**Conclusions**: These data suggest that interdigital glands may play a role in the dissemination of CWD prions into the environment; warranting future investigation.

**Funded by**: Alberta Prion Research Institute (APRI); Genome Canada; NSERC

**Grant number**: PEX19011

**Acknowledgement**: We thank Dr. Nick Nation for assisting with identification of the acrosyringeal histological structures. We thank Nathalie Daude and Trang Nguyen from the University of Alberta CPPFD histological core services for their work.

**A dividing cell model for stable propagation and curing of *bona fide* human sporadic Creutzfeldt-Jakob Disease prions**

Akin Nihat^a,b^, Parineeta Arora^a^, Melissa Rayner^a^, Christian Schmidt^a^, Jacqueline Linehan^a^, Sebastian Brandner^a,c^, Simon Mead^a,b^, John Collinge^a,b^, Parmjit Jat^a^

^a^MRC Prion Unit at UCL, UCL Institute of Prion Diseases, London, UK; ^b^National Prion Clinic, National Hospital for Neurology and Neurosurgery, University College London Hospitals NHS Foundation Trust, London, UK; ^c^Division of Neuropathology, National Hospital for Neurology and Neurosurgery, London, UK

**Aims**: Establishing cell-based models of human prion infection and propagation has been an important, yet elusive, goal of the prion field for decades. The repeated failure of therapeutics identified by screening with non-human species has renewed the search for cell models that allow interrogation of native human prions, in a robust and high-throughput system that propagates *bona fide* infectivity. Here, we present the first reported dividing cell model capable of persistent infection and clearance of human sporadic Creutzfeldt-Jakob Disease (sCJD) prions.

**Material and Methods**: We used a short hairpin RNA and anti-PrP FACS to knockdown murine *Prnp* in CAD5 cells. CAD5-PrP^−/−^ cells were reconstituted with human PrP expressing valine at codon 129, via a pLNCX2 retroviral vector. Subclones were screened for sCJD prion susceptibility using infected human brain homogenate. Pooled lysates from two CAD5-PrP^−/−^(HuPrP) cell clones exposed to sCJD-infected brain homogenate were inoculated into Tg(HuPrP129V^+/+^*Prnp^0/0^*)-152 (Tg152) mice, which were monitored for clinical signs of prion disease. Promising cell clones were iteratively subcloned and screened for prion susceptibility at increasing dilutions. Susceptible cells were used to establish chronic infection, and treated with anti-prion therapeutic agents to demonstrate PrP^Sc^clearance.

**Results**: CAD5-PrP^−/−^(HuPrP) cell subclones were identified with reproducible susceptibility to infection with prions from sporadic CJD patients with *PRNP* codon 129 VV/MV genotypes (types 2/3, London classification; Parchi classification types 1/2). A CAD5-PrP^−/−^(HuPrP) cell subclone was exposed to 0.03% frontal cortex homogenate containing T3MV (MV2) sCJD strain prions, and serially passaged to dilute the inoculum. Pooled cell lysate was inoculated into Tg152 mice, resulting in a 100% clinical attack rate (18/18 mice, mean incubation period 251 ± 4 days post-inoculation). 9/9 of mice inoculated with 1% frontal cortex from the same patient were clinically affected, mean incubation 235 ± 15 DPI. Histopathological examination and immunoblot analysis for PrP^Sc^were identical between both groups. Sequential subcloning resulted in a cell line reliably susceptible to sCJD prion infection using frontal cortex homogenates diluted to approximately 10^−5^– 10^−6^. The most susceptible subclone was used to establish a pilot semi-automated robotic assay, persistently-infected cells that allow cryopreservation, and to demonstrate PrP^Sc^clearance with anti-prion therapeutic agents.

**Conclusions**: We have engineered the first immortalised cell line that can reproducibly propagate infective human sCJD prions, maintain and be cured of persistent infection, and is amenable to high-throughput assay. We believe these cells will be of considerable value in investigating putative prion therapeutics, factors associated with human prion cell and strain tropism, and infectivity.

**Funded by**: Akin Nihat is supported by a MRC Clinical Research Training Fellowship (grant number MR/P019862/1)

**Grant number**: MR/P019862/1

**Acknowledgement**: We thank all the affected individuals who kindly donated the tissue that supported this work.

**Amyloidogenesis of SARS-COV-2 Spike protein cause impaired fibrinolysis *in vitro***

Sofie Nyström, and Per Hammarström

IFM-Chemistry, Linköping Univeristy, Linköping, Sweden

**Aims**: SARS-CoV-2 infection is associated with a number of symptoms with striking similarities to blood coagulation and fibrinolytic disturbances as well as neurologic and cardiac problems associated with amyloid disease. This led us to perform *in vitro* investigation of SARS-CoV-2 spike protein (S-protein) amyloidogenicity. The leucocyte subclass Neutrophils are highly abundant at site of infection in COVID-19 patients. A major mode of action for Neutrophils is the release of Neutrophil elastase (NE). This protease will digest intruding pathogens as a first response to infection. The enzyme is often entrapped in Neutrophil extracellular traps (NETs) to keep the proteolytic activity near the infected site rather than floating away in the blood stream. We therefore also investigated if Spike amyloid fibrils influences the fibrin(ogen)-fibrinolysis system *in vitro*.

**Material and Methods**: Seven amyloidogenic amino acid sequences in the SARS-COV-2 S-protein (Wuhan strain) were identified using the WALTZ algorithm. Peptides were synthesized based on the prediction and were subjected to amyloid formation conditions *in vitro*. To verify their amyloidogenicity, the fibrillation kinetics was monitored by ThT and the endpoint samples were analyzed using transmission electron microscopy (TEM) and Congo red birefringence (CR). Recombinant expressed full-length S-protein was subjected to cleavage by neutrophil elastase *in vitro*. An *in vitro* fibrin(ogen)-fibrinolysis assay in absence and presence of Spike amyloids was performed and monitored by sample turbidity by absorption at 350 nm. Mass spectrometry was also performed at endpoint of elastase cleavage to determine which peptides were abundant after intermediate and complete cleavage of S-protein.

**Results**: Three 20-amino acid long synthetic spike peptides fulfilled our three amyloid fibril criteria: nucleation dependent polymerization kinetics by ThT, Congo red positivity, and ultrastructural fibrillar morphology. All seven peptides fulfilled at least one of the three criteria. Full-length folded S-protein by itself did not form amyloid fibrils. However, amyloid-like fibrils with evident branching were formed during 24 h of S-protein coincubation NE *in vitro*. Prescence of spike amyloid during fibrin formation from fibrinogen resulted in impairment of fibrinolysis.

**Conclusions**: Our data propose a molecular mechanism for potential amyloidogenesis of SARS-CoV-2 S-protein in humans facilitated by endoproteolysis. S-protein amyloid fibrils cause fractions of fibrin to be plasmin-resistant *in vitro* suggestively associated with pathogenic microclotting *in vivo*. The prospective of S-protein amyloidogenesis in COVID-19 disease associated pathogenesis can be important in understanding the disease and long COVID-19 in relation to diverse SARS-CoV-2 strains.

**References**: https://doi.org/10.1021/jacs.2c03925

**Funded by**: Swedish research council

**Grant number**: 2019–04405

**Chronic wasting disease risk assessment in Portugal: analysis of variability and genetic structure of the Portuguese roe deer population**

Nuno Gonçalves-Anjo^a,b^, Jorge C. Pereira^c^, Ana Machado^a^, Mafalda Saianda^a^, Estela Bastos^a,b^, Ana C. Matos^d^, Adelina Gama^c^, Anabela Alves^c^, Alexandra Esteves^c^, Sara Rocha^a^, Luís Figueira^d^, Carla Lima^e^, Filipe Silva^c^, Fernanda Seixas^c^, Isabel Pires^c^, João Silva^e^, Madalena Vieira-Pinto^c^, Maria L. Pinto^c^, Paula Mendonça^e^, Paulo Carvalho^e^, Paula Tavares^e^, Roberto Sargo^c^, Leonor Orge^c,e^, and Maria A. Pires^c^

^a^Department of Genetics and Biotechnology, University of Trás-os-Montes and Alto Douro (UTAD), Vila Real, Portugal; ^b^Centre for the Research and Technology of Agro-Environmental and Biological Sciences (CITAB), UTAD, Vila Real, Portugal; ^c^Veterinary and Animal Science Research Center (CECAV), AL4AnimalS, UTAD, Vila Real, Portugal; ^d^Polytechnic Institute of Castelo Branco (IPCB), Castelo Branco, Portugal; ^e^Pathology Laboratory, UEISPSA, National Institute for Agricultural and Veterinary Research (INIAV), I.P., Oeiras and Vairão, Portugal

**Aims**: Among the Transmissible Spongiform Encephalopathies, Chronic Wasting Disease (CWD) in cervids is now the rising concern in wildlife within Europe after the first case detected in Norway in 2016. CWD shows a notable horizontal transmission, affecting both free-ranging and captive cervids. Furthermore, several genetic variants in the Prion Protein (*PRNP*) gene coding sequence of the cervid were identified, which increase the susceptibility to the disease.

Roe deer has a widespread geographical distribution which extends from the Iberian Peninsula to the north of Scandinavia, being found as well in Turkey, Israel, and Jordan. In recent centuries, a significant number of fluctuations have occurred in these deer populations, leading to a decline in deer population and distribution. These alterations combined with translocations of animals of the same species, can drive to meaningful consequences on the genetic structure, diversity, and fitness of populations.

**Material and Methods**: In this study we aimed to outline the genetic diversity and structure of Western Iberian roe deer populations using mitochondrial DNA (mtDNA) molecular analysis and link to sequence diversity of *PRNP* gene performed to evaluate the susceptibility to CWD of this population.

**Results**: The preliminary results from the analysis of 60 roe deer samples from different Western Iberian areas (wild and fenced populations) allowed the confirmation of the *consensus PRNP* gene CDS sequence and revealed the presence of 13 haplotypes by mtDNA molecular analysis.

**Conclusions**: These studies are of great importance to obtain information about the phylogeography, which can be used to design appropriate strategies for the conservation and management of populations, but as well to maintain the genetic heritage of roe deer in Europe. Moreover, the establishment of risk assessment projects, even in countries with no cases of CWD is very important to predict the effects of possible contamination in the future.

**Funded by**: This work was supported by the project WastingPrionRisk [POCI-01-0145-FEDER-029,947/PTDC/CVT-CVT/29947/2017] funded by the Portuguese Foundation for Science and Technology (FCT). FCT PhD grant [SFRH/BD/146961/2019] financed by FCT through FSE (Fundo Social Europeu). This work was also supported by national funds [UIDB/CVT/00772/2020], [LA/P/0059/2020] and [UIDB/04033/2020] by FCT.

**Grant number**: [POCI-01-0145-FEDER-029,947], [PTDC/CVT-CVT/29947/2017], [UIDB/CVT/00772/2020], [LA/P/0059/2020], [UIDB/04033/2020] and [SFRH/BD/146961/2019].

**DTI Abnormalities in Healthy E200K Carriers May Serve as an Early Biomarker for Genetic Creutzfeldt-Jakob Disease (gCJD)**

Omer Nurit^a,b,e^, Silbak Rawan^c^, Trabulus Noa^a^, Shiner Tamara^a,b,c^, Moore Orna^a^, Gana-Weisz Mali^d^, Goldstein Orly^d^, Glinka Tal^d^, Orr-Urterger Avi^b,d^, Giladi Nir^a,b,c^, Droby Amgad^a,c,e^, and Bregman Noa^a,b^

^a^Cognitive Neurology Unit, Neurological Institute, Tel-Aviv Medical Center; ^b^Sackler School of Medicine, Tel-Aviv University; ^c^Sagol School of Neuroscience, Tel-Aviv University; ^d^Genomic Research Laboratory for Neurodegeneration, Tel-Aviv Medical Center; ^e^Laboratory of Early Markers of Neurodegeneration, Neurological Institute, Tel-Aviv Medical Center, Tel-Aviv, Israel

**Aims**: To investigate microstructural changes in healthy E200K carriers using diffusion tensor imaging (DTI).

**Material and Methods**: Seven symptomatic gCJD patients and N = 60 healthy relatives of gCJD patients were included. Participants underwent genetic testing for the E200K mutation, MRI scans at 3T, and a lumbar puncture (LP) for total Tau protein levels (t-Tau). Diffusion tensor imaging (DTI) metrics including; fractional anisotropy (FA), mean diffusivity (MD), radial diffusivity (RD) and axonal diffusivity (AD) were calculated along 45 WM tracts.

**Results**: N = 30 participants were found to be E200K carriers (mean age 56.73±7.27, 18 F). Of those; N = 23 underwent an LP, and 8 showed above normal t-Tau values (> 290 pg/ml). Higher MD, RD as well as lower FA values were observed in symptomatic CJD patients compared to healthy relatives in several WM tracts (Two-samples t-test; *p* < 0.05). No significant differences in FA, MD, AD and RD were detected between healthy carriers and healthy non-carriers within the WM tracts. Finally, significantly higher FA and lower MD, RD, and AD were found in carriers with high level of t-Tau compared to carriers with normal levels of t-Tau along several WM tracts (Two-samples t-test; *p* < 0.05).

**Conclusions**: DTI abnormalities along WM tracts were found in gCJD patients and in healthy E200K carriers with elevated t-Tau in CSF. These findings suggest a possible role for DTI imaging as a non-invasive biomarker for prodromal gCJD. Ongoing work is focused on establishing the sensitivity of further DTI-derived measures and additional MRI markers prior to phenoconversion.

**Funded by**: IONIS pharmaceuticals

**Cerebrospinal fluid (CSF) and Plasma Biomarkers in patients with genetic Creutzfeldt-Jakob disease (gCJD) and healthy relatives, carriers of the E200K mutation: Results from an ongoing longitudinal study**

Omer N^a,b^, Shiner T^a,b^, Trablus N^a^, Silbak R^b^, Bar-David A^a^, Kave G^a,b^, Alcalay Y^a^, Gana-Weiss M^a,b^, Goldstein O^a,b^, Orr-Urteger A^a,b^, Glinka T^a^, Giladi N^a,b^, Moore O^a^, and Bregman N^a,b^

^a^The Neurological Institution, Tel-Aviv Medical Center, Tel-Aviv, Israel; ^b^Tel-Aviv University, Tel-Aviv, Israel

**Aims**: The genetic form of CJD caused by an E200K mutation on the *PRNP* gene is relatively prevalent among Jews from North African origin. Carriers of the E200K mutation are prone to develop CJD, however age of onset is variable and unpredictable, and the prodromal stage of disease is still not well characterized. A longitudinal study following healthy relatives of gCJD patients, carriers of the E200K mutation, was established aiming to characterize the prodromal stage and identify people at risk for phenoconversion. Here we present the cross-sectional (first year) results of plasma and CSF biomarkers for neurodegeneration in this population.

**Material and Methods**: Clinical data, genetics, plasma and CSF were collected from CJD patients and from healthy first degree relatives of gCJD patients, as part of a longitudinal study conducted in the Tel-Aviv Medical Center. All participants were tested for the E200K mutation on the *PRNP* gene. CSF and plasma were tested for total Tau (t-Tau), Neurofilaments Light (NfL), GFAP, and UCHL1 levels. Real-time quaking-induced conversion (RT-QuIC) was performed on CSF to detect the presence of the misfolded pathological form of prion protein (PrP^Sc^).

**Results**: All gCJD patients (N = 7) and 42/84 healthy relatives were positive for the E200K mutation. No significant differences were found in age (mean age of carriers was 57.5 ± 7.5), gender, clinical and cognitive measures when comparing patients, healthy carriers, and non-carriers. Total-Tau, NfL, and GFAP were significantly higher in CSF and plasma of patients compared to healthy relatives (p < 0.0001). UCHL1 was significantly higher in CSF (p < 0.0001) but not in plasma when comparing patients to healthy relatives (p = 0.3). No significant differences were found in these biomarkers when comparing healthy carriers to non-carriers (p > 0.38). RT-QuIC was positive for the presence of **PrP^Sc^** in all patients, and in 1/42 E200K carriers. In this individual, t-Tau levels in CSF, and levels of GFAP and UCHL1 in plasma were significantly elevated compared to all other healthy relatives. Two months later, she phenoconverted.

**Conclusions**: RT-QuIC for PrP^Sc^and other CSF and plasma biomarkers may serve as a useful tool for the detection of prodromal gCJD in at-risk individuals. The data presented here requires further validation in an ongoing longitudinal follow-up.

**Funded by**: Ionis Pharmaceuticals

**Acknowledgement**: Ionis Pharmaceuticals

**Performance of α-synuclein RT-QuIC in relation to neuropathological staging of Lewy body disease**

Christina D. Orrù, PhD^a^*, Sara Hall, MD, PhD^b,c^*, Geidy E. Serrano, PhD^d^, Douglas Galasko, MD, PhD^e,f^, Andrew G. Hughson, MSc^a^, Bradley R. Groveman, PhD^a^, Charles H. Adler, MD, PhD^g^, Thomas G. Beach, MD, PhD^d^, Oskar Hansson, MD, PhD^b,c^**, and Byron Caughey PhD^a^**

*Both contributing equally as first authors. **Both contributing equally as senior authors. ^a^LPVD, Rocky Mountain Laboratories, NIAID, NIH, Hamilton, MT, USA; ^b^Clinical Memory Research Unit, Department of Clinical Sciences Malmö, Lund University, Sweden; ^c^Memory Clinic, Skåne University Hospital, Malmö, Sweden; ^4^Banner Sun Health Research Institute, Sun City, Arizona, USA; ^5^Department of Neurosciences, University of California San Diego, La Jolla, CA, USA; ^6^Veterans Affairs San Diego Healthcare System, San Diego, CA, USA; ^7^Department of Neurology, Mayo Clinic College of Medicine, Mayo Clinic Arizona, Scottsdale, AZ, USA

**Aims**: α-synuclein (**α**Syn) RT-QuIC is a promising new assay thate addreses the need for a sensitive and specific diagnostic test for Lewy body disorders (LBD). By detecting misfolded αSyn in biological samples, such as CSF, LBD can be detected in clinically or neuropathologically established patients with various synucleinopathies. In this study, we analyzed and compared lumbar CSF in a clinical cohort (Swedish BioFINDER) and postmortem ventricular CSF in a neuropathological cohort [Arizona Study of Aging and Neurodegenerative Disorders/Brain and Body Donation Program (AZSAND/BBDP)] using the αSyn RT-QuIC assay.

**Material and Methods**: The clinical cohort from BioFINDER included 64 PD/PDD, 15 MSA, 15 PSP, 47 controls and two controls who later converted to PD/DLB. The neuropathological cohort from AZSAND/BBDP included 101 cases including LBD and controls, as well as other brain disorders. CSFs from these cohorts were blinded with respect to clinical status, neuropathological status, and diagnosis of the patient and analyzed by **α**Syn RT-QuIC.

**Results: α**Syn RT-QuIC discriminated LBD (i.e. PD, PDD and converters) from controls with a sensitivity of 95% and a specificity of 83% in the clinical BioFINDER cohort. There were two controls that were **α**Syn RT-QuIC positive who later converted to LBD. **α**Syn RT-QuIC analysis of the neuropathological cohort from AZSAND/BBDP, distinguished neuropathologically verified ‘standard LBD’ cases (i.e. PD, PD with AD and DLB; n = 25) from those lacking LB pathology (n = 53) with high sensitivity (100%) and specificity (94%). In the subgroup with ‘non-standard’ LBD (i.e., AD with Lewy bodies not meeting criteria for DLB or PD, and incidental LBD, n = 23) only 57% were **α**Syn RT-QuIC positive. Interestingly, **α**Syn RT-QuIC was effective in discriminating cases with LB pathology in the cortex (97% sensitivity) from cases with no LBs or LBs present only in the olfactory bulb (93% specificity). In cases with LB pathology restricted to the brainstem or amygdala, and not the allocortex or neocortex, the sensitivity was 50%.

**Conclusions**: Testing of CSF specimens with **α**Syn RT-QuIC is highly sensitive and specific for detecting cases with clinicopathologically-defined Lewy body disorders. While the sensitivity is lower for non-standard LBD, asymptomatic LBD, or in cases with modest LB pathology not affecting the cortex, the CSF **α**Syn RT-QuIC assay remains a robust and valuable tool for LBD diagnosis.

**Funded by:/Grant number**: The Swedish Research Council (2016–00906), the Knut and Alice Wallenberg foundation (2017–0383), the Marianne and Marcus Wallenberg foundation (2015.0125), the Strategic Research Area MultiPark (Multidisciplinary Research in Parkinson’s disease) at Lund University, the Swedish Alzheimer Foundation (AF-939932), the Swedish Brain Foundation (FO2021-0293), The Parkinson foundation of Sweden (1280/20), the Konung Gustaf V:s och Drottning Victorias Frimurarestiftelse, the Skåne University Hospital Foundation (2020-O000028), Regionalt Forskningsstöd (2020–0314) and the Swedish federal government under the ALF agreement (2018-Projekt0279). This work was supported in part by the Intramural Research Program of the NIAID, NIH.

We are grateful to the Banner Sun Health Research Institute Brain and Body Donation Program of Sun City, Arizona for the provision of human biological materials. The Brain and Body Donation Program has been supported by the National Institute of Neurological Disorders and Stroke (U24 NS072026 National Brain and Tissue Resource for Parkinson’s Disease and Related Disorders), the National Institute on Aging (P30 AG19610 and P30AG072980, Arizona Alzheimer’s Disease Core Center), the Arizona Department of Health Services (contract 211,002, Arizona Alzheimer’s Research Center), the Arizona Biomedical Research Commission (contracts 4001, 0011, 05–901 and 1001 to the Arizona Parkinson’s Disease Consortium) and the Michael J. Fox Foundation for Parkinson’s Research .

**Acknowledgement**: The authors thank the study participants and their families. Without their invaluable contribution, this study would not have been possible.

**Innate immune tolerance in microglia does not impact on CNS prion disease**

Reiss Pal, Barry M. Bradford, and Neil A. Mabbott

The Roslin Institute & R(D)SVS, The University of Edinburgh. Edinburgh, UK

**Aims**: The microglia provide neuroprotection during prion disease, but their pro-inflammatory activation may exacerbate the development of the neuropathology. Innate immune tolerance to consecutive bacterial lipopolysaccharide (LPS) treatment can induce epigenetic changes in the microglia that can dampen their responsiveness to subsequent LPS-treatment and several months later impede the development of neuritic damage in a transgenic mouse model of Alzheimer’s like pathology. We therefore reasoned that innate immune tolerance in microglia might similarly impede the development of prion disease.

**Material and Methods**: Mice were injected with LPS intraperitoneally once or 4x every 24hrs to induce immune training and tolerance, respectively. Brains and peripheral blood were collected 3 h after the final treatment. Cytokines were analysed by ELISA and RT-qPCR. Glial activation was assessed by RT-qPCR and immunohistochemistry. Mice were infected with ME7 by intracerebral injection, and 35 days later given 4xLPS. Prion protein load was measured by immunohistochemistry and western blot.

**Results**: Pro-inflammatory cytokines Interleukin-1β (IL-1β), IL-6 and tumour necrosis factor-α (TNF-α) were detected in the serum after 1x LPS. However, the levels of IL-6 and TNF-α in 4xLPS-treated mice were undetectable which coincided with increased amount of the anti-inflammatory cytokine IL-10 in the serum. In the brain, *Il1β, Il6* and *Tnf* were induced after 1xLPS treatment. In contrast, expression of *Il1b, Tnf* and *Il6* were reduced after 4xLPS when compared to mice given 1xLPS, and this was accompanied by an increase in *Il10* in the brains of 4xLPS-treated mice. Consistent with these changes, the expression of the microglial homeostatic related genes *Cx3cr1* and *Tmem119* were also significantly increased in the brains of 4xLPS-treated mice. However our data also shows that consecutive systemic LPS treatment did not affect the subsequent development of prion disease. Innate immune tolerance induced after 4x LPS did not affect the expression level of these cytokines at the terminal stage of prion disease when compared to prions+4xPBS controls. However, microglia training in response to 1xLPS treatment coincided with an increase in *Il1β* expression compared to prions+4xPBS controls, and this increase was ablated in the brains of prions+4xLPS treated mice. The level of *Tnf* expression in the brains of prions+4xLPS treated mice was also reduced compared to prions+1xLPS treated mice.

**Conclusions**: Innate immune tolerance in microglia does not influence the subsequent onset of prion disease-induced neuropathology in mice, despite previously published evidence of this effect in an Alzheimer’s disease mouse model.

**Funded by**: This work was supported by project (BB/S005471/1) and Institute Strategic Programme Grant funding from the Biotechnology and Biological Sciences Research Council (grant numbers BBS/E/D/20002173 & BBS/E/D/10002074). RP was supported by a PhD studentship from the Royal (Dick) School of Veterinary Studies (University of Edinburgh, UK).

**Acknowledgement**: We thank Aileen Boyle, Bob Fleming, Graeme Robertson, Lee McManus, the Biological and Veterinary Services, Bioimaging and Pathology Services groups, and the Roslin Institute Histopathology Suite (University of Edinburgh, UK) for helpful advice and technical support.

**Evaluation of the therapeutic action of poly(propylene Imine) glycodendrimers in prion disease mouse model**

Dimitra Paschaloudi^a^, Nikolaos Mpekas^b^, Sotiria Solomou^a^, Eirini Kanata^a^, Dimitra Dafou^b^, Konstantinos Xanthopoulos^a^, Inga Zerr^c^, Matthias Schmitz^c^, D. Appelhans^d^, and Theodoros Sklaviadis^a^

^a^School of Pharmacy, Aristotle University of Thessaloniki, Thessaloniki, Greece; ^b^School of Biology, Aristotle University of Thessaloniki, Thessaloniki, Greece; School of Pharmacy, Aristotle University of Thessaloniki, Thessaloniki, Greece; ^c^Department of Neurology University Medical School Göttingen, Germany; ^d^Leibniz-Institut für Polymerforschung Dresden e.V., Dresden, Germany

**Aims**: Prion Diseases are transmissible proteinopathies that affect the nervous system through the accumulation of the misfolded prion protein. Despite fervent research, an efficient therapeutic approach has not been developed and the diseases remain lethal. A series of poly(propylene imine) (PPI) Glycodendrimers has been generated and their anti-prion efficiency was previously tested on scrapie-infected neuroblastoma cell models (ScN2a) and a cell-free prion amplification assay (RT-QuIC). A dense shell 4^th^generation PPI glycodendrimer (G4-Mal) displayed promising results in these assays was further tested in a murine prion model.

**Material and Methods**: 27 mice (C57Bl/6, female, aged 12 weeks) were housed in a biosafety level 3 facility with controlled temperature, humidity, and photoperiod and *ad libitum* access to water and feed. Mice were challenged i.p. with the RML strain and divided into three groups. The PPI Glycodendrimer was dissolved in drinking water and administered to the first group since day 10 post inoculation (prophylaxis group). The second group received the same treatment since the 90^th^day (treatment group), while the third group did not receive any treatment (control group). Mice were examined for the appearance of symptoms including loss of motor coordination and strength, reduced self-grooming, etc. The progression of the disease was evaluated using a clinical scoring system, in which the presence of each symptom was assigned a score of 0.5 or 1, depending on its severity. The sum of scores characterized the clinical condition of each mouse throughout disease progression.

**Results**: The control group reached an average clinical score above 1.5 in 157 ± 20 days, while the treated groups showed a delay in the onset of symptoms. This delay was significant (p = 0.0048) for the treatment group, (reached average score 1.5 176.3 ± 20 days after inoculation). However, all mice reached a clinical score between 4 and 6 in the later stages of the disease and eventually succumbed without statistically significant differences in the survival time (204 ± 4, 202 ± 3, and 202 ± 6 days after the induction, for the prophylaxis, treatment, and control group respectively). To further characterize the disease progression, the AUC for the clinical scores vs time plots was calculated and found significantly reduced (p = 0,0225) for the treatment group.

**Conclusions**: Our results indicate that although the glycodendrimer did not prolong the survival interval in the animal model used, it led to a delay in disease onset and disease burden. Further studies on the pharmacokinetics and pharmacodynamics of the compound are underway.

**Determining prion protein gene (PRNP) genetic variability in portuguese cervidae population. An important task in chronic wasting disease (CWD) risk assessment projet in Portugal**

Jorge C. Pereira^a^, Nuno Gonçalves-Anjo^b,e^, Estela Bastos^b,e^, Leonor Orge^a,c^, Ana C. Matos^d^, Adelina Gama^a^, Anabela Alves^a^, Alexandra Esteves^a^, Sara Rocha^b,e^, Luís Figueira^d^, Carla Lima^c^, Filipe Silva^c^, Fernanda Seixas^a^, Isabel Pires^a^, João Silva^c^, Madalena Vieira-Pinto^a^, Maria L. Pinto^a^, Paula Mendonça^c^, Paulo Carvalho^c^, Paula Tavares^c^, Roberto Sargo^a^, and Maria A. Pires^a^

^a^Animal and Veterinary Research Centre (CECAV), AL4AnimalS – Associate Laboratory for Animal and Veterinary Sciences, University of Trás-os-Montes e Alto Douro (UTAD), Vila Real, Portugal; ^b^Centre for the Research and Technology of Agro-Environmental and Biological Sciences (CITAB), UTAD, Vila Real, Portugal; ^c^Pathology Laboratory, UEISPSA, National Institute for Agricultural and Veterinary Research, I.P.(INIAV) Oeiras and Vairão, Portugal; ^d^Polytechnic Institute of Castelo Branco (IPCB), Castelo Branco, Portugal; ^e^Department of Genetics and Biotechnology, University of Trás-os-Montes and Alto Douro (UTAD), Vila Real, Portugal

**Aims**: The chronic wasting disease (CWD) in cervids is now a rising concern in wildlife within Europe, since the first case detected in Norway in 2016, 40 more appear until May of 2022, in Norway, Sweden and Finland. The unclear origin of these new European cases and the risk that CWD poses to cohabiting animals or more importantly to humans is largely unknown, is very important for the establishment of risk assessment projects, even in countries with no cases of CWD to forecast possible infections. In this way, a synergistic collaborative project was established between the UTAD, INIAV and IPCB to evaluate the risk of a potential occurrence of CWD in cervid Portuguese populations. The study of prion protein gene, *PRNP*, has been proved to be a valuable tool for determining the relative susceptibility to TSEs since this is influenced by polymorphisms in this gene. The aim of this work is the screening for PrP^res^and determination of the *PRNP* genotyping profile on Portuguese cervids, as the survey and georeferencing of these animals will contribute to delineating the risk of dissemination of CWD in Portugal.

**Material and Methods**: This study includes 200 animals of three different cervid species: red deer (*Cervus elaphus*), fallow deer (*Dama dama*) and roe deer (*Capreolus capreolus*). Masseter muscle and lymph node of each animal were collected for genomic DNA extraction and genetic analysis of prion protein gene, *PRNP*. The full exon 3 of *PRNP* gene (771 bp) was amplified by PCR using the primers F223-ACACCCTCTTTATTTTGCAG and R224-AGAAGATAATGAAAACAGGAAG. PCR products were purified, sequenced, and analyzed using SnapGene Viewer v. 5.1.5, Unipro UGENE v. 40.0 and Jalview 2.11.1.4.

**Results**: The comparison of the coding region of *PRNP* gene and protein sequence – PrP^C^(256 aa) in red deer, fallow deer, and roe deer, showed high conservation. In red deer, three polymorphisms were identified: one synonymous, codon A136A and two non-synonymous codons T98A and Q226E. The synonymous mutation at codon 136 showed to be linked to the non-synonymous mutations at codon 226. Three haplotypes were identified based on the sequence variations: T98-Q226 (TQ), T98-E226 (TE) and A98-Q226 (AQ). In fallow deer and roe deer, no intra variation was found.

**Conclusions**: The multi-disciplinary approaches including genotyping, PrP^res^detection, identification of risk factors, clinical and pathology evaluations, are of great importance to evaluate the risk of occurrence of CWD in Europe and more specifically in Iberian Peninsula. We did not find any positive cases of CWD in the animals under study and the genetic variations do not allow to conclude about its resistance to this disease.

**Funded by**: This work was supported by the project WastingPrionRisk [POCI-01-0145-

FEDER-029,947/PTDC/CVT-CVT/29947/2017] funded by the Portuguese Foundation for

Science and Technology (FCT). FCT PhD grant [SFRH/BD/146961/2019] financed by FCT

through FSE (Fundo Social Europeu). This work was also supported by national funds

[UIDB/CVT/00772/2020], [LA/P/0059/2020] and [UIDB/04033/2020] by FCT.

**Grant number**: [POCI-01-0145-FEDER-029,947], [PTDC/CVT-CVT/29947/2017],

[UIDB/CVT/00772/2020], [LA/P/0059/2020], [UIDB/04033/2020] and

[SFRH/BD/146961/2019].

**Blood microRNA sequencing in prion diseases**

Sonia Pérez Lázaro^a^, Inmaculada Martín-Burriel^b^, Luca Cozzuto^c^, Julia Ponomarenko^c^, Juan J. Badiola^a^, Janne M. Toivonen^b^, and Rosa Bolea^a^

^a^Centro de Encefalopatías y Enfermedades Transmisibles Emergentes, Facultad de Veterinaria, Universidad de Zaragoza – IA2, Zaragoza, Spain; ^b^Laboratorio de Genética bioquímica (LAGENBIO), Facultad de Veterinaria, Universidad de Zaragoza – IA2, Zaragoza, Spain; ^c^Bioinformatics Unit, Centre de Regulació Genòmica (CRG), Barcelona, Spain

**Aims**: MicroRNAs, non-coding small RNA molecules that regulate post-transcriptional gene expression, play a key role in neuronal survival and function, as well as in neuroinflammation. Since more than a half of the protein-coding genes are regulated by microRNAs, alterations in their expression have been associated with neurodegenerative diseases. We have studied microRNAs alterations in scrapie as a model of prion disease. Due to the fatality of these diseases and the lack of in vivo definitive diagnosis, next generation sequencing techniques have become relevant for searching potential biomarkers. Because of their marked stability in body fluids, microRNAs can be considered as potential minimally invasive biomarkers of these diseases, and we have selected blood as an easily accessible body fluid.

**Material and Methods**: In this study, smallRNA sequencing was performed in blood samples from sheep naturally infected with classical scrapie in different stages of the pathogenesis of scrapie infection: 5 sheep in preclinical stage, 10 clinical sheep and 10 negative healthy controls. Afterwards, a bioinformatic analysis was performed. For the validation of results, we used quantitative real-time PCR selecting those with less changes as housekeeping microRNAs and validating the ones with the most significantly altered expression.

**Results**: We identified 136 microRNAs significantly dysregulated in blood from clinical sheep compared to that from negative controls. Specifically, 69 microRNAs were downregulated whereas 67 microRNAs were upregulated. However, only 1 microRNA was found to be significantly dysregulated in preclinical sheep.

**Conclusions**: Our results suggest that most changes in microRNAs are not detected until the onset of clinical symptoms. The alterations shown in the clinical stage may reflect the potential usefulness of microRNAs as biomarkers of prion diseases. In fact, one microRNA was significantly altered in blood in the preclinical stage of the disease suggesting its potential use as a diagnostic tool. To make progress in this field, we will conduct further studies to investigate the microRNAs that were altered and the metabolic pathways that may be involved.

**Funded by, grant number**: RTI2018-098711-B-I00, EFA 148/16

**Application of PMCA to understand CWD prion strains, species barrier and zoonotic potential**

Sandra Pritzkow^a^, Damian Gorski^a^, Frank Ramirez^a^, Fei Wang^a^, Glenn C. Telling^b^, Justin J. Greenlee^c^, Sylvie L. Benestad^d^, and Claudio Soto^a^

^a^Department of Neurology, University of Texas Medical School at Houston, Houston, Texas, USA; ^b^Department of Microbiology, Immunology and Pathology, Colorado State University, Fort Collins, Colorado, USA; ^c^Virus and Prion Research Unit, United States Department of Agriculture, Ames, Iowa, USA; ^d^Norwegian Veterinary Institute, OIE Reference Laboratory for CWD, Ås, Norway

**Aims**: Chronic wasting disease (CWD) is a prion disease affecting various species of cervids that continues to spread uncontrollably across North America and has recently been detected in Scandinavia (Norway, Sweden and Finland). The mechanisms responsible for the natural transmission of CWD are largely unknown. Furthermore, the risk of CWD transmission to other species, including humans, is also unknown and remains a dangerous enigma. In this study, we investigated the potential of CWD prions to infect several other animal species (sheep, cattle, pig, hamster, and mouse) including humans, by examining their capacity to convert the normal prion protein of distinct species in a PMCA reaction. Moreover, we also investigated whether the *in vivo* passage of CWD through intermediate species alters their capacity for zoonotic transmission, which may represent a major hazard to human health.

**Material and Methods**: For these studies, we used brain material from CWD-infected white-tailed deer (*Odocoileus virginianus*), elk (*Cervus canadensis*), and mule deer (*Odocoileus hemionus*) as species native to North America. We also used CWD-infected Moose (*Alces alces*), reindeer (*Rangifer tarandus*) and red deer (*Cervus elaphus*) as Norwegian cervids. We also used brains from cattle, sheep and pigs experimentally infected by CWD. To study interspecies-transmission and zoonotic potential, samples were tested via PMCA for the conversion of PrP^C^into PrP^Sc^using different combinations of inoculum and host species. Based on these analyses we estimated the spillover and zoonotic potential for different CWD isolates. We define and quantify spillover and zoonotic potential indices as the efficiency by which CWD prions sustain prion generation *in vitro* at the expense of normal prion proteins from various mammals and human, respectively.

**Results**: Our results show that prions from some cervid species, especially those found in Northern Europe, have a higher potential to transmit disease characteristics to other animals. Conversely, CWD-infected cervids originated in North America appear to have a greater potential to generate human PrP^Sc^. We also found that *in vivo* transmission of CWD to cattle, but not to sheep or pigs substantially increases the ability of these prions to convert human PrP^C^by PMCA.

**Conclusions**: Our findings support the existence of different CWD prion strains with distinct spillover and zoonotic potentials. We also conclude that transmission of CWD to other animal species may increase the risk for CWD transmission to humans. Our studies may provide a tool to predict the array of animal species that a given CWD prion could affect and may contribute to understanding the risk of CWD for human health.

**Funded by**: National Institute of Health

**Grant number**: P01 AI077774

**A role for PrP^C^in the cellular uptake of extracellular vesicles**

B. Puig^a^, S. Brenna^a^, B. Mohammadi^b^, M. Glatzel^b^, H. C. Altmeppen^b^, and T. Magnus^a^

^a^Department of Neurology, Experimental Research in Stroke and Inflammation (ERSI), UKE, Hamburg, Germany; ^b^Institute of Neuropathology, University Medical Center Hamburg-Eppendorf (UKE), Hamburg, Germany

**Aims**: Extracellular vesicles (EVs) are nanoparticles released into the extracellular space by probably all types of cells and are currently considered as an important mechanism of information transfer in cell-to-cell communication. They carry and deliver a myriad of biologically active molecules critically involved in physiological but also pathological processes. While much knowledge has recently been acquired about their biogenesis, their uptake and processing by recipient cells is still not fully understood. The prion protein and its proteolytically generated C1 fragment are particularly enriched on EVs and we postulate a role for PrP in EV uptake, fusion, and cargo delivery on the recipient cell.

**Material and Methods**: EVs are directly isolated from brains of WT, Prnp-/- (Zh3/Zh3), and Tga20 mice, subsequently labeled with different dyes (mCling, R18, and ExoGlow RNA), incubated with murine primary astrocytes as recipient cells, and fixed at different time points. Employing confocal and super-resolution microscopy, the localization of the EVs presenting different amounts of surface PrP (or lacking PrP) is monitored inside the recipient cells.

**Results**: Already at 1 h after incubation with the recipient cells, EVs isolated from Prnp-/- mice are rapidly endocytosed and delivered to the late endosomal compartment (thus colocalizing with LAMP1, a marker of late endosomes/lysosomes), whereas EVs from WT mice, containing PrP and its C1 fragment, are found in close contact with the plasma membrane, but are mostly not endocytosed at that time point. However, at later time points (3 and 6 h) WT EVs also colocalize with LAMP1. Mechanistic proof-of-concept experiments are currently being performed in suitable cell lines with depletion or overexpression of PrP or C1 alone.

**Conclusions**: Our preliminary results indicate a role for PrP in affecting EV´s uptake. Although further experiments are needed and are currently being setted up, we postulate that, due to the characteristics of the C1 fragment with its hydrophobic domain being exposed towards the extracellular space, it may act as a tethering protein supporting interaction with the recipient cell`s plasma membrane in close resemblance to certain viral surface/fusion proteins. As EVs are envisioned as potential carriers for drug delivery, manipulation of PrP levels on the EV surface could be important to increase the efficiency of therapeutic cargo delivery.

**Funded by**: Hermann and Lilly Schilling Stiftung (to TM), Creutzfeldt-Jakob Disease Foundation, Inc. (to HCA)

**High transmissibility of splenic prions in cervidized transgenic mice as a diagnostic marker for CWD infection in human**

Xu Qi, Liuting Qing, Manuel Camacho, Ignazio Cali, and Qingzhong Kong

Department of Pathology, Case Western Reserve University, Cleveland, USA

**Aims**: Whether CWD prion can infect humans in real life remains controversial. Multiple in vitro CWD-seeded human PrP conversion experiments and some animal model studies indicate that the species barrier for CWD to human transmission can be overcome. One of the challenges of CWD zoonosis studies is the lack of a reliable marker for identification of acquired human CWD cases, should they occur. We aim to stablish a reliable diagnostic marker for CWD infections in humans.

**Material and Methods**: A couple of prion-positive spleens were identified from humanized transgenic mice inoculated with either CWD or sCJDMM1. Prions in these spleens were compared by bioassays in cervidized or humanized transgenic mice.

**Results**: We have detected a couple of prion-positive spleens in a group of humanized transgenic mice (Tg40h) inoculated with certain CWD isolates. Such experimentally generated splenic humanized CWD prions (termed eHuCWD^sp^) appear indistinguishable from prions in the brain of sCJDMM1 patients on Western blot. Significantly, we found that eHuCWD^sp^can efficiently infect not only the humanized Tg40h mice but also cervidized transgenic mice (Tg12). Tg12 mice infected by eHuCWD^sp^produced prions and brain pathology that are practically identical to those of CWD-infected Tg12 mice. In contrast, prions from the spleen of Tg40h mice inoculated with sCJDMM1 (termed sCJD^sp^), similar to prions from sCJDMM1 patient brains, is poorly transmissible in the Tg12 mice.

**Conclusions**: Our data demonstrate that high transmissibility with CWD features of splenic prions in cervidized transgenic mice is unique to acquired human CWD prions, and it may serve as a reliable marker to identify the first acquired human CWD cases.

**Funded by**: NIH

**Grant number**: R01NS052319, R01NS088604, R01NS109532

**Acknowledgement**: We want to thank the National Prion Disease Pathology Surveillance Center and Drs. Allen Jenny and Katherine O’Rourke for providing the sCJD samples and the CWD samples, respectively.

**Transmission properites of North American sheep scrapie prions in transgenic mouse models**

EmmaKate Raisley^a,b^, Julianna Sun^a,c^, Nick Heyer^a^, Jifeng Bian^a^, Sehun Kim^a^, Jenna Crowell^a^, Jason Bartz^d^, Tracy Nichol^e^, Terry Spraker^f^, Juergen Richt^g^ and Glenn Telling^a,c^

^a^Prion Research Center and the Department of Microbiology, Immunology, and Pathology, Colorado State University, Fort Collins, Colorado, USA; ^b^Walter Scott Jr. College of Engineering, Colorado State University, Fort Collins, Colorado, USA; ^c^Program in Cell and Molecular Biology, Colorado State University, Fort Collins, Colorado, USA; ^d^Department of Medical Microbiology and Immunology, Creighton University, Omaha, Nebraska, United States; ^e^United States Department of Agriculture, Animal Plant Health Inspection Service, Veterinary Services, Washington DC, United States; ^f^College of Veterinary Medicine and Biomedical Sciences, Veterinary Diagnostic Laboratory, Colorado State University, Fort Collins, Colorado, USA; ^g^Center of Excellence for Emerging and Zoonotic Animal Diseases, Kansas State University, Manhattan, Kansas, US

**Aims**: Validate the use of transgenic mice for modeling North American scrapie. Characterize the transmission properties of North American scrapie field isolates and their responses to variation at residue 136 of sheep PrP. Analyze the plausibility of a species crossover from sheep scrapie to manifest as chronic wasting disease in cervids.

**Material and Methods**: Four lines of transgenic mice were intracerebrally inoculated with brain homogenates from scrapie positive sheep found in Idaho and Colorado. We used our previously described lines expressing ARQ and VRQ sheep PrP variants to assess their responses to infection with North American scrapie prions. Transgenic mice expressing cervid PrP with either Q or E at residue 226 representing North American deer and elk respectively were also challenged. Brain materials from clinically diseased and asymptomatic mice were analyzed by western blotting for PrP^Sc^detection, histoblotting, and immunohistochemistry to identify infectious prions.

**Results**: Positive transmissions were produced in the ARQ and VRQ sheep Tg mice. TgARQ mice were more readily infected with field scrapie isolates compared to the TgVRQ mice for most field isolates. TgQ and TgE mouse lines had evidence of very weak transmission from the sheep scrapie inoculums.

**Conclusions**: Varying efficiency of scrapie transmission into ARQ and VRQ mice support the predicted dependence of scrapie susceptibility on the natural A/V 136 variance. The weak transmission from sheep scrapie isolates into TgE and TgQ lines may support the species crossover from sheep scrapie into cervids as chronic wasting disease.

**Funded by**: National Institutes of Health

**Grant number**: 1R01NS121682, 1R01NS109376, and PO1-0011877A

**Acknowledgement**: Many thanks to the members of the Telling Lab and the Prion Research Center. This work was supported by the NIH.

**Specific labeling of native PrPSc in RML-infected CAD5 cells using a single-chain fluobody**

Vineet Rathod^a,b^, Hailey Pineau^b,c^, Grant Norman^b,c^, Valerie Sim^b,c,d^, Holger Wille^a,b,d^

^a^Department of Biochemistry, University of Alberta, Edmonton, Alberta, Canada; ^b^Centre for Prions and Protein Folding Diseases, University of Alberta, Edmonton, Alberta, Canada; ^c^Department of Medicine, University of Alberta, Edmonton, Alberta, Canada; ^d^Neuroscience and Mental Health Institute, University of Alberta, Edmonton, Alberta, Canada.

**Aims:** The goal of this study was to engineer a recombinant, PrPSc-specific “fluobody” based on the PrPSc-specific monoclonal antibody YEG mAb Sc-G1. A fluobody is a genetically encoded antibody probe, consisting of an enhanced green fluorescent protein (eGFP) linked to a single chain variable region fragment (scFv). This PrPSc-specific fluobody proved to be a unique tool for visualizing the spatiotemporal dynamics of PrPSc in RML-infected CAD5 cells.

**Material and Methods:** We previously generated the PrPSc-specific monoclonal antibody YEG mAb Sc-G1 that recognizes native PrPSc only using a PrPSc-specific prion vaccine (*Fang et al.* unpublished data). IgG-expressing hybridoma cell clones were characterized using total RNA isolation. cDNA of the IgG2b monoclonal antibody was obtained by reverse transcription PCR and amplified using IgG-specific primers. The rodent CDR sequences were grafted into an scFv human antibody framework, whereby the antibody variable (V) region fragments were conjugated with a C-terminal eGFP domain. The PrPSc-specific fluobody was expressed in *E. coli* and purified via metal-ion affinity chromatography. The fluobody activity was tested using immunocytochemistry assays with RML-infected and uninfected CAD5 cells.

**Results:** YEG mAb Sc-G1 recognizes PrPSc under native conditions and does not react with PrPC or denatured PrP. Additionally, it reacts with all strains of PrPSc tested (12 strains). Characterization of the YEG mAb Sc-G1 epitope revealed a discontinuous epitope that recognizes PrPSc in its putative ß-solenoid configuration. Specifically, discontinuous residues H110 and D146 appear to play a pivotal role for the antigen-antibody recognition. Results from a first confocal microscopy study using the fluobody revealed widespread surface labelling of PrPSc on RML-infected cells, while uninfected control cells remained completely unlabelled. PrPSc was seen in small, diffuse punctae and larger foci, suggesting different stages of replication or aggregation. Additional studies with the fluobody in organotypic slice cultures infected with different prion strains are on-going.

**Conclusions:** scFv antibodies have the advantage of being able to penetrate deep into antigen complexes in cells and tissues due to their small size. Conjugation of the scFv probe with eGFP is a powerful strategy for imaging protein aggregates in living cells or lightly fixed tissues without the need for secondary detection systems. The PrPSc-specific fluobody promises to be a highly beneficial tool versus regular, linear epitope antibodies due to its strict specificity, selectivity, and affinity for native PrPSc. Hence, this tool may allow new insights into important physiological phenomena of prion-infections in living cells, tissues, and organisms.

**Development of a cell-based bioassay to propagate human variant Creutzfeldt-Jakob disease prions**

MLD Rayner, P Arora, A Nihat, C Schmidt, JM Ribes, P Klöhn, J Collinge and P Jat

MRC Prion Unit at UCL, London W1W 7FF, UK

**Aims**: The aim of this project is to develop a specific and highly sensitive cell-based bioassay which can detect pre-clinical levels of vCJD infection in human tissues and biofluids, particularly an assay that directly measures infectious prions. There are two main objectives: 1) develop a CAD5 cell derivative susceptible to 10^7 dilutions of vCJD infected human homogenates and 2) develop an automated scrapie cell assay for high throughout screens.

**Material and Methods**: Murine CAD5 cells were silenced for the endogenous mouse prion protein by gene editing using CRISPR /Cas9 and reconstituted with the human prion protein containing methionine at amino acid 129. These cells were challenged with vCJD inocula in scrapie cell assay (SCA), to identify a clone that was reproducibly susceptible and readily quantifiable. The optimal cell clone, was taken forward and serially single cell cloned to increase susceptibility.

**Results**: Seven rounds of single cell cloning were conducted resulting in 2–5 fold increases in susceptibility at each round. This has led us to identify a CAD5 cell clone (10E3) that is susceptible to 10^7 dilutions of vCJD infected brain homogenates. These cells are also susceptible to MV heterozygous vCJD infected brain inocula as well as multiple MM homozygous vCJD inocula. Chronically vCJD infected cell lines have also been produced from three brain homogenates to create a tool to explore the factors required for susceptibilty and screening of therapeutic. These chronically infected cells can be cured by treatment with the mouse anti-PrP antibody, ICSM-18.

The cells have also been used to devlop an automated SCA to enable high-throughput screening of human homogenates. This has the potential to be scaled up to process large numbers of samples per year, as has already been done for the mouse prion bioassay.

**Conclusions**: We have successfully produced a cell line that is susceptible to 10^7 dilutions of human vCJD brain homogenates. Amouse bioassay is underway to confirm that the vCJD prions produced in these cells are analogous to the original human innoculum.

**Funded by**: Department of Health and the MRC Prion Unit core funding.

**Grant number**: PR-R17- 0916- 23,004

**Titanium dioxide and carbon black nanoparticles disrupt neuronal homeostasis via excessive activation of PrP^c^signaling**

Luiz W. Ribeiro^a,b^, Mathéa Pietri^a,b^, Hector Ardila-Osorio^a,b^, Anne Baudry^a,b^, Chloé Bizingre^a,b^, Zaira E. Arellano-Anaya^a,b^, Anne-Marie Haeberlé^c^, Nicolas Gadot^c^, Sonja Boland^d^, Stéphanie Devineau^e^, Yannick Bailly^c^, Odile Kellermann^a,b^, Anna Bencsik^f^, and Benoit Schneider^a,b^

^a^INSERM UMR-S 1124, F-75006 Paris, France; ^b^Université Paris Cité, UMR-S 1124, F-75006 Paris, France; ^c^CNRS UPR 3212, Université de Strasbourg, Institut des Neurosciences Cellulaires et Intégratives, F-67084 Strasbourg, France; ^d^Plateforme anatomopathologie recherche, Université de Lyon, Université Claude Bernard Lyon 1, INSERM 1052, CNRS 5286, Centre Léon Bérard, Centre de recherche en cancérologie de Lyon (CRCL), Lyon, 69,373, France; ^e^Université Paris Cité, Unité de Biologie Fonctionnelle et Adaptative, CNRS UMR 8251, F-75013 Paris, France; ^f^French Agency for Food, Environmental and Occupational Health & Safety (ANSES) Université Claude Bernard Lyon 1, ANSES Laboratoire de Lyon, F-69364 Lyon, France

**Aims**: Cellular prion protein (PrP^C^) binds oligomers of unrelated misfolded proteins (PrP^Sc^, Aβ, α-synuclein), suggesting that PrP^C^recognizes protein assemblies based on a defined shape, surface properties, or steric hindrance, rather than the protein sequence of the amyloid. We aimed to assess (i) whether PrP^C^also displays the capacity to attract aggregates of nanoparticles (NPs) whose hydrodynamic diameter is comparable to that of amyloid oligomers, and (ii) whether nanoparticles cause adverse effects evocative of amyloid-based neurodegenerative diseases through PrP^C^interaction.

**Material and Methods**: To this purpose, we exploited (i) the 1C11 cell line, a neuronal stem cell endowed with the capacity to differentiate into serotonergic neurons, that was exposed to two different NPs, titanium dioxide (TiO_2_) and carbon black (CB) nanoparticles, and (ii) C57Bl/6 J mice that were intracerebrally injected with TiO_2_-NPs.

**Results**: We provide prime evidence that TiO_2_- and CB-NPs bind PrP^C^at the surface of 1C11 neuronal cells and corrupt the PrP^C^signaling function. The NPs – PrP^C^interaction triggers PrP^C^-dependent activation of NADPH oxidase and subsequent production of reactive oxygen species (ROS) that alters redox equilibrium. Through PrP^C^interaction, NPs also promote the activation of 3-phosphoinositide-dependent kinase 1 (PDK1), which in turn provokes the internalization of the neuroprotective TACE α-secretase (ADAM17). This diverts TACE cleavage activity away from (i) TNFα receptors (TNFR), whose accumulation at the plasma membrane augments the vulnerability of NP-exposed neurons to TNFα-associated inflammation, and (ii) the amyloid precursor protein APP, leading to overproduction of neurotoxic amyloid Aβ40/42 peptides. The silencing of PrP^C^or the pharmacological inhibition of PDK1 protects neurons from TiO_2_- and CB-NPs effects regarding ROS production, TNFα hypersensitivity, and Aβ rise. Finally, we show that dysregulation of the PrP^C^-PDK1-TACE pathway likely occurs in the brain of mice injected with TiO_2_-NPs by the intracerebral route as we monitor a rise of TNFR at the cell surface of several groups of neurons located in distinct brain areas.

**Conclusions**: Our study posits for the first time normal PrP^C^as being a neuronal receptor of TiO_2_- and CB-NPs and identifies PrP^C^-coupled signaling pathways by which those nanoparticles alter redox equilibrium, augment the intrinsic sensitivity of neurons to neuroinflammation, and provoke a rise of Aβ peptides. By showing that TiO_2_- and CB-NPs trigger molecular signs of Alzheimer’s disease via PrP^C^, our data shed light on how human exposure to some NPs present in the environment would contribute to the onset and/or progression of Alzheimer’s disease.

**Funded by**: INSERM and French Agence Nationale de la Recherche (ANR).

**Grant number**: ANR-14-JPCD-0003-01.

**Acknowledgement**: Imaging experiments, mRNA/protein studies, and DLS analyses were performed at the SCM, Cyto2BM, and AMS core facilities, respectively, of BioMedTech Facilities INSERM US36/CNRS UMS2009/Université Paris Cité. We acknowledge the ImagoSeine core facility of the Institut Jacques Monod. The authors thank the members of the ‘Plateforme anatomopathologie recherche, Université Claude Bernard Lyon 1, Centre Léon Bérard, Centre de recherche en cancérologie de Lyon (CRCL)’, for their excellent histotechnical assistance.

**Prion protein converts at two distinct cellular sites and precedes fibril formation**

Juan Manuel Ribes^a^, Mitali P. Patel^a^, Hazim A. Halim^a^, Sharon A. Tooze^b^, and Peter-Christian Kloehn^a^

^a^Medical Research Council Prion Unit at UCL, Institute of Prion Diseases, University College London, London W1W 7FF, United UK; ^b^Molecular Cell Biology of Autophagy Laboratory, the Francis Crick Institute, London NW1 1BF, UK

**Aims**: The cellular site of prion propagation and the kinetics of PrP conversion remain a matter of ongoing debate. A major roadblock for molecular studies of prion replication is the lack of research tools to discriminate cellular from abnormal conformers of the prion protein. We here describe a two-pronged approach to overcome this problem. We used highly discriminatory anti-PrP antibodies and conversion-tolerant PrP chimera to investigate the cellular and molecular mechanism of PrP conversion, amyloid formation and trafficking of abnormal PrP, here termed disease-associated PrP (PrP^d^).

**Material and Methods**: We use correlative imaging and prion titer output methodology to examine aggregation phenomena in freshly and persistently prion-infected neuronal cells.

**Results**: We provide evidence of phenotypically distinct PrP^d^species which differ in their cellular localization, proteolytic processing and aggregation state in neuronal cells. A full-length PrP^d^type (FL-PrP^d^) is formed exclusively at the plasma membrane and is associated with fibril formation and elongation. A truncated PrP^d^type (TR-PrP^d^) at the perinuclear region segregates into synaptic and large-dense core vesicles of the regulated exocytosis pathway to reach the plasma membrane. The infectious state of cells is dependent on functional dynamins and Cdc42, but is refractory to receptor-mediated endocytosis, suggesting that prion steady state levels are maintained by clathrin-independent endocytosis. We further show that the mechanism of *de novo* PrP conversion and formation of fibril-like PrP aggregates following prion infection is distinct in mechanistic and kinetic terms. While *de novo* PrP conversion occurs within minutes at two subcellular locations after infection, the perinuclear region and the plasma membrane, fibril-like FL-PrP^d^ aggregates at the plasma membrane are formed with a delay of about 24 hours after infection.

**Conclusions**: We present first evidence of a spatial separation between seed and amyloid-forming aggregates and their molecular link by vesicular transport. Our data suggest that inhibition of seed trafficking may lead to a cessation of amyloid formation and fibrillization. We further show that *de novo* PrP conversion leads to the formation of N-terminally truncated PrP aggregates while fibrillar full-length aggregates lag behind the fast initial PrP conversion, thus seem energetically less favorable.

**Funded by**: UK Medical Research Council (MC_UU_00024/4) and UK Biotechnology and Biological Sciences Research Council (BBSRC, BB/V001310/1); S. A. T. was supported by The Francis Crick Institute which receives its core funding from Cancer Research UK (FC001187), the UK Medical Research Council (FC001187). This research was funded in whole, or in part, by the Wellcome Trust (FC001187).

**Acknowledgement**: We thank Christian Schmidt, Parvin Ahmed and George Thirlway for technical assistance with Automated Scrapie Cell Assays.

**Clustering of human prion protein and α-synuclein oligomers requires the prion protein N-terminus**

Nadine S. Rösener^a,b^, Lothar Gremer^a,b^, Michael M. Wördehoff^a^, Tatsiana Kupreichyk^a,b^, Manuel Etzkorn^a,b^, Philipp Neudecker^a,b^, and Wolfgang Hoyer^a,b^

^a^Institut für Physikalische Biologie, Heinrich Heine University Düsseldorf, Düsseldorf, Germany; ^b^Institute of Biological Information Processing (IBI-7) and JuStruct: Jülich Center for Structural Biology, Forschungszentrum Jülich, Jülich, Germany

**Aims**: The interaction of human prion protein (huPrP) and α-synuclein oligomers (αSynO) causes synaptic impairment that might trigger Parkinson’s disease and other synucleinopathies. Here, we investigated the interaction of huPrP and αSynO. In particular, we tested for higher order heteroassociation, motivated by previous observations of large complexes of amyloid-β oligomers (AβO) and huPrP that are suggested to be involved in neurotoxic signaling.

**Material and Methods**: HuPrP-αSynO heteroassemblies were investigated by density gradient centrifugation, dynamic light scattering, atomic force microscopy, TIRF microscopy, biolayer interferometry, CD spectroscopy and NMR spectroscopy. HuPrP-αSynO heteroassembly was compared to heteroassembly of huPrP with AβO and other amyloid oligomers.

**Results**: αSynO cluster with huPrP into micron-sized condensates. Multivalency of αSyn within oligomers is required for condensation, since clustering with huPrP is not observed for monomeric αSyn. The stoichiometry of the heteroassemblies is well-defined with an αSyn:huPrP molar ratio of about 1:1. The αSynO-huPrP interaction is of high affinity, signified by slow dissociation. The huPrP region responsible for condensation of αSynO, residues 95–111 in the intrinsically disordered N-terminus, corresponds to the region required for αSynO-mediated cognitive impairment. HuPrP moreover achieves co-clustering of αSynO and Alzheimer’s disease-associated amyloid-β oligomers, providing a case of a cross-interaction of two amyloidogenic proteins through an interlinking intrinsically disordered protein region.

**Conclusions**: The multivalency of αSyn within αSynO leads to efficient condensation with huPrP, mediated by residues 95–111 in the intrinsically disordered huPrP N-terminus. The results suggest that αSynO-mediated condensation of huPrP is involved in the pathogenesis of synucleinopathies.

**Funded by**: European Research Council

**Grant number**: 726,368

**The Expression and Purification of GPI Anchored and Glycosylated PrPC for Use in Structural Studies**

Graham P. Roseman, and Stephen M. Strittmatter

Cellular Neuroscience, Neurodegeneration, Repair, Departments of Neurology and of Neuroscience, Yale University School of Medicine, New Haven, USA

**Aims**: The cellular prion protein (PrP^C^) not only causes transmissible spongiform encephalopathies (TSE’s), but also facilitates the toxic signaling cascade in Alzheimer’s disease. PrP^C^is ubiquitously expressed throughout the body, however it can be found at high concentrations in the brain. PrP^C^is found on the extracellular side of cellular membranes where it is both glycophosphatidylinositol (GPI) anchored and variably glycosylated. To understand the relationship between structure and function, PrP^C^is typically overexpressed using E.coli. This leads to PrP^C^that is devoid of posttranslational modifications as it is found in the native cellular context. The aim of this study is to express PrP^C^in a system that retains its GPI anchor and glycosylation state.

**Material and Methods**: PrP^C^is first overexpressed in suspension cell culture using Expi293F. After lysis and detergent solubilization of the membrane pellet, the sample is passed over resin covalently attached to an anti-PrP^C^ antibody. After elution, PrP^C^is mostly pure, but contains a major contaminant, the heat shock protein 60 kDa (HSP60), a previously described binding protein to PrP^C^. To remove this contaminant and to simultaneously concentrate PrP^C^without concentrating the detergent, the sample if further purified over immobilized metal affinity chromatography (IMAC) resin charged with Ni^2+^.

**Results**: After the full purification and dialysis, PrP^C^is purified to greater than 90% and is fully GPI anchored and glycosylated. With this full purification scheme, PrP^C^can be purified in milligram quantities from 1L of Expi293F cells.

**Conclusions**: PrP^C^purified in this manner is amenable to biophysical techniques that require higher concentrations of protein such as biolayer interferometry (BLI) and electron microscopy. Furthermore, PrP^C^is known to bind to several transmembrane proteins implicated in different toxic signaling cascades. These complexes can now be studied *in vitro* using this highly purified preparations of GPI anchored and glycosylated PrP^C^.

**Funded by:**

**Grant number**: R01AG034924

**Towards an improved ‘quantitative’ α-synuclein Real-Time Quaking-Induced Conversion assay to assess Lewy body pathology in vivo**

Marcello Rossi^a^, Simone Baiardi^b^, Corinne Quadalti^a^, Sofia Dellavalle^a^, Angela Mammana^a^, Sabina Capellari^a^,^c^ Kathrin Brockmann^d^, Piero Parchi^a^,^b^

^a^IRCCS, Istituto delle Scienze Neurologiche di Bologna, Bologna, Italy; ^b^Department of Experimental, Diagnostic and Specialty Medicine, University of Bologna, Bologna, Italy; ^c^Department of Biomedical and Neuromotor Sciences, University of Bologna, Bologna, Italy; ^d^Center of Neurology, Department of Neurodegeneration and Hertie-Institute for Clinical Brain Research, University of Tuebingen, Germany

**Aims**: The α-synuclein Real-Time Quaking Induced Conversion (α-syn RT-QuIC) assay is a promising diagnostic test for the Lewy body (LB) pathology. Several studies highlighted the outstanding ability of the assay to detect α-syn seeding activity with high sensitivity and specificity across the LB disease (LBD) spectrum. Although the robust ‘qualitative,’ dichotomized (positive vs. negative) output of the assay is by now consolidated, whether and to what extent RT-QuIC also provides ‘quantitative’ data remains largely unexplored. To investigate the latter informative value of the assay, we aim to compare the α-syn seeding activity in the patient groups affected, on average, by LBD pathology at different stages. They included dementia with Lewy body (DLB), mild cognitive impairment due to probable LB (MCI-LB), and other disorders in which LB manifests as co-pathology.

**Material and Methods**: We analyzed with α-syn RT-QuIC CSF samples from well-characterized patients with DLB (n = 33), MCI-LB (n = 45), Alzheimer’s disease (AD) (n = 249) and idiopathic normal pressure hydrocephalus (iNPH) (n = 127). Moreover, we had the opportunity to study a patient with an *SNCA* gene triplication and early-onset Parkinson’s disease with dementia (PDD). We run each sample in quadruplicates. We then compared the distribution of the positive α-syn RT-QuIC replicates and the values of kinetic parameters (Lag phase and Imax) of the fluorescence curves of positive replicates to investigate potential differences in the α-syn seeding activity between groups.

**Results**: Across the analyzed cohorts we observed a positive outcome in 32 (97.0%) DLB, 44 (97.8%) MCI-LB, 41 (16.5%) AD, and 28 (22.0%) iNPH patients. There were statistically significant differences in the median number of RT-QuIC positive replicates between the DLB and AD/iNPH (p < 0.001) cohorts. Moreover, DLB patients showed a statistically significant lower lag phase and a higher Imax when compared with the MCI-LB group (lag phase p = 0.003; Imax p < 0.001) and even more with AD/iNPH groups (lag phase p < 0.001; Imax p < 0.001). We detected the highest kinetic parameters in the early-onset patient carrying the *SNCA* triplication. The case was positive in all four replicates and showed a significantly shorter Lag phase than the DLB group (12.2 h vs. 15.8 h, p = 0.018).

**Conclusions**: Our work demonstrates that the α-syn RT-QuIC assay detects differences in α-syn seeding activity across the analyzed study groups, likely reflecting the amount and spread of LB pathology. Further steps are needed to fully develop a ‘quantitative’ RT-QuIC delivering reliable and reproducible quantitative data on α-syn seeding activity in individual patients.

**Funded by**: Grants from the Italian Ministry of Health (‘Ricerca corrente’)

**Standardization of Data Analysis for RT-QuIC-based detection of Chronic Wasting Disease**

Gage R. Rowden^a,b^, Catalina Picasso-Risso^b,c^, Manci Li^a,b^, Marc D. Schwabenlander^a,b^, Tiffany Wolf^b,c^,and Peter A. Larsen^a,b^

^a^Department of Veterinary and Biomedical Sciences, University of Minnesota, St. Paul, MN; ^b^Minnesota Center for Prion Research and Outreach, University of Minnesota, St. Paul, MN; ^c^Department of Veterinary Population Medicine, University of Minnesota, St. Paul, MN

**Aims**: Chronic Wasting Disease (CWD) is a transmissible spongiform encephalopathy affecting cervids caused by prions accumulating as amyloid fibrils in lymphoid and CNS tissues. Real-time quaking-induced conversion (RT-QuIC), which quantifies amyloid formation as relative fluorescent units (RFU), has rapidly become a strong candidate for CWD diagnosis. However, there is little consensus on the best approach to interpret RT-QuIC data for diagnostic purposes. Data are typically assessed by a predetermined number of replicates crossing a threshold defined as some number of standard deviations above the average RFU of the entire plate at time zero, and replicates are given a rate of amyloid formation (RAF) based on the time-to-threshold. Rather, by using a ratio based on each replicate’s independent background fluorescence, reactions can be normalized between runs and plate readers, and any fluctuations between plates can be eliminated. Given the potential use of RT-QuIC for CWD regulatory testing, we propose a more rigorous method of data analysis using a ‘maxpoint ratio’ (MPR) which is the maximum RFU obtained by a replicate during the run divided by its background RFU.

**Material and Methods**: A total of 535 white-tailed deer were sampled from CWD endemic areas in Minnesota, and individual lymph node samples were tested with RT-QuIC. All RT-QuIC data was interpreted using RAF and MPR. MPR values were plotted in histograms, and their distributions were used to determine an empirical threshold for discerning positivity. MPRs and RAFs were also compared between plate readers and substrate batches to establish any variability. Test results were compared against ELISA results when available.

**Results**: Our results reveal a distinct MPR threshold of two (i.e. twice the background RFU) for dichotomizing RT-QuIC runs. Differences in MPRs in control samples were observed between plate readers (P-value < 0.001) while none were observed between substrate batches (P-value > 0.4). RAF vs. MPR values showed little correlation (r: 0.5449, 95%CI: 0.494–0.592). MPR results showed excellent concordance (К: 0.957, 95%CI: 0.875–1.000) with ELISA results.

**Conclusions**: Our findings suggest that MPR is a viable option for future diagnostic purposes for RT-QuIC without the need for multiple comparisons analysis. Using a combination of MPR and RAF allows for a more statistically robust method for analyzing RT-QuIC data.

**Funded by**: Minnesota Department of Natural Resources; the Minnesota State Legislature through the Minnesota Legislative-Citizen Commission on Minnesota Resources (LCCMR); Minnesota Agricultural Experiment Station Rapid Agricultural Response Fund; Minnesota Agricultural, Research, Education, Extension and Technology Transfer (AGREETT) program

**Acknowledgement**: We thank F. Schendel, T. Douville, and staff of the University of Minnesota Biotechnology Resource Center for providing the large scale production of recombinant substrate necessary for RT-QuIC reactions. We thank B. Caughey, C. Orru, and A. Hughson of NIH Rocky Mountain Laboratories for providing training on RT-QuIC methods and for providing the recombinant PrP clone. We thank the Minnesota Department of Natural Resources, especially M. Carstensen, L. Cornicelli, E. Hildebrand, P. Hagen, and K. LaSharr, for providing the tissues used in our analyses. K. Wilson of the Colorado State University Veterinary Diagnostic Laboratory conducted ELISA and IHC testing of samples reported herein. S. Stone managed laboratory logistics and finance. This study was executed under the Minnesota Center for Prion Research and Outreach (MNPRO) at the University of Minnesota.

**AMYSEEDS: Targeting amyloid beta seeds at the initial stage of Alzheimer’s disease**

Alejandro Ruiz Riquelme

University of Santiago de Compostela, Santiago de Compostela, Spain

**Aims**: This project builds on recent findings that pathogenic Aβ seeds might be present in AD patients before the start of Aβ-associated pathology (early seeds). Those seeds conformation and/or biochemical nature might be different from Aβ seeds isolated from brains of late-stage AD patients. Thus, this project intends to isolate and characterize these early Aβ seeds from human brain tissue and, based on their structural features, find new therapeutic agents with potential to delay AD when applied early enough.

Aim 1 – Identification and isolation of early Aβ seeds from human brain tissue.

Aim 2 – Characterization of isolated early Aβ seeds.

Aim 3 – Screening of structure-based therapeutic compounds.

**Material and Methods**:

- Identification of healthy human brains containing Aβ seeds. Brain samples from patients with no AD diagnostic will be assessed for the presence of Aβ seeds using a recently developed cellular assay.
- Isolation of early Aβ seeds. A phase transition-based method will be used to isolate and concentrate Aβ assemblies under native conditions from human tissue.
- Biochemical and structural analysis. The composition and aggregation state of the Aβ seeds will be assessed by different methods (e.g., mass spectrometry) and the structure by cryo-electron microcopy.
- Virtual and high-throughput screening (HTS). Based on the structural and conformational features of the Aβ seeds, a variety of compounds will be selected, and Aβ seeds/compounds interaction will be assessed by HTS.
- *In vitro* and *in vivo* validation. The capacity of the selected compounds to block the seeding capacity of the Aβ seeds will be *in vitro* (cellular assay) and *in vivo* (APP Tg mice) tested.

**Results**: To date, the presence of early Aβ seeds in human brains before the start of deposition has not been confirmed. This project will probe their existence for the first time. The identification and validation of structure-based compounds against early Aβ seeds will establish the grounds for a future AD therapy. Remarkably, this same approach of blocking the early seeds could be adapted to other proteins involved in neurodegenerative diseases such as synuclein and tau, which also form pathogenic protein assemblies that propagate in a prion-like fashion.

**Conclusions**: The successful completion of the AMYSEEDS project will then suppose a great step forward for the understanding of the start of AD and hopefully will lead to the discovery of a disease-modifying therapy.

**Funded by**: Alzheimer Forschung Initiative e.V.

**Grant number**: #19079p

**Acknowledgement**: Biobank IIS Galicia Sur, Mathias Jucker (Hertie Institute for Clinical Brain Research, Germany), Jesús R. Requena (University of Santiago de Compostela, Spain), Stephen Strittmatter (Yale School of Medicine, USA).

**Subclinical infection in sheep exposed to low doses of prions by blood transfusion.**

MKF Salamat^a^, P Stewart^a^, H Brown^a^, KBC Tan^a^, A Smith^a^, C de Wolf^a^, AR Alejo Blanco^a^, M Turner^b^, JC Manson^a^, S McCutcheon^a^, and EF Houston^a^

^a^The Roslin Institute, Royal (Dick) School of Veterinary Studies, University of Edinburgh, Easter Bush, Midlothian, Edinburgh, UK; ^b^Scottish National Blood Transfusion Service (SNBTS), The Jack Copland Centre, Edinburgh, UK

**Aims**: Although the incidence of confirmed cases of infectious prion diseases such as bovine spongiform encephalopathy (BSE), and the related variant Creutzfeldt-Jakob disease, has declined to very low levels, there is limited evidence that long term infection of some individuals may occur without causing disease symptoms (subclinical infection). The aim of this study was to look for evidence of subclinical prion infection in sheep following exposure to low titres of BSE by blood transfusion, a clinically relevant route. These sheep, many of which survived for significant periods (for more than 10 years) after transfusion^1,4^, did not show any clinical signs of disease and standard post mortem tests of immunohistochemistry and Western blot were unable to detect PrP^Sc^.

**Material and Methods**: We applied microplate-based, miniaturized bead serial PMCA (mb-PMCA)^2–4^, a highly sensitive PrP^Sc^detection assay to lymph nodes from a large cohort of sheep 9 (n = 61) which had been transfused with blood components from BSE-infected donors, and a number of secondary recipients transfused with whole blood from primary recipients (n = 10). Brain homogenate from TgShpXI transgenic mice overexpressing sheep ARQ PrP was used as substrate. Negative control samples came from mock-infected donors (n = 5) and recipients (n = 5).

**Results**: We found that in over half of primary recipients tested, PrP^Sc^was detected in a peripheral (subcutaneous) lymph node by mb-PMCA. Titration experiments revealed the levels of PrP^Sc^in lymphoid and brain samples from these sheep were approximately a million-fold lower than in clinically affected sheep, and no PrP^Sc^was detected in blood samples. Tissues from a small number of secondary recipients transfused with blood from PMCA positive sheep also tested negative by mb-PMCA.

**Conclusions**: Our results demonstrate for the first time that subclinical infection is a frequent outcome following exposure to low prion doses by clinically relevant route, and can persist until close to the natural lifespan of sheep without progressing to clinical disease. Individuals with subclinical prion infection are difficult to detect, but could potentially transmit infection and/or allow persistence and evolution of prions leading to future disease outbreaks, with obvious implications for disease control.

^a^McCutcheon, S. et al. (2011) PLoS one 6, e23169.

^b^Moudjou et al. (2013) MBio. 31;5(1):e00829-13.

^c^Lacroux et al. (2014) PLoS Pathog 10:e1004202.

^4^Salamat el al. (2021) PLoS Pathog 17:e1009276.

**Funded by**: The study was funded by the Policy Research Programme of the Department of Health and Social Care (‘The Effect of Leucodepletion on Transmission of BSE by Transfusion of Sheep Blood Components’)

**Grant number**: 007/0162

**Acknowledgement**: The authors thank past and present members of large animal research services at the Institute for Animal Health, Compton and the Roslin Institute for excellent care of the sheep and technical assistance with experimental procedures. We are grateful for the generous provision of TgShpXI transgenic mouse brains for use as PMCA substrate by Olivier Andreoletti (INRAE-ENVT, Toulouse, France).

**Revisiting phylogeny within the class Mammalia using the prion protein sequence from hundreds of species**

Cristina Sampedro-Torres-Quevedo^a^, Hasier Eraña^a,b,c^, Jorge M. Charco^a,b,c^, Carlos M. Díaz-Domínguez^a^, Leire FernándezVeiga^a^, Juan Tasis-Galarza^a^, Ana R. Cortazar^a^, Roberto F. Nespolo^d,e,f^, Julian F. Quintero-Galvis^d^, Africa Manero-Azua^g^, Diego Polanco-Alonso^g^, Guiomar Perez de Nanclares^g^, Ana M. Aransay^a^ and Joaquín Castilla^a,c,h^

^a^Center for Cooperative Research in Biosciences (CIC bioGUNE), Basque Research and Technology Alliance (BRTA), Bizkaia Technology Park, Derio, Spain; ^b^ATLAS Molecular Pharma S. L. Bizkaia Technology Park, 800, Derio, Spain; ^c^Centro de Investigación Biomédica en Red de Enfermedades Infecciosas (CIBERINFEC), Carlos III National Health Institute, Madrid, Spain; ^d^Instituto de Ciencias Ambientales y Evolutivas, Universidad Austral de Chile, Valdivia. Chile; ^e^Center of Applied Ecology and Sustainability (CAPES), Facultad de Ciencias Biológicas, Universidad Católica de Chile, Santiago, Chile; ^f^Millenium Institute for Integrative Biology (iBio), Santiago, Chile; ^g^Molecular (Epi)Genetics Laboratory, Bioaraba Health Research Institute, Araba University Hospital, Vitoria-Gasteiz, Araba, Spain; ^h^IKERBASQUE, Basque Foundation for Science, Prion Research Lab, Bilbao, Spain

**Aims**: To generate a phylogenetic tree using the PrP sequence of what is to our knowledge the largest collection of PrP sequences from mammalian origin and compare our results to the classic phylogeny of species.

**Material and Methods**: Recombinant protein sequences from 655 different mammalian species were aligned using CLUSTALW. After the alignment, identical sequences were merged in order to reduce background noise, leaving 323 different sequences. Trees were generated using the Bayesian Evolutionary by Sampling Trees package (BEAST) using the best fit model identified by MEGA X. Analysis were run for 10,000,000 generations, sampling every 5,000 chains under a relaxed log normal clock model and Yule model. Three independent runs were combined in LogCombiner. Posterior result analysis was done grouping species by orders.

**Results**: There is a strong tendency of the classical phylogenetic orders to maintain their clusterization when using the *PRNP* gene as a readout of phylogenetic similarity.

**Conclusions**: Despite the fact that only one gene was used to generate the phylogenetic tree, leading to an increase in uncertainty of the analysis performed by the bioinformatics tools, the general topology of the phylogenetic tree was considerably well conserved compared to the canonical mammalian phylogenetic tree. It is possible this is due to the high number of sequences that have been included in the analysis to generate the alignment and subsequent phylogenetic tree.

Moreover, looking at the resulting tree it may be possible to identify clusters of ‘prion resistance/susceptibility’ focusing on the species included in said clusters and the tendency of those species’ PrP to misfold *in vivo* or *in vitro*, i.e. swine, suggested to be more resistant are separated from other susceptible artiodactyls (sheep, cattle, camels).

**Funded by**: Spanish Ministry of Science and Innovation

**Grant number**: PID2021-122201OB-C21

***In utero* transmission of chronic wasting disease in free-ranging white-tailed deer**

Audrey M. Sandoval^a^, Amy V. Nalls^a^, Erin E. McNulty^a^, Nathaniel D. Denkers^a^, Zoe Olmstead^a^, Ethan Barton^b^, James M. Crumc, Mark G. Ruder^b^, and Candace K. Mathiasona ^a^

^a^Department of Microbiology, Immunology, and Pathology, College of Veterinary Medicine and Biomedical Sciences, Colorado State University, Fort Collins, Colorado, USA; ^b^Southeastern Cooperation Wildlife Disease Study (SCWDS), University of Georgia, Athens, Georgia; ^c^Wildlife Resources, West Virginia Division of Natural Resources, Elkins, WV, USA

**Aims**: Chronic Wasting Disease (CWD) is a prion disease affecting cervid species in North America, Korea, and Scandinavia. Historically, CWD has been attributed to horizontal transmission i.e., direct contact between cervids, or indirect contact with infectivity present in bodily secretions shed into the environment. However, previous studies in captive Reeve’s muntjac deer and free-ranging elk have demonstrated the involvement of vertical transmission, transmission from mother-to-baby during pregnancy, as another likely source of infection. In this multi-state study, we were interested to determine the potential role of *in utero* or vertical transmission, in free-ranging naturally exposed white-tailed deer. CWD prion seeding activity was assessed in tissues and fluids harvested from the pregnancy microenvironment of white-tailed dams from Arkansas, Tennessee and West Virginia by conventional and prion amplification detection assays.

**Material and Methods**: Maternal and *in utero-*derived fetal tissues (n = 56) were harvested from healthy appearing dams (n = 31) in three states with known CWD+ status (Arkansas, Tennessee, West Virginia) and one state with no known CWD infections (Georgia). Maternal tissues were assessed for amyloid seeding activity by real time quaking induced conversion (RT-QuIC) in addition to immunohistochemistry (IHC). Amniotic fluid was assessed with the additional step of iron- oxide magnetic extraction, while fetal tissues were subject to protein misfolding cyclic amplification (PMCA) before detection with RT-QuIC and Western blotting.

**Results**: Prion seeding activity was detected in retropharyngeal lymph node, tonsil, recto-anal mucosal associated lymphatic tissue (RAMALT) and/or obex of 18/28 pregnant does. Further testing of maternal uterus (n = 29), placentomes (n = 200), or amniotic fluid (n = 50) revealed detection in 8 of the 18 CWD+ dams. Detection was not found in the negative dam controls from Georgia (n = 0/3).

**Conclusions**: Our findings indicate the presence of CWD prions in maternal tissues and fluids of the reproductive tract, suggesting the likelihood of fetal exposure prior to parturition. Ongoing testing in fetal tissues as well as mouse bioassays on both maternal and fetal tissues will further shed light on the presence of infectivity, and determine the role of vertical transmission in the efficient transmission of CWD.

**Funded by**: Multistate Conservation Grant Program, The Association of Fish and Wildlife Agencies

**Grant number**: F20AP00172

**Quantitative 14-3-3 protein and prion RT-QuIC concordance analysis of patients with suspected prion diseases in Spain**

Jordi Sarto^a^, Laura Naranjo^b^, Carlos Nos^c^, Raquel Ruíz-García^b^, and Raquel Sánchez-Valle^a^

^a^Alzheimer’s Disease and Other Cognitive Disorders Unit, Neurology Service, Hospital Clínic de Barcelona, Institut d’Investigacions Biomèdiques August Pi i Sunyer (IDIBAPS), Universitat de Barcelona, Barcelona, Spain; ^b^Immunology Service, Biomedical Diagnostic Center, Hospital Clínic de Barcelona, Barcelona, Spain; ^c^General Subdirectorate of Surveillance and Response to Emergencies in Public Health, Department of Public Health in Catalonia, Barcelona, Spain

**Aims**: Prion diseases are rare fatal diseases typically characterized by a rapid cognitive decline in addition to other neurological signs and symptoms. Updated diagnostic criteria include the prion (PrPsc) identification by Real-Time Quaking-Induced Conversion (RT-QuIC) as an additional biomarker. Recently, validation studies of a commercially available 14-3-3 ELISA showed similar diagnosis validity to the to the established Western blot method (Schmitz, 2016). In this study, we aim to study the concordance of quantitative 14-3-3 and RT-QuIC results in CSF samples of patients with suspected prion diseases.

**Material and Methods**: Retrospective analysis of all CSF samples from patients with suspected prion disease received in the Immunology Service of the Hospital Clínic de Barcelona for 14-3-3 and RT-QuIC determination during 4 years (2018–2021). 14-3-3 was determined by enzyme-linked immunosorbent assay (ELISA). 14-3-3 levels higher than 20.000 AU/mL were considered positive for Creutzfeldt-Jakob diagnosis. RT-QuIC assay for PrPsc detection was performed according to the established methodology (McKenzie, 2022) and the result was reported as positive or negative.

**Results**: 351 samples were received during the study period for 14-3-3 and RT-QuIC determination. Two hundred thirty-seven (68%) tested negative for both 14-3-3 and RT-QuIC (-/-), 50 (14%) tested positive for both biomarkers (+/+), and 64 showed conflicting results: 45 (13%) had a positive 14-3-3 results and negative RT-QuIC result (±) and 19 (5%) had a negative 14-3-3 and positive RT-QuIC (-/+). Mean (standard deviation) 14-3-3 levels were 6,405 (4,238), 56,669 (23,063), 50,011 (24,866) and 12,238 (4,799) for the -/-, +/+, ± and -/+ groups, respectively. 14-3-3 levels were lower in the -/- group compared to -/+ patients, p < 0.001. 14-3-3 levels were also lower when RT-QuIC was negative (mean 13,364 [SD 19,179] vs 44,434 [28,091]), p < 0.001. CSF 14-3-3 levels had a diagnostic accuracy to predict the RT-QuIC result, as assessed by the area under the receiver operating characteristic curve (AUC), of 0.87 (95% confidence interval: 0.83–0.91). The 20.000 AU/mL cut-off had 73% sensitivity, 84% specificity and an accuracy of 82% to classify the RT-QuIC results. Out of 52 participants also referred for *PRNP* gene analysis, six carried a pathogenic mutation: 2 D178N, 1 E200K and 1 P102L were -/-, 1 E200K was -/+ and 1 E200K was +/+.

**Conclusions**: Using the established 14-3-3 levels cut-off, 82% of patients presented concordant 14-3-3 and RT-QuIC results. 14-3-3 levels in patients with both tests negative were lower than in patients 14-3-3 negative but with positive RT-QuIC.

**Funded by**: Dr Josep Baselga funds for research in prion diseases.

**Targeting sodium-potassium pumps for the treatment of prion diseases**

Gerold Schmitt-Ulms^a^, Shehab Eid^a^, Declan Williams^a^, Mohadeseh Mehrabian^a^, Thomas Zerbes^a^, Xinxhu Wang^a^, Hamza Arshad^b^, Joel Watts^b^, Wenda Zhao^a^, and Chris Sackmann^a^

^a^Department of Laboratory Medicine and Pathobiology, University of Toronto, Toronto, Canada; ^b^Department of Biochemistry, University of Toronto, Toronto, Canada

**Aims**: A wide range of observations in humans and animals indicate that a reduction in steady-state levels of the cellular prion protein (PrP^C^) is both safe and may extend survival of prion diseases. We recently discovered that PrP^C^binds to sodium-potassium ATPases (NKAs), which led us to hypothesize that targeting NKAs with their natural inhibitors, cardiac glycosides (CGs), may cause cells to internalize and degrade NKAs and PrP^C^. We tested this hypothesis and sought to identify a novel CG that exhibits lower toxicity and improved blood brain barrier (BBB) penetrance, relative to oleandrin, widely considered the best CG for brain applications.

**Material and Methods**: Atomic structures of NKAs helped us predict the binding poses of candidate CGs within human NKAs. A subsequent *in silico* screen identified a small number of CGs expected to exhibit favorably characteristics for semi-synthesis, NKA binding, and BBB penetrance. Next, we undertook extensive toxicological, pharmacological and biochemical comparisons of oleandrin and a CG, termed KDC203, that was shortlisted based on its predicted features.

**Results**: We will present data, which establish that CG exposure of human neural cell models causes the anticipated reductions in steady-state levels of PrP^C^. Moreover, we have validated that low nanomolar concentrations of KDC203 reduce PrP^C^levels by 85% in cultured human neural cell lines, thereby establishing this compound to be 10- to 1000-fold more potent than other small-molecule compounds reported to date for this application. KDC203 also possesses promising pharmacological characteristics and tenfold lower toxicity than oleandrin. Consistent with these data, KDC203 reached twofold higher brain concentrations than oleandrin in pilot *in vivo* work, making it a promising compound for further pre-clinical evaluations.

**Conclusions**: This work identified a novel modality for the treatment of prion diseases that makes use of low nanomolar concentrations of a small molecule from a compound class that is pharmacologically well-understood and exhibits excellent *in vitro* potency for reducing PrP^C^levels.

**Funded by**: This work received generous grant support from the Krembil Foundation.

**Grant number**: Krembil Foundation fund 507,571

**Acknowledgement**: We gratefully acknowledge philanthropic support by the Irwin family.

**Investigate the genetic and molecular landscape of the hnRNP K cellular essentiality by performing unbiased CRISPR screens**

Stefano Sellitto, Davide Caredio, Lukas Frick, Emina Lames, Dalila L. Vena, Sandesh Neupane, and Adriano Aguzzi

Institute of Neuropathology, University of Zurich, CH-8091Zurich, Switzerland

**Aims**: Recently, our lab described a role of the heterogenous nuclear ribonucleoprotein K (hnRNP K) in prion diseases. hnRNP K is an essential gene encoding an RNA Binding Protein and it was identified as a target in several malignancies. More recently, hnRNP K also emerged as an interesting protein in neurophysiology and neuropathology.

A better understanding of these functions, with particular focus on prion diseases, will be of remarkable importance. In addition, a deeper characterization of the two faces of hnRNP K in cancer and neuropathology could be seminal to elucidate shared mechanisms of genetic and molecular abnormalities.

According to this, genome-wide screens offer a great opportunity to dissect the different cellular roles of hnRNP K by identifying synthetic genetic interactions and functional essential domains of this protein.

**Material and Methods**: The project is organized in two experimental screens: 1) a whole genome screen to identify genes whose perturbation suppresses the low cellular fitness induced by the hnRNP K loss-of-function; 2) a domain-dependency screen to assess the functional essentiality of single hnRNP K’s domains. For the first goal, we used the Brunello library to perform a whole genome pooled CRISPR knockout screen. Two human glioblastoma cell lines (LN-229 and U251-MG), knockout for the hnRNP K gene and expressing the Cas9 endonuclease, were used as cellular models.

For the second screen, we combined the exogenous expression of seven different hnRNP K’s delated variants with a set of intronic sgRNAs to knock out only the endogenous hnRNP K gene in Cas9-expressing LN-229 and U-251 MG cell lines.

**Results**: In the first screen, we identified 763 and 37 significantly enriched and depleted genes respectively. The enriched genes were selected based on the FDR, the fold change, the number of guides enriched and the cellular fitness resulting from their individual knockout. The hits were further clustered for functional interactions using the STRING database and a final list of 41 candidates was generated.

In the second screen, we identified 3 indispensable and 4 dispensable hnRNPK’s domains in the LN-229 and U-251 MG cell lines.

**Conclusions**: Investigating the genetic and molecular landscape of hnRNP K essentiality in an unbiased and comprehensive way will highlight the mechanistical underpinnings of its cellular indispensability. Moreover, the biological understanding of how hnRNP K exerts its fundamental role inside the cell may provide insights into the complex biology of this protein and it could potentially track unknown pathways at the intersections between cancer, neurodevelopment and neurodegeneration.

**Acknowledgement**: Daniel Heinzer, Merve Avar, Kathi Gin

**Stabilization of monomeric α-synuclein by all-D-enantiomeric peptide ligands as therapeutic strategy for Parkinson’s disease and other synucleinopathies**

Marc Sevenich^a,c^, Ian Gering^a^, Madita Vollmer^a^, Selma Aghabashlou Saisan^a,b^, Markus Tusche1, Tatsiana Kupreichyk^a,b^, Thomas Pauly^b^, Matthias Stoldt^a^, Wolfgang Hoyer^a,b^, Antje Willuweit^c,d^, Janine Kutzsche^a^, Nils-Alexander Lakomek^a,b^, Luitgard Nagel-Steger^b^, Lothar Gremer^a,b^, Gültekin Tamgüney^a,b^, Jeannine Mohrlüder^a^ and Dieter Willbold^a,b,e^

^a^Institute of Biological Information Processing (IBI-7), Forschungszentrum Jülich, Jülich, Germany; ^b^Institut für Physikalische Biologie, Heinrich-Heine-Universitêt Düsseldorf, Düsseldorf, Germany; ^c^Priavoid GmbH, Düsseldorf, Germany; ^d^Institute of Neuroscience and Medicine (INM-4), Forschungszentrum Jülich, Jülich, Germany; ^e^JuStruct, Forschungszentrum Jülich, Jülich, Germany

**Aims**: Parkinson’s disease (PD) is the most common neurodegenerative movement disorder worldwide. One of its central features is the neurodegeneration that starts in the substantia nigra and progressively tends to involve other brain regions. α-synuclein (α-syn) and its aggregation during pathogenesis have been drawn into the center of attention, where especially soluble oligomeric structures are thought to play a key role in prion-like cell-to-cell transmission and induction of toxic effects. Here, we report the development of all-D-enantiomeric peptide ligands that bind monomeric α-syn with high affinity, thereby stabilizing the physiological intrinsically disordered structure and preventing initiation of aggregation as well as eliminating already existing aggregates.

**Material and Methods**: Mirror-image phage display, next generation sequencing (NGS), surface plasmon resonance (SPR), microscale thermophoresis (MST), atomic forced microscopy (AFM), dynamic light scattering (DLS), size exclusion chromatography (SEC), paramagnetic relaxation enhancement (PRE), nuclear magnetic resonance spectroscopy (NMR), Thioflavin-T (ThT) aggregation assay, cell-viability assay (MTT-assay).

**Results**: Based on mirror-image phage display on the D-enantiomeric full-length α syn target, we identified two lead compounds, SVD-1 and SVD-1a, by NGS, ThT screens and rational design. The compounds were analyzed with regard to their anti-aggregation potential and both compounds showed aggregation delaying as well as seed capacity reducing effects in *de novo* and seeded environments, respectively. By SPR and MST, a high affinity for the monomeric α-syn was found in the nano- to picomolar K_D_ range. 2D-^a^H-^15^N NMR of isotope labeled α-syn monomer and SVD-1a revealed shift changes in ppb scale in presence of SVD-1a for most of the side resonances with an emphasis on the C-terminal part. This was confirmed by PRE-NMR measurement with paramagnetic spin labeled SVD-1a, where again, interaction over the whole α-syn monomer with an emphasis on the C-terminus was identified.

Finally, SVD-1a reduced toxic effects as well as intracellular seeding capacity of preformed fibril (PFF) oligomers in cell culture and was able to specifically disassemble α-syn PFF oligomers into monomers as identified by AFM, time dependent DLS and SEC analysis.

**Conclusions**: The present work provides promising results for the development of lead compounds with an anti-prionic mode of action for the future treatment of Parkinson’s disease and other synucleinopathies. It also gives insights into how to characterize the interaction of a therapeutic peptide with an intrinsically disordered target protein, providing the basis for future optimizations.

**Funded by**: Michael J. Fox Research Foundation

**Grant number**: MJFF-000934

**Compilation of Research on Prion therapeutics**

Niti Sharma, Kyu Hwan Shim, and Seong Soo A. An

Department of Bionano Technology, Gachon University, Sujeong-Gu, Seongnam, South Korea

**Prion diseases** are a group of incurable neurodegenerative diseases caused by the prions, affecting both humans and animals. The atypical folding and aggregation of the soluble cellular prion proteins (PrP^C^) into scrapie isoform (PrP^Sc^) in the CNS, results in brain damage and other symptoms associated with it. Different therapeutic approaches ranging from organic compounds to antibodies have been proposed, including stalling PrP^C^to PrP^Sc^conversion, increasing PrP^Sc^removal, and/or PrP^C^stabilization using different research methodologies like cell-therapy, immunotherapy, pharmacotherapy and compounds ranging from chemicals to proteins have been studied to target the disease with special attention to PrP^Sc^aggregation inhibition. Compounds destabilizing PrP^Sc^and reducing infection have also been identified. A few important **chemical compounds** (Sulfated polyanions, diazo dyes, Phenothiazine derivative, Cyclic Tetrapyrroles, Diphenylmethane derivatives, Diphenylpyrazole derivatives, Indole-3-Glyoxylamides, 2-Aminothiazoles, Carbazole derivatives, Benzoxazole derivative, Ethanolamine, Dimethyl sulfoxide); **repurposed drugs** (Quinacrine, *Chlorpromazine*, Celecoxib, Flupirtine, Imatinib, Efavirenz, Simvastatin, Glimepiride, Doxycycline); **natural products** (Polydatin, Curcumin, Resveratrol, Epigallocatechin gallate, Cannabidiol, Baicalein, Hinokitiol, Ginsenoside, Bile acids) and **anti-prion antibodies** (6H4, D13, D18, 8B4, 8H4, ICSM18, ICSM35, POM1-2, 4H11, 44B1). All these compounds have displayed anti-prion activity *in vitro*, but only a few were effective *in vivo*. Recently, encouraging results of a **prion protein monoclonal antibody** (an IgG_4_κ isotype; **PNR100**) in a clinical trial study on CJD patients have been announced which stabilized PrP^C^and increased the survival in infected mice. After such promising results, PRN100 will be evaluated for Phase-II trials. The success story doesn’t end here as another promising molecule MC and GN8 are ready for human clinical trials on prion-diseases while Anle138b is in Phase 1b for PD patients. The ineffectiveness of most of the molecules tested was either due to inability to cross BBB, toxicity or transitory accumulation of drug resistant prions. Hence, it is incredibly important to learn from the backstory. By understanding what all has already been done, what is the mechanism of drug action, why the clinical trials failed, we can comprehend prion diseases better and chalk out the right direction leading to more effective treatment. Also, targeting more than one pathway involved in prion diseases may provide synergistic benefits.

**Funded by**: This research was funded by the National Research Foundation of Korea and by the Korean Government (2020R1A2B5B01002463 and 2021R1A6A1A03038996).

**Characterization of a novel prion protein mutation of serine to proline at residue 245 linked to VPSPr-like phenotype *in vivo* and *in vitro***

Pingping Shen^a,b^, Johnny Dang^a^, Mark Cohen^a,c^, Jue Yuan^a^, Zerui Wang^a^, Yue Lang^a,b^, Tricia Gilliland^a^, Jessica Ludwig^c^, Maria Gerasimenko^a^, Michelle Tang^a^, Sarada Rajamanickam^a^, Anika Yadati^a^, Jiri Safar^a,d^, Lawrence B. Schonberger^e^, Shulin Zhang^a^, Brian S. Appleby^a,c,d^, Robert B. Petersen^a,f^,and Wen-Quan Zou^a,c,d^

^a^Department of Pathology, Case Western Reserve University School of Medicine, 2085 Adelbert Road, Cleveland, OH, USA;^b^Department of Neurology, First Hospital of Jilin University, Changchun, Jilin Province, China; ^c^National Prion Disease Pathology Surveillance Center, Case Western Reserve University, Cleveland, OH, USA; ^d^Department of Neurology, Case Western Reserve University School of Medicine, Cleveland, OH, USA; ^e^Division of High-Consequence Pathogens and Pathology, Centers for Disease Control and Prevention, 1600 Clifton Rd, Atlanta, GA, USA; ^f^Foundation Sciences, Central Michigan University College of Medicine, Mount Pleasant, MI, USA

**Aims**: More than 50 deleterious mutations in the prion protein (PrP) have been linked to genetic human prion diseases to date, including Creutzfeldt-Jakob disease (CJD), Gerstmann-Sträussler-Scheinker (GSS) disease. All but two of the mutations localize within the mature PrP between residues 23 and 231. The aim of this study is to characterize the pathological PrP (PrP^Sc^) in a case with prion disease linked to a novel PrP mutation and the effect of this mutation on PrP trafficking, misfolding and aggregation in a cell model.

**Material and Methods**: Autopsy brain tissues from a cadaver with an atypical prion disease carrying a novel mutation of serine (S) to proline (P) in the C-terminal signal peptide region of PrP at residue 245 (PrP^S245P^) were examined by western blotting and neurohistology. As controls, autopsy brain tissues from cases with GSS, sporadic sCJD, variably protease-sensitive prionopathy (VPSPr), and a normal subject were also examined, respectively. Human neuroblastoma cells (M17) were transfected with either human wild-type PrP or PrP^S245P^and the transfected cells were characterized for comparison of the mutant and wild-type PrP.

**Results**: Through US national prion disease surveillance, we identified a clinically atypical prion disease that had a novel mutation of serine (S) to proline (P) in the C-terminal signal peptide region of PrP at residue 245 (PrP^S245P^). Neuropathologically, spongiform degeneration and PrP^Sc^staining were observed in frontal, temporal and occipital cortex as well as basal ganglia and thalamus. Biochemically, this mutation exhibited the electrophoretic gel profile of proteinase K (PK)-resistant pathogenic PrP (PrP^Sc^), reminiscent of that seen in VPSPr, a sporadic human prion disease with glycoform-selective PrP^Sc^. PrP^Sc^was observed to form multiple ladder-like protein bands after treatment with PK. These PrP bands exhibited a higher affinity for the 1E4 antibody and lower affinity for the 3F4 antibody. As PrP^Sc^in VPSPr, two sets of N- and C-terminally truncated PrP^res^were identified by 1E4 and Anti-C antibodies. PrP^S245P^and wild-type PrP in the transfected M17 cells were compared for trafficking, misfolding, and aggregation of PrP.

**Conclusions**: Our study revealed that the novel PrP^S245P^mutation confers atypical clinical and pathological changes and a PrP^Sc^gel profile similar to that seen in VPSPr, suggesting that it represents the second genetic form of VPSPr, following the first genetic VPSPr with PrP^V180I^mutation.

**Funded by**: CJD Foundation and NIH.

**Multi-centric plaques in kuru: a fingerprint of its origin**

Beata Sikorska, and Pawel P. Liberski

Department of Molecular Pathology and Neuropathology, Medical University of Lodz, Lodz, Poland

**Aims**: Kuru, brought to medical attention by D. Carleton Gajdusek and Vincent Zigas, was the first human prion disease proved to be transmissible. Spread by ritual endocannibalism among the Fore linguistic group of Papua New Guinea, kuru was a uniformly fatal cerebellar ataxia syndrome. The most striking neuropathological feature of kuru was the presence of numerous amyloid plaques; hence, the name ‘kuru plaques’ was coined. Here, we present a detailed study of amyloid plaques in archived brain from a well-documented kuru case, using electron microscopy, immunohistochemistry and confocal laser microscopy.

**Material and Methods**: We employed modern neuropathological techniques to re-evaluate archival formalin-fixed, paraffin-embedded brain specimens, including cerebellum, several cortical areas, hippocampus, and basal ganglia, of a 16-year-old male who died of kuru. Immunohistochemical stainings with antibodies against PrP, βAPP, pTau, alpha-synuclein, TDP43 and p62 were performed. Double and triple immunofluorescent stainings were used for confocal laser microscopy. 3D image analysis software with volume rendering was used to study the structure of the amyloid plaques, their spatial organization and association with cells.

**Results**: The immunohistochemical reaction against PrP^Sc^revealed diffuse synaptic deposits in all areas examined. The typical uni-centric kuru plaques were numerous in the granular layer of the cerebellar cortex but were also present in the striatum and hippocampal formation. Apart from these classic changes, we observed a few florid plaques similar to those described in vCJD and most interestingly, multi-centric plaques unique to GSS. The multi-centric plaques, reconstructed into 3D-structural images, using confocal laser microscopy, showed several neurites crossing the amyloid plaques. The 3D images confirmed structural similarity of the multicentric plaques of kuru to those observed in GSS.

**Conclusions**: The multi-centric neuritic plaques found in the hippocampus of this kuru-affected brain are typical only of GSS, and to our knowledge have never been reported in any other disease. Taking into consideration that iatrogenic cases of prion disease and vCJD cases transmitted by blood transfusion showed that prion transmission from human to human does not significantly change the neuropathological and biochemical phenotype and that the unique type of plaques was observed only in GSS and kuru, it may be hypothesized that kuru originated from a GSS case cannibalized incidentally, resulting in subsequent epidemics.

**Funded by**: Statutory funds of Medical University of Lodz

***In silico* study of drugs docking against cellular, mutated and scrapie forms of prion protein**

Christopher Situ^a^, Lyudmyla Dorosh^b^, Sara Amidian^c^, Maria Stepanova^b^, and Holger Wille^c^

^a^Old Scona Academic, Edmonton, Canada; ^b^Electrical & Computer Engineering Department, University of Alberta, Edmonton, Canada; ^c^Centre for Prions and Protein Folding Diseases, University of Alberta, Edmonton, Canada; Department of Biochemistry, University of Alberta, Edmonton, Canada

**Aims**: Conversion of the prion protein from the cellular form (PrP^C^) to the infectious scrapie prion (PrP^Sc^) and further aggregation of misfolded isoforms is believed to cause transmissible spongiform encephalopathies (TSEs), a group of fatal neurodegenerative conditions that is currently lacking effective treatment options. Finding chemotherapeutic agents capable of inhibiting the conversion of PrP^C^into PrP^Sc^without interfering with the normal functions of the protein, as well as identifying drugs capable of preventing further conversion and aggregation are critical for the development of disease-modifying and preventive treatments of TSEs.

**Material and Methods**: We performed *in-silico* docking of 125 drug candidates to various isoforms of the human prion protein including wild type (WT) PrP^C^, mutants P102L and E200K, as well as two different PrP^Sc^conformations, the in-register cryo-EM based PIRIBS structure [Kraus et al., 2021] and the β-solenoidal theoretically predicted model [Spagnolli et al., 2019]. Importantly, docking of drug candidates against the PrP^Sc^models allowed for a direct identification of binding sites at the surface of misfolded fibrillary seeds.

**Results**: Out of 125 drug candidates, ten structurally diverse compounds with the strongest binding scores were identified. Effects of electrostatic and sterical compatibility with cavities at the surface of PrP were analyzed. The identified compounds were separated in groups that may target different stages of misfolding and aggregation of the prion protein.

**Conclusions**: suramin, Fe(III)TMPyP, amphotericin B, methyl blue, tegobuvir, tariquidar, diosgenin, rapamycin, and elacridar are the most promising drugs that may bind to PrP^Sc^. However, some of them also exhibit a strong binding propensity to PrP^C^, which might interfere with its functions. Drug candidates that strongly bind exclusively to PrP^Sc^include amphotericin B, diosgenin, tariquidar, filipin, cmp10, NPR-015, doxycycline, cmpd12, and tetracycline.

**Funded by**: Alberta Innovates

**Grant number**: 20,170,016

**Acknowledgement**: Access to drug data offered by the DrugBank is gratefully acknowledged.

**RNA Editing in Neurodegenerative Disorders**

Theodoros Sklaviadis

Laboratory of Pharmacology, School of Pharmacy, Aristotle University of Thessaloniki, Thessaloniki, Greece

**Aims**: We aimed to address how RNA editing patterns of different tissue/cell populations contribute to the pathologies of protein aggregation and subsequent neurodegeneration (sCJD, PD, AD, ALS & MS). RNA editing is a prevalent type of RNA modification where the RNA sequences are altered through insertion, deletion or substitution of nucleotides. RNA editing may modify the structure, stability, and processing of a transcript with subsequent translation deregulation. Although the brain is an immune-privileged organ, cross-talks between peripheral and central inflammation have been reported. Therefore, it is obligatory to study in parallel RNA editing in distinct tissue (tissue/cell populations, in each of the above pathologies, in order to understand if editing contribute to pathogenesis as a potential origin of an initial epitranscriptomic error.

**Material and Methods**: We utilized a combination of several disease patient *in vitro* and **in vivo** transcriptomes, that faithfully recapitulate the molecular and pathological alterations of the abovementioned neurodegenerative diseases. RNA sequencing data were subjected to an advanced ‘in house’ QC pipeline, followed by gene expression and RNA editing analyses based on the DESeq and REDItools/SPRINT algorithms. Integrative and comparative editome analyses followed by Gene Ontology and pathway gene enrichment has identified common RNA editing alterations in the context of each disease-protein aggregation.

**Results**: Remarkably, we find distinct changes in RNA editing profiles with specifically altered RNA edited pathways (eg., ER stress, lysosome, splicing) with a common pattern presented in total brain tissue as well as in distinct cellular subtypes (neuronal and immune) in the context of neurodegenerative disease progression. It seems that RNA editing has a protective role for preclinical and clinical disease stages. Furthermore, differentially edited sites were preferentially predicted to disrupt miRNA binding and induce coding changes in genes previously associated with disease phenotypes. These findings suggest that RNA editing has an important post-transcriptional regulatory role in all neurodegenerative pathogenesis.

**Conclusions**: Our results highlight a great need for the inclusion of epitranscriptomic mechanisms in the study of neurodegeneration. We hope our work will pave the way for the discovery of more effective therapies to treat patients suffering from neurodegenerative diseases.

**Funded by**: This research has been co‐financed by the European Regional Development Fund of the European Union and Greek national funds through the Operational Program Competitiveness, Entrepreneurship and Innovation, under the call RESEARCH – CREATE – INNOVATE (project code: T1EDK-03884); Hellenic Foundation for Research and Innovation (H.F.R.I) under the “2nd Call for H.F.R.I

Research Projects to support Post-Doctoral Researchers” (project number: 1146).

**Single cell transcriptional profiling of the cortex and hippocampus from mice infected with RML scrapie**

Jessy Slota^a,b^, Babu Sajesh^a^, Kathy Frost^a^, Sarah Medina, and Stephanie Booth^a,b^

^a^One Health Divsion, National Microbiology Laboratory, Public Health Agency of Canada, Winnipeg, MB, Canada; ^b^Medical Microbiology and Infectious Diseases, University of Manitoba, Winnipeg, MB, Canada

**Aims**: The precise molecular mechanisms that link PrP^Sc^accumulation in the brain with associated pathological changes have remained a mystery despite intense efforts to characterize transcriptional changes within bulk brain tissues. This may be partly attributed to the inherent complexity of numerous brain cell subtypes and the overwhelming induction of inflammatory transcripts that possibly mask subtle changes occurring within neurons. Thus, improved approaches could provide insights into mechanisms of pathogenesis and/or clarify the response of cellular subpopulations to prion infection. Here, we attempt to profile transcriptional changes in cortical and hippocampal tissues from a well-established mouse model of RML scrapie using single-cell RNA sequencing (scRNAseq).

**Material and Methods**: CD1 mice were intraperitoneally inoculated with either scrapie (RML) or non-infectious (Mock) brain homogenate and sacrificed at onset of clinical signs ranging from 150–173 days post infection (dpi). Cortical and hippocampal brain tissues were dissected and dissociated with papain before clearing debris using Miltenyi debris removal solution and myelin removal beads. The single-cell suspensions were processed using the 10x genomics 3’ gene expression reagents according to manufacturer’s instructions and the resulting libraries were sequenced on an Illumina Nextseq2000 instrument. Sequencing data was pre-processed using the cellranger pipeline and analyzed further using the Seurat R package.

**Results**: We prepared scRNAseq libraries from the cortex and hippocampus taken from 5 Mock treated mice (collected at timepoints from 110 dpi to 180 dpi) and 7 RML treated mice (collected at clinical endpoints from 150 to 173 dpi). Each library was prepared from a target population of 10,000 cells and sequenced to a minimum depth of 30,000 reads/cell. Using graph based clustering and dimensionality reduction techniques, we identified multiple populations of microglia, astrocytes, vascular cells and neurons. This will enable us to track prion disease-associated markers across brain cell types and compare how different cellular subpopulations respond to prion infection.

**Conclusions**: scRNAseq may represent a useful tool to dissect prion induced transcriptional responses for specific subpopulations of microglia, astrocytes, vascular cells and neurons.

**Funded by**: Public Health Agency of Canada

**Acknowledgement**: We wish to thank the staff of the veterinary technical services at the NML for animal manipulations and the staff of DNA core services at the NML for assistance with sequencing runs.

**Distribution of PrP^CWD^in tissues of CWD affected sika deer using RT-QuIC following experimental oral transmission**

HJ Sohn^a^, KJ Park^a^, YR Lee^a^, HC Park^a^, and G Mitchell^b^

^a^Foreign animal disease division (FADD), Animal and Plant Quarantine Agency (APQA), Gimcheon, Korea; ^b^National & OIE Reference Laboratory for Scrapie and CWD, Canadian Food Inspection Agency, Ottawa, Ontario, Canada

**Aims**: Chronic wasting disease (CWD) is the only prion disease affecting free-ranging animals, reported in North America, South Korea and Scandinavia. Unlike in most other prion diseases, CWD agents are shed in blood, saliva, urine and feces which most likely contributes to the horizontal transmission between cervid species. Using NaPTA precipitation and real-time quaking-induced conversion (NaPTA/RT-QuIC) or only RT-QuIC, we established an ultrasensitive detection method for PrP^CWD^in the various tissues and body fluids of CWD affected sika deer following experimental oral transmission.

**Material and Methods**: Two Sika deer were orally inoculated with a brain homogenate (5 g) prepared from a farmed Canadian elk with clinical CWD. Deer were euthanized due to intercurrent disease or following the development of signs consistent with terminal CWD.

An array of tissues was collected and stored frozen, and were tested for the presence of PrP^CWD^by RT-QuIC or NaPTA/RT-QuIC.

**Results**: Primary oral transmission of CWD from elk to sika deer occurred in all inoculated animals, and was detected by RT-QuIC. Consistent with other cervids in the terminal stages of CWD, pathological prions were distributed throughout the central nervous system and lymphoid tissues including spleen. PrP^CWD^was also detected in the urinary system (kidney, urinary bladder, urine), salivary system (salivary glands and saliva), heart and skin. Detection in the skin occurred after collagenase treatment, and PrP^CWD^in the urinary system was associated with renal nerve plexus.

**Conclusions**: CWD transmits efficiently from elk to sika deer via the oral route. Widespread detection of PrP^CWD^by RT-QuIC suggests that, similar to other cervid species, infectivity is distributed throughout a wide range of tissues in sika deer with clinical CWD.

**Funded by: Animal and Plant Quarantine Agency**

**Grant number: B-154085-2022-24-01**

**Detection of PrP^CWD^in ear skin from CWD affected cervid**

HJ Sohn, KJ Park, HC Park, YR Lee, and HE Kang

Foreign animal disease division (FADD), Animal and Plant Quarantine Agency (APQA), Gimcheon, Korea

**Aims**: Chronic wasting disease (CWD) is the only prion disease affecting free-ranging animals, reported in North America, South Korea and Scandinavia. Using real-time quaking-induced conversion (RT-QuIC), we established an ultrasensitive detection method for PrP^CWD^to determine the availability of using ear skin for preclinical diagnosis, here we report RT-QuIC assays of ear skin samples from cervid. In addition, we confirmed infectivity of four RT-QuIC positive skins thorough Tg Elk mouse bioassay.

**Material and Methods**: CWD-derived samples were obtained from animals at different clinical stage in Korean cervid farm. This classification of clinical stage was assigned based on post mortem diagnosis of PrP^CWD^detection in RPLN, tonsil and obex.

An array of tissues was collected and prepared 10% ear homogenate of farmed Korean cervid and then were tested for the presence of PrP^CWD^by RT-QuIC after incubated collagenase (2 mg/ml) and phospholipase (1.6 umol) 37°C for 1 hr. To confirm ear skin infectivity, we inoculated 4 RT-QuIC positive tissues into Tg elk mice (n = 6 per group) by intracranial route.

**Results**: PrP^CWD^is not evenly distributes in cervid ear and detects in seven out of eight cervid at late pre-symptomatic stage. The average ThT fluorescence unit in ear skin gave rapid increase within 11 h in just two cervid of the last end-stage. All inoculated groups showed 80 ~ 100% attack rates and developed the disease during survival times.

**Conclusions**: Based on our data, we were confirmed in PrP^CWD^detection and infectivity from CWD infected cervid ear skin. And raising the possibility that simple ear-punch biopsies might be used for CWD antemortem monitoring.

**Funded by: Animal and Plant Quarantine Agency**

**Grant number: B-154085-2022-24-01**

**Carrot plants as potential vectors for CWD transmission**

Paulina Soto^a,b^, Francisca Bravo-Risi^a,b^, Claudio Soto^a^, and Rodrigo Morales^a,b^

^a^Department of Neurology, McGovern Medical School, University of Texas Health Science Center at Houston, Texas, USA; ^b^Universidad Bernardo O’Higgins, Santiago, Chile

Prion diseases are infectious neurodegenerative disorders afflicting humans and other mammals. These diseases are generated by the misfolding of the cellular prion protein into a disease-causing isoform. Chronic wasting disease (CWD) is a prevalent prion disease affecting cervids (captive and free-range). CWD is thought to be transmitted through direct animal contact or by indirect exposure to contaminated environments. Many studies have shown that infectious prions can enter the environment through saliva, feces, or urine from infected animals and decaying carcasses. However, we do not fully understand the specific contribution of each component to disease transmission events. Plants are logical environmental components to be evaluated since they grow in environments contaminated with CWD prions and are relevant for animal and human nutrition.

**Aims**: The main objective of this study is to study whether prions are transported to the roots and leaves of carrots, an edible plant commonly used in the human diet and as deer bait.

**Methods**: We have grown carrot plants in CWD-infected soils. After 90 days, we harvested the carrots and separated them from the leaves. The experiment was controlled by growing plants in soil samples treated with brain extracts from healthy animals. These materials were interrogated for their prion seeding activity using the Protein Misfolding Cyclic Amplification (PMCA) technique. Infectivity was evaluated in mouse bioassays (intracerebral injections in Tg1536 mice). The animals were sacrificed when they showed established signs of prion disease. Animals not displaying clinical signs were sacrificed at 600 days post-inoculation.

**Results**: The PMCA analysis demonstrated CWD seeding activity in soils contaminated with CWD prions, as well as in carrot plants (leaves and roots) grown on them. Bioassays demonstrated that both leaves and roots contained CWD prions in sufficient quantities to induce disease (92% attack rate). As expected, animals treated with prion-infected soils developed prion disease at shorter incubation periods (and complete attack rates) compared to plant components. Animals treated with soil and plant components exposed with CWD-free brain extracts did not display prion-associated clinical signs or evidence of sub-clinical prion infection.

**Conclusions**: We show that edible plant components can absorb prions from CWD contaminated soils and transport them to their aerial parts. Our results indicate that plants could participate as vectors of CWD transmission. Importantly, plants designated for human consumption represent a risk of introducing CWD prions into the human food chain.

**Funded by**: NIH

**Grant number**: R01AI132695

**Chronic wasting disease detection in environmental and biological samples from a taxidermy site**

Paulina Soto^a,b^, J. Hunter Reed^c^, Mitch Lockwood^c^, and Rodrigo Morales^a,b^

^a^Department of Neurology, McGovern Medical School, University of Texas Health Science Center at Houston, Texas, USA; ^b^Universidad Bernardo O’Higgins, Santiago, Chile; ^c^Texas Parks and Wildlife Department, Texas, USA

Chronic wasting disease (CWD) is a transmissible spongiform encephalopathy affecting captive and free-ranging cervids (*e.g*., mule deer, white-tailed deer, elk, reindeer, and moose). Nowadays, CWD is widely distributed in North America. It is suggested that CWD spreads due to direct animal contact or through exposure to contaminated environments previously inhabited by infected animals. CWD may also be spread through the movement of infected animals and carcasses. Taxidermy practices involve processing deer tissues (or whole animal carcasses). In many cases, the CWD status of processed animals is unknown. This can generate risks of disease spread and transmission. Taxidermy practices include different steps involving physical, chemical, and biological procedures. Without proper tissue handling or disposal practices, taxidermist facilities may become a focus of prion infectivity.

**Aims**: In this study, we evaluated the presence of infectious prions in a taxidermy facility believed to be exposed to CWD. Detection was performed using the Protein Misfolding Cyclic Amplification (PMCA) technique in biological and inert environmental samples.

**Methods**: We collected biological and environmental samples (plants, soils, insects, excreta, and others) from a taxidermy facility, and we tested these samples using the PMCA technique. In addition, we swabbed different surfaces possibly exposed to CWD-infected animals. For the PMCA reaction, we directly used a swab piece or 10 µL of 20% w/v homogenized samples.

**Results**: The PMCA analysis demonstrated CWD seeding activity in some of the components of this facility, including insects involved in head processing, soils, and a trash dumpster.

**Conclusions**: Different areas of this property were used for various taxidermy procedures. We were able to detect the presence of prions in i) soils that were in contact with the heads of dead animals, ii) insects involved in the cleaning of skulls, and iii) an empty dumpster where animal carcasses were previously placed. This is the first report demonstrating that swabbing is a helpful method to screen for prion infectivity on surfaces potentially contaminated with CWD. These findings are relevant as this swabbing and amplification strategy may be used to evaluate the disease status of other free-ranging and captive settings where there is a concern for CWD transmissions, such as at feeders and water troughs with CWD-exposed properties. This approach could have substantial implications for free-ranging cervid surveillance as well as in epidemiological investigations of CWD.

**Funded by: USDA**

**Grant number**: AP20VSSPRS00C143

**Nasal bot: an emerging vector for natural chronic wasting disease transmission**

Paulina Soto^a,b^, Francisca Bravo-Risi^a,b^, Carlos Kramm^a^, Nelson Perez^a^, Rebeca Benavente^a^, J. Hunter Reed^c^, Mitch Lockwood^c^, Tracy A. Nichols^d^, and Rodrigo Morales^a,b^

^a^Department of Neurology, McGovern Medical School, University of Texas Health Science Center at Houston, Texas, USA; ^b^Universidad Bernardo O’Higgins, Santiago, Chile; ^c^Texas Park and Wildlife Department, Texas, USA; ^d^Veterinary Services Cervid Health Program, United States Department of Agriculture, Animal and Plant Health Inspection Service, Fort Collins, Colorado, USA

Chronic wasting disease (CWD) is a fatal neurodegenerative disease that affects farmed and free-ranging cervids populations. The spread of CWD in cervids is thought to occur through the direct contact between cervids or through the exposure of naïve animals to contaminated environments. Parasites are known vectors of multiple diseases in animals. However, the potential role of parasites in CWD transmission remains unclear.

**Aims**: The main objective of this study was to determine if CWD prions could be detected in the larvae of deer nasal bot flies, a common deer parasite, taken from CWD-infected white-tailed deer (*Odocoileus virginianus*).

**Methods**: Bot fly larvae were collected from the nasal cavity of naturally infected CWD- positive or CWD non-detect white-tailed deer. The CWD seeding activity of the larvae was interrogated by PMCA. Prion infectivity was also evaluated in cervidized transgenic mouse bioassay (intra-cerebral administration in Tg1536 mice). Mice inoculated with bot larvae homogenate were sacrificed when they showed established signs of prion disease, or at extended periods after treatment (600 days). All inoculated mouse brains were evaluated for protease resistant prions to confirm clinical or sub-clinical infection. Bot larvae from CWD non-detect deer were used as controls. To further mimic environmental transmission, bot larvae homogenates were mixed with soils and plants were grown on them. Both plants and soils were tested for prion seeding activity.

**Results**: PMCA analysis demonstrated CWD seeding activity in nasal bot larvae from captive and free-ranging white-tailed deer. CWD-contaminated bots efficiently infected transgenic mice, with attack rates and incubation periods suggesting high infectivity titers. Further analyses of treated animals (biochemical characterization of protease resistant prions and immunohistochemistry) confirmed prion infection. Analyses on dissected parts of the bot larvae demonstrate that the infectivity is concentrated in the larvae cuticle (outer part). Nasal bot larvae extracts mixed with soils showed seeding activity by PMCA. Interestingly, plants grown in soil contaminated with the nasal bot larvae extract were found to produce seeding activity by PMCA.

**Conclusion**: In this study we described for the first time that deer nasal bot larvae from CWD-infected deer carry high CWD infectivity titers. We also demonstrate that CWD prions in these parasites can interact with other environmental components relevant for disease transmission. Considering this information, we propose that deer nasal bot larvae could act as vectors for CWD transmission in wild and farming settings.

**Funded by**: NIH/NIAID and USDA/APHIS

**Grant number**: R01AI132695 and AP20VSSPRS00C143

**Large and small extracellular vesicles differ in the level of prion associated infectivity in cell culture**

Jakub Soukup^a,b^, Sami Kereïche^c^, Tibor Moško^a^, and Karel Holada^a^

^a^Institute of Immunology and Microbiology, First Faculty of Medicine, Charles University, Prague, Czech Republic; ^b^Department of Genetics and Microbiology, Faculty of Science, Charles University, Prague, Czech Republic; ^c^Institute of Biology and Medical Genetics, First Faculty of Medicine, Charles University, Prague, Czech Republic

**Aims**: Prion diseases are fatal neurodegenerative disorders connected to the accumulation of misfolded isoform of prion protein (PrP^TSE^). PrP^TSE^spread in the organism is critical for the disease manifestation and direct contact of PrP^TSE^with cellular prion protein (PrP^C^) is needed for cell infection. Three ways of PrP^TSE^intercellular transmission were identified: cell-to-cell contact, tunnelling nanotubes and extracellular vesicles (EVs). EVs are capable of long-distance transmission and crossing of blood-brain barrier. EVs are divided by the mechanism of biogenesis and size to exosomes, microvesicles and apoptotic bodies. Yet, they cannot be isolated in clean fractions, they are referred as small EVs (sEVs, mostly exosomes) and large EVs (lEVs, mostly microvesicles). The aim of our study was to compare the ability of sEVs and lEVs to spread prions in cell culture.

**Material and Methods**: EVs were isolated from medium of RML chronically infected mouse CAD5 and PK1 cells. lEVs were isolated by centrifugation at 14,000 × g. sEVs were isolated by 110,000 × g using sucrose cushion. Isolated EVs were characterized by cryo-TEM and western blot utilizing calnexin, β-1 integrin, HSP70, TSG-101 and Alix markers. Isolated lEVs and sEVs were used for infection of native CAD5 and PK1 cells in two ways: 1) infection by the fractions containing relative amount of sEVs or lEVs present in the conditioned medium; 2) infection by sEVs and lEVs fractions standardized to contain the same amount of protein. The effectiveness of prion infection was analysed by cell blot, western blot and cell scrapie assay. Prion converting activity was measured by RT-QuIC.

**Results**: The isolated CAD5 lEVs contained ~2× less total protein and had different protein profile then sEVs fractions. Both fractions were enriched in PrP^C^and PrP^TSE^compared to the cell lysate. The lEVs fraction demonstrated ~10× higher prion converting activity measured by RT-QuIC. In the infectious experiments, lEVs infected native CAD5 4× more effectively than sEVs even though they contained less total protein. The infection of PK1 cell by lEVs was also (1.5×) more effective. To confirm the higher presence of prions in lEVs, we standardized amount of protein in the fractions and repeated the experiments. Again, all employed methods showed stronger infection by lEVs in both studied cell lines.

**Conclusions**: Our data revealed that lEVs fraction contains more infectious prions and has higher prion converting activity than more abundant sEVs. Our study suggests that microvesicles may contribute to the spread of PrP^TSE^more than widely investigated exosomes.

**Funded by**: Charles University

**Grant number**: Cooperatio 207,032–3, SVV 260520

**Acknowledgement**: The authors thank Charles Weissmann and Dennis Burton from The Scripps Research Institute for CAD 5 cells.

**4R tau seeds are a prevalent co-pathology across neurodegenerative diseases**

Heidi G. Standke^a^, Matteo Manca^a^, Mikayla L. Huntly^a^, Olivia R. Thomas^a^, Yongya Kim^b^, Annie Hiniker^b^, David G. Coughlin^b^, Douglas Galasko^b^, and Allison Kraus^a^

^a^Department of Pathology, Case Western Reserve University School of Medicine, Cleveland, OH, USA; ^b^Department of Neurosciences, University of California San Diego, CA, USA

**Aims**: Tauopathies are neurodegenerative diseases defined by the accumulation of misfolded tau, with 3 R/4R tau deposits being characteristic of Alzheimer’s disease (AD), 3 R tau of Pick’s disease (PiD), and 4R tau of progressive supranuclear palsy (PSP) and corticobasal degeneration (CBD). Using ultrasensitive real-time quaking induced conversion (RT-QuIC) assays selective for different tau strains, we identified 3 R/4R tau seeds are a prominent co-pathology across neurodegenerative diseases, including synucleinopathies and 4R tauopathies. Here, we explore the prevalence of 4R tau seeds as a co-pathology in non-4R tauopathies using 4R RT-QuIC for the detection of 4R tau seeds in brain tissue of neuropathologically well-defined neurodegenerative cases. We also exploit the ability of the RT-QuIC assay to discriminate disease specific 4R tau amyloid core structures characteristic of PSP and CBD (Shi et al., 2021).

**Material and Methods**: We used 4R RT-QuIC (Saijo et al., 2020) to evaluate 63 neuropathologically well-characterized brain tissue samples including cases with AD (n = 15), Parkinson’s disease (PD) (n = 8), multiple system atrophy (MSA) (n = 6), Lewy body dementia (LBD) (n = 13), PSP (n = 6) CBD (n = 6) and age-comparable controls consisting of cases <Braak III (n = 9). RT-QuIC readouts were further analyzed for differences in ThT amplitude, and we used electron microscopy (EM) to examine ultrastructural characteristics of the seeded amyloids.

**Results**: 4R RT-QuIC identified all 4R tauopathy cases, with 4R tau seeds being on average ~ 1,000-fold higher than age-comparable controls. However, 4R tau seeds were also detected, albeit in predominantly low quantities, in non-4R tauopathy diseases including select AD (11/15), PD (7/8), MSA (4/6), and LBD (9/13) cases. While a previous study showed low 4R seeding activity in brain tissue of some AD and PiD cases (Saijo et al., 2020), here we evaluate additional disease diagnoses and cases than previously reported, including in primary synucleinopathies. PSP and CBD seeds could be distinguished by differences in resulting RT-QuIC ThT amplitude readouts.

**Conclusions**: 4R RT-QuIC assay was highly specific in the detection of 4R tau seeds in 4R tauopathies including PSP and CBD, and further distinguished PSP and CBD strains via ThT amplitudes of the RT-QuIC readouts, Thus, with use of selective RT-QuIC assays for 3 R/4R and 4R tau seeds, we can detect and discriminate tau strains even when they co- occur. Our results further indicate 4R tau seeds can occur across other neurodegenerative disorders, however to a lesser extent when compared to 3 R/4R tau seeds.

**References**: Saijo, E., Metrick, M. A., Koga, S., Parchi, P., Litvan, I., Spina, S., Boxer, A., Rojas, J. C., Galasko, D., Kraus, A., Rossi, M., Newell, K., Zanusso, G., Grinberg, L. T., Seeley, W. W., Ghetti, B., Dickson, D. W., & Caughey, B. (2020). 4-Repeat tau seeds and templating subtypes as brain and CSF biomarkers of frontotemporal lobar degeneration. *Acta Neuropathologica, 139*(1), 63–77. https://doi.org/10.1007/s00401- 019-02080-2

Shi, Y., Zhang, W., Yang, Y., Murzin, A. G., Falcon, B., Kotecha, A., van Beers, M., Tarutani, A., Kametani, F., Garringer, H. J., Vidal, R., Hallinan, G. I., Lashley, T., Saito, Y., Murayama, S., Yoshida, M., Tanaka, H., Kakita, A., Ikeuchi, T., … Scheres, S. H. W. (2021). Structure-based classification of tauopathies. *Nature, 598*(7880), 359–363. https://doi.org/10.1038/s41586-021-03911-7

**Funded by**: Biomarkers Across Neurodegenerative Diseases (BAND) **Grant number**: R01NS118760 and R01AG067607

**Mechanisms of prion-induced damage in retina: Roles of microglia and sites of PrPSc deposition**

James F. Striebel^a^, Brent Race^a^, James A. Carroll^a^, Jacqueline Leung^a^, Cindi Schwartz^a^, Katie Williams^a^, Chase Baune^a^, Mikael Klingeborn^b^, and Bruce Chesebro^a^

^a^Laboratory of Persistent Viral Diseases, Rocky Mountain Laboratories, National Institute of Allergy and Infectious Diseases, National Institutes of Health, Hamilton, Montana, USA; ^b^Department of Ophthalmology, Duke Eye Center, Duke University, Durham, NC, 27,710, USA

**Aims**: In prion and prion-like disorders, such as Alzheimer’s and Parkinson’s diseases, accumulation of misfolded host-proteins, reactive-gliosis and neuronal damage are central to pathogenesis in brain, and retina. Retinal pathology is concurrent with brain pathology, and can now be assessed with non-invasive imaging, allowing early diagnosis. Yet, details of retinal pathology are needed. Previously, we found that prion-induced retinal damage mainly occurred in photoreceptor cells and, that onset of apoptosis and photoreceptor degeneration correlated with two key events: 1) invasion of photoreceptor layers by activated microglia, and 2) accumulation of misfolded disease-associated prion protein (PrPSc) in photoreceptor-associated layers. These correlations suggest a causal role for microglia and/or PrPSc accumulation in retinal pathogenesis.

**Material and Methods**: To investigate the role of microglia in retinal degeneration, we fed prion-infected mice a *CSF-1* receptor-blocking drug(PLX5622) to eliminate microglia *in vivo*, and effects on retinal degeneration were analyzed over time. In separate experiments, to follow PrPSc deposition in retina, we used confocal, epifluorescent and electron microscopy to track its association with damage to critical retinal structures, after intracerebral prion-inoculation. The association of PrPSc with retinal-neuron subtypes was also analyzed.

**Results**: PLX5622 was highly effective at ablating retinal microglia. However, lack of microglia during prion infection did not prevent photoreceptor degeneration. Therefore, microglia were not required for the photoreceptor damage process. In fact, mice lacking microglia had faster onset of photoreceptor damage, suggesting microglia were protective.

The earliest PrPSc deposits were consistently found within inner segments of cone neurons, which remarkably, compromise only 3% of photoreceptors in mice. Subsequently, in both rods and cones, PrPSc was associated with the connecting-cilium, joining photoreceptor inner and outer segments. This site of deposition may interfere with transport of molecules critical to phototransduction and cell viability. Slightly later in disease, PrPSc accumulations were detected on bipolar cell dendrites at their connections with photoreceptor cells. These connections, called ribbon synapses, can be detected by electron and/or immunofluorescent microscopy and their decrease was concomitant with PrPSc accumulation and photoreceptor death.

**Conclusions**: Importantly, our data suggest microglia play a protective role in prion-induced retinal degeneration, slowing the degenerative process. However, PrPSc accumulations, near connecting-cilia and in ribbon synapses, were critical early events preceding damage and death of photoreceptors. The preference of PrPSc for early accumulation in cone photoreceptors and lack of PrPSc deposition and damage in other retinal-neurons, such as horizontal cells, was intriguing and indicated high selectivity among neuron types for injury by prions.

**Funded by**: National Institutes of Health, Department of Intramural Resources

**Acknowledgement**: National Institutes of Health, Department of Intramural Resources

**Faithful propagation of vCJD prions from frozen and fixed central nervous system and appendix tissues using highly sensitive Protein Misfolding Cyclic Amplification**

Suzanne Suleiman^a^, Lynne I. McGuire^b^, Angela Chong^a^, Diane L. Ritchie^a^Aileen Boyle^b^, Lee McManus^b^, Fraser Brydon^a^, Colin Smith^a^, Richard Knight^a^, Abigail B. Diack^b^, Alison Green^a^, and Marcelo A. Barria^a^

^a^National CJD Research & Surveillance Unit, Centre for Clinical Brain Sciences, Deanery of Clinical Medicine, The University of Edinburgh, Edinburgh, UK; ^b^The Roslin Institute and R(D)SVS, The University of Edinburgh, Easter Bush, UK

**Aims**: The Appendix Studies I, II & III aimed to estimate the UK prevalence of asymptomatic variant Creutzfeldt-Jakob disease (vCJD), following exposure of the population to the bovine spongiform encephalopathy (BSE) agent in the late 1980s and early 1990s. These studies evaluated the presence of the abnormal prion protein aggregates, in anonymised archived formalin-fixed paraffin-embedded (FFPE) appendectomy samples, by immunohistochemistry. Although there was concordance in the estimated prevalence of asymptomatic vCJD in these studies, the identification of positive specimens from pre- and post-BSE-exposure periods in Appendix study III raised questions regarding the nature and origin of the abnormal prion protein that was detected. We aimed to develop a solid platform for investigating the *in vitro* and *in vivo* propagation properties of the abnormal prion protein in the positive samples from Appendix studies II and III.

**Materials and Methods**: We used post-mortem frozen and FFPE brain and appendix tissues from a confirmed vCJD patient to optimise a procedure for extracting the disease-associated prion protein. The recovered material was used to seed the highly sensitive Protein Misfolding Cyclic Amplification assay (hsPMCA). We then tested whether the prion strain features of the extracted and amplified material are conserved, by means of bioassay using both wild-type (RIII) and gene-targeted mice expressing *PRNP* 129 MM (HuMM).

**Results**: The extracted abnormal prion protein from frozen brain and appendix specimens from a single vCJD case, could be successfully propagated by hsPMCA. Crucially, the *in vitro* amplified material was protease-resistant, and produced the characteristic ‘2B’ biochemical isotype observed in vCJD patients. Inoculation of serially-amplified products resulted in successful transmission to both RIII and HuMM mice. The biochemical and histopathological features observed for the two mouse lines are consistent with that of the vCJD prion agent demonstrating they are maintained upon transmission of the amplified material.

**Conclusions**: These initial observations prove that vCJD prions can be successfully extracted and amplified from FFPE brain and appendix tissue specimens. The hsPMCA can faithfully replicate the infectivity and strain properties of the vCJD prion agent. This information will provide a solid and reliable platform to proceed with analysis on the archived FFPE appendix tissue derived from the Appendix II and III surveys, to further evaluate the nature of the abnormal prion protein detected in the positive samples.

**Funded by**: The work presents in this abstract is based on independent research commissioned and funded by the Policy Research Programme, Department of Health and Social Care and the Scottish Government, grant number PR-R17-0916-23,001, and the National CJD Research and Surveillance Unit (NCJDRSU), PR-ST-0614-00008_18. The views expressed in this abstract are those of the author(s) and not necessarily those of the NHS, the NIHR, the Department of Health and Social Care, the Scottish Government, ‘arms’ length bodies or other government departments.

**Grant number**: PR-R17-0916-23,001

**Acknowledgement**: We are particularly thankful to the families of patients for their cooperation.

**Detailed investigation of the role played by residue 226 of PrP in chronic wasting disease pathogenesis and strain selection**

Julianna L. Sun, Sarah Jo Kane, Sehun Kim, Jenna Crowell, Bailey Webster, Emma Raisley, and Glenn C. Telling

Prion Research Center, Colorado State University, Fort Collins, USA

**Aims**: Whereas North American deer or moose PrP encodes glutamine at residue 226 (Q226), North American elk PrP encodes glutamate (E226). To precisely assess the effects of this difference on CWD pathogenesis, we created gene targeted (Gt) mice in which the murine PrP coding sequence was targeted and replaced with CerPrP-Q226 or CerPrP-E226, referred to as GtQ and GtE mice. Previous studies showed that GtQ and GtE mice were susceptible to North American CWD, and that time to disease onset was faster in GtE mice. To fully understand the mechanism underlying this difference, we conducted a longitudinal analysis of disease in GtE and GtQ.

**Material and Methods**: GtQ and GtE mice were intracerebrally inoculated with elk CWD prions previously passaged in transgenic mice expressing CerPrP^C^-Q226 or CerPrP^C^-E226 respectively. Mice were collected every 15 days until terminal disease. Brain extracts were analyzed for PrP27-30 and glycoform ratio profiling by western blotting, disease-associated PrP by immunohistochemistry and histoblotting, titer determination by the cervid prion cell assay (CPCA), and the appearance of PrP^Sc^using mAb PRC7 in ELISA format.

**Results**: While GtE mice succumbed to clinical disease after 196 ± 4 days, disease onset in GtQ mice was protracted by > 40 % (277 ± 7 days). PrP^Sc^was detectable by immunoblotting and ELISA ~ 100 days prior to the onset of clinical signs with levels increasing steadily until mice became moribund. CerPrP^Sc^-Q226 was more extensively glycosylated than CerPrP^Sc^-E226. CWD titers (assessed by CPCA) increased exponentially during the early phases of disease in GtE and GtQ mice. PrP^Sc^was detected in the CNS of GtE and GtQ mice by immunohistochemistry and histoblotting as early as 15 to 30 days after infection. At the end stage of disease in GtE mice, CNS PrP^Sc^distribution was diffuse and symmetrical, while the brains of GtQ mice contained dense, disordered, asymmetrical PrP^Sc^deposits. By contrast, at timepoints earlier than 60 days, PrP^Sc^distribution was identical in GtQ and GtE mice.

**Conclusions**: Primary structural differences at residue 226 of CerPrP have pronounced effects on the outcomes of disease in Gt mice infected with North American CWD prions. Since North American deer and moose express CerPrP^C^-Q226 and elk express CerPrP^C^-E226, this study lends insight into the natural pathogenesis of CWD in these species.

**Funded by**: National Institutes of Health

**Grant number**: P01AI077774

**Acknowledgement**: Thank you to the members of the Telling lab who made this work possible.

**A diverse spectrum of novel strains among Nordic cervids with chronic wasting disease**

J. Sun^a^, S. Kim^a^, J. Crowell^a^, J. Bian^a^,S.-L Korpenfelt^b^, M. Nöremark^c^, S. Benestad^d^, and G. Telling^a^

^a^Prion Research Center (PRC), Colorado State University, Fort Collins, USA; ^b^Finnish Food Authority, Helsinki, Finland; ^c^Department of Disease Control and Epidemiology, Uppsala, Sweden; ^d^Norwegian Veterinary Institute, Oslo, Norway

**Aims**: Cervid PrP (CerPrP) coding sequences are generally invariant except at codon 226. Whereas deer, reindeer and moose PrP encode glutamine at residue 226 (Q226), elk or red deer may encode glutamate (E226) at this position. In order to precisely assess the effects of this primary structural difference on CWD strain selection we created gene targeted (Gt) mice in which the murine PrP coding sequence was targeted and replaced with that of CerPrP-Q226 or CerPrP-E226, referred to as GtQ and GtE mice. Our recently published studies showed that prion diseases in Norwegian reindeer and moose are caused by different CWD strains that are in turn different from those causing North American CWD. Here we aimed to assess the responses of GtE and GtQ mice and their overexpressing transgenic counterparts to additional emergent CWD prions with a view to defining CWD strain prevalence in Nordic countries and to compare this strain portfolio with that of North American CWD.

**Material and Methods**: We intracerebrally or intraperitoneally inoculated mice with homogenates of frozen brain and lymphoid tissue materials from Norwegian, Swedish, and Finnish CWD cases. We assessed conventional measures of prion strain properties including differences in susceptibilities and disease kinetics, neuropathology, lymphotropism, and various biochemical and cell biological assessments of the resultant prions. We also assessed the properties of these prions after iterative transmissions in mice.

**Results**: Certain characteristics of additional emergent CWD cases from Norway, Finland and Sweden are concordant with our previously published findings on differences between Norwegian and North American CWD, including responses to variation at PrP residue 226; biochemical profiles in the central nervous system; and ability to replicate in lymphoid tissues. However, additional differences between isolates, including pronounced conformational and incubation time differences, reveal a wide diversity of novel strain properties among Nordic CWD cases.

**Conclusions**: The differential responses of GtQ and GtE mice to CWD prions underscores the importance of this key primary structural difference on pathogenesis at the level of strain selection and, by extension, a rigorous and definitive means to identify and characterize the properties of novel emergent prion strains. Our new findings allow us to broaden our previous conclusion that the etiology of Nordic CWD is distinct from North American CWD. However, the incompletely overlapping properties of newly analyzed emergent CWD cases from Norway, Finland and Sweden reveals a surprising diversity of strains among Nordic cervids which stands in contrast the relatively consistent strain profile of established CWD in North America.

**Funded by**: The United States of America National Institutes of Health

**Grant numbers**: 1R01NS121682, 1R01NS109376, and PO1-0011877A

**Direct Observation of Prion Protein Fibril Elongation Kinetics Reveals Competing Fibril Populations with Distinct Strain-like Structural and Dynamic Properties**

Yuanzi Sun, Kezia Jack, Mark Batchelor, Daljit Sangar, Tiziana Ercolani, Laszlo Hosszu, John Collinge, and Jan Bieschke

UCL Institute of Prion Diseases/MRC Prion Unit, London, UK

**Aims**: In prion diseases, benign cellular prion protein (PrP^C^) is converted to PrP^Sc^, fibrillar assemblies of misfolded PrP. PrP^Sc^self-propagates by recruiting PrP^C^into the growing fibril. In vitro, PrP^C^is able to form amyloid fibrils that share structural characteristics of PrP^Sc^and that can elongate and replicate by a nucleation-polymerisation mechanism. The aim of our study was to analyse elongation kinetics of PrP fibrils on a single-particle level to reveal polymorphic fibril populations featuring structural and dynamic heterogeneity, which were previously hidden in ensemble measurements.

**Material and Methods**: PrP fibrils were seeded either from recombinant mouse PrP (90–230) seeds or authentic prion rods. The incorporation of mPrP (90–230) monomers was imaged in real time using total internal reflection microscopy and transient amyloid binding (TAB) super-resolution microscopy. Fibril structures were further characterized by electron microscopy, spectral profiling, and polarized TAB microscopy. Elongation kinetics of single PrP fibrils were recorded at different monomer concentrations, Temperatures, and Guanidine concentrations to analyse the mechanisms of fibril elongation.

**Results**: PrP fibrils elongated along a preferred direction by an intermittent ‘stop-and-go’ mechanism. Multiple competing fibril types with characteristic and distinct structures elongated from the same seed population at distinct rates and with distinct mechanisms. Fibrils fell into three main populations, type I, II, and III, whose formation was favoured under different solution conditions, respectively, and which maintained their structural and kinetic properties even under elongation conditions favouring a different fibril type. Type I, II, and III fibrils elongated by distinct mechanisms through the incorporation of unfolded (type III) or partially folded (type I, II) monomers.

The elongation kinetics of purified prion rods from two prion strains, RML and ME7, likewise exhibited unique kinetic features. The different fibril types showed distinct polarisation signals and spectral signatures differentiating helical and straight fibrils.

**Conclusions**: Analysis of PrP fibril seeding by TIRF and TAB super-resolution microscopy revealed polymorphic fibril populations featuring structural and dynamic heterogeneity similar to prion strains, which were previously hidden in ensemble measurements. This application demonstrates that single molecule measurements have the potential to differentiate in situ polymorphic fibril populations of amyloid and prions growing in competition and analyze their replication mechanisms.

**Funded by: UKRI, MRC, NIH**

**Grant number**: MRC MC_UU_00024/6, NIH, 1R21NS101588-01A1

**Acknowledgement**: The authors gratefully acknowledge the help of A. Wenborn and J. Wadsworth, MRC Prion Unit at UCL, in prion rod preparations.

**Mast Cells in Human Carotid Bodies Express PrP^C^**

Sweetland, G.D., Eggleston, C.J. and Kincaid, A.E.

Department of Pharmacy Sciences, Creighton University, Omaha Nebraska, USA

**Aims**: The human carotid bodies are highly vascularized structures located in the walls of the carotid arteries and contain chemosensitive cells that detect oxygen and carbon dioxide levels in blood. They are surrounded by leaky capillaries and are innervated by peripheral branches of the glossopharyngeal nerve; the central processes of this nerve terminate in the nucleus of the solitary tract in the brainstem. Given that the carotid bodies are exposed to blood and innervated by branches of a nerve that terminates in a site of early prion neuroinvasion we sought to determine if the normal isoform of the prion protein, PrP^C^, was expressed in cells in the carotid bodies.

**Material and Methods**: Segments of right and left arteries containing the bifurcation of the common carotid artery into internal and external carotid arteries were collected from human cadavers at the end of the medical school term. Nine artery segments from 5 donors were determined to be free of calcium deposits, embedded in paraffin, and sectioned at 7 µm. Every 10th section was stained with hematoxylin and eosin and examined using a light microscope for the presence of the carotid body. Sections containing the carotid body were immunohistochemically processed for the presence of PrP^C^using several antibodies directed against the prion protein (8H4, 3F4, Bar 224).

**Results**: Neither of the predominant cell types of the human carotid body (type I or type II) expressed the prion protein. A relatively small number of immunoreactive cells were detected in all carotid body samples using the 8H4 antibody. The cells were identified within the carotid body and surrounding connective tissue and the morphology and distribution of these cells resembled mast cells. Subsequent staining of sections using toluidine blue as a mast cell marker and 3 different antibodies directed against mast cells supported this finding. When cells were stained using the 8H4 antibody and lightly counterstained with toluidine blue the PrP^C^expressing cells were positively identified as mast cells.

**Conclusions**: This is the first report of mast cells in the human carotid body expressing PrP^C^. Mast cells have previously been shown to express PrP^C^and release it upon activation. The potential for PrP^C^conversion to PrP^Sc^and release via mast cells following exposure to prion-infected blood in the carotid body could result in either trafficking of infected mast cells to the brain, or transport of PrP^Sc^via the glossopharyngeal nerve to the nucleus of the solitary tract.

**Funded by**: National Institutes of Health

**Grant number**: NIH RO1 NS107246-01

**Acknowledgement**: We thank Carol Lomneth for her assistance with this project.

**A new bioassay for the sensitive detection of blood-borne CWD prions**

Alana M. Thackray^a^, Erin E. McNulty^b^, Amy V. Nalls^b^, Candace K. Mathiason^b^, and Raymond Bujdoso^a^

^a^Department of Veterinary Medicine, University of Cambridge, Madingley Road, Cambridge, CB3 0ES, UK; ^b^Department of Microbiology, Immunology and Pathology, Colorado State University, Fort Collins, CO, USA

**Aims**: Chronic Wasting Disease (CWD) is a natural transmissible spongiform encephalopathy, or prion disease, that affects free-ranging and captive cervids. Prion infectivity has been detected in the blood of CWD-infected cervids by bioassay in an appropriate indicator species, including the natural host. These present bioassays are cumbersome, time-consuming and expensive. We have developed a novel system using the invertebrate host *Drosophila* to establish a tractable bioassay to assess prions in the blood of CWD-infected animals.

**Material and Methods**: We have generated white tailed deer PrP *Drosophila* by pUASTattB-mediated transgenesis. Cervid PrP *Drosophila* were exposed to CWD-infected or prion-free white tailed deer brain homogenate at the larval stage. After hatching, the locomotor ability of *Drosophila* was assessed by a negative geotaxis climbing assay and their survival monitored. At regular time points during their lifespan, head homogenate was prepared from groups of euthanised flies and assessed for prion seeding activity by RT-QuIC. *Bona fide* prion infectivity was assessed by passage of fly head homogenate in cervid PrP transgenic mice.

**Results**: Cervid PrP *Drosophila* showed a neurotoxic phenotype in adulthood, evidenced by an accelerated loss of locomotor ability and survival, after exposure to CWD prions at the larval stage. The neurotoxic phenotype was coupled with the accumulation of prion seeding activity and *bona fide* prions that were transmissible to cervid PrP mice. Cervid PrP *Drosophila* showed a significant neurotoxic response to dilutions of 10**^–2^** to 10**^–14^** of CWD-infected cervid brain homogenate, which was considered sufficiently sensitive to detect prion infectivity in the blood of CWD-infected animals. We demonstrated this was the case by exposure of cervid PrP *Drosophila* to blood fractions from CWD-infected white tailed deer. Our experiments showed that cervid PrP *Drosophila* were capable of the detection of CWD prion-infected blood fractions, including whole blood, plasma and buffy coat cells, from cervids with experimental or natural CWD disease. In the case of buffy coat, cervid PrP *Drosophila* detected ≤100 blood cells from CWD-infected cervids.

**Conclusions**: These novel data show that cervid PrP *Drosophila* can efficiently bioassay prion infectivity in blood from CWD-infected hosts in a reasonably rapid, efficient and cost-effective manner.

**Funded by**: BBSRC (grant number PNAG/644), NIH (grant number NIAID 2R01AI112956-06).

**Acknowledgement**: We acknowledge the University of Cambridge Department of Genetics Fly Facility.

**Comparison of in vitro tests (PMCA and RT-QuIC) and bioassay for longitudinal prion detection in preclinical blood samples from BSE infected sheep**

Charlotte M. Thomas^a^, M. Khalid F. Salamat^a^, Jillian K. Cooper^b^, Kaetan Ladhani^b^, Florian Almela^c^, Olivier Andreoletti^d^, Daisy Bougard^c^ and E. Fiona Houston^a^

^a^The Roslin Institute, R(D)SVS, University of Edinburgh, Edinburgh, UK; ^b^The National Institute for Biological Standards and Controls (NIBSC), Medicines and Healthcare products Regulatory Agency (MHRA), South Mimms, UK; ^c^Etablissement Français du Sang, Inserm, Université de Montpellier, Montpellier, France; ^d^UMR INRA ENVT 1225- IHAP, École Nationale Vétérinaire de Toulouse, Toulouse, France

**Aims**: A diagnostic test for prion diseases that can reliably detect preclinical infection in easily accessible biological samples, such as blood, would be an invaluable tool to protect against potential spread of disease in humans and animals. Yet the development of such a test has remained challenging; partly due to the extremely low concentrations of disease-associated prion protein (PrP^Sc^) present in blood during preclinical stages of infection.

To determine which existing technologies are best able to detect preclinical infection, we have exploited an extensive archive of preclinical blood samples from sheep experimentally infected with BSE (as a model of vCJD) to compare three *in vitro* tests for prion disease, including two based on the ‘protein misfolding cyclic amplification’ assay (PMCA), and one based on ‘real time quaking-induced conversion’ (RT-QuIC). By comparing the outcome of these tests with infectivity titres (as determined by bioassay) we also investigated whether in vitro seeding activity correlated with prion infectivity in blood.

**Material and Methods**: Three blinded panels, comprising a longitudinal series of n = 139 blood samples from BSE-infected sheep collected at regular time points throughout the course of infection (alongside appropriate negative controls) were independently tested using two previously established PMCA methods, ‘microplate-based PMCA’ (mb-PMCA) [1] and ‘plasminogen bead-capture PMCA’ (capture-PMCA) [2], and a novel RT-QuIC assay which incorporates capture of PrP^Sc^from whole blood on iron oxide beads. A subset of blood samples represented in the panels was inoculated intracerebrally into transgenic (tgBov) mice overexpressing bovine PrP, to allow estimation of BSE infectivity titres.

**Results**: By comparing assay performance on equivalent blinded panels, we demonstrated that: (i) all three tests (mb-PMCA, capture-PMCA, RT-QuIC) showed 100% specificity, (ii) both PMCA assays were significantly more sensitive than RT-QuIC in detecting prion infected samples, (iii) the route of infection was a major factor influencing test results, as a substantially higher proportion of samples from intravenously infected sheep tested positive, compared to samples from orally infected animals. Furthermore, by comparing these data with bioassay results, we showed that in vitro seeding activity correlated with detectable prion infectivity in blood, and that infectivity titres were positively correlated with PrP^Sc^concentration.

**Conclusions**: The in vitro tests evaluated in this study represent accurate and sensitive diagnostic tools for detection of preclinical prionemia. It is hoped that the outcome of this study will improve methods for early detection of prion diseases and guide policies to reduce the risk of prion transmission through blood donation.

**References:**

[1] Moudjou M., et al. mBio 2014; 5(1):e00829-13.

[2] Bougard D, et al., Sci Transl Med 2016; 8(370):370ra182.

**Funded by: Department of Health, UK**

**Grant number: PR-R17-0916-23,006**

**Transmission of CH1641 in cattle**

Jemma K. Thorne, Janet Hills, M. Carmen Garcia-Pelayo, Timm Konold, and John Spiropoulos

Pathology and Animal Sciences Department, Animal and Plant Health Agency, Addlestone, UK

**Aims**: Classical BSE (C-BSE) was first identified in UK in the 1980s and is the only TSE that has proven zoonotic potential. The emergence of C-BSE was associated with a change in rendering practices implying that prions were able to escape inactivation. However, the exact origin of C-BSE remains unknown to this date although several theories have been proposed. CH1641 is a type of scrapie that biochemically is most akin to BSE. In addition CH1641 is the only scrapie type that can transmit as efficiently as C-BSE to bovinised mice (tg110) suggesting that the agent can propagate with ease on a bovine PrP background in contrast to other scrapie strains. This study was designed to investigate the transmissibility of CH1641 into cattle and characterise the resulting phenotype.

**Material and Methods**: To examine the ability of CH1641 to transmit to cattle, 5 animals were inoculated intracerebrally with an ovine CH1641 source. The clinical status of the animals was monitored and when they developed neurological signs they were euthanised on welfare grounds. Another 5 cattle were inoculated intracerebrally with saline solution to serve as negative, age-matched controls. Disease status was confirmed postmortem by statutory testing (Immunohistochemistry and Western blot).

**Results**: All CH1641 inoculated animals succumbed to clinical TSE with incubation periods 609–654 days post inoculation (dpi). One negative control died at 37 dpi and was excluded from the analysis as an intercurrent death. The remaining negative controls were killed at predetermined points to age match the CH1641 challenged cattle; they all were TSE negative. Western blot analysis revealed that in some animals the agent retained a CH1641 signature whilst in others the molecular profile acquired properties resembling C-BSE. Immunohistochemical analysis showed a similar phenotypic spectrum.

**Conclusions**: These preliminary data suggest that transmission of CH1641 in cattle is efficient and it results in a variable disease phenotype. Further studies are currently ongoing and include inoculation of bovinised and ovinised mice to identify if the CH1641 agent changed biological properties upon transmission to cattle. Secondary passages in cattle to investigate if intraspecies transmission can alter further the properties of the agent forcing it to converge towards C-BSE are also under consideration.

**Funded by**: Defra

**Grant number**: SE1962

**Acknowledgement**: Pathology and Animal Science Department staff members for technical excellence

**Chemical Synthesis of Prion Protein**

Baotong Tian, and Qiang Zhang

Department of Chemistry, State University of New York, University at Albany, Albany, USA

Protein misfolding and subsequent aggregation are the hallmarks of many neurogenerative diseases. Transmissible spongiform encephalopathies (TSEs, a.k.a. mad cow disease, or prion disease), are a class of infectious and fatal neurodegenerative disorders solely caused by the misfolding of prion protein. Structurally, prion protein is a membrane glycoprotein with two native N-glycosylation sites (Asn158, Asn174). Cellular prion protein (PrP^C^) is non-infectious, whereas misfolded cellular protein termed PrP*^Sc^*, which is prone to forming aggregates and is resistant to enzyme degradation. While significant attentions have been drawn to prion diseases research, many aspects of the molecular mechanism of prion transmission remain unclear. In the case of prion glycoprotein, one of the main obstacles is the inability to acquire high structurally defined prion strains due to the impact of their oligosaccharides. Prion protein strains exhibit not only different glycosylation patterns, but also differing in levels of glycosylation. Obtaining structurally defined protein entities for conformational-change interrogation is the foundation of reliable protein misfolding investigations. Abundant literature re-ports suggest that N-glycans that are conjugated to prion proteins, play critical roles in prion properties and pathogenic misfolding process. Furthermore, prion research is heavily reliant on high fidelity of prion conformations due to different strains exhibit distinct phenotypes. We engaged total chemical synthesis approach to obtain non-glycosylated and glycosylated prion.

**Material and Methods**: Protocols such as Native Chemical Ligation (NCL), Expressed Protein Ligation (EPL) and desulfurization approaches were applied towards the synthesis of PrP^C^.

**Results**: We have successfully prepared full construct of non-glycosylated prion protein via chemical methods. More specifically, β-thiolactone mediated sequential native chemical ligation tactics were employed for the synthesis. Currently spectra data has validated our results.

**Conclusions**: We present a novel chemistry method enabled prion preparation methods, which will provide a repertoire of highly desired prion proteins congeners that were previously inaccessible. The investigation of these synthetic agents will shift the paradigm of prion disease investigation. We believe that our studies will not only address the dire need for structurally well-defined substances needed for biological evaluations but will also serve as a cornerstone to facilitate the establishment of synergistic and collaborative research consortia across the chemical biology landscape, for the advancement of prion disease research.

**Grant number**: NIH R35 GM138336

**Acknowledgement**: National Institute of Health

**Arrayed CRISPR activation screen of the human transcription factors to identify modifiers of prion protein PrP^C^**

Trevisan C.^a^, Ging K. A.^a^, Frick L.^a^, Dhingra A.^b^, Avar M.^a^, Heinzer D.^a^, Heutink P.^b^, and Aguzzi A.^a^

^a^Institute of Neuropathology, University Hospital Zürich, Zürich, Switzerland; ^b^German Center for Neurodegenerative Diseases, Tübingen, Germany

**Aims**: The cellular prion protein (PrP^C^) is a cell-surface glycoprotein, responsible for the pathogenesis of prion diseases, fatal neurodegenerative disorders that affect humans and a large variety of animals.

The biosynthesis of PrP^C^is a prerequisite for PrP^Sc^formation, an abnormally folded isoform of the cellular prion protein which lead to a severe and progressive neuronal death through yet poorly defined pathways. Since the physiological role of PrP^C^is still not completely clear, different effort were performed in our group to find regulators of its expression through genome-wide screens. The following project aims to perform a CRISPR arrayed screen on a human glioblastoma cell line, using in-house human activation library. Such study will hopefully unravel new genes playing a role in PrP^C^activity and therefore it will contribute to broaden the spectrum of available pharmacological targets which can be considered for therapies against prion.

**Material and Methods**: We performed an arrayed CRISPRa screens, in a 384 well plate format, using the entire set of human transcription factors (1636 genes), packaged in lentiviruses. The advantage of activating the expression of the TFs with CRISPRa may exceed the limitation of previous study: indeed, through siRNAs and CRISPRi it was not possible to identify any transcription factors specifically controlling PrP^C^expression, perhaps because transcriptional gene regulation relies on redundant factors. A stable expressing dCas9-VPR human glioblastoma cell line (U251-MG) was used as a cellular model. Time-resolved fluorescence resonance energy transfer (TR-FRET) detected endogenous PrP^C^expression in cell lysate. We used Europium (EU)-conjugated-POM2 and allophycocyanin (APC)-labelled- POM1, a pair of antibodies binding distinct domains of PrP^C^.

**Results**: The identification of transcription factors regulating PrP^C^expression is ongoing. Hit calling was based on an absolute log_2_ fold change of ≥1 and a p-value of ≤ 0.05. Based on these cut- offs, 24 and 12 genes out of 1634 were found to upregulate or downregulate PrP^C^expression, respectively. The validation of the candidate genes, to exclude cell-line specific hits, will be performed on a second cell line, possibly on iPSC-derived neurons.

**Conclusions**: The only strong genetic risk factor for prion disease has remained PRNP, the human gene encoding for the prion protein. Thanks to the recent generation of the human CRISPRa arrayed libraries in our lab, it will be possible to search for other novel genes driving PrP^C^expression. This is the fundamental reason why the proposed work focuses on a genetic screen: the founding of new modifiers could provide unexpected regulatory pathway which involve PrP^C^expression.

**Funded by**: Swiss Personalized Health Network (SPHN)

**Grant number**: 2017DRI17

**Acknowledgement**: Yin Jiang-An

**Prion conformer-dependent Chaperone interactions in a chaperonopathy**

Heather True, Ankan Bhadra, Kevin Stein, and Chris Weihl

Washington University, St. Louis, MO, USA

**Aims**: Protein chaperones are essential to maintain cellular protein homeostasis. Mutations in the Hsp40 DNAJB6 cause a degenerative muscular disease (Limb-Girdle Muscular Dystrophy (LGMDD1)), which exhibits an accumulation of protein aggregates and vacuoles in skeletal muscle. We have now identified mutations in two domains of DNAJB6 that cause LGMDD1. We aim to test our central hypothesis that LGMDD1 mutations alter the conformation of DNAJB6 such that the interface between these two domains is disrupted, leading to inefficient processing of certain conformers of misfolded proteins. This results in protein aggregates, myofibril disorganization, and muscle dysfunction. Furthermore, using our yeast prion system, we aim to identify viable therapeutic candidates to treat this degenerative disease.

**Material and Methods**: In order to tackle this challenging problem, we will explore DNAJB6 chaperone function and dysfunction utilizing multiple systems. We have investigated DNAJB6 dysfunction using yeast, in vitro, and animal models. We will determine the effect of LGMDD1 mutations on chaperone and co-chaperone function in vivo and in vitro. Our yeast system has also provided key information from second-site suppressor screens and the discovery of mutants that rescue prion propagation defects in cells expressing LGMDD1 mutants.

**Results**: We found that homologous LGMDD1 mutations in Sis1 impair its function in recognizing and modulating the aggregated state of select yeast prion strains. These data suggest that mutations in DNAJB6 may abrogate its chaperone activity toward select aggregate conformers of the same aggregated protein. We found that manipulating interactions between DNAJB6 and HSP70 rescues LGMDD1 phenotypes in yeast, mouse models, and human cells. We found that LGMDD1 mutants impair most aspects of Hsp40 function, and the client conformer-specific effects were also evident in vitro. We also found that these mutants alter the phenotypic distribution of prion protein conformers. Finally, by using prion and non-prion substrates, we found that modulation of nucleotide exchange factors rescues the client-conformer processing defects of LGMDD1 mutants.

**Conclusions**: Based on our results, we hypothesize that all LGMDD1 mutations in DNAJB6 can be rescued by perturbation of Hsp70 interaction or activity. Our data suggest that we have identified additional therapeutic targets (NEFs) that may be better tolerated in patients than global inhibitors of Hsp70. The overarching goal of this work is to leverage our transdisciplinary success to develop therapeutic interventions for patients with LGMDD1. Additionally, our results highlight the importance of assessing multiple prion conformers when investigating putative interacting proteins.

**Funded by: NIH**

**Grant number:R01 AR068797**

**Prion disease features in Japan according to the national surveillance from 1999 to 2022**

Tadashi Tsukamoto^a^, Ryuusuke Ae^b^, Tsuyoshi Hamaguchi^c^, Nobuo Sanjo^d^, Yoshikazu Nakamura^b^, Katsuya Satoh^e^, Tetsuyuki Kitamoto^f^, Masaki Takaog, Masahito Yamada^c^,^h^, and Hidehiro Mizusawa^a^ and Prion Diseases Surveillance Committee in Japan

^a^Department of Neurology, National Center Hospital, National Center of Neurology and Psychiatry, Tokyo, Japan; ^b^Department of Public Health, Jichi Medical University, Shimotsuke, Japan; ^c^Department of Neurology and Neurobiology of Aging, Kanazawa University Graduate School of Medical Science, Kanazawa, Japan; ^d^Department of Neurology and Neurological Science, Graduate School, Tokyo Medical and Dental University, Tokyo, Japan;^e^Department of Locomotive Rehabilitation Sciences, Nagasaki University Graduate School of Medicine, Nagasaki, Japan; ^f^Department of Prion Protein Research, Division of CJD Science and Technology, Tohoku University Graduate School of Medicine, Sendai, Japan; gDepartment of Clinical Laboratory, National Center of Neurology and Psychiatry, National Center Hospital, Tokyo, Japan; ^h^Division of Neurology, Department of Internal Medicine, Kudanzaka Hospital, Tokyo, Japan

**Aims**: The outbreak of variant CJD (vCJD) in UK since 1996 led to 2 urgent surveys in Japan and the establishment of a national prion disease surveillance system in 1999. The aim of this report is to clarify characteristics of prion disease (PrD) in Japan according to the surveillance from 1999 to 2022, which include dura mater-associated CJD (dCJD) and genetic PrDs unique to Japan.

**Material and Methods**: We divided Japan into 10 districts and assigned a surveillance committee member to each district. The surveillance committee composed of district members and specialists of MRI, EEG, genetic analysis, CSF analysis, neuropathology, neurosurgery and psychological counseling. The committee members review each suspected PrD case twice a year based on information from the doctors in 10 districts as well as the results of genetic and CSF analyses. Patient information is recorded in a database, and the results of the analysis are published on the website (http://prion.umin.jp/survey/survey.html).

**Results**: We obtained information on 6094 patients who were suspected of having PrDs up to Feb.2022. Among them, 4166 patients were confirmed as PrDs. The details of these patients included 3167 cases of sporadic CJD, one case of variant CJD, 93 cases of dCJD, and 888 cases of genetic PrD (726 cases of genetic CJD (gCJD), 158 cases of Gerstmann-Sträussler-Scheinker disease, and 4 cases of fatal familial insomnia). The apparent annual incidence of PrDs has gradually increased from 0.7 per million in 1999 to 2.3 in 2015. Genetic analysis of the prion protein gene of all the PrD patients agreed with the test showed that the ratio of codon 129 polymorphism methionine/methionine to methionine/valine and valine/valine was very high (in sCJD, MM:MV:VV = 95:4.1; in control, MM:MV:VV = 94:7:1,) in Japan as compared with those in Western countries. This difference has not changed since we reported it in 2010. The number of dCJD was a total of 156 cases from the first urgent survey before 1999. Among 757 genetic PrDs in Japan, gCJD was found in 18%, GSS in 4%, and FFI in only 4 cases. Among gCJD, V180I accounted for 64%, E200K for 15%, and M232R for 15%.

**Conclusions**: During the 20 years since 1999, the incidence of PrD in Japan has continued to increase. This increase can be attributed to the improvement of PrD diagnostic techniques and the spread of medical knowledge, in addition to the fact that Japan has become a hyper-aged society.

**Funded by**: 1) The Research Committee of Surveillance and Infection Control of Prion Disease, the Ministry of Health, Labour, and Welfare of Japan

2) The Research Committee of Prion Disease and Slow Virus Infection, Research on Policy Planning and Evaluation for Rare and Intractable Diseases, Health and Labour Sciences Research Grants,

**Grant number**: 1) 22FC2002; 2) 20FC1054

**Acknowledgment**: We are grateful to the prion disease specialists in the prefectures, clinical physicians, the patients with prion disease and their families for providing clinical information about the patients.

**An optimized western blot method for the analysis of PrP^C^endoproteolytic cleavages**

I. Vanni^a^, F. Iacobone^a^, C. D’Agostino^a^, M. Giovannelli^a^, L. Pirisinu^a^, H.C. Altmeppen^b^, J. Castilla^c,d^, J.M. Torres^e^, U. Agrimi^a^, and R. Nonno^a^

^a^Department of Food Safety, Nutrition and Veterinary Public Health, Istituto Superiore di Sanità, Rome, Italy; ^b^Institute of Neuropathology, University Medical Center Hamburg-Eppendorf, Martinistraße 52, Hamburg, Germany; ^c^Prion Research Lab, Basque Research and Technology Alliance (BRTA), Center for Cooperative Research in Biosciences (CIC BioGUNE), Derio, Spain; ^d^Centro de Investigación Biomédica en Red de Enfermedades infecciosas (CIBERINFEC), Instituto de Salud Carlos III, Madrid, Spain; ^e^Centro de Investigación en Sanidad Animal (CISA-INIA), Valdeolmos, Madrid, Spain

**Aims**: PrP^C^and its endoproteolytic fragments are key in the pathology of prion and other neurodegenerative diseases. Despite the number of studies on proteolytic processing of PrP^C^, western blotting (WB) remains the most-widely used technique for the analysis of PrP^C^constitutive cleavages. However, WB methods allowing a reliable identification and quantification of all PrP fragments are not available. We aimed at exploring the potential of WB-based approaches to fill this gap.

**Material and Methods**: Brain tissues from bank voles, wild-type (wt) mice and transgenic mice expressing sheep, bovine and human PrP were PNGase F-treated or left untreated, subjected to WB and analysed by extensive epitope mapping. Antibodies were selected so to have conserved epitopes among the models used and to cover the entire sequence of PrP. Inhibitors of endogenous proteases were added to avoid the generation of non-specific PrP-derived fragments and electrophoresis was conducted under strictly reduced conditions to obtain a high discrimination of fragments with similar molecular mass.

**Results**: The optimized WB method allowed detecting all PrP^C^fragments derived from alpha-, beta-, gamma-cleavages and shedding. Deglycosylation was key to identify and reliably quantify full-length PrP^C^(FL-PrP^C^), its C-terminal fragments C1 and C2 as well as their N-terminal counterparts and shed PrP isoforms, with shed FL-PrP^C^ clearly distinguishable from FL-PrP^C^ independently from the antibody used. PrP fragments generated by gamma-cleavage were detected but not amenable to quantification due to their low amount. By assessing the relative quantity of the most represented PrP^C^fragments, we found similar PrP^C^fragment patterns in wt and transgenic models, with C1 representing the most abundant PrP fragment, followed by FL-PrP^C^, C2, shed PrP, N1 and N2. PrP^C^shedding was a prominent proteolytic event in the brain of all the models analysed, accounting for 7–10% of total PrP^C^and for up to 30% of total FL-PrP^C^. The relative quantity of shed FL-PrP^C^ over FL-PrP^C^ was amenable to quantitative assessment, showing higher scores in wt mice than in most other models.

**Conclusions**: We provide a reliable method for the analysis of PrP^C^and its endoproteolytic cleavages. As far as we know, this is the first available WB tool that identifies, differentiates from its GPI-anchored counterpart and reliably quantifies shed PrP^C^in biological tissues using pan-PrP antibodies. Our results also show that PrP^C^processing is not appreciably affected by the PrP expression level or the species genetic background but can be impacted by the PrP sequence.

**Funded by**: Ministero della salute

**Grant number**: RF-2016-02364498

***Bona fide* spontaneous and atypical scrapie faithfully reproduced through the expression of a polymorphic variant of ovine prion protein**

Enric Vidal^a,b^, Manuel A. Sanchez-Martín^c^, Hasier Eraña^f^, Sonia Pérez Lázaro^d,e,o^, Miguel A. Pérez-Castro^d^, Alicia Otero^f^, Jorge M. Charco^d,e,o^, Belén Marín^f^, Rafael López-Moreno^d^, Carlos M Díaz-Domínguez^d^, Mariví Geijo^g^, Montserrat Ordóñez^a,b^, Michele di Bari^h^, Nuria L. Lorenzo^m^, Laura Pirisinu^h^, Claudia D’Agostino^h^, Juan María Torres^i^, Vincent Béringue^j^, Juan J. Badiola^f^, Glenn Telling^k^, Martí Pumarola^l^, Rosa Bolea^f^, Romolo Nonno^h^, Jesús R. Requena^m^ and Joaquín Castilla^d,e,n,o^

^a^Unitat mixta d’Investigació IRTA-UAB en Sanitat Animal. Centre de Recerca en Sanitat Animal (CReSA), Campus de la Universitat Autònoma de Barcelona (UAB), Bellaterra, Catalonia; ^b^IRTA. Programa de Sanitat Animal. Centre de Recerca en Sanitat Animal (CReSA). Campus de la Universitat Autònoma de Barcelona (UAB), Bellaterra, Catalonia.^c^Transgenic Facility. Department of Medicine. University of Salamanca, Salamanca, Spain; ^d^Center for Cooperative Research in Biosciences (CIC BioGUNE), Basque Research and Technology Alliance (BRTA), Prion Research Lab, Derio, Spain; ^e^ATLAS Molecular Pharma S. L. Derio (Bizkaia), Spain.; ^f^Centro de Encefalopatías y Enfermedades Transmisibles Emergentes, Facultad de Veterinaria, Universidad de Zaragoza – IA2, Zaragoza, Spain; ^g^Animal Health Department, NEIKER-Basque Institute for Agricultural Research and Development, Basque Research and Technology Alliance (BRTA), Parque Científico y Tecnológico de Bizkaia, Derio, Spain; ^h^Istituto Superiore di Sanità, Department of Food Safety, Nutrition and Veterinary Public Health, Rome, Italy; ^i^Centro de Investigación en Sanidad Animal, CISA-INIA-CSIC, Valdeolmos, Madrid, Spain; ^j^Molecular Virology and Immunology, French National Research Institute for Agriculture, Food and Environment (INRAE), Université Paris-Saclay, Jouy-en-Josas, France; ^k^Prion Research Center (PRC) and the Department of Microbiology, Immunology, and Pathology, Colorado State University, Fort Collins, Colorado; ^l^Departament de Medicina i Cirurgia Animals, Facultat de Veterinària, Campus de UAB, Bellaterra, Barcelona, Catalonia; ^m^CIMUS Biomedical Research Institute, University of Santiago de Compostela-IDIS, Spain; ^n^IKERBASQUE, Basque Foundation for Science, Bilbao, Bizkaia, Spain; ^o^Centro de Investigación Biomédica en Red de Enfermedades infecciosas, Carlos III National Health Institute, Madrid, Spain

**Aims**: Atypical scrapie, which is not linked to epizootics, appears to be the only truly sporadic prion disease in small ruminants. Therefore, its occurrence is unlikely to be controlled through selective breeding or other strategies in place for classical scrapie outbreaks. Its spontaneous nature and its sporadic emergence worldwide are reminiscent of the occurrence of sporadic prion diseases in humans, accounting for more than 85% of the cases. Hence, the development of animal models that consistently reproduce this phenomenon of spontaneous PrP misfolding, is greatly needed to study the pathobiology of sporadic prion disorders.

**Material and Methods**: Transgenic mice overexpressing sheep PrP^C^with I112 polymorphism (TgShI112, 1–2x PrP levels compared to sheep brain) manifest clinical signs of a spongiform encephalopathy spontaneously at 380 days of age. The brains of these animals show the pathological hallmarks of prion disease and biochemical analyses of the misfolded prion protein show a ladder-like PrP^res^pattern with a predominant 7–10 kDa band.

Brain homogenates from spontaneously diseased TgShI112 transgenic mice were inoculated in several models to assess their transmissibility and characterize the prion strain generated: TgShI112 (ovine I112 ARQ PrP^C^), Tg338 (ovine VRQ PrP^C^), Tg501 (ovine ARQ PrP^C^), TgVole (bank vole I109 PrP^C^), bank vole (I109I PrP^C^), and Churra-tensina breed sheep (AHQ/AHQ and AHQ/ARR).

**Results**: The results of the aforementioned bioassays are discussed, concluding that the prion strain generated spontaneously in this model is indistinguishable from that causing atypical scrapie (Nor98).

**Conclusions**: We present the first faithful model for a *bona fide*, spontaneous, transmissible, small ruminant, atypical prion disease.

**Funded by:/Grant number**: This study was funded by MINECO research project references AGL2013-46,756-P and RTI2018-098515-B-I00, by RedPRION (Interreg POCTEFA EFA148/16) and by Fundació la Marató de TV3 ATYPRION (201,821–30-31-32).

**ATYPRION project: assessing the zoonotic potential of interspecies transmission of CWD isolates to livestock (preliminary results).**

Enric Vidal^a,b^, Juan Carlos Espinosa^c^, Samanta Giler^a,b^, Montserrat Ordóñez^a,b^, Guillermo Cantero^a,b^, Vincent Béringue^d^, Justin J. Greenlee^e^, and Juan Maria Torres^c^

^a^Unitat mixta d’Investigació IRTA-UAB en Sanitat Animal. Centre de Recerca en Sanitat Animal (CReSA). Campus de la Universitat Autònoma de Barcelona (UAB), Bellaterra, Catalonia; ^b^IRTA. Programa de Sanitat Animal. Centre de Recerca en Sanitat Animal (CReSA). Campus de la Universitat Autònoma de Barcelona (UAB), Bellaterra, Catalonia; ^c^Centro de Investigación en Sanidad Animal, CISA-INIA-CSIC, Valdeolmos, Madrid, Spain; ^d^Molecular Virology and Immunology, French National Research Institute for Agriculture, Food and Environment (INRAE), Université Paris-Saclay, Jouy-en-Josas, France; ^e^Virus and Prion Research Unit, National Animal Disease Center, ARS, United States Department of Agriculture, Ames, IA, USA

**Aims**: Since variant Creutzfeldt-Jackob disease was linked to the consumption of bovine spongiform encephalopathy prions, the study of the pathobiological features of animal prions, particularly their zoonotic potential, is of great concern to the scientific community and public health authorities. Furthermore, interspecies transmission of prions has been demonstrated as a putative evolutionary mechanism for prions, that can lead to the emergence of new features including the ability to infect humans. For instance, small ruminants’ atypical scrapie prions, when propagated in a bovine or porcine host, can shift to a classical BSE phenotype thus posing a potential risk in case of human exposure. So far, no hard evidence of zoonotic transmission of cervids’ chronic wasting disease (CWD) to humans has been published, however experimental transmission to bovine, ovine and caprine hosts has been achieved. Our goal is to investigate if, once passaged through these domestic species, CWD prions might become infectious to humans.

**Material and Methods**: Different CWD isolates experimentally adapted to cattle, sheep and goat (Hamir et al, 2005, 2006, 2007, Greenlee et al 2012) have been intracerebrally inoculated to transgenic mouse models expressing the human cellular prion protein either homozygous for methionine or valine at codon 129 (Tg340-Met129 and Tg362-Val129). Additionally, inocula obtained from experimental transmission of elk CWD to ovinized (Tg501) and bovinized (BoTg110) transgenic mice, as well as white-tailed deer CWD to BoTg110 mice, are currently being bioassayed in both human PrP^C^transgenic models.

**Results and conclusions**: No evidence of transmission has been found on first passage for bovine adapted elk and mule deer CWD to none of the humanized models. The remaining bioassays are ongoing without showing clinical signs yet, as well as second passages for the negative 1^st^passages.

**Funded by**: *La Marató de TV3* foundation.

**Grant number**: ATYPRION (201,821–30-31-32)

**Transmission properties of 129 MV vCJD prions in humanized transgenic mice**

Jonathan DF Wadsworth, and John Collinge

MRC Prion Unit at UCL, Institute of Prion Diseases, University College London,London W1W 7FF, UK

**Aims**: In 2016 we identified the first definite variant Creutzfeldt-Jakob disease (vCJD) patient with a codon 129 MV genotype (Mok *et al*. 2017; *N. Engl. J. Med*. 376, 292–294). This case provided the first opportunity to study the distribution and strain properties of prions propagated in brain and lymphoreticular tissues. Using biochemical analyses of patient tissues and transmission studies to humanized transgenic mice and to wild-type mice we aimed to address the following key questions. (1) whether 129 MV vCJD has a peripheral pathogenesis similar to 129 MM vCJD and (2) whether there is co-propagation of vCJD prions with sporadic CJD-like or novel prions strains in 129 MV vCJD patient brain or lymphoreticular tissues.

**Material and Methods**: We prepared multiple homogenates from autopsy brain and lymphoreticular tissues from the 129 MV vCJD patient and biochemically characterised these to determine the presence of protease-resistant disease-related prion protein. Homogenates from four brain regions and from appendix, mesenteric lymph nodes and spleen were inoculated intracerebrally into transgenic mice expressing human prion protein with codon 129 MM, VV and MV genotypes on a congenic mouse prion protein null background and into wild-type FVB/N mice.

**Results**: Biochemical and immunohistochemical analyses of mouse brains from primary and secondary transmissions of 129 MV vCJD patient tissues are now largely complete enabling comparison with findings from our historical transmission series of 129 MM vCJD prions to the same mice.

**Conclusions**: Caution must be exercised when extrapolating findings from a single 129 MV vCJD patient. Nevertheless this first case appears to demonstrate that 129 MV vCJD has prion transmission properties that are distinct from 129 MM vCJD.

**Funded by**: This research was funded by the UK National Institute for Health Research (NIHR) Policy Research Programme (project reference PR-R17-0916-23,002). The views expressed are those of the authors and not necessarily those of the NIHR or the Department of Health and Social Care.

**Grant number**: PR-R17-0916-23,002

**Acknowledgements**: This research would not have been possible without the support of patients and their families and we are extremely grateful to them for their consent to use human tissues in this research. All experimental protocols were approved by the Local Research Ethics Committee of UCL Queen Square Institute of Neurology/National Hospital for Neurology and Neurosurgery.

**Strain Types for Chronic Wasting Disease and Effort Towards a Virtual Tissue Repository**

W. David Walter^a^, Chia-Hua Lue^b^, and Jason Bartz^c^

^a^U.S. Geological Survey, Pennsylvania Cooperative Fish and Wildlife Research Unit, The Pennsylvania State University, University Park, PA; ^b^Pennsylvania Cooperative Fish and Wildlife Research Unit, The Pennsylvania State University, University Park, PA; ^c^Medical Microbiology and Immunology, Creighton University, Omaha, NE

**Aims**: Chronic wasting disease (CWD) is a prion disease that affects Cervidae species and is a prominent transmissible spongiform encephalopathy (TSE) in wildlife. Since CWD was first identified in the 1960s in the United States, it has been documented in 29 states in captive and wild cervids, along with being present in Canada, South Korea, and Scandinavia. When normal cellular prion protein (PrP^c^) misfolds to an abnormal conformation (PrP^sc^) it causes CWD infections and different conformations of PrP^sc^result in various disease phenotypes that characterize different prion strains. There is a need to summarize the known CWD strains and to expand our general knowledge of the CWD strains. One of the main obstacles in CWD research, however, is a lack of a centralized collection of wild CWD-positive tissues. We will attempt to overcome this obstacle by introducing a national repository for CWD.

**Material and Methods**: Advancements in Real-Time Quaking-Induced Conversion (RT-QuIC) assays and strain-specific biochemical and biological properties of CWD prions, have provided a unique opportunity to explore prion strains in wild cervids. We summarized this literature to identify the current state of knowledge of prion strains in various cervid species. We then used this information to design a virtual repository of tissue and reagents for chronic wasting disease. The virtual repository will be housed on a remote server that can be maintained by a representative organization for future generations. This virtual repository will be accessible by researchers throughout the world to request samples to achieve study objectives involving prion strain type and prion protein genotypes.

**Results**: We are soliciting cooperating researchers and organizations for tissues collected on a large geographic scale in North America to assess the distribution, frequency, and strain types to their point of origin. Important metadata (coordinates for collection location, species, tissue type (e.g. retropharyngeal lymph node, obex)) will be linked to each tissue type. The virtual repository provides maps of locations of standardized CWD-infected and uninfected tissue resources and polymorphisms of the host prion protein gene for each tissue available.

**Conclusions**: Availability of a virtual repository of this nature will serve as a centralized platform to facilitate cooperation and the sharing of resources between state agencies and research institutes. Monitoring these data over time will provide important information regarding CWD strain dynamics and will allow for identification of novel emerging strains that may have altered pathogenicity and/or zoonotic potential compared to currently circulating CWD strains.

**Funded by**: U.S. Geological Survey, Ecosystems Mission Area, Disease Cyclical Funds

**Grant number**: GRANT13389923

**Acknowledgement**: North American Interdisciplinary Chronic Wasting Disease Consortium, U.S. Department of Agriculture, NC1209

**Faithful propagation of prion strain-specific conformation to recombinant protein**

Fei Wang, Andrew Scowcroft, Rodrigo Diaz-Espinoza, Luis Concha-Marambio, Damian Gorski, Sandra Pritzkow, and Claudio Soto

Mitchell Center for Alzheimer’s Disease and Related Brain Disorders, Department of Neurology, McGovern Medical School, University of Texas Health Science Center at Houston, Houston, Texas, USA

**Aims**: Prions are self-propagating proteinaceous agents that cause fatal neurological disorders called Transmissible Spongiform Encephalopathies (TSEs). Prions replicate through the template-guided conversion of the normally folded prion protein (PrP^C^) by its misfolded, infectious form (PrP^Sc^). Different prion strains composed of PrP^Sc^sharing identical primary sequences are known to exhibit distinguishable molecular properties and pathologies. Using the PMCA (Protein Misfolding Cyclic Amplification) technique, it has been reported that bona fide recombinant prions can be generated with bacterially expressed recombinant PrP (recPrP) and specific cofactors, either spontaneously or through seeded reactions. However, previous efforts to faithfully propagate native prion strain properties using recPrP as a substrate have failed. Here, we studied whether recPrP can serve as a substrate for efficient and faithful *in vitro* propagations of the RML prion by maintaining the strain-specific properties.

**Material and Methods**: In a seeded PMCA reaction supplemented with anionic cofactors, 10% brain homogenate (BH) from a terminally ill RML-infected mouse was used as the template to amplify the protease-resistant PrP species (PrP-res) from the protease-sensitive recPrP. Wild-type mice were intracerebrally inoculated with 10% RML BH or the newly generated recPrP-res and monitored for clinical signs of prion disease. At the terminal stage, diseased animals were euthanized and brain tissues were collected and used for the subsequent passaging in wild-type animals. Brain tissues from all animals were subjected to an array of biochemical and histopathological analyses for prion strain characterization.

**Results**: Wild-type mice infected with RML-seeded recPrP-res and animals of the subsequent passage developed classical prion clinical signs with a very synchronized incubation time, similar to that of the RML-infected mice. Further biochemical and histopathological analyses confirmed that these animals succumbed to the RML prion disease.

**Conclusions**: As a proof of concept, our results support the notion that PMCA can faithfully propagate prion conformations to recombinant proteins under defined conditions and in the absence of brain-derived components.

**Funded by**: National Institute of Health

**Grant number**: P01 AI077774

**Loss of homeostatic microglia in prion diseases**

Yue Wang*, Daniela Waddell*, Kristin Hartmann, Edda Thies, Diego Sepulveda-Falla, Markus Glatzel, and Susanne Krasemann

*contributed equally

Institute of Neuropathology, University Medical Center Hamburg-Eppendorf, Hamburg, Germany

Microglia are the innate immune cells of the brain. Microglia can be both protective and detrimental, and understanding the physiological functions of these cells is crucial to determining their roles in disease. Activated microglia represent a common pathological feature of neurodegenerative diseases. While the role of activated microglia have been studied in prion disease mouse models, less is known about the dysregulation of homeostatic signature of microglia in disease. Moreover, the role of microglia in human and primate prion disease have not been thoroughly investigated so far.

**Material and Methods**: To determine the shift in the microglia profile, we used immunohistochemical detection of pan microglia/monocyte, homeostatic microglia, and disease marker in brains of mice experimentally infected with RML5.0 prions. Moreover, we investigated in a primate model the microglia signature at different pre-clinical time points and terminal disease. The latter data will be compared to the situation in the human brain at terminal prion disease and compared to the microglia found in brains of patients with Alzheimer’s disease.

**Results**: Microglia get activated during the course of a prion disease. This is accompanied by an increase in microglia number and a change of the morphologic appearance from ramified to amoeboid. The degree of microglia dysregulation correlate with brain areas displaying deposition of misfolded PrP^Sc^. Whereas we could not detect Clec7a, a microglia disease marker that is upregulated in mouse models of Alzheimer’s disease, the homeostatic microglia marker TMEM119 was significantly reduced in terminal disease. The loss of homeostatic microglia marker could also be detected in the primate model and in human prion disease. Interestingly, upregulation of the activated microglia marker CD68 is more prominent in Prion disease in contrast to Alzheimer’s disease.

**Conclusions**: Microglia are highly dysregulated in prion diseases of animals and humans and show a significant loss of their homeostatic signature. On the other hand, they display marker of activation that are distinct from those in Alzheimer’s disease. Although microglia are commonly dysregulated in neurodegenerative disease, they differ in their dysregulation degree and profile.

**Generation of human chronic wasting disease in transgenic mice**

Zerui Wang^a^, Kefeng Qin^b^, Manuel V. Camacho^a^, Ignazio Cali ^a,c^, Jue Yuan^a^, Pingping Shen^a^, Tricia Gilliland^a^, Syed Zahid Ali Shah^a^, Maria Gerasimenko^a^, Michelle Tang^a^, Sarada Rajamanickam^a^, Anika Yadati^a^, Lawrence B. Schonberger^d^, Justin Greenlee^e^, Qingzhong Kong^a,c^, James A. Mastrianni^b^, and Wen-Quan Zou^a,c^

^a^Department of Pathology, Case Western Reserve University School of Medicine, Cleveland, OH, USA; ^b^Department of Neurology and Center for Comprehensive Care and Research on Memory Disorders, the University of Chicago Pritzker School of Medicine, Chicago, USA; ^c^National Prion Disease Pathology Surveillance Center, Case Western Reserve University School of Medicine, Cleveland, OH 44106, USA; ^d^Division of High-Consequence Pathogens and Pathology, Centers for Disease Control and Prevention, 1600 Clifton Rd, Atlanta, GA, USA; ^e^Virus and Prion Research Unit, National Animal Disease Center, USDA, Agricultural Research Service, 1920 Dayton Avenue, Ames, IA, USA

**Aims**: Chronic wasting disease (CWD) results from the accumulation of an infectious misfolded conformer (PrP^Sc^) of cellular prion protein (PrP^C^) in the brains of deer and elk. It has been spreading rapidly throughout many regions of North America, exported inadvertently to South Korea, and more recently identified in Europe. Mad cow disease has caused variant Creutzfeldt-Jakob disease (vCJD) in humans and is currently the only known zoonotic prion disease. Whether CWD is transmissible to humans remains uncertain. The aims of our study were not only to confirm whether CWD prion isolates can convert human brain PrP^C^into PrP^Sc^*in vitro* by serial protein misfolding cyclic amplification (sPMCA) but also to determine whether the sPMCA-induced CWD-derived human PrP^Sc^is infectious.

**Material and Methods**: Eight CWD prion isolates from 7 elks and 1 deer were used as the seeds while normal human brain homogenates containing either PrP-129 MM (n = 2) or PrP-129 VV (n = 1) were used as the substrates for sPMCA assay. A normal elk brain tissue sample was used as a negative control seed. Two lines of humanized transgenic (Tg) mice expressing either human PrP-129VV or −129 MM polymorphism were included for transmission studies to determine the infectivity of PMCA-amplified PrP^Sc^. Wester blotting and immunohistochemistry and hematoxylin & eosin staining were used for determining PrP^Sc^and neuropathological changes of inoculated animals.

**Results**: We report here the generation of the first CWD-derived infectious human PrP^Sc^using elk CWD PrP^Sc^to initiate conversion of human PrP^C^from normal human brain homogenates with PMCA *in vitro*. Western blotting with a human PrP selective antibody confirmed that the PMCA-generated protease-resistant PrP^Sc^was derived from the human brain PrP^C^substrate. Two lines of humanized transgenic mice expressing human PrP^C^with either Val or Met at the polymorphic codon 129 developed clinical prion disease following intracerebral inoculation with the PMCA-generated CWD-derived human PrP^Sc^. Diseased mice exhibited distinct PrP^Sc^patterns and neuropathological changes in the brain.

**Conclusions**: Our study, using PMCA and animal bioassays, provides the first evidence that CWD PrP^Sc^has the potential to overcome the species barrier and directly convert human PrP^C^into infectious PrP^Sc^that can produce bona fide prion disease when inoculated into humanized transgenic mice.

**Funded by**: CJD Foundation and NIH

**Anti-prion systems in yeast cooperate to cure or prevent the generation of nearly all variants of the [PSI+] and [URE3] prions in normal cells**

Reed B. Wickner, Moonil Son, Herman Edskes, Songsong Wu, and Madaleine Niznikiewicz

Laboratory of Biochemstry and Genetics, National Institute of Diabetes and Digestive and Kidney Diseases, National Institutes of Health, Bethesda, MD, USA

**Aims**: Human prion and amyloid diseases are largely untreatable. We previously found anti-prion systems in yeast whose human analogs or homologs might be manipulated to treat these conditions in the same way that humoral, cellular and innate immunity is used to treat or prevent viral and bacterial infections. Here we examine the interactions of these systems and characterize the prion variants whose propagation or generation they block.

**Material and Methods**: We constructed all possible combinations of mutations in five anti-prion genes (*ssz1*Δ, *upf1*Δ, *btn2*Δ, *cur1*Δ and *hsp104^T160M^*) active against [PSI+] (prion of Sup35p) or [URE3] (prion of Ure2p). We measured the frequency of [PSI+] prion generation (spontaneous or induced by prion domain overexpression), and determine which anti-prion system(s) cure which prion variants that arise in the multiple mutants.

**Results**: The *ssz1*Δ, *upf1*Δ, and *hsp104^T160M^* synergistically elevated the spontaneous frequency of [PSI+] arising so that the triple mutant has up to a 5000-fold increase above wild type strains. [PSI+] arising in the triple mutant include those curable by normal levels of any of the three missing proteins, those needing one specific anti-prion to be cured, and those not cured in normal cells at all, but arising at 25-fold or high frequency compared to normal cells. The absence of Btn2 (a ‘sequestrase’ that collects prion amyloids and other denatured proteins) actually decreases the frequency of [PSI+], but increases the frequency of [URE3]. However, *btn2*Δ does not affect the frequency of [PSI+] appearance in *ssz1*Δ *upf1*Δ *hsp104^T160M^* triple mutant strains.

**Conclusions**: Prion formation is not a rare event. Instead, prions are quite common, but yeast has multiple systems that block prion formation, cure the overwhelming majority of prions formed, limit the infection by prions from another cell and partially block the lethality of those prions that escape the other anti-prion systems.

**Funded by**: The Intramural Program of the National Institute of Diabetes and Digestive and Kidney Diseases of the National Institutes of Health

**Grant number**: DK024950

**Acknowledgement**: This work was supported by the Intramural Program of the National Institute of Diabetes and Digestive and Kidney Diseases of the National Institutes of Health.

**PrP shedding from mast cells is dependent upon proteases released during degranulation**

Steven D. Willows^a^, and Marianna Kulka^a,b^

^a^Nanotechnology Research Centre, National Research Council Canada, Edmonton, Canada; ^b^Department of Medical Microbiology and Immunology, University of Alberta, Edmonton, Canada

**Aims**: The prion protein (PrP) is best known for its role in several infectious prion diseases in both humans and animals. The physiological role of PrP is still under debate, especially its role on several types of immune cells where it is highly expressed. Our aim was to better understand the expression of PrP on granulated mast cells that store and release specific proteases upon activation. Mast cells are considered some of the first responders in the immune system and play a significant role in allergy, detoxification of venoms/toxins, as well as response to infection, tissue remodeling and neuroplasticity. Upon stimulation, mast cells release pre-stored granules containing several signaling molecules and proteases. Although mouse mast cells have previously been shown to release PrP after degranulation, the mechanism has never been elucidated. We therefore sought to investigate if human mast cells also release PrP and to better understand the mechanism by which PrP is released from mast cells.

**Material and Methods**: Mouse bone marrow mast cells (BMMC) and the human mast cell line LAD2 were used in all experiments. To induce degranulation, BMMC were activated by calcium ionophore A23187 or IgE + antigen. LAD2 were degranulated by stimulating with compound 48/80 (C4880) or IgE + anti-IgE. Protease inhibitors, including complete protease inhibitor cocktail (CPI) or 4-(2-aminoethyl)benzenesulfonyl fluoride hydrochloride (AEBSF), were included just before addition of degranulating reagents. LAD2 were treated with the mast cell-specific protease, recombinant tryptase, to determine its autocrine effect on PrP expression. Cells were incubated for 30–90 minutes at 37°C before fixation and levels of PrP were determined by flow cytometry.

**Results**: Degranulation reduced the amount of PrP on mast cells by 50–80% depending on the stimulus. PrP levels recovered partially by three hours but took 48 hours to regain the same expression levels as untreated. Both CPI and AEBSF abrogated the amount of PrP lost after degranulation. Exposure of LAD2 cells to recombinant tryptase reduced the amount of PrP on cells by 36%.

**Conclusions**: Degranulation resulted in decreased PrP expression on both human and mouse mast cells regardless of the stimulus. The ability of protease inhibitors to inhibit this decrease suggests that this is a protease-dependent mechanism. The ability of recombinant tryptase to decrease PrP levels on cells suggests that this enzyme may be at least partly responsible for this loss of PrP expression on the cell surface.

**Funded by: National Research Council Canada**

**A case of probable Creutzfeldt-Jakob disease with the PrP G114V mutation**

Otto Windl^a^, Monika Empl^b^, Selamawit Gebrekidan^c^, Peter Bartenstein^c^, Inga Zerr^d^, Jochen Herms^a^, and Adrian Danek^b^

^a^Center for Neuropathology and Prion Research, LMU Munich, Munich, Germany; ^b^Department of Neurology, University Hospital, LMU Munich, Munich, Germany; ^c^Department of Nuclear Medicine, University Hospital, LMU Munich, Germany; ^d^Prion Research Group, Department of Neurology, University Hospital, GAU Göttingen, Göttingen, Germany

At 21 years of age this patient presented with a range of neurological signs and developed rapidly progressive dementia and myoclonus. Various diagnoses were considered, including Creutzfeldt-Jakob disease (CJD), but 14-3-3 protein was undetectable in cerebrospinal fluid and EEG abnormalities were nonspecific. FDG-PET showed elevated bilateral basal ganglia signal of unknown significance.

Molecular analysis revealed the very rare G114V mutation on one allele of the PrP gene. This mutation has been described in a handful of CJD cases/families across continents and ethnicities worldwide. Dying at age 36, the patient had an unusually long disease duration, but brain autopsy was not performed.

Genetic family study indicates incomplete penetrance of this mutation as the patient´s father, also with G114V, has remained asymptomatic to this day at age 74.

Pathogenic causality and structural implications of the G114V mutation will be discussed along with the issue of incomplete penetrance in genetic prion diseases.

**Structure and dynamics of alpha-synuclein interaction with fibrillary seeds**

Min Wu, and Maria Stepanova

Electrical & Computer Engineering Department, University of Alberta, Edmonton, Canada

**Aims**: Alpha-synuclein (α-syn) is a protein commonly found in the nervous system. At normal conditions α-syn adopts disordered unfolded conformations, although it also can form α-helices upon binding to lipid membranes. Under conditions that are not yet fully understood α-syn misfolds and aggregates giving rise to β-sheet rich amyloid fibrils, which tend to accumulate in the body. This leads to Parkinson’s disease (PD) and several other conditions collectively termed synucleinopathies. Development of disease-modifying treatments requires detailed understanding of structure and dynamics of α-syn’s misfolded aggregates and fibrils.

**Material and Methods**: We have employed extensive all-atom molecular dynamics (MD) simulations to investigate the interaction of both unstructured and α-helical monomeric α-syn_38-95_ fragments, which contain the most important amyloidogenic regions, with pre-formed fibrillary seed composed of five staggered, β-sheet rich α-syn chains of matching length. We have considered both disordered and α-helical initial conformations of α-syn_38-95_ monomers. We have applied our original essential collective dynamics (ECD) method to analyze dynamical stability of the α-syn aggregates.

**Results**: The simulations indicate that the two differently structured α-syn_38-95_ monomeric fragments tend to form aggregates with the fibrillary seeds, although we have not observed alignment of the monomeric chains with β-strands of the fibril. The ECD analysis revealed extensive dynamical coupling across the initially monomeric α-syn chains and the core of the fibrillary seed including distal regions of the fibril that have not contacted the monomer directly. This includes changes in the structure and dynamics of remote regions of the fibril.

**Conclusions**: The observed long-range dynamical coupling across α-syn monomeric fragment and the core of the fibrillary seed suggests that the propensity of the fibril to elongate might be mediated by binding of small molecules or other ingredients to a broad range of locations at the surface of the fibril. In particular, C-terminal regions of α-syn_38-95_ chains of the fibril might be a promising target for anti-PD therapeutic interventions.

**Funded by**: Alberta Innovates

**Grant number**: 20170016

**Acknowledgement**: The authors would like to thank Dr. Lyudmyla Dorosh for helpful discussions of the work.

**Uncoupling of Aβ load and neurodegeneration in APP transgenic mouse model**

Ying Xu^a,b,c,^^*^, Christine Rother^a,b,c,^^*^, Ruth E. Uhlmann^a,b,c^, Stephan A. Müllerd^e^, Juliane Schelle^a,b,^ Angelos Skodras^a^, Ulrike Obermüller^a,b^, Lisa M. Häsler^a,b^, Marius Lambert^a,b,^ Frank Baumann^a,b,^ Carina Bergmann^a,b,c^, Irena Brzak^f^, Derya Shimshek^f^, Ulf Neumann^f^, Stephan A. Kaeser^a,b^, Stefan F. Lichtenthalerd^e,g^, Lary C. Walker^h^, and Matthias Staufenbiel^a^, and Mathias Jucker^a,b^

^a^Department of Cellular Neurology, Hertie Institute for Clinical Brain Research, University of Tübingen, Tübingen, Germany; ^b^German Center for Neurodegenerative Diseases (DZNE), Tübingen, Tübingen, Germany; ^c^Graduate School of Cellular and Molecular Neuroscience, University of Tübingen, Tübingen, Germany, ^d^German Center for Neurodegenerative Diseases (DZNE), Munich, Germany; ^e^Neuroproteomics, School of Medicine, Klinikum rechts der Isar, Technische Universität München, Munich, Germany; ^f^Neuroscience, Novartis Institutes for Biomedical Research, Basel, Switzerland; ^^g^^Munich Cluster for Systems Neurology (SyNergy), Munich, Germany; ^h^Department of Neurology and Emory National Primate Research Center, Emory University, Atlanta, GA

**Aims**: Genetic, pathologic and biochemical data support a primary role of amyloid-β (Aβ) aggregation in Alzheimer’s disease (AD), but clinical trials of agents targeting Aβ have not revealed robust clinical benefit. These observations are in line with the view that Aβ aggregation is the trigger of AD, but that the pathogenic cascade becomes independent of Aβ load at later and symptomatic stages. To elucidate the dependency of downstream pathologies on Aβ, we analysed brain Aβ load, Aβ seeding activity, and neurofilament light chain protein (NfL, a presumed marker of neurodegeneration) in the cerebrospinal fluid (CSF) in transgenic mice expressing AD-mutant Aβ-precursor protein (APP) and presenilin-1 (PS1) at different disease stages.

**Material and Methods**: To block Aβ generation, APPPS1 mice were treated with an inhibitor of β-site APP-cleaving enzyme 1 (BACE1) either short-term or long-term at different ages. Aβ and NfL were measured by electrochemiluminescence-linked immunoassay (Meso Scale Discovery) and/or the Simoa platform (Quanterix). Aβ seeding activity was estimated using a well-established in vivo endpoint titration assay. Brain tissues were examined postmortem by routine immunohistological analysis.

**Results**: In APPPS1 mice Aβ deposition increases linearly until it reaches a plateau at a late age. In contrast, Aβ seeding activity increases more rapidly and reaches a plateau much earlier. A robust increase of CSF NfL was observed only after Aβ seeding activity had plateaued. Inhibition of Aβ generation in amyloid-laden mice reduced Aβ deposition, but failed to reduce Aβ seeding activity, and CSF NfL continued to increase. When Aβ generation was inhibited starting at pre-amyloid stages, CSF NfL no longer increased despite some Aβ deposition and robust Aβ seeding activity.

**Conclusions**: Our data indicate that neurodegeneration (as assessed by CSF NfL) starts when Aβ seeding activity is saturated, a phenomenon reminiscent of the two pathogenic phases in prion disease. Blocking Aβ deposition in AD is likely to be most beneficial at a much earlier time-point than that targeted in past clinical trials.

**Funded by**: Cure Alzheimer’s Foundation; the EU/EFPIA/Innovative Medicines Initiative (2) Joint Undertaking (IMPRiND); German Research Foundation; Alexander von Humboldt Foundation; the Chinese Scholarship Council.

**Acknowledgement:** We thank Carina Leibsle, Jörg Odenthal (Tübingen), Anna Berghofer (Munich), and all the other members of our departments for experimentalhelp.

**Pathological alpha-synuclein profiling in nasal specimens of patients with Parkinson’s disease**

Mary Xylaki^a^, Michael Bartl^a^, Jonas Franz^b^, Michael Schlossmacher^c^, Christine Stadelmann^b^, and Brit Mollenhauer^a,d^

^a^Department of Neurology, University Medical Center Goettingen, Goettingen, Germany; ^b^Institute of Neuropathology, University Medical Center Göttigen, Göttingen, Germany; ^c^Division of Neurology, Department of Medicine, Ottawa Hospital, University of Ottawa, Ottawa,Canada; ^d^Paracelsus-Elena-Klinik, Kassel, Germany

**Aims**: Parkinson’s disease (PD) is an increasingly prevalent neurodegenerative disorder for which diagnosis is based on clinical criteria that can be difficult to interpret and distinguish from other parkinsonian syndromes. Biomarkers such as pathological alpha-synuclein (asyn) detection are established in cerebrospinal fluid (CSF) collected by lumbar puncture. The underlying pathogenesis of PD is still unclear, but a peripheral origin is discussed, and some PD is thought to start in the olfactory bulb which is connected to the nose, rendering easily accessible nasal samples potent for biomarker development. We sought to identify whether asyn seed amplification assay developed to detect pathological asyn in CSF using samples from PD patients and healthy controls could detect pathological asyn in nasal lavage samples and olfactory mucosa to be used as a biomarker.

**Material and Methods**: In this study, olfactory mucosa and nasal lavage samples were collected from PD patients recruited at the Paracelsus-Elena-Klinik, Kassel, Germany (DeNoPa Cohort) and from controls free of neurological disease. Samples were analysed using seed amplification assay and their seeding ability was compared to that of CSF samples.

**Results**: The asyn seed amplification assay activity in olfactory mucosa and nasal lavage samples from PD patients compared to the controls indicated the specificity and sensitivity of these samples. In addition, accuracy among results of asyn seed amplification assay activity for CSF, olfactory mucosa and nasal lavage from the same patient was estimated. **Conclusions**: Our results suggest that asyn seed amplification assay analysis of nasal samples alone or combined with CSF testing are useful for increasing the diagnostic accuracy of PD. Finally, more research is necessary to establish the use of the assay in peripheral samples as a biomarker to detect the disease earlier and monitor progression and response to disease modifying approaches.

**Funded by**: ASAP Grant number:020625

**Structural and Kinetic Characterization of Disease Associated Tau Mutants**

Allan Yarahmady^a,b^, Jónathan Heras^c^, and Sue-Ann Mok^a,b^

^a^Biochemistry, University of Alberta, Edmonton, Canada; ^b^Centre for Prions and Protein Folding Diseases, University of Alberta, Edmonton, Canada; ^c^Department of Mathematics and Computer Sciences, University of La Rioja, Logrono, Spain

**Aims**: While great advancements have been made in the field of tau biology, one particularly elusive component is the significance of structural variance of tau aggregates linked to disease: we see great structural heterogeneity among the aggregates obtained from various tauopathies despite being made from the same protein. Furthermore, these profiles appear to be consistent across patients with a given disease. This suggests disease-specific cellular triggers that favour certain aggregate structures over others. Point mutations in tau, such as those identified in FTLD-tau, can have marked effects on aggregate kinetics, structure, and morphology. Probing the effect these mutations have on aggregate structure could give us a better understanding of these differences and bring us closer to defining the critical early stages of disease onset and spread.

**Material and Methods**: Thirty-six disease associated tau mutants were recombinantly expressed and purified using an optimized small-scale purification protocol yielding pure tau. The kinetics of aggregation were studied utilizing heparin as an accelerant and monitoring by thioflavin T fluorescence in a time-course assay. Resultant aggregates were characterized by core structure using trypsin digestion and visualization by capillary gel electrophoresis.

**Results**: Most mutants studied exhibited WT-like aggregation kinetics. Mutants which delayed aggregation tended to be outside the region comprising the aggregate core (R2 and R3) and those which increased aggregation tended to be inside that region. Trypsin digestion revealed ten structural subtypes the mutants fell into as indicated by altered protease resistant banding patterns.

**Conclusions**: Certain tau mutants associated with disease exhibit variable structure and kinetic profiles. A deep mutational scan of tau generating a library of mutants could elucidate the connection between sequence and aggregate structure as well as its impact of aggregation kinetics.

**Funded by**: Alberta Prion Research Institute, Alzheimer’s Society of Alberta and Northwest Territories

**Acknowledgements**: Dr. David Westaway

**Interactome remodeling of prion/prion-like proteins in response to oxidative stress**

Neelam Younas ^a,b^, Saima Zafar ^a,b,c^, Tayyaba Saleem^a,b^, Leticia C. Fernandez^a,b^, Matthias Schmitz^a,b^, and Inga Zerr^a,b^

^a^Department of Neurology, Georg-August University, Goettingen, Germany; ^b^German Center for Neurodegenerative Diseases (DZNE), Göttingen, Germany, ^c^Biomedical Engineering and Sciences Department, School of Mechanical and Manufacturing Engineering (SMME), National University of Sciences and Technology (NUST), Islamabad, Pakistan

**Aims**: Main aim of the current study is to characterize stress-induced network rearrangements of prion/prion-like proteins (PrP, tau and synuclein) under basal and stressful conditions. Emerging evidence indicate that dysregulation of stress response lead to misfolded protein aggregation in the pathophysiology of many neurodegenerative diseases. Gaining insights into the molecular pathways associated with prion/prion-like proteins (PrP, tau and synuclein) under stressful conditions is critical for understanding pathological cascades linked to these proteins.

**Material and Methods**: To find out interacting partners and their posttranslational modifications associated with bait proteins at physiologically relevant levels in the absence of cross-linking agent, endogenous, native, and untagged proteins were co-immunoprecipitated. We employed mass spectrometry analysis to identify the interactomes of tau, synuclein and PrP, representing 30 biological samples (10 samples/bait protein). Multiple biochemical approaches were used for validations including co-immunoprecipitaion, co-immunofluorescence, immunoblotting and subcellular fractionation.

**Results**: In total, 597 proteins passed our cutoff criteria and were classified as potential interactors. Our comparative interactome maps provide comprehensive network rearrangements of three aggregation-prone proteins, identify novel interacting components, their post-translational modifications (PTMs) and validate interactions with target proteins. In addition, we discovered dysregulation of PrP, exportin-5, and translocation of exportin-5 into nucleus under stressful condition in vitro, highlighting remodeling of nucleocytoplasmic transport during stress response.

**Conclusions**: Overall, using protein-protein interaction networks, current study broadens the understanding of the pathobiological context of known neurodegenerative disease-associated proteins. We have established a new category of interest (stress mediated PTMs) for exploration. We hope further studies of prion/prion-like proteins and stress granule biology; and their relation to DNA and RNA processing will further elucidate the mechanisms of neurodegeneration linked to these proteins.

**Funded by**: Supported by Helmholtz-Alberta Initiative – Neurodegenerative Diseases Research (HAI-NDR); Alberta innovates Bio solutions, and DZNE Goettingen.

**Early preclinical proteomic signatures of prion infection**

Neelam Younas^a,b^, Saima Zafar ^a,b,c^, Matthias Schmitz^a,b^, Niccolò Candelise^a,b^, Maria Cramm^a,b^, Susana Correia^a,b^, Olivier Andréoletti^d^, and Inga Zerr^a,b^

^a^Department of Neurology, Georg-August University, Göttingen, Germany;^b^German Center for Neurodegenerative Diseases (DZNE), Göttingen, Germany), ^c^Biomedical Engineering and Sciences Department, School of Mechanical and Manufacturing Engineering (SMME), National University of Sciences and Technology (NUST), Islamabad, Pakistan;^d^Institut National de la Recherche Agronomique/Ecole Nationale Vétérinaire, Toulouse, France

**Aims**: Main aim of the current study is to find out early preclinical signatures of prion infection. Prion diseases characteristically have long preclinical incubation periods during which the pathological prions and infectivity gradually propagate in the brain. The earliest molecular changes that accompany neuronal damage and ultimately lead to neuronal death remain enigmatic. In the current study, we employed RT-QuIC and proteomics analysis to find out initial seeding and temporal proteomic response in prion-infected mice.

**Material and Methods**: We inoculated intracerebrally (i.c.) human sCJD-MM1 and sCJD-VV2 brain homogenate in tg340 and tg361 mice (expressing about four fold of human PrP MM129 and VV129 respectively) and non-infectious brain homogenate (control) as well. Mice were sacrificed at 60, 120, 160 and 180 (days post inoculation), corresponding to early preclinical, late preclinical, early clinical and late clinical. To find out the time of initial prion seeding during prion infection, we investigated the seeding activity of prion protein temporally using RT-QuIC. Next, to identify molecular changes associated with initial prion seeding events and how they change with the progression of the disease, we performed time-dependent proteomics analysis (SWATH-MS).

**Results**: Interestingly, positive RT-QuIC responses were seen already at early preclinical stage (60 dpi) in both CJD-MM1 and VV2 inoculated mouse lines. The fluorescence threshold was 10,000 rfu, which was the basis of determining positive RT-QuIC response. RT-QuIC reactivity was increased (THT fluorescence intensity) gradually from early preclinical stage to late clinical stage in MM1-inoculated mice. For sCJD-VV2, inoculated mice reactivity was increased from early preclinical stage to early clinical stage but was decreased at late clinical stage. The differential expression analysis revealed 940 proteins that were differentially expressed between diseased and control mice. Proteomic alterations were already evident at early preclinical time point. The most prominent finding in our analysis was that proteomic alterations followed a strictly temporal pattern and were subtype-specific. Time-dependent functional profile showed significant alterations in synaptic transmission, abnormal cognition and defects in neuronal cytoskeleton at preclinical stages. Derangement in neurofilament-cytoskeleton, exocytosis and innate immune system were the most prominent terms at clinical stages.

**Conclusions**: In summary, we found a differential seeding activity during the prion infection, with detection of seeding already at early preclinical time point (60 dpi). Our work provides an insight of temporal proteomic alterations that accompany prion infection. Further investigations of these proteomic dysregulations will provide an inroad for the study of early biomarkers and pathological mechanisms.

**Acknowledgement**: We thank Dr. Christof Lenz for helping in bioinformatics analysis.

**Quantitative measurements of chronic wasting disease prions recovered from swab samples and environmentally relevant surfaces**

Qi Yuan^a^, Gage Rowden^b^, Tiffany M. Wolf^c^, Marc D. Schwabenlander^b^, Peter A. Larsen^b^, Shannon L. Bartelt-Hunt^d^ and Jason C. Bartz^a^

^a^Department of Medical Microbiology and Immunology, Creighton University, Omaha, Nebraska; ^b^Department of Veterinary and Biomedical Sciences, University of Minnesota, Saint Paul, MN, USA ^c^Department of Veterinary Population Medicine, University of Minnesota, Saint Paul, MN; ^d^Department of Civil and Environmental Engineering, Peter Kiewit Institute, University of Nebraska-Lincoln, Omaha, Nebraska, USA

**Aims**: To develop a novel method for extracting chronic wasting disease (CWD) prions from swabs and, in combination with ultrasensitive detection methods, detecting CWD prions recovered from environmentally relevant surfaces at low levels.

**Material and Methods**: Brain tissues collected from hamsters infected with hyper strain (HY) transmissible mink encephalopathy (TME) or from elk infected with CWD were used. Foam-tipped and cotton-tipped swabs, and environmental relevant surfaces including stainless steel sheets, microscope glass slides, and oak wood coupons were contaminated with brain homogenates and dried at 22°C for different lengths of period up to 24 hours followed with extraction by shaking or sonication. Extracts were vacuum concentrated and analyzed with 96-well immunodetection and/or real-time quake-induced conversion (RT-QuIC).

**Results**: Drying on swabs decreased prion recovery with shaking extraction. However, sonication improved the recovery of swab-dried prions. Using the developed swab extraction technique by sonication, the recovery of CWD prions dried to glass or stainless steel was approximately 30% in most cases, whereas that from wood was undetectable by 96-well immunodetection. RT-QuIC analysis of CWD prions recovered from stainless steel sheets resulted in an increase of the detection limit by 4 orders of magnitude. More importantly, the RT-QuIC detection of CWD prions recovered from stainless steel surfaces using this method was similar to the original CWD prion load applied to the surface.

**Conclusions**: We developed a novel swab-extraction procedure for field deployable sampling of CWD prions from stainless steel, glass, and wood. Extended swab-drying was unfavorable for prion extraction, indicating that hydrated storage of swabs after sampling aided in prion recovery. This combined surface swabbing with sonication extraction and RT-QuIC detection method provides an ultrasensitive means for prion detection across many settings and applications.

**Funded by**: National Institutes of Health, National Science Foundation

**Grant number**: NIH: P01 2P01AI077774 to J.C.B., NSF: CBET-1149424 to S.L.B.

**Acknowledgement**: We thank Drs. Nicholas Haley and Ken Clinkenbeard for providing CWD-infected tissues.

**Prion-like characteristics of Amyloid-β deriving clinical variants of Alzheimer’s disease**

Saima Zafar^a,b^, Aneeqa Noor^a,b^, Mohsin Shafiq^a,c^, Neelam Younas^a^, Anna Siegert^a^, Florian A Manna^d^, Sebastian Kruss^d^, Matthias Schmitz^a^, Hassan Dihazi^e^, Isidre Ferrer^f^, and Inga Zerr^a^

^a^Department of Neurology, University Medical Center Goettingen, Goettingen, Germany; ^b^Department of Neuropathology, University Medical Center Eppendorf, Hamburg, Germany;^c^ Institute of Neuropathology, University Medical Center Hamburg-Eppendorf, Hamburg, Germany; ^d^Institute of Physical Chemistry, Georg-August University, Tammannstraße 6, 37,077, Göttingen, Germany; ^e^Department of Nephrology and Rheumatology, Georg-August University, University Medical Center Göttingen, Robert-Koch-Straße 40, 37,075, Göttingen, Germany; ^f^Department of Pathology and Experimental Therapeutics, University of Barcelona CIBERNEDBellvitge University Hospital (IDIBELL), Carrer de la Feixa Llarga, 08907, Hospitalet de Llobregat, Spain

**Aims**: The molecular determinants of atypical clinical variants of Alzheimer’s disease, including the recently discovered rapidly progressive Alzheimer’s disease (rpAD), are unknown to date. Fibrilization of the amyloid-β (Aβ) peptide is the most frequently studied candidate in this context. The Aβ peptide can exist as multiple proteoforms that vary in their post-translational processing, amyloidogenesis, and toxicity.

**Material and Methods**: The current study was designed to identify these variations in Alzheimer’s disease patients exhibiting classical (sAD) and rapid progression, with the primary aim of establishing if these variants may constitute strains that underlie the phenotypic variability of Alzheimer’s disease. We employed two-dimensional polyacrylamide gel electrophoresis and MALDI-ToF mass spectrometry to validate and identify the Aβ proteoforms extracted from targeted brain tissues. The biophysical analysis was conducted using RT-QuIC assay, confocal microscopy, and atomic force microscopy. Interactome analysis was performed by co-immunoprecipitation.

**Results**: We present a signature of 33 distinct pathophysiological proteoforms, including the commonly targeted Aβ40, Aβ42, Aβ4-42, Aβ11-42, and provide insight into their synthesis and quantities. Furthermore, we have validated the presence of highly hydrophobic Aβ seeds in rpAD brains that seeded reactions at a slower pace in comparison to typical Alzheimer’s disease. In vitro and in vivo analyses also verified variations in the molecular pathways modulated by brain-derived Aβ.

**Conclusions**: These variations in the presence, synthesis, folding, and interactions of Aβ among sAD and rpAD brains constitute important points of intervention. Further validation of reported targets and mechanisms will aid in the diagnosis of and therapy for Alzheimer’s disease.

**Funded by**: Open Access funding enabled and organized by Projekt DEAL. This research was partially funded by the Physics‐to‐Medicine Initiative Göttingen (LM der Niedersächsischen Vorab)

**Serpins in prion diseases**

Marco Zattoni^a,*^, Arianna Colini Baldeschi^a,*^, Silvia Vanni^a,b^, Marika Mearelli^a,c^, Thanh Hoa Tran^a,d^, Chiara Ferracin^a^, Lea Nikolic^a^, Marcella Catania^b^, Fabio Moda^b^, Giuseppe Di Fede^b^, Giorgio Giaccone^b^, Fabrizio Tagliavini^c^, Gianluigi Zanusso^d^, James W. Ironside^e^, Isidre Ferrer^f^, Giuseppina La Sala^g, 5^, Maria Summa^h^, Rosalia Bertorelli^h^, Sine Mandrup Bertozzi^i^, Paolo Carloni^J^, Maria Laura Bolognesi^k^, Marco De Vivo^g^and Giuseppe Legname^a^

^a^Laboratory of Prion Biology, Department of Neuroscience, Scuola Internazionale Superiore di Studi Avanzati (SISSA), Trieste, Italy; ^b^Fondazione IRCCS Istituto Neurologico Carlo Besta, Division of Neurology 5 and Neuropathology, Milan, Italy; ^c^Fondazione IRCCS Istituto Neurologico Carlo Besta, Scientific Directorate, Milan, Ital; ^d^Department of Neurosciences, Biomedicine and Movement Sciences, University of Verona, Verona, Italy; ^e^National CJD Research & Surveillance Unit, Centre for Clinical Brain Sciences, University of Edinburgh, Edinburgh, UK; ^f^Department of Pathology and Experimental Therapeutics, University of Barcelona; Institute of Biomedical Research of Bellvitge (IDIBELL); Biomedical Research Network Center of Neurodegenerative Diseases (CIBERNED); Hospitalet de Llobregat, Spain; ^g^Molecular Modeling & Drug Discovery Lab, Istituto Italiano di Tecnologia, Via Morego 30, 16,163 Genoa, Ital; ^h^Translational Pharmacology, Istituto Italiano di Tecnologia, Via Morego 30, 16,163 Genoa, Italy; ^i^Analytical Chemistry Lab, Istituto Italiano di Tecnologia, Via Morego 30, 16,163 Genoa, Italy; ^j^Institute for Advanced Simulations (IAS)-5/Institute for Neuroscience and Medicine (INM)-9, Forschungszentrum Jülich, 52,428 Jülich, Germany; ^k^Department of Pharmacy and Biotechnology, University of Bologna, Via Belmeloro 6, 40,126 Bologna, Italy; ^a^current affiliation: Institute of Biomedicine, Department of Pathology and Experimental Therapeutics, Bellvitge University Hospital-IDIBELL, Barcelona, Spain; ^b^current affiliation: Osteoncology Unit, Bioscience Laboratory, IRCCS Istituto Romagnolo Per Lo Studio Dei Tumori (IRST) ‘Dino Amadori’, 47,014 Meldola, Italy; ^c^current affiliation: German Center for Neurodegenerative Diseases (DZNE), Tübingen, 72,076, Germany; ^d^current affiliation: VN-UK Institute for Research and Executive Education, The University of Danang, Da Nang, Vietnam; ^e^current affiliation: Medicinal Chemistry, Research and Early Development, Cardiovascular, Renal and Metabolism (CVRM), BioPharmaceuticals R&D, AstraZeneca, Gothenburg, Sweden.

* these authors contributed equally to this work

**Aims**: Serpins represent the most broadly distributed superfamily of proteases inhibitors. They contribute to a variety of physiological functions and any alteration of the serpin-protease equilibrium can lead to severe consequences. SERPINA3 dysregulation has been associated with prion diseases and Alzheimer’s disease. In this study, we investigated the differential expression of other serpin superfamily members in prion diseases. Furthermore, we analyzed the biochemical activity of its murine orthologue, SerpinA3n, in prion-affected brain tissue. We also modulated SerpinA3n levels, either genetically or pharmacologically, performing *in vitro* experiment in scrapie-infected immortalized cell line, to better investigate its role in prion pathogenesis.

**Material and Methods**: *SERPIN* expression was analyzed, by RT-qPCR, in human frontal cortex samples from cases of sporadic Creutzfeldt-Jakob disease (sCJD) and age-matched controls not affected by neurodegenerative disorders. In addition, we studied whether *Serpin* expression was dysregulated in RML-infected mouse model.

To check SERPINA3/SerpinA3n activity in tissue, mouse brain samples were incubated with a known target protease, chymotrypsin, and SDS-PAGE and Western blot analysis were performed to evaluate the formation of a covalent complex between SerpinA3n and the protease.

Moreover, SerpinA3n-overexpressing cell conditioned medium and recombinant SerpinA3n treatment, on scrapie-infected N2a cells, were performed to investigate changes in prion accumulation levels. Similarly, we evaluated prion load in cells transfected with SerpinA3n-directed siRNA and shRNA and treated with small molecules targeting SerpinA3n.

**Results**: Our analysis revealed that, besides the already observed strong upregulation of *SERPINA3* in patients with prion disease, *SERPINB1, SERPINB6, SERPINE1, SERPING1, SERPINH1* and *SERPINI1* were mild dysregulated in sCJD individuals compared to controls.

Furthermore, we analyzed whether other serpin members were differentially expressed in prion-infected mice compared to controls and, together with *SerpinA3n, SerpinF2* increased levels were observed.

The SerpinA3n increased anti-protease activity found in brain tissue of RML-infected mice suggests its involvement in prion disease pathogenesis. A SERPINA3/SerpinA3n role prion infection was further corroborated by *in vitro* SerpinA3n-dependent prion accumulation changes in chronically-infected cells.

**Conclusions**: SERPINA3/SerpinA3n marked dysregulation in prion diseases and its effect on the prion accumulation process, suggests its consideration as a potential therapeutic target.

Although the low bioavailability of the most potent anti-prion compound targeting SerpinA3n (ARN1468) does not allow *in vivo* studies in infected mice, our strategy emerges as a novel and effective approach to the treatment of prion disease. Further analyses in other neurodegenerative disorders are needed to understand whether the neurodegenerative mechanism is SERPINA3/SerpinA3n-dependent or whether other serpin superfamily members are involved in these pathological processes.

**Funded by**: Intramural SISSA Funding, Helmholtz Partnering Project on ‘Innovative high-performance computing approaches for molecular neuromedicine’.

**Acknowledgement**: The authors wish to thank Professor Remo Sanges for assistance with data source analysis, Helena Krmac, Christina Vlachouli and Nicoletta Brindani for technical support. Paolo Carloni and Marco De Vivo acknowledge financial support from the Helmholtz Society for this project. The authors acknowledged SISSA intramural grant support for carrying out the study.

**Engineered zinc finger protein transcription factors potently reduce brain PrP expression and extend survival in prion-infected mice**

Bryan Zeitler^a^, Meredith A Mortberg^b^, Mohad Mehrabian^a^, Kimberly Marlen^a^, Shih-Wei Chou^a^, Michael Howard^b^, Samantha Graffam^b^, Kenney Lenz^b^, Tyler Caron^b^, Qi Yu^a^, Angelica Phillips^a^, Jing Hu^a^, Sarah Hinkley^a^, Alicia Goodwin^a^, Asa Hatami^a^, Alaric Falcon^a^, Lei Zhang^a^, Kathleen Meyer^a^, Jason Fontenot^a^, Amy M Pooler^a^, Eric Vallabh Minikel^b^, and Sonia M Vallabh^b^

^a^Sangamo Therapeutics, Richmond, CA 94804 USA; ^b^Broad Institute of Harvard and MIT, Cambridge, MA 02142, USA

**Aims**: Prion disease is an invariably fatal and rapidly progressing neurodegenerative disorder caused by aggregation of misfolded prion protein, PrP, encoded by the *PRNP* gene. Most cases are sporadic or caused by inherited dominant mutations in *PRNP*. There are currently no approved or clinical-stage disease-modifying therapies for the prevention or treatment of prion disease. Lowering endogenous PrP levels by 50% either genetically or with antisense oligonucleotides can approximately double the lifespan of prion-infected mice. Moreover, *Prnp* null animals are completely resistant to prion inoculation. We investigated a single-administration AAV approach with zinc finger protein transcription factors (ZF-TFs) as a potential therapeutic strategy to achieve sustained and widespread reduction of PrP in the brain and rapid pharmacological effect.

**Material and Methods**: Engineered ZFPs targeting the transcription regulatory elements of either mouse *Prnp* or human *PRNP* were fused to the human KRAB repression domain and screened in mouse Neuro2A or human SK-N-MC cells. RT-qPCR analysis identified dozens of ZF-TFs that reduced prion mRNA by 50–99%. Selected ZF-TFs potently reduced prion mRNA levels in cultured primary mouse cortical and human iPSC-derived neurons with no detectable off-target activity, as evaluated by transcriptome-wide profiling.

**Results**: ZF-TFs under the control of the human synapsin promoter reduced bulk prion protein mRNA and protein levels by >50% across the brain when administrated to adult wild-type and humanized mice. Multiplexed RNAscope and immunohistochemistry demonstrated a strong negative correlation between ZF-TF and *Prnp* expression at the single-neuron level throughout the brain. Wild-type mice were inoculated with RML prions and treated with a single dose of ZF-TFs at 60 days post inoculation (dpi), near the onset of plasma neurofilament light chain (NfL) rise, or at 122 dpi, near the onset of symptoms. While control groups reached terminal endpoint at 160 ± 8 dpi (mean±sd), 9/19 mice treated with PrP-lowering AAV-ZF-TFs were alive at 400 dpi, with attendant improvements in weight gain, nest-building, and plasma NfL. Notably, increasing plasma NfL levels were arrested abruptly following ZF-TF treatment at both intervention points, suggesting a rapid attenuation of neurodegeneration.

**Conclusions**: ZF-TFs can substantially lower PrP in the brain, extend survival, and alter disease trajectory in a robust prion inoculation mouse mode. These results support the continued development of a genomic medicine for the treatment of prion disease.

**Funded by**: Sangamo Therapeutics, CJD Foundation

**An imaging-based bimolecular fluorescence complementation assay to screen for unconjugated degraders for the cellular prion protein**

Ilaria Zeni^a^, Valerio Bonaldo^a^, Tania Massignan^b^& Emiliano Biasini^a^

^a^Department CIBIO, University of Trento; ^b^Sibylla Biotech S.R.L.

**Aims**: Bimolecular fluorescence complementation (BiFC) has been initially developed to detect protein-protein interactions. In the standard BiFC design, two fragments of a fluorescent protein are individually fused to two potentially interacting proteins. As a consequence of such interaction, the fluorescent protein fragments come to proximity, enabling fluorophore reconstitution (complementation) and leading to a fluorescent signal. Fluorescent protein fragments with an intrinsically high interaction affinity could theoretically allow employing BiFC as a way to artificially bring to proximity two proteins residing in the same intracellular compartment. We aim to develop an imaging-based bimolecular fluorescence complementation assay (BiFC) to rapidly quantify the cellular prion protein’s expression, trafficking, and degradation (PrP) in real-time. The technique will also allow us to screen directly for compounds that promote PrP degradation.

**Material and Methods**: The opportunity for the application of BiFC to study the expression, trafficking, and degradation of PrP is provided by the recent development of a superfolder green fluorescent protein (sfGFP) split into two non-symmetrical halves, one containing the first ten β-strands of the original β-barrel domain (GFP1-10) and the other represented by the single missing β-strand (GFP11) (1). GFP1-10 and GFP11 re-assemble spontaneously, without the need to be fused to interacting proteins. We have attached GFP11 to the C-terminus of PrP and conjugated the GFP1-10 reporter with a signal peptide that restricts expression to the endoplasmic reticulum (ER-GFP1-10). This system could allow monitoring of the expression kinetics of PrP in real-time.

**Results**: We have collected preliminary evidence about the usefulness and feasibility of the GFP1-10/GFP11 system for rapidly monitoring the expression of PrP by live imaging. We have transfected PrP-GFP11 expressed under the control of an inducible promoter into HEK293 recipient cells transduced with a lentiviral vector encoding for an ER-GFP1-10. Imaging analysis revealed a specific intracellular fluorescent signal only when both PrP-GFP11 and ER-GFP1-10 were co-expressed, suggesting that the assay is a viable method to monitor PrP expression.

**Conclusions**: Our results introduced a novel imaging-based paradigm to study PrP expression in real-time. The recent generation of fluorescently diversified GFP1-10 reporters, which could emit at different wavelengths once complemented by the same GFP11 fragment, will allow us to employ different GFP1-10 constructs restricted to specific cellular sites along the expression pathway of PrP, including ER, Golgi, plasma membrane, endosomal-recycling vesicles, and lysosomes. This strategy could provide a unique opportunity for live tracking PrP expression in different experimental conditions.

**References**: 1. Kamiyama et al., Nat Comm 2017.

**Funded by**: Fondazione Telethon, Italy

**Grant number**: GGP20043

**Funded by**: CJD Foundation, USA

**Acknowledgments**: We thank Antonio Casini, Alia Therapeutics S.R.L., for useful advice regarding the generation of lentiviral vectors.

**ASO-mediated PrP suppression as disease modifying therapy for prion disease**

Hien T. Zhao^a^, Deborah Cabin^b^, Jill O’Moore^b^, Eric V. Minikel^c^, Sonia M. Vallabh^c^, and Holly B. Kordasiewicz^a^

^a^Neurology Research, Ionis Pharmaceuticals, Inc., Carlsbad, CA, USA; ^b^McLaughlin Research Institute, Great Falls, MT, USA; ^c^Broad Institute, MIT, Cambridge, MA, USA

**Aims**: To evaluate plasma neurofilament light chain (NfL) response, a disease biomarker of neuroaxonal injury, and survival effects in the RML prion mouse model following treatment with a PrP-lowering antisense oligonucleotide (ASO)

**Material and Methods**: Groups of N = 12 wildtype C57BL/6N mice were inoculated with RML prions and received either 100 µg, 300 µg or 500 µg PrP ASO or saline via CSF delivery at 60 days post infection (dpi), while uninoculated mice were included as controls. These ASO dose levels correspond to roughly 20–60% *Prnp* mRNA knockdown based on prior studies (Minikel et al., 2020). At 60 dpi, plasma NfL is elevated, prion pathology is established and neuroinflammation is increasing; overt behavioral phenotypes typically develop by 125 dpi (Minikel et al., 2020). Plasma NfL was quantified from bleeds taken at 1-day pre-ASO treatment, then every 30 days onward. Animals were followed to the terminal disease endpoint, as defined by 20% weight loss compared to baseline 60 dpi, or death. Paradigms with multiple ASO administrations with varied dosing intervals were also assessed, e.g. 2 administrations 60 days apart, or up to 4 administrations at 60, 90, or 120 days interval. ASO administration at 120 dpi was also evaluated.

**Results**: Plasma NfL levels steadily rose through terminal illness in RML-inoculated mice treated with saline and remained unchanged in uninoculated animals. A dose-dependent delay in plasma NfL rise was observed in RML-inoculated mice treated with a single administration of ASO at 60 dpi, which corresponded to a dose-dependent extension in survival of up to 40%. Administrations of additional ASO further delayed plasma NfL rise and further extended survival in RML-inoculated mice up to 200%. In an RML-inoculated cohort where treatment was initiated at 120 dpi, plasma NfL levels fell significantly in ASO-treated mice compared to the pre-dose time point, suggesting a reversal of pathology driving the 35% – 53% increase in survival time to terminal endpoint.

**Conclusions**: These data demonstrate efficacy of ASO-mediated PrP suppression across varying degrees of *Prnp* mRNA suppression and therapeutic paradigms. Our studies support the use of plasma NfL as a disease biomarker indicative of disease state, as it correlates with disease course in the animal model and with clinical benefit (i.e., survival extension) following ASO-mediated PrP lowering.

**Funded by**: Ionis Pharmaceuticals, Inc.

**Acknowledgement**: The authors thanked Brittany Ford for technical assistance.

